# The extracellular RNA complement of *Escherichia coli*

**DOI:** 10.1002/mbo3.235

**Published:** 2015-01-21

**Authors:** Anubrata Ghosal, Bimal Babu Upadhyaya, Joëlle V Fritz, Anna Heintz-Buschart, Mahesh S Desai, Dilmurat Yusuf, David Huang, Aidos Baumuratov, Kai Wang, David Galas, Paul Wilmes

**Affiliations:** 1 Luxembourg Centre for Systems Biomedicine, University of LuxembourgCampus Belval, 7 avenue des Hauts-Fourneaux, Esch-sur-Alzette, L-4362, Luxembourg; 2 Pacific Northwest Diabetes ResearchSeattle, Washington; 3 Institute for Systems BiologySeattle, Washington

**Keywords:** *Escherichia coli*, extracellular, outer membrane vesicle, RNA

## Abstract

The secretion of biomolecules into the extracellular milieu is a common and well-conserved phenomenon in biology. In bacteria, secreted biomolecules are not only involved in intra-species communication but they also play roles in inter-kingdom exchanges and pathogenicity. To date, released products, such as small molecules, DNA, peptides, and proteins, have been well studied in bacteria. However, the bacterial extracellular RNA complement has so far not been comprehensively characterized. Here, we have analyzed, using a combination of physical characterization and high-throughput sequencing, the extracellular RNA complement of both outer membrane vesicle (OMV)-associated and OMV-free RNA of the enteric Gram-negative model bacterium *Escherichia coli* K-12 substrain MG1655 and have compared it to its intracellular RNA complement. Our results demonstrate that a large part of the extracellular RNA complement is in the size range between 15 and 40 nucleotides and is derived from specific intracellular RNAs. Furthermore, RNA is associated with OMVs and the relative abundances of RNA biotypes in the intracellular, OMV and OMV-free fractions are distinct. Apart from rRNA fragments, a significant portion of the extracellular RNA complement is composed of specific cleavage products of functionally important structural noncoding RNAs, including tRNAs, 4.5S RNA, 6S RNA, and tmRNA. In addition, the extracellular RNA pool includes RNA biotypes from cryptic prophages, intergenic, and coding regions, of which some are so far uncharacterised, for example, transcripts mapping to the *fimA-fimL* and *ves-spy* intergenic regions. Our study provides the first detailed characterization of the extracellular RNA complement of the enteric model bacterium *E. coli*. Analogous to findings in eukaryotes, our results suggest the selective export of specific RNA biotypes by *E. coli*, which in turn indicates a potential role for extracellular bacterial RNAs in intercellular communication.

## Introduction

While growing either in a natural ecosystem or artificial conditions, bacteria secrete intracellular products into their extracellular milieu (Tseng et al. 2009). Secretory products are not only involved in bacterial social behavior, usually referred to as quorum sensing (Molloy 2010) but also in pathogenicity (Lee and Schneewind 2001) and inter-kingdom communication (Hughes and Sperandio 2008; Shen et al. 2012; Furusawa et al. 2013). A large diversity of molecules including small hormone-like autoinducer molecules, peptides, proteins, glycoproteins, and DNA have already been identified as mediators in both intra- and inter-kingdom communication (Waters and Bassler 2005; Molloy 2010; Shen et al. 2012) and in some cases as effectors in pathogenicity (Tseng et al. 2009; Jun et al. 2013). Bacteria disseminate secreted products either through their secretory systems via continuous or discontinuous passages across the bacterial membrane or by the release of outer membrane vesicles (OMVs) (Tseng et al. 2009; Shen et al. 2012). Gram-negative bacteria are also able to export their secretory products into the cytosol of host cells using their penetrating appendages of type III, type IV, and type VI secretion systems, whereas OMV-mediated transport allows bacteria to deliver secretory products remotely without direct contact with host cells (Tseng et al. 2009; Shen et al. 2012; Casson et al. 2013). OMVs may be taken up by host cells and may contribute to pathogenesis, as already demonstrated for OMVs derived from *Helicobacter pylori*, which are taken up by gastric epithelial cells (Parker et al. 2010) and enhance the carcinogenic potential of this specific bacterium (Chitcholtan et al. 2008).

The release of OMVs is a common phenomenon observed for many Gram-negative bacteria, including *Escherichia coli* (Wai et al. 2003; Kesty et al. 2004; Park et al. 2010; Roy et al. 2011). In addition, a recent study has reported that vesicular secretion is also common in Gram-positive bacteria (Thay et al. 2013). Single membrane OMVs comprised outer membrane proteins and periplasmic components (Carmen Schwechheimer and Kuehn 2013), whereas bilayer forms of OMVs also contain inner membrane and cytoplasmic constituents (Pérez-Cruz et al. 2013). In terms of nucleic acids, the DNA content of OMVs has been characterized for *Shewanella vesiculosa*, *Neisseria gonorrhoeae*, and *Pseudomonas aeruginosa* (Dorward et al. 1989; Renelli et al. 2004; Pérez-Cruz et al. 2013). So far, no studies have reported OMVs produced by heterotrophic bacteria to contain RNA, except a suggestion in one study (Dorward et al. 1989). A very recent report, however, has identified RNA in OMVs released by marine photoautotrophs (Biller et al. 2014). Eukaryotic cells also secrete cellular products by shedding of extracellular vesicles (Théry et al. 2002; El Andaloussi et al. 2013), which are analogue to OMVs. Vesicles derived from mammalian cells have recently been extensively characterized for their RNA content, since the RNA is thought to be involved in intercellular regulation (Kogure et al. 2011; Chen et al. 2012; Hoen et al. 2012). Similarly, fungal extracellular vesicles are also known to contain RNA (Nicola et al. 2009).

RNA secreted by bacteria may play important roles in microbe–microbe and host–microbe interactions. In this study, we aimed to characterize, using a combination of physical characterization and high-throughput sequencing, the extracellular RNA complement (both OMV-associated and OMV-free) of the enteric Gram-negative bacterium *E. coli* K-12 substrain MG1655 and compared it to its intracellular RNA complement.

## Materials and Methods

### Bacterial strain, extraction of OMVs, and OMV-free supernatant

OMVs were derived from the enteric Gram-negative bacterium *E. coli* K-12 substrain MG1655. Bacteria were grown in Luria-Bertani (LB) broth or M9 minimal medium at 37°C. OMVs were isolated as previously described (Wai et al. 2003) with slight modifications to the protocol as detailed below. Media were inoculated with a single bacterial colony and grown until early stationary phase. The bacterial cultures were then centrifuged at 4700*g* for 30 min at 4°C. After centrifugation, the supernatants were separated from the cell pellets and concentrated using a tangential flow filtration device (QuixStand Benchtop System; GE HealthCare) with a 100 kDa hollow fiber membrane (GE HealthCare Europe Gmbh, Freiburg, Germany). The concentrated supernatants were then further passed through a 0.8/0.2 *μ*m filter (PALL Life Sciences, Ann Arbor, MI, USA). As a control, filtered supernatants were checked for bacterial contamination by plating 1 mL of supernatant on a LB-agar plate followed by overnight incubation at 37°C. The filtered supernatants were then ultracentrifuged at 150,000*g* for 3 h at 4°C in order to separate the bacteria-free media into an OMV containing fraction (pellet) and OMV-free fraction (supernatant). Thus, OMVs were contained in the pellet fractions, which were then washed with Phospate buffered saline (PBS) (1X), and finally resuspended in PBS and stored at −80°C. The OMV-depleted supernatants were also stored at −80°C for extraction of RNAs. Unless otherwise stated, all experiments were performed with samples prepared by the method described above.

The resulting OMV fractions were then further separated into low- and high-density fractions (HDFs) by gradient ultracentrifugation as previously described (Horstman et al. 2000) with slight modifications as detailed below. In brief, the OMV fractions were precisely adjusted to a concentration of 45% Optiprep (Sigma-Aldrich, Diegem, Belgium) in 2 mL of volume and layered at the bottom of ultracentrifugation tubes, followed by 2 mL of 40%, 35%, 30%, 25%, and 20% Optiprep layered on top of the sample in sequence. The prepared tubes were then subjected to ultracentrifugation at a speed of 100,000 for 16 h at 4°C. After centrifugation, 12 fractions were collected from the top (fractions were labeled 1 to 12 according to the order of collection). Fractions 2–6 (termed the low-density fraction, LDF) and 7–12 (termed the high-density fraction) were pooled for subsequent RNA extractions.

Similarly, OMV and OMV-free fractions were also obtained from exponentially growing bacteria in LB up to ODs between 0.4 and 0.5.

### Transmission electron microscopy

OMV samples were fixed in 2.5% glutaraldehyde and 2.5% paraformaldehyde in PBS (1X) and washed with PBS. Samples were placed on Formvar-coated nickel grids, air dried, and examined at 80 kV with a Morgagni 268D transmission electron microscope. Images were captured digitally by a Mega View III camera (Olympus Soft Imaging solutions Gmbh, Münster, Germany).

### Extraction of proteins and SDS-PAGE electrophoresis

Protein samples were dissolved in lysis buffer (25 mmol/L of 4-(2-hydroxyethyl)-1-piperazineethanesulfonic acid (HEPES) pH 7.4, 5 mmol/L of Ethylenediaminetetraacetic acid (EDTA), 1 mmol/L of Dithiothreitol (DTT), 1 mg/mL of Lysozyme) and passed through three cycles of freezing and thawing. Finally, MgSO_4_ was added to the sample and incubated on ice for 30 min. Samples were centrifuged at a speed of 14,000*g* for 30 min at 4°C. The supernatant was then separated from the samples and used for protein analyses.

Protein concentrations were determined using a Qubit Protein Assay Kit (Invitrogen/Life Technologies Europe: Ghent, Belgium) and separated on a 4–15% of polyacrylamide gel (Bio-Rad Laboratories N.V, Temse, Belgium). Gels were stained with either Imperial Protein Stain (Thermo Scientific/Piercenet, Erembodegem-Aalst, Belgium) or the SilverQuest™ Silver Staining Kit (Invitrogen/Life Technologies Europe: Ghent, Belgium).

### Extraction of RNAs and RNAseq

RNA was extracted from *E. coli* cells, OMVs, OMV-free supernatant, low-density OMV fractions (fractions 2–6), high-density OMV fractions (fractions 7–12), and culture media using the All-in-One Purification Kit (Norgen Biotek/LabOmics, Nivelles, Belgium). Samples were precipitated with 20% (w/v) trichloroacetic acid (TCA) followed by 80% (v/v) acetone washing before the extraction of RNA (Bauman and Kuehn 2006). Total RNA from each sample was collected in two fractions, the large RNA fraction included RNA of the size greater than 200 nucleotides in length and the small RNA fraction (“microRNA” fraction) contained RNAs shorter than 200 nucleotides in length. Extracted RNAs were treated with DNase I (Thermo Scientific) to remove genomic DNA contamination. The size distributions of RNAs in both the large and small RNA fractions were determined using a Bioanalyzer (Agilent Technologies, Diegem, Belgium) instrument with the Agilent RNA 6000 Nano Kit and Agilent Small RNA kit (Agilent Technologies), respectively. The quantity of extracted RNA for the individual fractions ranged from 500 ng to 3 *μ*g.

The RNAseq deep-sequencing library was prepared from the small RNA fractions according to a previously described protocol (Wang et al. 2012), and the library was sequenced using a single-end sequencing strategy on an Illumina (San Diego, California, USA) Genome Analyzer where the maximum read length was 50 nt.

### Bioinformatic analysis

Raw small RNA sequence libraries of different samples were subjected to preprocessing for quality control and adapter removal using the FastX toolkit (http://hannonlab.cshl.edu/fastx_toolkit/). A primer contamination table was generated by analyzing the sequence libraries. Contaminant adapters (RNA 3′ adapter and RNA PCR primer) were subsequently trimmed using the FastX toolkit (http://hannonlab.cshl.edu/fastx_toolkit/). This step was repeated three times in order to achieve a high accuracy for adapter trimming. Following adapter trimming, the sequence reads were subjected to quality trimming to remove noise using a Phred quality scores threshold of 25, which corresponds to an error probability of 0.003 per base. In addition, sequence reads failing to retain all bases with Phred quality score of 25 per base throughout the read were removed. Reads shorter than 14 nucleotides were removed. Identical read sequences after adapter trimming and quality filtering were then collapsed while maintaining the count of the identical reads in the library. Finally, for further analyses of the sequence libraries, identical reads present in the libraries obtained from cell-free growth media and cell-free growth media incubated overnight were subtracted from the reads in all libraries.

Processed read libraries were mapped to the *E. coli* strain K-12 substrain MG1655 reference genome (accession number: NC_000913.2) from the RefSeq database (http://www.ncbi.nlm.nih.gov/refseq/) using novoalign (http://www.novocraft.com) with zero mismatches allowed. Unmapped reads from the library were filtered using SAMtools (Li et al. 2009). Reads mapped to more than one genomic location were given proportional weights using the strategy and algorithm described in De Hoon et al. (2010). Mapped reads were annotated using BEDTools (Quinlan et al. 2010) the gene feature file of *E. coli* strain K-12 substrain MG1655 available from the RefSeq database.

### Staining of OMVs and confocal laser scanning microscopy

OMVs were stained as described previously (Nicola et al. 2009) with slight modifications detailed below. Briefly, DNase I-treated OMV samples were incubated with 1 *μ*mol/L of SYTO® RNASelect™ Green Fluorescent Cell Stain (Life Technologies) and 5 *μ*mol/L of red fluorescent Lipophilic Tracer DiD (Life Technologies) for an hour at 37°C. Samples were washed once in PBS (1X) followed by centrifugation at a speed of 150,000*g* for 1 h at 4°C and mounted onto a glass slide with mounting medium, Fluoroshield™ (Sigma).

Confocal images were acquired on a laser scanning confocal microscope (LSM Zeiss 710) and processed using the ZEN software package (Zeiss, Zaventem, Belgium). The background was adjusted with dye and unstained control samples. For each sample, images were taken separately in rapid succession. The excitation and emission spectra were set at a wavelengths of *λ*_638nm_ and *λ*_700nm_ for DID, and *λ*_490nm_ and *λ*_530nm_ for SYTO RNASelect, respectively. Images were adjusted for brightness.

### RT-qPCR

Equal amounts of DNase I-treated RNA were polyadenylated and converted to cDNA using the qScript microRNA cDNA Synthesis Kit (Quanta BioSciences, Gaithersburg, MD, USA). About 1 ng of cDNA was amplified in combination with sequence-specific forward primers such as: tRNA-Asn: GTATGTCACTGGTTCGAGTC, 23S rRNA: GTTAAGCGACTAAGCGTACAC, tRNA-met: TGCTCTAACCAACTGAGCTAC, tRNA-Thr: CTCAGTAGGTAGAGCAACTG, tRNA-Ile: CTTGTAGCTCAGGTGGTTAGAG, tRNA-His: CTATAGCTCAGTTGGTAGAG, tRNA-Val: TCGAGTCCACTCGGACGCAC, tmRNA: GCTGATTCTGGATTCGACG, 4.5S RNA: CTCTGTTGGTTCTCCCGCAAC or 6S RNA: TCTCTGAGATGTTCGCAAGC (obtained from Eurogentec) and PerfeCTa Universal PCR Primer (Quanta BioSciences) in a total volume of 10 *μ*L of reaction mixture where primers were added at a concentration of 0.2 *μ*mol/L, 5 *μ*L of PerfeCTa SYBR Green SuperMix (Quanta BioSciences) and the remaining volume was adjusted for cDNA amount and water. Primer efficiency was calculated using a 10-fold dilution series of the template cDNA, and for all primer sets efficiency values were close to 100%. Real-time PCR was run on a LightCycler® 480 Real-Time PCR System (Prophac/Roche, Luxembourg-Howald, Luxembourg) using the thermal cycles of 95°C for 2 min and 40 cycles of 95°C for 10 sec, 57–62°C for 5 sec, and 72°C for 10 sec. C_t_ values were obtained using automatic baseline and threshold settings provided by the LightCycler® 480 Software, Version 1.5(Prophac/Roche, Luxembourg-Howald, Luxembourg). Data were analyzed manually using the comparative C_T_ method (ΔΔC_T_). Individual targets were analyzed in three biological replicates and represented as a mean.

## Results and Discussion

### *Escherichia coli*-derived OMVs contain RNA

**Figure 1:**
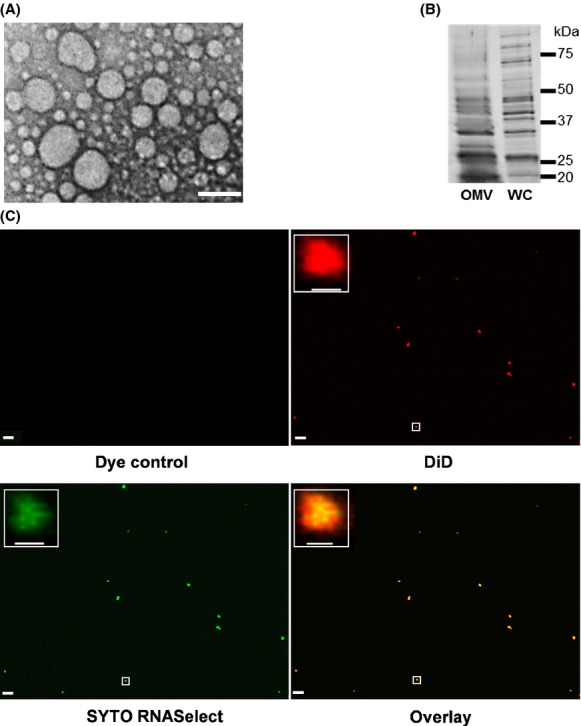
Characterization of outer membrane vesicular fractions isolated from *Escherichia coli* MG1655. (A) Visualization of isolated outer membrane vesicles (OMVs) using transmission electron microscopy (TEM). The size of the scale bar is equivalent to 50 nm. (B) SDS-PAGE gel image of protein samples from the OMV fraction and whole cells. Molecular weight markers are provided at the right side of the figure. (C) Confocal microscopy analysis of OMVs, stained with lipid tracer dye, DiD (red), and RNA-specific dye, SYTO RNASelect (green). The area highlighted within the white rectangular box is magnified in the inset. The scale bar is equivalent to 5 *μ*m in the main images and equivalent to 500 nm in the magnified images.

We hypothesized that bacteria release intracellular RNAs into their extracellular milieu, either through the shedding of OMVs or by using secretory pathways, in ways analogous to eukaryotic cells. In order to test our hypothesis, we first confirmed the presence of OMVs in the standard growth medium (LB) of *E. coli* K-12 substrain MG1655 using standard isolation procedures and transmission electron microscopy (Fig.1A; Materials and Methods section). The enriched LB medium was chosen over a synthetic medium to obtain high growth yields of *E. coli* for subsequently being able to extract sufficient amounts of RNA. In addition, we analyzed the protein banding profiles of the OMV fractions and found these to be distinct from whole-cell protein extracts (Fig.1B) (Wai et al. 2003). These commonly used analyses confirmed the presence of OMVs.

Next, we examined whether the OMV fraction from *E. coli* contains RNA, by applying a fluorescent labeling method which was adapted for staining of the OMV fraction (Nicola et al. 2009). Using this method, OMV-associated lipids were labeled with the lipid tracer dye DID and RNAs with SYTO RNAselect dye, respectively (Fig.1C). In most cases, RNA is found to be associated with OMV-associated lipids but the observed patterns also suggest the presence of nucleoprotein complexes and/or free extracellular RNA (Fig.1C). Each individual color dot in the images likely represents the aggregation of several OMVs, as the typical sizes of OMVs are below the limit of resolution of confocal microscopy. As a negative control, a mixture of both dyes was also analyzed using the same procedure and no particles were identified (Fig.1C). Through application of protocols similar to those used for the characterization of eukaryotic extracellular RNA, we were subsequently able to isolate both free and OMV-associated RNA from cultures of *E. coli* K-12 substrain MG1655.

### Characterization of extracellular RNAs

**Figure 2:**
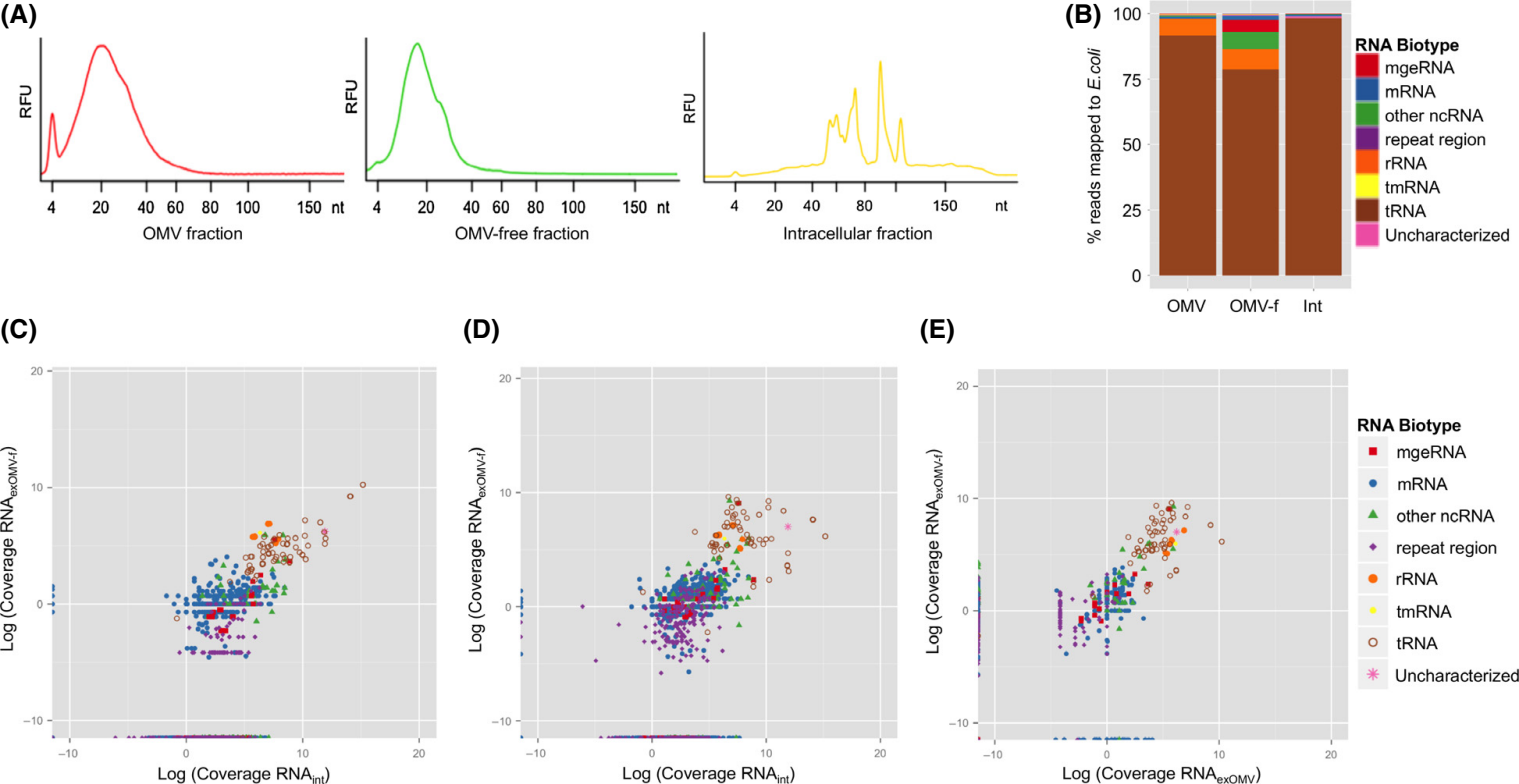
Characterization of the extracellular RNA complement of *Escherichia coli*. (A) Electropherograms of obtained RNA fractions below the length of 200 nucleotides. (B) Annotated profile of RNA_exOMV_ (OMV), RNA_ex__OMV__-f_ (OMV-f), and RNA_int_ (Int). (C), (D) and (E) represent plots of the inferred abundance levels of different RNA biotypes in different RNA fractions, that is, RNA_int_, RNA_ex__OMV_, and RNA_ex__OMV__-f_, against each other. Mobile genetic element- mge.

Following OMV isolation, RNA was extracted from the extracellular OMV fraction (RNA_exOMV_), extracellular OMV-free fraction (RNA_exOMV-f_), and from whole cells (subsequently referred to as the intracellular RNA fraction; RNA_int_). To minimize potential confounding effects of the presence of RNA in the RNA_exOMV-f_ fraction due to the lysis of cells and/or OMVs, RNA was always isolated from the respective fractions from early stationary growth phase. The presence of distinct RNAs in the RNA_exOMV-f_ fraction compared to RNA_int_ (please also see below) suggests the involvement of unknown mechanisms in the secretion of bacterial intracellular RNA into the extracellular milieu in addition to their release through OMVs. Each RNA fraction was subdivided into a <200 nt long RNA fraction (henceforth referred to as the “small RNA fraction”) and >200 nt long RNA fraction (“large RNA fraction”) and analyzed using an Agilent Bioanalyzer (Materials and Methods section). The majority of the RNA obtained from the RNA_exOMV_ and RNA_exOMV-f_ has a length less than 60 nt with an enrichment in the size range between 15 and 40 nucleotides (Fig.2A, Fig. S1).

Given the distinct enrichment in small RNA in the extracellular RNA complements, we further characterized these by deep RNAseq (Materials and Methods section). In order to compare the extracellular RNA profiles with the intracellular RNA profiles, the extracted <200 nt long intracellular RNA fraction was also sequenced. To identify and remove possible contaminant RNAs present in the LB growth medium (Pavankumar et al. 2012) from the subsequent analyses, RNA was extracted from freshly prepared cell-free LB medium and cell-free LB medium incubated overnight (without bacterial inoculation), and sequenced in the same way (Materials and Methods section). Analysis of the medium-derived reads highlight that the vast majority of these map to the yeast genome which can be explained by the presence of yeast extract in the LB medium. Prior to a detailed analysis of the sequence data mapping to the *E. coli* MG1655 genome, RNAseq read sequences in the extracellular and intracellular libraries, which were identical to those in the RNAseq libraries of cell-free LB media samples, were removed from the libraries to strictly limit potential contamination. This resulted in the removal of about 3% and 90% of the reads from intracellular and extracellular RNA read libraries, respectively (Table S1). Reads which mapped to both the media RNA libraries and the *E. coli* genome were termed “cross-mapping reads.” The majority of the cross-mapping reads from RNA_int_ map to tRNA and protein coding genes in *E. coli*, whereas protein coding genes dominate the cross-mapping reads in RNA_exOMV_, and tRNAs in RNA_exOMV-f_, respectively (Fig. S2 and Table S2).

The reads not subjected to cross-mapping to RNAs in the growth medium were mapped to the *E. coli* MG1655 genome and annotated based on known RNA biotypes using the RefSeq annotation (http://www.ncbi.nlm.nih.gov/refseq/). Reads mapped to *E. coli* but not to known RNA biotypes, were designated as uncharacterized (Fig.2B, Table S3). The distributions of different *E. coli* RNA biotypes in RNA_exOMV_, RNA_exOMV-f_ and RNA_int_ are distinct. However, in all fractions, the majority of the reads mapped to tRNA genes. In order to confirm the *E. coli* origin of these reads, all tRNA reads mapped to *E. coli* were again blasted against the yeast transcriptome obtained from the NCBI database and none of the identified *E. coli* tRNA reads aligned significantly to the yeast transcripts. In RNA_exOMV_ and RNA_exOMV-f_, the second major RNA biotype is represented by fragments of rRNA, which comprise 6.4% and 7.7% of the total sequenced reads mapped to the *E. coli* genome, whereas only 0.14% are found in RNA_int_. Other noncoding RNAs (ncRNAs) and RNAs encoded by mobile genetic elements (including cryptic prophage) comprise 0.54% and 0.19% of the total mapped sequence reads in RNA_exOMV_, compared to 6.5% and 4.5% in RNA_exOMV-f_, and 0.19% and 0.05% in RNA_int_, respectively. Furthermore, the mapping of reads corresponding to individual RNA biotypes reinforces the intracellular origin of extracellular RNAs while discrete differences in coverage may indicate discrete processing of these RNAs according to their localization (Fig.2C and D). There are numerous biotypes that are unique to RNA_int_, whereas very few unique biotypes, represented mainly by mRNA fragments, are present in the RNA_ex_ (Table S4). On the one hand, the observation of numerous biotypes unique to RNA_int_ underpins the notion of selective export of RNA biotypes, analogous to observations in eukaryotes (Hoen et al. 2012). On the other hand, the detection of unique biotypes in RNA_ex_ may be due to the intracellular forms of these fragments being of longer length than 60 nt and, thus, not represented in the intracellular smallRNAseq data. Moreover, the fractions of RNA_exOMV_ and RNA_exOMV-f_ are distinct in their RNA profile as both fractions hold unique biotypes (Fig.2E, Table S5, S6) leading to distinct distribution patterns of RNA biotypes (Fig.2B). More specifically, a significant number of unique reads in RNA_exOMV-f_ map to mobile genetic elements, ncRNA genes, and protein coding regions, whereas they mainly map to protein coding regions for RNA_exOMV_.

### Analysis of dominant extracellular RNA biotypes

**Figure 3:**
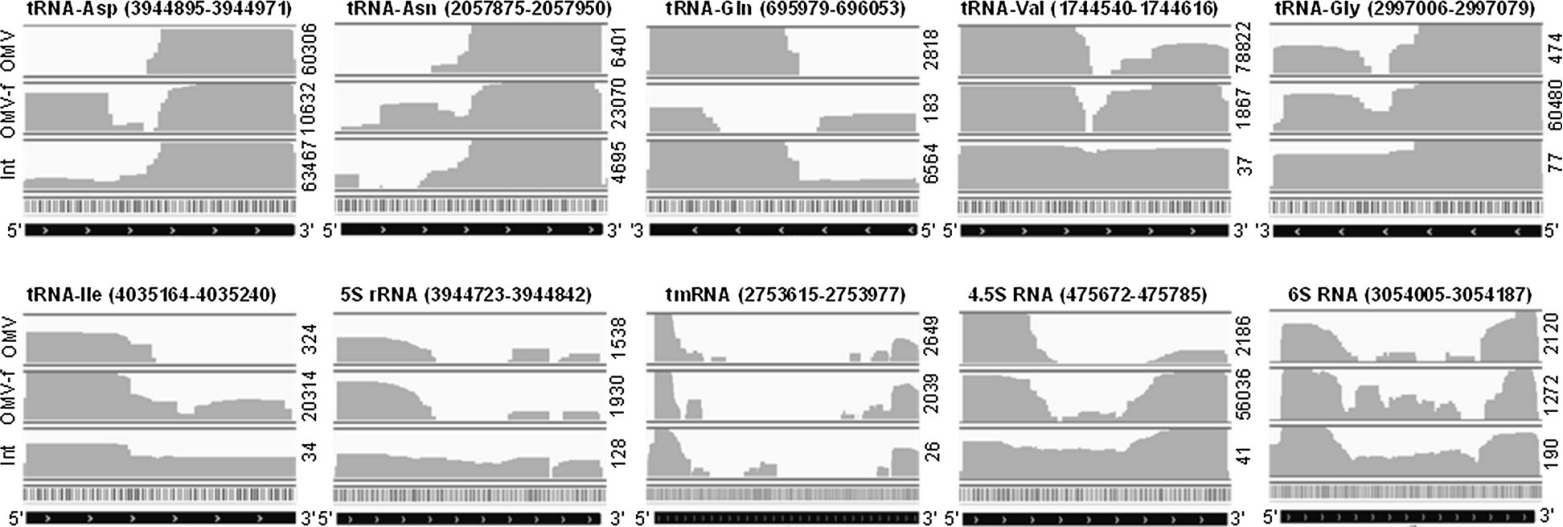
Coverage of *Escherichia coli* genomic regions by the intra- and extracellular RNA complement. Images were exported from the Integrative Genome Viewer software (http://www.broadinstitute.org/igv/). Histograms represent the abundances of RNA reads mapped over the length of the respective genomic regions. Gene names are indicated below the respective mappings. Int, OMV-f and OMV represent the histograms of RNA_int_, RNA_ex__OMV__-f_ and RNA_ex__OMV_, respectively. For each individual genomic region, CPM (counts per million) values were obtained for each type of sample and these values are reported on the right hand side of the images.

The majority of the sequenced reads represent tRNA fragments (tRFs). These map mostly to the 5′ and 3′ ends of mature tRNAs (Fig.3). Despite their high coverage in both RNA_ex_ and RNA_int_, the coverages of reads mapping to tRFs vary among the three different fractions (Table1). High abundance of full-length tRNAs is only apparent in RNA_int_ (Figs.2A, 3). Based on read coverage, clear differences in the levels of tRFs between RNA_int_, RNA_exOMV_, and RNA_exOMV-f_ are apparent. More specifically, the tRFs, which are highly abundant in RNA_int_, are partially reflected in RNA_exOMV_, whereas RNA_exOMV-f_ appears significantly different, as determined by comparing the distinct abundance ranks in each fraction. For example, in RNA_exOMV_ and RNA_exOMV-f,_ tRFs from tRNA-Glu are ranked 1st and 28th, tRFs from tRNA-Asp are ranked 2nd and 8th, tRFs from tRNA-Gln are ranked 5th and 109th, tRFs from tRNA-Asn are ranked 3rd and 3rd, tRFs from tRNA-Ser are ranked 24th and 57th, and tRFs from tRNA-Val are ranked 20th and 2nd, respectively. Comparison among the tRFs obtained from RNA_exOMV_, RNA_exOMV-f_, and RNA_int_ suggests that the majority of the 5′ and 3′ tRFs are exported into the extracellular milieu (Fig.3) as previously observed in eukaryotes (Hoen et al. 2012). The abundances of tRFs in the RNA_ex_ inferred from read counts (Table1) and their read mapping patterns (Fig.3) indicate that the export of tRFs into the extracellular milieu may involve specific processing as well as selection. Most tRFs are in the size range between 15 and 38 nt, and tRFs in that range have been reported to be functional in bacteria (Haiser et al. 2008) and eukaryotes (Thompson et al. 2008; Lee et al. 2009). In a recent study on human prostate cancer cells, tRFs of length between 17 and 26 nt were identified to constitute the second largest pool of total intracellular RNA, and more specifically tRFs originating from tRNA-Asp, tRNA-Leu, tRNA-Ala, tRNA-Gly, and tRNA-Glu constitute a significant portion of total tRFs (Lee et al. 2009). During stress, especially oxidative stress, the levels of tRFs have been shown to increase in yeast (Thompson et al. 2008). Here, increased amounts of tRFs are typically associated with specific cleavage products of tRNAs and such findings have also been reported for plants and humans (Thompson et al. 2008). In *Streptomyces coelicolor*, the abundances of distinct tRFs follow a growth pattern, and abundant tRFs originate from tRNA-Asn, tRNA-Gln, tRNA-Gly, tRNA-Val, and others (Haiser et al. 2008). Analogous to these previous findings in human, yeast, and bacterial cells, our results suggest that specific tRFs may play a range of important functional roles including possible intercellular interaction.

**Table 1:** List of abundant tRNA fragements (tRFs)

Fractions	Gene product	Genomic region	No. of reads^2^	Most abundant read (raw counts^3^)
OMV RNA	tRNA-Glu	4	111,349	GGCGGTAACAGGGGT (110,432)
	tRNA-Asp	3	30,817	GCAGGGGGTCGCGGGTTCGAGTCCCGTCCGTTCCGCCA (23,916)
	tRNA-Asn	4	4368	TCCGTATGTCACTGGTTCGAGTCCAGTCAGAGGAGCCA (2118)
	tRNA-Thr	4	1879	AGTTCGATTCCGGTAGTCGGCACCA (128)
	tRNA-Gln	4	1517	ACCGGCATTCCCTGGTTCGAATCCAGGTACCCCAGCCA (465)
OMV-free RNA	tRNA-Thr	4	28,299	GGGTGAGGTCGGCAGT (3732)
	tRNA-Val	7	19,834	TCGAGTCCACTCGGACGCAC (4069)
	tRNA-Asn	4	17,691	GTATGTCACTGGTTCGAGTCCAGTCAGAGGAGC (2772)
	tRNA-Gly	6	13,466	GCGGGCGTAGTTCAATGGTAGAACGAGAGCTTCCC (3571)
	tRNA-Ala	5	11,776	GGGGCTATAGCTCAGCTGGGAGAGCGCCTGCTT (2074)
	tRNA-Ile	3	11,683	AGGCTTGTAGCTCAGGTGGTTAGAGC (4330)
	tRNA-Leu	8	7273	GCCGAAGTGGCGAAATCGGTAGACGCAGTTGATT (1202)
	tRNA-Asp	3	6168	GGAGCGGTAGTTCAGTCGGTTAGA (899)
	tRNA-His	1	5920	GGTGGCTATAGCTCAGTTGGTAGAGCCC (1084)
Intracellular RNA^1^	tRNA-Glu	4	15,469,939	GGCGGTAACAGGGGT (15,382,097)
	tRNA-Asp	3	3,908,936	GCAGGGGGTCGCGGGTTCGAGTCCCGTCCGTTCCGCCA (3,049,567)
	tRNA-Gln	4	591,759	TTCCGGCATTCCGAGGT (244,848)
	tRNA-Asn	4	399,219	TCCGTATGTCACTGGTTCGAGTCCAGTCAGAGGAGCCA (231,638)
	tRNA-Ser	1	148,018	ACCGGCGACCCGAAAGGGTTC (138,124)
	tRNA-Val	7	105,509	GGAGGGGGTCGGCGGTTCGATCCCGTCATCACCCACCACT (56,389)

1 The intracellular RNA fraction contains full-length tRNAs along with tRFs.

2 Total number of reads mapped to the genomic region.

3 Coverage of individual reads.

Apart from tRFs, the second major constituents of extracellular RNAs are fragments of rRNA (Fig.2B). These rRNA fragments map to different discrete regions of the 23S rRNA, 16S rRNA, and 5S rRNA genes in both RNA_ex_ and RNA_int_. These rRNA fragments may represent rRNA degradation products. However, we identified a 23 nt long fragment of the 5′ end of the 5S rRNA [TGCCTGGCGGCCGTAGCGCGGTG (+st)] and two 17 nt long fragments of the 3′ end of the 5S rRNA [GTGGGGTCTCCCCATGC (+st) and AGGGAACTGCCAGGCAT (+st)], which seem to be exclusively exported as suggested by the mapping patterns of read sequences (Fig.3). rRNA fragments of similar size are known to be constituents of small RNA fractions not only in bacteria (Haiser et al. 2008; Kang et al. 2013) but also in eukaryotes (Thompson et al. 2008; Hoen et al. 2012). Significant amounts of various fragments of bacterial rRNA have also been observed in human plasma (Wang et al. 2012). A recent study has demonstrated that a conserved 23S rRNA motif “CGGAAAGACC” is recognized by the TLR13 receptor in mice, and modifications in the motif allows bacteria, for example, erythromycin-resistant *Staphylococcus aureus*, to bypass host immune recognition (Oldenburg et al. 2012). Although TLR13 is not found in humans, it is possible that human pattern recognition receptors (PRRs) exist which may ensure similar functions in humans. The “CGGAAAGACC” motif was identified in RNA_ex_ and RNA_int_, albeit with low coverage values of 19 in RNA_exOMV_, 24 in RNA_exOMV-f_ and 43 in RNA_int_, respectively. From our own data, it is intriguing that the tRF originating from tRNA-Glu has the sequence “CGGTAACAGG” which differs from the sequence of the motif described for fragments of the 23S rRNA by the following highlighted (bold, underlined) nucleotides: “CGG**A**AA**G**A**CC.**” For the tRF, thymine is substituted by adenine, whereas cytosine is substituted by guanine and vice versa. This sequence is present on a 15 nt long tRF, which was found in RNA_exOMV_ (coverage of 111,349), RNA_exOMV-f_ (coverage of 270), and RNA_int_ (coverage of 15,469,939) (Table1). Further investigation on the tRF originating from tRNA-Glu may identify new PRRs, which may be involved in exogenous RNA recognition by hosts including human.

Other small ncRNAs such as parts of tmRNA, 4.5S RNA (SRP-RNA), 6S RNA, and others are also selectively enriched in the RNA_ex_ (Table2 and Fig.3). Further analysis confirmed that 29 and 23 nt long small ncRNAs [GGGGGCTCTGTTGGTTCTCCCGCAACGCT (+st) and TGTAGCTGGCAGGGCCCCCACCC (+st)] result from the 5′ and 3′ ends of the 4.5S RNA, respectively. These are selectively exported in OMVs but are also found in RNA_exOMV-f_. In bacteria, 4.5S RNA is bound to the Ffh protein and constitutes the signal recognition particle (SRP). One of the functions of SRP involves the targeting of membrane and secretory proteins to the plasma membrane of bacteria following the recognition of a signal peptide sequence on the nascent polypeptide (Wassarman et al. 1999; Batey 2000). The SRP is also conserved in mammals (Batey 2000). In particular, immune cells are known to release fragments of SRP-RNA into the extracellular space (Hoen et al. 2012). Although the functions and the sequence information of exported fragments of SRP-RNA have not yet been described in detail, this does suggest a commonality in exported RNA in both bacteria and eukaryotes. Likewise, 22 and 16 nt long small ncRNAs [TTTCTCTGAGATGTTCGCAAGC (+st) and CATCTCGGAGATTCCC (+st)] from the 5′ and 3′ ends of the 6S RNA, respectively, and a 25 nt long small ncRNA [GGGGCTGATTCTGGATTCGACGGGA (+st)] from the 5′ end of tmRNA are also released by *E. coli* into its extracellular milieu. 6S RNA and tmRNA are not well conserved in eukaryotes (Wassarman et al. 1999) but play an important role in transcription and translation in bacteria (Wassarman and Storz 2000; Janssen and Hayes 2013). The presence of fragments of 6S RNA and tmRNA in the extracellular milieu, similar to fragments of SRP-RNAs, suggests selective mechanisms for their export.

**Table 2:** List of abundant small non-coding RNAs

Fractions	Gene product	No. of reads^1^	Most abundant read (raw counts^2^)	Rank^3^
OMV RNA	tmRNA 105a RNA (*ssrA*)	452	GGGGCTGATTCTGGATTCGACGGG (108)	124
	4.5S sRNA component of Signal Recognition Particle (*ffs*)	373	GGGGGCTCTGTTGGTTCTCCCGCAACGCT (82)	95
	6S RNA inhibits RNA polymerase promoter binding (*ssrS*)	361	CATCTCGGAGATTCCC (52)	47
OMV-free RNA	4.5S sRNA component of Signal Recognition Particle (*ffs*)	10,743	GGGGGCTCTGTTGGTTCTCCCGCAACGCT (1383)	95
	Novel sRNA (*ryeA*)	405	CAAGAGCCATTTCCCTGGACCG (103)	40
	tmRNA 105a RNA (*ssrA*)	391	GGGGCTGATTCTGGATTCGACGGGA (87)	124
	Novel sRNA (*ryfD*)	369	AAGACGATCCGGTACGC (108)	54
	6S RNA inhibits RNA polymerase promoter binding (*ssrS*)	244	TTTCTCTGAGATGTTCGCAAGC (19)	47
	sRNA antisense regulator of gadAB transcriptional activator GadX mRNA (*gadY*)	230	TCTGGAGACGGCAGACT (104)	100
	sRNA antisense regulator represses oppA, dppA, gltI and livJ expression (*ryeB*)	124	AGAGAGCCGTGCGCTAAAAGT (61)	72
Intracellular RNA	Novel sRNA(*ryeA*)	4907	AGAGAGCCGTGCGCTAAAAGT (477)	1
	sRNA antisense regulator blocking mokB (*sokB*)	4730	ATTCGTTGGCCTCGGTTGA (3580)	2
	sRNA antisense regulator affects LdrB translation (*rdlB*)	4413	GTCAGGTTTTACCTCTCAACGTGCGGGGGTTTTCTC (2180)	3
	6S RNA inhibits RNA polymerase promoter binding (*ssrS*)	4050	TTTCTCTGAGATGTTCGCAAGCGGGCCAGTCC (287)	4
	Novel sRNA (*ryfD*)	2575	GGAGGAGTTATGCGTCTGGATCGTCTTACT (536)	5
	sRNA antisense regulator of OM chitoporin (*chiX*)	2396	TCTTTGACGGGCCAATAGCGATATTGGCCATTTTTTT (413)	6

1 Total number of reads mapped to the genomic region.

2 Coverage of individual reads.

3 Ranks provided for small non-coding RNAs derived from RNA_exMOV_ and RNA_exMOV-f_ are ranked according to their abundance in RNA_int_.

In our analysis, we have found that a number of sequencing reads in both the RNA_ex_ and RNA_int_ mapped to mobile genetic elements of the *E. coli* MG1655 genome, primarily to the cryptic prophages CP4-6, DLP12, and e14, with the majority of those exported reads in the RNA_exOMV-f_ fraction mapping to cryptic prophage CP4-6. Cryptic prophages provide selective advantages to their hosts in different conditions (Wang et al. 2010) and they may confer protection against external bacteriophages (Casjens 2003). The two most abundant 35 nt and 28 nt long small ncRNAs [GAAAAAACTGGTACCGCCAAGACTACACACAGCAT (+st) and AATGACGCCCGCGAGTGTGCAGCTCCGG (+st)] are part of e14 and were found in high relative abundance in both RNA_int_ and RNA_ex_. Similar to the CRISPR/Cas system where transcripts of virus-originating spacer DNA sequence defend bacteria from invading viruses, small ncRNAs of cryptic prophage origin may play an analogous role in defense and/or virulence.

**Figure 4:**
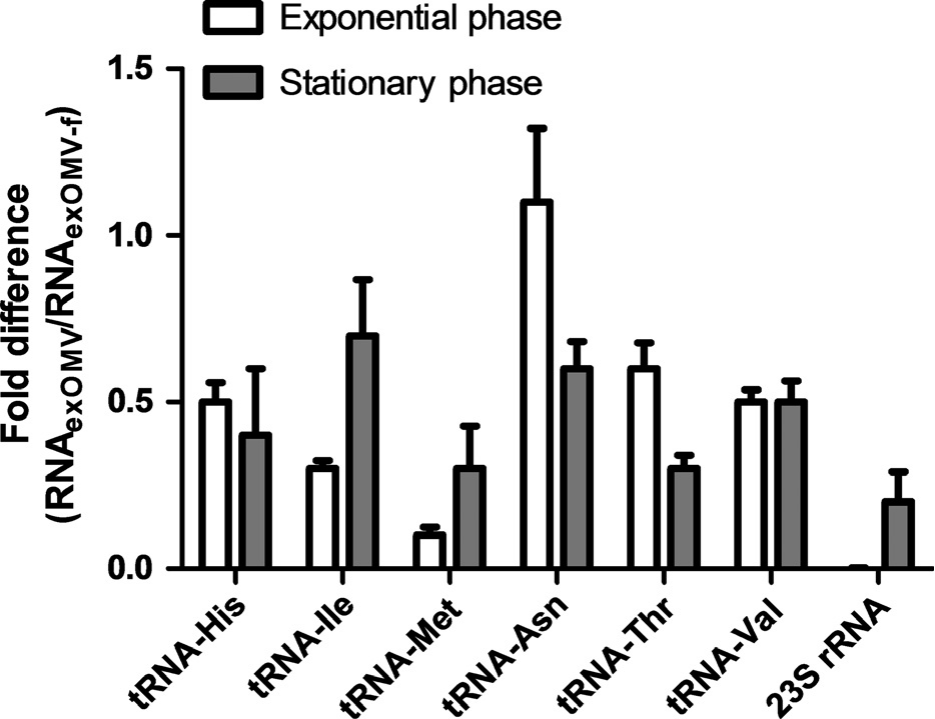
RT-qPCR validation of identified small noncoding RNAs. Extracellular RNAs quantified from exponentially growing *Escherichia coli* cultures and overnight grown cultures using RT-qPCR. Error bars represent standard error of the mean (*n = 3*).

**Figure 5:**
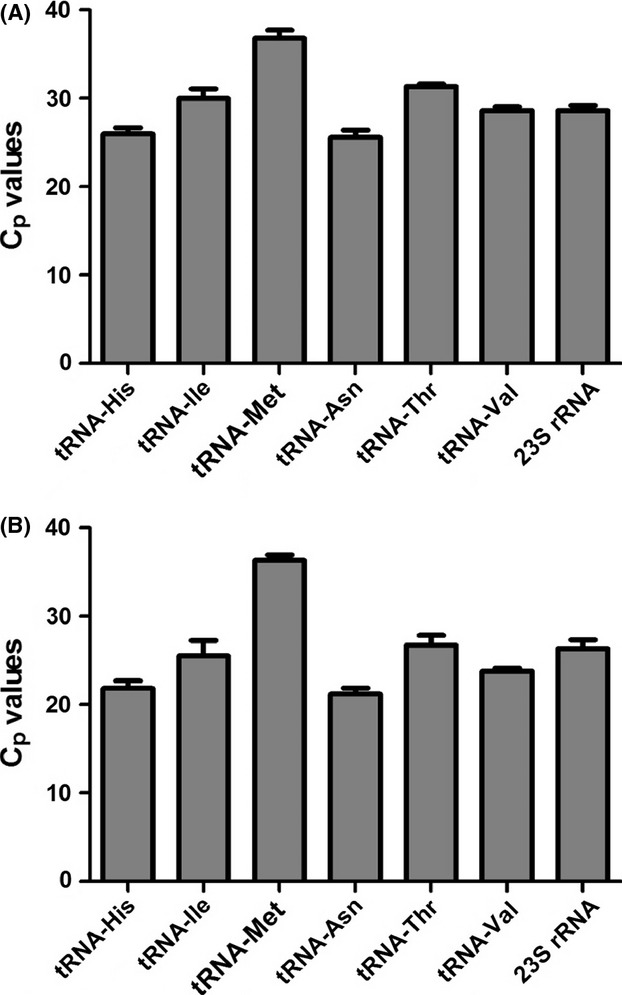
RT-qPCR validation of small noncoding RNAs in the (A) OMV and (B) OMV-f fractions obtained from bacterial cultures grown in M9 minimal medium. Error bars represent standard error of the mean (*n* = 3).

To identify the potential changes in RNA_ex_ at different growth phases and to validate the results obtained from RNAseq, equal amount of RNAs from RNA_exOMV_ and RNA_exOMV-f_ fractions, extracted from exponentially growing bacteria and overnight grown cultures were analyzed by RT-qPCR (Fig.4). For all the samples analyzed, the amounts of analyzed small ncRNAs are higher in RNA_exOMV_ in comparison with the RNA_exOMV-f_, and the observed differences are consistent in both exponential and stationary growth phases (Fig.4). By and large, the amount of small RNA fragments released is also consistent in both bacterial growth phases with the exception of RNA fragments originating from tRNA-Asn and 23S rRNA (Fig.4). For additional validation of these findings, we also extracted small RNA from the OMV and OMV-f fractions obtained from bacterial cultures grown in synthetic M9 minimal medium (RNA-free) and analyzed these in an analogous way. The results from these experiments highlight the presence of the previously identified small RNAs in the M9 OMV and OMV-f fractions (Fig.5), which lends additional independent support to the previous RT-qPCR results from samples obtained from cultures grown in LB medium. Taken together, these RT-qPCR results validate the previous observations based on deep-sequencing analyses and put forward a notion of continuous release of small RNA into the extracellular milieu.

**Figure 6:**
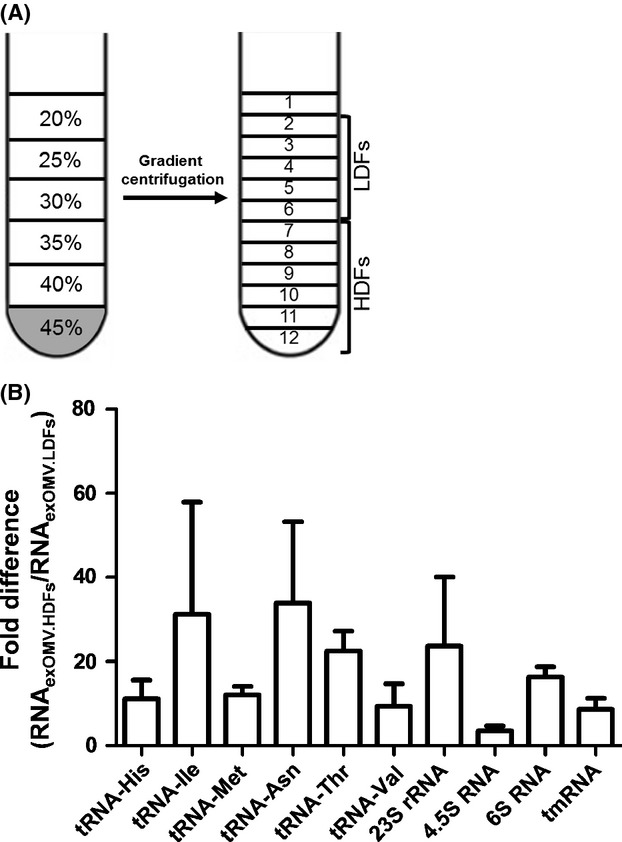
Analysis of small noncoding RNAs in subfractionated OMV samples. (A) Representation of the differential centrifugation scheme used to subfractionate the obtained- *Escherichia coli* OMV fraction into low- and high-density fractions (LDF and HDF), respectively. (B) Comparison of the relative abundances of identified small noncoding RNAs between HDFs (less pure OMV fractions) and LDFs (pure OMV fractions). RNA quantities were determined by RT-qPCR. LDFs: low-density fractions, HDFs: high-density fractions. Error bars represent the standard error of the mean (*n = 3*).

The secretion of vesicles is a common, conserved process which exists in both eukaryotes and prokaryotes (Mashburn-Warren et al. 2008; Chen et al. 2012). Vesicles are known to contain signaling molecules (Mashburn-Warren et al. 2008; Hannafon and Ding 2013) and are thought to be important mediators of intercellular information exchange via transfer of small RNA in eukaryotes (Valadi et al. 2007). To further characterize the RNA of the *E. coli* OMV fraction, we subfractionated the OMV fraction into low- and HDFs (Fig.6A). The LDFs contain “pure OMVs” (Horstman et al. 2000), whereas the HDFs may contain nucleic acid–protein complexes and RNA associated with the OMV surface dislodged during the fractionation process, in addition to OMVs (Fig. S3). RNA from these fractions was further analyzed by a combination of RT-qPCR and high-throughput sequencing. The amounts of previously identified extracellular RNAs comprising tRFs, fragments of rRNA, and small ncRNAs are higher in the HDFs compared to the LDFs, although the amount of individual small ncRNAs varies significantly between samples (Fig.6B). Even though the amount of small RNAs is higher in the HDFs, all RT-qPCR analyzed small RNAs are present in the LDFs (pure OMV fraction). The RNA obtained from the pure OMV fraction was further analyzed by deep sequencing. Similar to the previous observations of RNA_exOMV_, tRFs, and fragments of rRNA along with other small RNAs from coding regions dominate this OMV fraction (Table S7). Although, according to these results, OMVs contain RNA, our results also indicate that RNA may be associated with the OMV surface as previously described for DNA in *Moraxella catarrhalis*, where OMV-associated DNA is recognized by toll-like receptor 9 (TLR9) inside immune cells and thereby stimulating an immune response (Vidakovics et al. 2010). Similar to the situation described for DNA, OMV surface-associated RNA may be protected from degradation and may be involved in remote immune system stimulation through OMV-mediated transport. However, the determination of the precise localization of OMV-associated RNAs will require further in-depth studies.

### Identification of previously uncharacterized sRNAs

**Figure 7:**
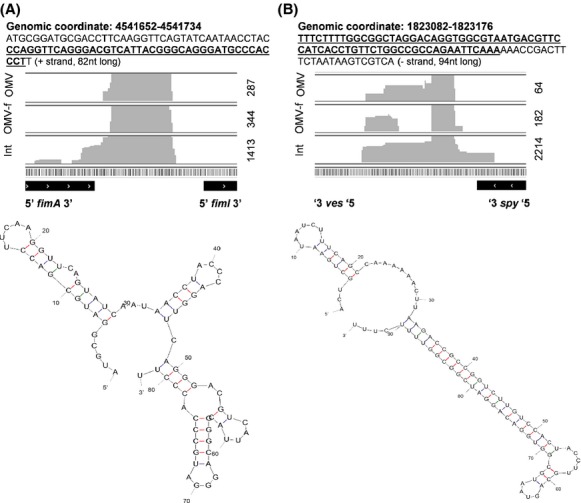
Identification of uncharacterized noncoding RNAs by mapping RNAseq data onto the *Escherichia coli* genome. (A) fimA-fimL and (B) ves-spy. In (A) and (B) the genomic coordinates represent the region where sequence reads were mapped. The nucleotide sequence is provided below the genomic coordinate. Underlined areas within the nucleotide sequence represent their overrepresentation in the RNAseq libraries. Mappings are provided for fimA-fimL and ves-spy. Flanking regions are named with genes. Int, OMV-f, and OMV represent the histogram of RNA_int_, RNA_ex__OMV__-f_, and RNA_ex__OMV_, respectively. For each individual genomic region, CPM (counts per million) values were obtained for each type of sample and these values are reported on the right-hand side of the images. Noncoding RNA sequences were structured in Mfold web server (http://mfold.rna.albany.edu/?q=mfold/RNA-Folding-Form)RNA-Folding-Form).

By careful inspection of read mapping profiles of short RNA, we identified genomic regions with high read coverages. These regions include two so far uncharacterized genomic regions in the *E. coli* genome (Fig.7). Both of these intergenic regions, *fimA*-*fimL* and *ves*-*spy*, have previously been found to exhibit high levels of expression (Kawano et al. 2005). In particular, a 35 nt long transcript originating from the *fimA*-*fimL* intergenic region was previously found to be abundant in *E. coli* in stationary growth phase (Kawano et al. 2005). Interestingly, we have observed transcripts of lengths between 31 and 37 nt from the same genomic region, which are specifically released into the extracellular milieu (Fig.7A). In addition, a previously uncharacterized 82 nt long transcript from the same region was identified in RNA_int_ close to what has previously been reported in the ECSBrowser (http://rna.iab.keio.ac.jp/, (Shinhara et al. 2011)).

Similarly, high levels of expression were observed for the intergenic region between *ves*-*spy*, where the majority of the reads correspond to a ∽66 nt long transcript which appears to originate from an ∽94 nt long genomic region (Fig.7B). In a previous study, high expression of a 45 nt long transcript and lower expression of 70 and 120 nt long transcripts from this intergenic region was observed at early stationary growth phase of *E. coli* (Kawano et al. 2005). In ECSBrowser, the transcript length is reported to be 86 nt between the genomic region of *ves* and *spy* genes. Interestingly, we identified a specific 17 nt long transcript [GACCGGCGGTCTTAAGT (+st)] encoded by this region which was present in both RNA_exOMV-f_ and RNA_exOMV_ (Fig.7B).

## Conclusions

We have carried out an in-depth characterization of the extracellular RNA complement of the enteric bacterium *E. coli*. We found that the secreted RNA complement is largely smaller than 60 nt, that it is enriched in nonprotein coding RNAs, that it is distinct from the intracellular RNA pool, and that the identities and abundances of the secreted RNA differ between the extracellular milieu and outer membrane vesicular fractions.

The observations reported here raise a number of important questions. These include the following: (1) How are non-OMV-associated bacterial RNAs transported across bacterial membranes and subsequently released into the extracellular space? (2) How are the RNAs selected for export? (3) Is there a difference in the extracellular RNA profile between pathogenic and non-pathogenic bacteria? Apart from seeking answers to these questions, extracellular bacterial RNAs should be investigated for their potential protein binding partners, host target cells and their potential as biomarkers. Understanding the role of bacteria-derived exogenous RNA in host–microbe interactions, in pathogenesis as well as in mutualism, will elucidate new mechanisms and perhaps allow the identification of new drug targets and/or the development of RNA-based vaccines. Further investigations in the field of extracellular bacterial RNAs are clearly needed to shed light on their potential role as mediators of microbe–microbe and host–microbe intercellular communication.

## Supporting Information

**Figure S1.**Electropherograms of *Escherichia coli* RNA fractions above the length of 200 nucleotides. (A) OMV fraction, (B) OMV-free fraction, (C) intracellular RNA fraction. **Figure S2.** Distribution of cross-mapping reads. **Figure S3.** Comparative analysis of protein fractions extracted from the OMV high density fractions (HDFs), the OMV low density fractions (LDFs) and whole cells (WC). Molecular markers are showed on the right side of the gel image.

**Table S1.** Analysis of sequence reads.

**Table S2.** Genomic features and corresponding read counts of cross-mapping reads.

**Table S3.** Genomic features and corresponding read counts.

**Table S4.** Unique biotypes represented by RNAex.

**Table S5.** Unique biotypes represented by RNA_exOMV_ in comparison with RNA_exOMV-f_.

**Table S6.** Unique biotypes represented by RNA_exOMV-f_ in comparison with RNA_exOMV_.

**Table S7.** Genomic features and corresponding read counts in low density fractions (LDFs).
