# Supplementary material for: The extracellular RNA complement of Escherichia coli
Source: Microbiologyopen. 2015 Jan 21;4(2):252–66. doi: 10.1002/mbo3.235 (PMC4398507; doi:10.1002/mbo3.235)
Supplement: Supplementary file 1 — Figure S1.Electropherograms of Escherichia coli RNA fractions above the length of 200 nucleotides. (A) OMV fraction, (B) OMV-free fraction, (C) intracellular RNA fraction. Figure S2. Distribution of cross-mapping reads. Figure S3. Comparative analysis of protein fractions extracted from the OMV high density fractions (HDFs), the OMV low density fractions (LDFs) and whole cells (WC). Molecular markers are showed on the right side of the gel image. [file mbo30004-0252-sd1.docx]

**The extracellular RNA complement of *Escherichia coli***

Anubrata Ghosal^1^, Bimal Babu Upadhyaya^1^, Joëlle V. Fritz^1^, Anna Heintz-Buschart^1^, Mahesh S. Desai^1^, Dilmurat Yusuf ^1^, David Huang^2^, Aidos Baumuratov^1^, Kai Wang^3^, David Galas^1, 2^, Paul Wilmes^1*^

**Supplementary Figure S1. Electropherograms of *E. coli* RNA fractions above the length of 200 nucleotides.** (A) OMV fraction, (B) OMV-free fraction, (C) intracellular RNA fraction.

**Supplementary Figure S2. Distribution of cross-mapping reads.**

**Supplementary Figure S3.** Comparative analysis of protein fractions extracted from the OMV high density fractions (HDFs), the OMV low density fractions (LDFs) and whole cells (WC). Molecular markers are showed on the right side of the gel image.
