# Supplementary material for: The extracellular RNA complement of Escherichia coli
Source: Microbiologyopen. 2015 Jan 21;4(2):252–66. doi: 10.1002/mbo3.235 (PMC4398507; doi:10.1002/mbo3.235)
Supplement: Supplementary file 2 — Table S1. Analysis of sequence reads. [file mbo30004-0252-sd2.pdf]

Supplementary table S1: Analysis of sequence reads

**Samlpe names**	**Percentage of reads mapped to media controls (reads)**	**Percentage of reads mapped to the media controls and the *E. coli* genome (no. of reads)**	**Percentage of reads mapped to the *E. coli* genome after removing the reads identical with the media controls (no. of reads)**
RNA_{int}	2.9 (650943)	0.4 (100789)	97.7 (21234997)
RNA_{exOMV}	93.7 (7346337)	14 (1108085)	34.7 (170220)
$RNA_{exOMV\text{-}f}$	92.1 (4063788)	11 (510851)	52.6 (182819)
