# Supplementary material for: The extracellular RNA complement of Escherichia coli
Source: Microbiologyopen. 2015 Jan 21;4(2):252–66. doi: 10.1002/mbo3.235 (PMC4398507; doi:10.1002/mbo3.235)
Supplement: Supplementary file 3 — Table S2. Genomic features and corresponding read counts of cross-mapping reads. [file mbo30004-0252-sd3.pdf]

Supplementary table S2: **Genomic features and corresponding read counts of cross-mapping reads**

RNA biotype	Product information	Genomic coordinates	RNA_{int} (Read Counts)	RNA_{exOMV} (Read Counts)	$RNA_{exOMV-f}$ (Read Counts)
mRNA	ID=cds3227	3436046-3436456	72116	1097852	7483
mRNA	ID=cds1659	1748369-1749037	271	3080	1190
rRNA	product=16S ribosomal RNA of rrnA operon	4033554-4035095	27.571401	350.056793	108.495382
rRNA	product=16S ribosomal RNA of rrnB operon	4164682-4166223	27.571401	350.056793	108.495382
rRNA	product=16S ribosomal RNA of rrnC operon	3939831-3941372	27.571401	350.056793	108.495382
rRNA	product=16S ribosomal RNA of rrnE operon	4206170-4207711	27.571401	350.056793	108.495382
rRNA	product=16S ribosomal RNA of rrnH operon	223771-225312	27.571401	350.056793	108.495382
rRNA	product=16S ribosomal RNA of rrnD operon	3425243-3426784	27.571401	349.856793	108.095382
rRNA	product=16S ribosomal RNA of rrnG operon	2727638-2729179	27.571401	349.856793	108.095382
tRNA	product=tRNA-Ala	2516063-2516138	813.912897	236.68225	6874.931624
tRNA	product=tRNA-Ala	2516178-2516253	813.912897	236.68225	6874.931624
tRNA	product=tRNA-Gly	2997006-2997079	1163	206	115550
mRNA	ID=cds3482	3704121-3705728	32	197	1
tRNA	product=tRNA-Arg	2815806-2815882	1361	188.75	23536.75
tRNA	product=tRNA-Arg	2816081-2816157	1361	188.75	23536.75
tRNA	product=tRNA-Arg	2816220-2816296	1361	188.75	23536.75
tRNA	product=tRNA-Arg	2816495-2816571	1361	188.75	23536.75
tRNA	product=tRNA-Ile	225381-225457	836.66583	149.333184	64387.60228
tRNA	product=tRNA-Ile	3425098-3425174	836.66583	149.333184	64387.60228
tRNA	product=tRNA-Ile	4035164-4035240	836.66583	149.333184	64387.60228
mRNA	ID=cds601	637050-637796	10	144	56
mRNA	ID=cds657	694324-695499	16	133	0
mRNA	ID=cds740	791539-793011	28	122	38
mRNA	ID=cds2937	3130476-3131234	16	122	128
tmRNA		2753615-2753977	381	118	32
mRNA	ID=cds587	621523-622773	7	104	492
mRNA	ID=cds4016	4316784-4317629	6	96	26
tRNA	product=tRNA-Cys	1989938-1990011	96	88	9171
Uncharacterized			2117.999907	86.204172	77.999952
mRNA	ID=cds837	896307-897152	1	73	0
mRNA	ID=cds719	770681-772249	9	72	52
mRNA	ID=cds233	260727-261980	7	58	13
tRNA	product=tRNA-Thr	4173411-4173486	112	47	603
tRNA	product=tRNA-Gly	4173696-4173770	1927	39	598
mRNA	ID=cds2102	2202618-2203706	41	36	24
mRNA	ID=cds3543	3768266-3769402	0	35	53
tRNA	product=tRNA-Asp	228928-229004	590.332743	32.666634	5686.994313
tRNA	product=tRNA-Asp	236931-237007	590.332743	32.666634	5686.994313
tRNA	product=tRNA-Asp	3944895-3944971	590.332743	32.666634	5686.994313
tRNA	product=tRNA-Ala	225500-225575	200.391243	31.545149	12438.69908
tRNA	product=tRNA-Ala	3424980-3425055	200.391243	31.545149	12438.69908
tRNA	product=tRNA-Ala	4035283-4035358	200.391243	31.545149	12438.69908
mRNA	ID=cds1169	1238102-1239172	6.999993	29.333304	0
mRNA	ID=cds3998	4299050-4301101	6.999993	29.333304	0
mRNA	ID=cds4065	4372652-4373680	6.999993	29.333304	0
mRNA	ID=cds1251	1327356-1328405	11	29	74
mRNA	ID=cds705	748945-751392	2	28	1
ncRNA		3054005-3054187	712	27	31
mgeRNA	gbkey=misc_feature	262182-296489	91	23.666666	4389
tRNA	product=tRNA-Thr	262095-262170	91.930492	22.642858	4387.49882
tRNA	pseudo=true	296430-296478	91	22.5	4387
rRNA	product=23S ribosomal RNA of rrnA operon	4035542-4038446	40.785675	20.624981	67.214226
rRNA	product=23S ribosomal RNA of rrnB operon	4166664-4169567	40.785675	20.624981	67.214226
rRNA	product=23S ribosomal RNA of rrnE operon	4208066-4210969	40.785675	20.624981	67.214226
rRNA	product=23S ribosomal RNA of rrnG operon	2724303-2727206	40.785675	20.624981	67.214226
rRNA	product=23S ribosomal RNA of rrnH operon	225759-228662	40.785675	20.624981	67.214226

rRNA	product=23S ribosomal RNA of rrnD operon	3421902-3424805	40.285674	20.458314	67.214226
rRNA	product=23S ribosomal RNA of rrnC operon	3941727-3944630	40.785675	20.291647	64.714221
mRNA	ID=cds3933	4220827-4221651	0	20	48
mRNA	ID=cds2489	2628980-2630557	6	19	30
tRNA	product=tRNA-Asn	2042573-2042648	1788	18	131.5
tRNA	product=tRNA-Asn	2056051-2056126	1788	18	131.5
tRNA	product=tRNA-Asn	2057875-2057950	1788	18	131.5
tRNA	product=tRNA-Asn	2060284-2060359	1788	18	131.5
mRNA	ID=cds4193	4503310-4504428	8	17	3
mRNA	ID=cds336	359216-360370	4	17	464
mRNA	ID=cds1593	1677581-1678963	2.5	16.5	9
mRNA	ID=cds3202	3405629-3407080	2.5	16.5	9
tRNA	product=tRNA-Pro	2284233-2284309	354	16	1261
Other ncRNA		475672-475785	41	15	428
mRNA	ID=cds470	504138-505790	0	15	0
mRNA	ID=cds3840	4101625-4102998	4	14	6
tRNA	product=tRNA-Met	2945409-2945485	38	13.75	1246.25
tRNA	product=tRNA-Met	2945519-2945595	38	13.75	1246.25
tRNA	product=tRNA-Met	2945629-2945705	38	13.75	1246.25
tRNA	product=tRNA-Met	3316235-3316311	38	13.75	1246.25
mRNA	ID=cds2753	2921024-2921806	0	13	0
mRNA	ID=cds3146	3342739-3344172	1	12.650212	43.919492
mRNA	ID=cds1875	1965476-1966525	1	11	14
mRNA	ID=cds3936	4227476-4228165	1	11	18
mRNA	ID=cds2174	2284412-2286936	0	11	1
mRNA	ID=cds2880	3066969-3067829	0	11	7
mRNA	ID=cds2965	3156949-3159168	2	10	73.308932
mRNA	ID=cds1171	1241389-1242303	0	9	0
mRNA	ID=cds3335	3524491-3526626	0	8	0
mRNA	ID=cds321	342108-343157	1.5	7.5	0.5
mRNA	ID=cds3586	3810754-3811974	1.5	7.5	0.5
mRNA	ID=cds3432	3638885-3640354	4	7	3
mRNA	ID=cds1175	1244383-1244823	2	7	1
mRNA	ID=cds1274	1350660-1351652	1	7	0
mRNA	ID=cds2939	3132153-3132845	1	7	34
mRNA	ID=cds164	190857-191708	0	7	0
mRNA	ID=cds305	321562-322989	0	7	0
mRNA	ID=cds1641	1726371-1727018	1	6	1
mRNA	ID=cds390	416366-417055	1	6	16
mRNA	ID=cds1035	1118691-1119809	0	6	1
mRNA	ID=cds119	138835-141225	0	6	0
mRNA	ID=cds2930	3122258-3123481	0	5.789515	0
mRNA	ID=cds478	513625-514083	3	5	2
mRNA	ID=cds1802	1892829-1894190	1	5	0
mRNA	ID=cds2325	2455037-2457181	1	5	13
mRNA	ID=cds2526	2671838-2672710	0	5	0
mRNA	ID=cds3288	3476824-3478629	0	5	4
tRNA	product=tRNA-Pro	3706639-3706715	65	4	61
mRNA	ID=cds1215	1290680-1291588	2	4	0
mRNA	ID=cds2162	2270386-2272200	1	4	0
mRNA	ID=cds1302	1379971-1380876	1	3	6
mRNA	ID=cds1628	1716090-1716419	1	3	0
mRNA	ID=cds2623	2776168-2780748	1	3	3
mRNA	ID=cds2317	2447250-2448071	0	3	0
mRNA	ID=cds2640	2798156-2798497	0	3	1
mRNA	ID=cds4032	4331970-4333613	0	3	0
mRNA	ID=cds607	643420-644226	0	3	7
mRNA	ID=cds1791	1881212-1882657	0	2.5	0

tRNA	product=tRNA-Ile	2783784-2783859	0	2.5	2
tRNA	product=tRNA-Ile	3213620-3213695	0	2.5	2
tRNA	product=tRNA-Ser	2816575-2816667	66	2	25
mRNA	ID=cds2923	3110115-3110963	2	2	0
mRNA	ID=cds1005	1092099-1093457	1	2	0
mRNA	ID=cds3260	3451951-3453420	1	2	2
mRNA	ID=cds623	658474-659439	1	2	0
tRNA	product=tRNA-Sec	3834245-3834339	1	2	144
mRNA	ID=cds1026	1110086-1112629	0	2	1
mRNA	ID=cds1304	1382141-1383538	0	2	0
mRNA	ID=cds1454	1536874-1540614	0	2	98
mRNA	ID=cds1574	1656093-1658519	0	2	0
mRNA	ID=cds2226	2353543-2354733	0	2	0
mRNA	ID=cds2590	2746796-2748082	0	2	0
mRNA	ID=cds2698	2859452-2860360	0	2	7
mRNA	ID=cds2957	3149272-3150006	0	2	1
mRNA	ID=cds3054	3251340-3252236	0	2	6
mRNA	ID=cds3092	3291422-3293458	0	2	1
mRNA	ID=cds326	346081-347667	0	2	0
mRNA	ID=cds3291	3481224-3482246	0	2	2
mRNA	ID=cds3313	3502957-3504042	0	2	0
mRNA	ID=cds339	362455-365529	0	2	2
mRNA	ID=cds3429	3635665-3637164	0	2	2
mRNA	ID=cds3503	3725430-3725771	0	2	0
mRNA	ID=cds3535	3760206-3764339	0	2	45
mRNA	ID=cds3826	4086130-4087878	0	2	0
mRNA	ID=cds4001	4302635-4304620	0	2	1
mRNA	ID=cds4205	4512376-4514700	0	2	0
mRNA	ID=cds507	545904-547571	0	2	0
mRNA	ID=cds637	671424-674006	0	2	1
mRNA	ID=cds770	822959-823720	0	2	0
mRNA	ID=cds902	974845-975549	0	2	12
mRNA	ID=cds993	1072086-1073234	0	2	1
mRNA	ID=cds2476	2614116-2615579	0	1.5	1
mRNA	ID=cds2571	2729622-2732195	0	1.5	3
mRNA	ID=cds410	436385-437359	0	1.5	0.5
Other ncRNA		3984455-3984626	0	1.5	0.5
tRNA	product=tRNA-Glu	2727391-2727466	17.25	1.25	14.25
tRNA	product=tRNA-Glu	3941458-3941533	17.25	1.25	14.25
tRNA	product=tRNA-Glu	4166395-4166470	17.25	1.25	14.25
tRNA	product=tRNA-Glu	4207797-4207872	17.25	1.25	14.25
tRNA	product=tRNA-Tyr	1286467-1286551	23.499999	1.166666	2.333333
tRNA	product=tRNA-Tyr	1286761-1286845	23.499999	1.166666	2.333333
mRNA	ID=cds213	244327-245094	2	1	0
mRNA	ID=cds1326	1404587-1405819	1	1	0
mRNA	ID=cds2386	2522067-2522900	1	1	0
mRNA	ID=cds2916	3102115-3102390	1	1	0
mRNA	ID=cds4080	4384070-4387393	1	1	0
mRNA	ID=cds1002	1085744-1087069	0	1	0
mRNA	ID=cds1147	1218824-1221471	0	1	1
mRNA	ID=cds125	145081-146310	0	1	0
mRNA	ID=cds1305	1383535-1384596	0	1	1
mRNA	ID=cds136	156299-156883	0	1	0.5
mRNA	ID=cds1455	1540696-1542084	0	1	0
mRNA	ID=cds150	175107-176528	0	1	36
mRNA	ID=cds1554	1644429-1644761	0	1	0
mRNA	ID=cds1605	1692284-1694095	0	1	0
mRNA	ID=cds1899	1987705-1988916	0	1	0

mRNA	ID=cds1903	1992727-1993383	0	1	1
mRNA	ID=cds197	229967-230881	0	1	0
mRNA	ID=cds2076	2170945-2172300	0	1	0
mRNA	ID=cds2114	2215422-2216579	0	1	0
mRNA	ID=cds2187	2296737-2297600	0	1	1
mRNA	ID=cds2200	2311510-2314182	0	1	1
mRNA	ID=cds2207	2322778-2324100	0	1	1
mRNA	ID=cds2519	2666028-2666918	0	1	0
mRNA	ID=cds2551	2702357-2703331	0	1	1
mRNA	ID=cds2794	2970691-2971884	0	1	1
mRNA	ID=cds2807	2987326-2987808	0	1	0
mRNA	ID=cds2846	3031679-3033196	0	1	0
mRNA	ID=cds2960	3152284-3153240	0	1	0
mRNA	ID=cds2961	3153377-3154540	0	1	0
mRNA	ID=cds2984	3176137-3177618	0	1	0
mRNA	ID=cds3030	3229687-3231705	0	1	0
mRNA	ID=cds311	328687-330720	0	1	0
mRNA	ID=cds3192	3396409-3396897	0	1	2
mRNA	ID=cds322	343400-344215	0	1	0
mRNA	ID=cds3264	3457839-3459035	0	1	0
mRNA	ID=cds329	350439-351890	0	1	0
mRNA	ID=cds3308	3497932-3499269	0	1	0
mRNA	ID=cds3343	3534834-3535310	0	1	0
mRNA	ID=cds3603	3830242-3832560	0	1	2
mRNA	ID=cds3698	3931801-3933306	0	1	1
mRNA	ID=cds3712	3950507-3951436	0	1	0
mRNA	ID=cds3875	4137743-4140244	0	1	0
mRNA	ID=cds3908	4188758-4189891	0	1	0
mRNA	ID=cds3969	4263805-4264884	0	1	1
mRNA	ID=cds3970	4265137-4266330	0	1	0
mRNA	ID=cds4013	4314105-4315241	0	1	0
mRNA	ID=cds4059	4366687-4367163	0	1	0
mRNA	ID=cds4064	4371388-4372257	0	1	0
mRNA	ID=cds4146	4449081-4450583	0	1	2
mRNA	ID=cds418	444526-445890	0	1	7
mRNA	ID=cds4223	4530460-4531206	0	1	1
mRNA	ID=cds4236	4543119-4545755	0	1	0
mRNA	ID=cds4271	4587152-4589302	0	1	0
mRNA	ID=cds4305	4623935-4625317	0	1	0.5
mRNA	ID=cds4318	4638425-4638565	0	1	0
mRNA	ID=cds466	498238-499197	0	1	0
mRNA	ID=cds482	516649-517503	0	1	0
mRNA	ID=cds555	580883-581320	0	1	1
mRNA	ID=cds60	68348-70048	0	1	0.331575
mRNA	ID=cds73	83622-83708	0	1	9
mRNA	ID=cds77	88028-89032	0	1	0
mRNA	ID=cds822	882896-884128	0	1	1
mRNA	ID=cds836	895357-896310	0	1	0
mRNA	ID=cds83	94650-96008	0	1	0
mRNA	ID=cds875	943256-944119	0	1	1
mRNA	ID=cds876	944154-944780	0	1	0
mRNA	ID=cds89	102233-103153	0	1	3
mgeRNA	gbkey=misc_feature	1630310-1650767	0	1	0
mgeRNA	gbkey=misc_feature	564025-585326	0	1	1
tRNA	product=tRNA-Tyr	4173495-4173579	0.999999	0.666666	0.333333
mRNA	ID=cds1391	1467382-1468533	0	0.666666	0
mRNA	ID=cds249	269827-270978	0	0.666666	0
mRNA	ID=cds4198	4505489-4506640	0	0.666666	0

mgeRNA	gbkey=mobile_element	1467320-1468540	0	0.666666	0
mgeRNA	gbkey=mobile_element	269765-270985	0	0.666666	0
mgeRNA	gbkey=mobile_element	4505482-4506702	0	0.666666	0
Other ncRNA		1268546-1268612	399.5	0.5	0
Other ncRNA		1269081-1269146	399.5	0.5	0
mRNA	ID=cds1362	1433784-1434917	0	0.5	0
mRNA	ID=cds231	258269-259324	0	0.5	0
mRNA	ID=cds2391	2526183-2528198	0	0.5	0.5
mRNA	ID=cds262	282425-284392	0	0.5	2
mRNA	ID=cds2670	2827835-2828800	0	0.5	2
mRNA	ID=cds2691	2850158-2851279	0	0.5	0.5
mRNA	ID=cds3645	3874163-3874975	0	0.5	1
mRNA	ID=cds773	825342-826475	0	0.5	0
tRNA	product=tRNA-Gly	1990066-1990141	23.338075	0.392858	1.03081
tRNA	product=tRNA-Gly	4390383-4390458	23.338075	0.392858	1.03081
tRNA	product=tRNA-Gly	4390495-4390570	23.338075	0.392858	1.03081
tRNA	product=tRNA-Gly	4390606-4390681	23.338075	0.392858	1.03081
mRNA	ID=cds3965	4259737-4260729	0	0.349788	0.080508
mRNA	ID=cds2302	2432104-2432763	0	0.295804	0
tRNA	product=tRNA-Gln	695653-695727	0	0.25	1
tRNA	product=tRNA-Gln	695765-695839	0	0.25	1
tRNA	product=tRNA-Gln	695979-696053	0	0.25	1
tRNA	product=tRNA-Gln	696088-696162	0	0.25	1
mRNA	ID=cds3521	3746600-3747262	0	0.210485	0
tRNA	product=tRNA-His	3980532-3980608	2.6231	0.142858	976.38752
tRNA	product=tRNA-Lys	2519275-2519350	1.20553	0.142858	0.49882
tRNA	product=tRNA-Lys	779777-779852	1.20553	0.142858	0.49882
tRNA	product=tRNA-Lys	780066-780141	1.20553	0.142858	0.49882
tRNA	product=tRNA-Lys	780370-780445	1.20553	0.142858	0.49882
tRNA	product=tRNA-Lys	780592-780667	1.20553	0.142858	0.49882
tRNA	product=tRNA-Lys	780800-780875	1.20553	0.142858	0.49882
tRNA	product=tRNA-Thr	3421602-3421677	0.930492	0.142858	0.49882
tRNA	product=tRNA-Thr	4173777-4173852	0.930492	0.142858	0.49882
mRNA	ID=cds3752	3999449-4000399	0	0.125	1
mRNA	ID=cds1767	1860795-1861790	79	0	14
mRNA	ID=cds2858	3043180-3043923	26	0	0
Other ncRNA		1921090-1921338	12	0	3
Other ncRNA		1921188-1921308	12	0	3
rRNA	product=5S ribosomal RNA of rrnA operon	4038540-4038659	2.375	0	0
rRNA	product=5S ribosomal RNA of rrnB operon	4169660-4169779	2.375	0	0
rRNA	product=5S ribosomal RNA of rrnC operon	3944723-3944842	2.375	0	0
rRNA	product=5S ribosomal RNA of rrnD operon	3421445-3421564	2.375	0	0
rRNA	product=5S ribosomal RNA of rrnD operon	3421690-3421809	2.375	0	0
rRNA	product=5S ribosomal RNA of rrnE operon	4211063-4211182	2.375	0	0
rRNA	product=5S ribosomal RNA of rrnG operon	2724091-2724210	2.375	0	0
rRNA	product=5S ribosomal RNA of rrnH operon	228756-228875	2.375	0	0
mRNA	ID=cds1629	1716517-1717626	1	0	0
mRNA	ID=cds2448	2581568-2583547	1	0	1
mRNA	ID=cds2702	2863123-2864487	1	0	0
mRNA	ID=cds2704	2865636-2866775	1	0	0
mRNA	ID=cds3016	3209129-3210874	1	0	0
mRNA	ID=cds3303	3492033-3494576	1	0	2
mRNA	ID=cds370	397096-398190	1	0	0
mRNA	ID=cds3787	4040092-4040361	1	0	0
mRNA	ID=cds3983	4279806-4281098	1	0	0
mRNA	ID=cds4143	4446715-4447065	1	0	0
mRNA	ID=cds4273	4591384-4592745	1	0	0
mRNA	ID=cds966	1048662-1048967	1	0	2

tRNA	product=tRNA-Trp	3944980-3945055	1	0	0
tRNA	product=tRNA-Val	1744540-1744616	1	0	0
mRNA	ID=cds2432	2564903-2566129	0.5	0	0
mRNA	ID=cds3836	4097514-4098548	0.5	0	0
tRNA	product=tRNA-Val	2518953-2519028	0.2	0	0
tRNA	product=tRNA-Val	2519073-2519148	0.2	0	0
tRNA	product=tRNA-Val	2519195-2519270	0.2	0	0
tRNA	product=tRNA-Val	779988-780063	0.2	0	0
tRNA	product=tRNA-Val	780291-780366	0.2	0	0
mRNA	ID=cds1017	1101769-1102419	0	0	1
mRNA	ID=cds1221	1299206-1300837	0	0	1
mRNA	ID=cds1247	1322770-1324665	0	0	1
mRNA	ID=cds1257	1333855-1336530	0	0	4
mRNA	ID=cds1306	1384744-1386285	0	0	6
mRNA	ID=cds1519	1619356-1620543	0	0	3
mRNA	ID=cds1690	1782758-1785136	0	0	3
mRNA	ID=cds190	218887-219594	0	0	5
mRNA	ID=cds1947	2028923-2030341	0	0	1
mRNA	ID=cds2054	2147063-2149009	0	0	1.5
mRNA	ID=cds2209	2325389-2326165	0	0	1
mRNA	ID=cds2211	2327820-2332424	0	0	1
mRNA	ID=cds2249	2375611-2377281	0	0	0.5
mRNA	ID=cds2308	2438407-2439627	0	0	0.5
mRNA	ID=cds2357	2487264-2488208	0	0	4
mRNA	ID=cds2383	2517279-2518694	0	0	0.5
mRNA	ID=cds2531	2677486-2680767	0	0	1
mRNA	ID=cds2541	2695376-2695879	0	0	1
mRNA	ID=cds2757	2924330-2925694	0	0	2
mRNA	ID=cds2834	3014082-3017180	0	0	10
mRNA	ID=cds2835	3017183-3018511	0	0	1
mRNA	ID=cds2958	3150258-3151445	0	0	1
mRNA	ID=cds2992	3185443-3187887	0	0	0.5
mRNA	ID=cds3006	3199913-3201151	0	0	2
mRNA	ID=cds304	320832-321551	0	0	0.5
mRNA	ID=cds3104	3300511-3301389	0	0	0.5
mRNA	ID=cds3128	3329792-3330757	0	0	1
mRNA	ID=cds3133	3333257-3334516	0	0	1
mRNA	ID=cds3139	3337278-3338087	0	0	3
mRNA	ID=cds3327	3516565-3517086	0	0	2
mRNA	ID=cds334	358023-358682	0	0	1
mRNA	ID=cds3508	3731743-3732924	0	0	1
mRNA	ID=cds3884	4151719-4152870	0	0	1
mRNA	ID=cds3929	4213501-4215102	0	0	4
mRNA	ID=cds3934	4221851-4225534	0	0	5
mRNA	ID=cds4077	4381862-4383364	0	0	1
mRNA	ID=cds4113	4419416-4419721	0	0	9
mRNA	ID=cds4150	4453808-4455181	0	0	14
mRNA	ID=cds4212	4520150-4522117	0	0	2
mRNA	ID=cds4316	4636201-4637553	0	0	2
mRNA	ID=cds436	464836-466536	0	0	1
mRNA	ID=cds486	519640-522054	0	0	14
mRNA	ID=cds502	539789-541090	0	0	2
mRNA	ID=cds575	605488-606606	0	0	1
mRNA	ID=cds609	645854-646732	0	0	0.5
mRNA	ID=cds699	743466-744398	0	0	0.691068
mRNA	ID=cds737	788054-789202	0	0	0.5
mRNA	ID=cds774	826468-828204	0	0	1
mRNA	ID=cds90	103155-103985	0	0	0.5

mRNA	ID=cds911	989845-992457	0	0	1
