# Supplementary material for: The extracellular RNA complement of Escherichia coli
Source: Microbiologyopen. 2015 Jan 21;4(2):252–66. doi: 10.1002/mbo3.235 (PMC4398507; doi:10.1002/mbo3.235)
Supplement: Supplementary file 4 — Table S3. Genomic features and corresponding read counts. [file mbo30004-0252-sd4.pdf]

Supplementary table S3: **Genomic features and corresponding read counts**

RNA biotype	Product information	Genomic coordinates	RNA_{int} (Read Counts)	RNA_{exOMV} (Read Counts)	$RNA_{exOMV-f}$ (Read Counts)
tRNA	product=tRNA-Glu	4207797-4207872	3867485.148	27837.25	473.90868
tRNA	product=tRNA-Glu	3941458-3941533	3867485.127	27837.25	473.90868
tRNA	product=tRNA-Glu	2727391-2727466	3867484.362	27837.25	473.09132
tRNA	product=tRNA-Glu	4166395-4166470	3867484.362	27837.25	473.09132
tRNA	product=tRNA-Asp	3944895-3944971	1349264.965	10286.73502	2038.428461
tRNA	product=tRNA-Asp	228928-229004	1280236.108	10266.63338	2032.628996
tRNA	product=tRNA-Asp	236931-237007	1279435.348	10264.62169	2097.263357
tRNA	product=tRNA-Thr	4173411-4173486	26872	1322	10361
tRNA	product=tRNA-Asn	2057875-2057950	99826.41409	1092	4422.91944
tRNA	product=tRNA-Asn	2056051-2056126	99798.75533	1092	4422.91944
tRNA	product=tRNA-Asn	2042573-2042648	99797.34621	1092	4422.91944
tRNA	product=tRNA-Asn	2060284-2060359	99797.34621	1092	4422.91944
rRNA	product=23S ribosomal RNA of rrnB operon	4166664-4169567	1123.464929	976.572875	1295.51554
rRNA	product=23S ribosomal RNA of rrnG operon	2724303-2727206	1122.780316	976.572875	1295.51554
rRNA	product=23S ribosomal RNA of rrnE operon	4208066-4210969	1123.291446	975.412244	1295.905004
rRNA	product=23S ribosomal RNA of rrnH operon	225759-228662	1109.729917	975.383593	1268.117289
rRNA	product=23S ribosomal RNA of rrnA operon	4035542-4038446	1287.887147	972.235825	1280.92264
rRNA	product=23S ribosomal RNA of rrnD operon	3421902-3424805	1114.615826	962.228422	1258.264467
rRNA	product=23S ribosomal RNA of rrnC operon	3941727-3944630	1130.034968	955.543475	1259.349301
Uncharacterized			147191.4509	498.122919	1107.275255
tRNA	product=tRNA-Gln	695979-696053	139558.5595	480.699416	35.111757
tRNA	product=tRNA-Gln	696088-696162	139612.4937	478.699416	37.843983
tmRNA		2753615-2753977	565	452	391
other ncRNA		475672-475785	881	373	10743
tRNA	product=tRNA-Leu	4494428-4494512	3002.986985	371	4003
other ncRNA		3054005-3054187	4050.885847	361.646447	244
tRNA	product=tRNA-His	3980532-3980608	26424.83203	349.843131	5920.578819
rRNA	product=16S ribosomal RNA of rrnH operon	223771-225312	357.677312	335.201871	512.367579
rRNA	product=16S ribosomal RNA of rrnB operon	4164682-4166223	364.21653	320.01707	523.07761
rRNA	product=16S ribosomal RNA of rrnE operon	4206170-4207711	300.754701	320.01707	523.07761
tRNA	product=tRNA-Val	1744540-1744616	800.423167	318.540018	15111.38325
rRNA	product=16S ribosomal RNA of rrnA operon	4033554-4035095	299.255533	314.85669	504.212255
rRNA	product=16S ribosomal RNA of rrnC operon	3939831-3941372	301.573741	314.618125	530.03576
rRNA	product=16S ribosomal RNA of rrnD operon	3425243-3426784	354.875545	311.5856	569.203207
rRNA	product=16S ribosomal RNA of rrnG operon	2727638-2729179	344.113452	309.542157	525.002648
tRNA	product=tRNA-Ala	225500-225575	11797.34924	285.815797	3150.425272
tRNA	product=tRNA-Ala	3424980-3425055	11797.33897	285.815797	3150.425272
tRNA	product=tRNA-Ala	4035283-4035358	11797.33897	285.815797	3150.425272
tRNA	product=tRNA-Gln	695765-695839	156306.2964	279.605166	22.522129
tRNA	product=tRNA-Gln	695653-695727	156282.6005	277.996003	22.522129
tRNA	product=tRNA-Leu	3320094-3320180	6101	277	373
tRNA	product=tRNA-Thr	262095-262170	1942.801841	270.539293	8641.557111
rRNA	product=5S ribosomal RNA of rrnC operon	3944723-3944842	2737.757667	262.480845	370.024802
rRNA	product=5S ribosomal RNA of rrnH operon	228756-228875	2737.757663	262.480845	370.024802
rRNA	product=5S ribosomal RNA of rrnD operon	3421690-3421809	2737.752288	262.480845	370.024802
rRNA	product=5S ribosomal RNA of rrnG operon	2724091-2724210	2737.752244	262.480845	370.024802
mgeRNA	gbkey=misc_feature	262182-296489	2007.031823	255.232891	8641.23896
tRNA	pseudo=true	296430-296478	1792.411717	240.799558	8622.477847
rRNA	product=5S ribosomal RNA of rrnB operon	4169660-4169779	2191.879203	216.49083	163.222862
rRNA	product=5S ribosomal RNA of rrnE operon	4211063-4211182	2191.861303	216.49083	163.222862
rRNA	product=5S ribosomal RNA of rrnA operon	4038540-4038659	2311.355311	215.394139	160.51696
rRNA	product=5S ribosomal RNA of rrnD operon	3421445-3421564	2165.475617	182.476486	176.654044
tRNA	product=tRNA-Pro	3980758-3980834	4079.982428	178	551.717119
tRNA	product=tRNA-Leu	4604223-4604309	5869.311176	175.537551	289.085933
tRNA	product=tRNA-Ser	1030848-1030935	148018	175	109
tRNA	product=tRNA-Leu	1989839-1989925	708	172	1543
tRNA	product=tRNA-Leu	4604102-4604188	5640.412993	166.154005	253.304955
tRNA	product=tRNA-Leu	3980629-3980715	5628.615412	166.154005	253.304955
tRNA	product=tRNA-Leu	4604338-4604424	5627.664194	166.154005	253.304955
tRNA	product=tRNA-Thr	3421602-3421677	1439.324151	155.347265	3258.18638
tRNA	product=tRNA-Val	779988-780063	34645.85043	148.975415	381.028186
tRNA	product=tRNA-Met	2945409-2945485	567.77234	145.846697	1131.911262
tRNA	product=tRNA-Met	2945629-2945705	570.633732	145.430434	1131.911262
tRNA	product=tRNA-Met	2945519-2945595	573.878282	144.430434	1131.911262
tRNA	product=tRNA-Cys	1989938-1990011	1183.970358	137	2360
tRNA	product=tRNA-Thr	4173777-4173852	1085.889473	131.463653	6039.072882
tRNA	product=tRNA-Pro	2284233-2284309	12199.90666	129	357.174342
tRNA	product=tRNA-Met	3316235-3316311	738.715487	89.29207	1095.26357
tRNA	product=tRNA-Ser	2816575-2816667	16985.94156	84	240
tRNA	product=tRNA-Gly	2997006-2997079	1654	81	11595
tRNA	product=tRNA-Val	1744459-1744535	1196.038525	79.413973	2881.4122
tRNA	product=tRNA-Ala	2516178-2516253	1194.54097	73.983622	1171.205326
tRNA	product=tRNA-Ala	2516063-2516138	1190.152816	72.500296	1154.017579
tRNA	product=tRNA-Val	2519073-2519148	21409.79055	69.235724	368.673348
tRNA	product=tRNA-Val	780291-780366	21333.1808	68.855007	367.580391
tRNA	product=tRNA-Pro	3706639-3706715	4025.945597	68.5	2348.695342
tRNA	product=tRNA-Val	2519195-2519270	21413.88082	66.921465	368.673348
tRNA	product=tRNA-Tyr	1286467-1286551	281.118706	58.158121	232.712761
tRNA	product=tRNA-Tyr	1286761-1286845	251.664327	58.158121	232.712761
mRNA	ID=cds4150	4453808-4455181	32.837779	57	0
tRNA	product=tRNA-Gly	4173696-4173770	2301	56.883527	821
tRNA	product=tRNA-Arg	3980398-3980474	7546.547897	56.748272	207.340496
tRNA	product=tRNA-Ile	4035164-4035240	723.666697	55.333278	3894.662772
tRNA	product=tRNA-Ile	225381-225457	723.665571	55.333278	3894.662772
tRNA	product=tRNA-Ile	3425098-3425174	723.665571	55.333278	3894.662772
tRNA	product=tRNA-Tyr	4173495-4173579	166.997074	53.336196	206.939019
tRNA	product=tRNA-Val	2518953-2519028	4710.536999	52.029683	355.469836
tRNA	product=tRNA-Ser	925107-925194	28438.5402	46.287868	10.5
tRNA	product=tRNA-Ser	1096788-1096875	28438.41439	46.287868	10.5
tRNA	product=tRNA-Trp	3944980-3945055	2089	46	1052
other ncRNA		1921090-1921338	4907	44	405
mRNA	ID=cds285	304398-306041	12.360041	42	0
mgeRNA	gbkey=misc_feature	564025-585326	7348.741678	39.7	10.656513
tRNA	product=tRNA-Sec	3834245-3834339	1819	39	1661
other ncRNA		2732175-2732317	2575	38	369
tRNA	product=tRNA-Leu	696186-696270	4267	37	305
tRNA	product=tRNA-Arg	563946-564022	7209.82561	36	9.254598
tRNA	pseudo=true	585280-585324	7103.17439	35	5.745402
tRNA	product=tRNA-Arg	2816220-2816296	694.14749	32.535613	218.062433
tRNA	product=tRNA-Arg	2816081-2816157	729.539044	31.470553	218.062433
tRNA	product=tRNA-Arg	2815806-2815882	698.067899	31.470553	218.062433
mRNA	ID=cds2065	2162300-2163022	8	31	0
tRNA	product=tRNA-Arg	2816495-2816571	688.528098	30.263014	217.367561
mRNA	ID=cds3738	3982375-3984030	9	30	0
tRNA	product=tRNA-Ser	2041492-2041581	231	27	125
other ncRNA		3268238-3268614	24.407707	26.758571	41.360888
mRNA	ID=cds111	127912-129336	72	26	0.5
tRNA	product=tRNA-Phe	3108388-3108463	280.763344	20	516.5
tRNA	product=tRNA-Phe	4360574-4360649	262.236656	20	516.5
other ncRNA		1921188-1921308	1646	19	124
tRNA	product=tRNA-Met	695887-695963	211.101751	17.523004	197.102274
tRNA	product=tRNA-Met	696280-696356	253.436558	16.523004	197.102274
tRNA	product=tRNA-Gly	4390383-4390458	194.819389	15.061899	262.519461
tRNA	product=tRNA-Gly	4390495-4390570	193.817692	15.061899	262.519461
tRNA	product=tRNA-Gly	4390606-4390681	193.817692	15.061899	262.519461
tRNA	product=tRNA-Gly	1990066-1990141	193.239843	15.061899	262.519461
mRNA	ID=cds411	437539-439401	59	15	0
mRNA	ID=cds4244	4553513-4554343	1627	13	0
mgeRNA	gbkey=misc_feature	1195443-1210635	595.5	12	26
tRNA	product=tRNA-Lys	780066-780141	428.144303	11.35795	152.502193
tRNA	product=tRNA-Lys	779777-779852	426.691651	11.35795	152.502193
tRNA	product=tRNA-Lys	780592-780667	426.691651	11.35795	152.502193
tRNA	product=tRNA-Lys	2519275-2519350	425.757121	11.35795	152.502193
tRNA	product=tRNA-Lys	780370-780445	425.599697	11.35795	152.502193
tRNA	product=tRNA-Lys	780800-780875	422.830194	11.35795	152.502193
mRNA	ID=cds3840	4101625-4102998	14	11	2
mRNA	ID=cds2571	2729622-2732195	1849	10	16
mRNA	ID=cds3842	4103843-4104343	135	10	18
other ncRNA		2689179-2689362	16	10	2
mRNA	ID=cds12	12163-14079	201.333333	9	10
mRNA	ID=cds3227	3436046-3436456	18	9	2
mRNA	ID=cds264	286013-287623	2	9	2.5
tRNA	product=tRNA-Arg	2464331-2464405	359	8	68.8934
mRNA	ID=cds2080	2174372-2175226	580.731519	7	10

mgeRNA	gbkey=misc_feature	1409923-1432982	304.917606	7	4.5
mRNA	ID=cds792	847631-848134	86.808016	7	1
mRNA	ID=cds3544	3769405-3769767	2	7	0
tRNA	product=tRNA-Ile	3213620-3213695	42.552351	6.711325	701.383236
mRNA	ID=cds2984	3176137-3177618	84.166667	6	13
mRNA	ID=cds3890	4159147-4159794	46	6	1
mRNA	ID=cds1772	1866979-1868262	8	6	0
other ncRNA		506428-506511	2396	5	2
mRNA	ID=cds2220	2346844-2347494	168	5	4
mRNA	ID=cds3232	3438062-3439051	167	5	1
mRNA	ID=cds4062	4369048-4370694	135.289855	5	13
mRNA	ID=cds144	164730-167264	46	5	0
other ncRNA		2922178-2922546	19	5	11
mRNA	ID=cds1610	1698981-1700153	7	5	2
other ncRNA		3054871-3055010	708.955891	4.773545	16.333336
mRNA	ID=cds705	748945-751392	0	4.5	0
other ncRNA		1490143-1490198	4730	4	12
mRNA	ID=cds1406	1489986-1490153	4628	4	14
other ncRNA		4049059-4049303	1023	4	2
mRNA	ID=cds3152	3347103-3347831	842	4	7
other ncRNA		3662887-3662991	771	4	230.58167
mRNA	ID=cds2575	2735176-2735517	649	4	8
mRNA	ID=cds2199	2309668-2310771	544.49429	4	2
repeat_region		3390098-3390405	536.739579	4	21.111037
mRNA	ID=cds2079	2173081-2174343	464.67047	4	4
mRNA	ID=cds936	1018236-1019276	388.482678	4	24
mRNA	ID=cds3454	3664203-3665603	337.691113	4	13
mRNA	ID=cds860	921589-921813	319	4	47
mRNA	ID=cds4096	4402710-4404008	207	4	6
mRNA	ID=cds1479	1568669-1570069	204.418167	4	12
mRNA	ID=cds652	688566-690104	55	4	2
mRNA	ID=cds233	260727-261980	14	4	0
mRNA	ID=cds3469	3688291-3690630	3	4	0
mRNA	ID=cds4267	4581272-4584784	106	3.5	2
mRNA	ID=cds3698	3931801-3933306	92	3.5	1
mRNA	ID=cds4015	4316029-4316787	0	3.5	0.5
other ncRNA		1268546-1268612	2036.685968	3	0.19696
other ncRNA		3656009-3656077	1742.462808	3	29
other ncRNA		16952-17006	1575	3	0
mRNA	ID=cds3237	3440788-3442119	541.867712	3	20
mRNA	ID=cds951	1032477-1034270	401	3	4
repeat_region		3545909-3545995	261.298563	3	3.198933
other ncRNA		2311106-2311198	223	3	1
mRNA	ID=cds945	1027627-1027944	164	3	5
mRNA	ID=cds93	106557-107474	118.333334	3	7
mRNA	ID=cds2502	2645348-2650309	101.609905	3	3
mRNA	ID=cds720	772265-773404	98.723888	3	1
mRNA	ID=cds3904	4178583-4178948	97.318182	3	5
mRNA	ID=cds4090	4397275-4398225	89.5	3	3
mRNA	ID=cds2742	2906051-2907688	83.040161	3	5
mRNA	ID=cds949	1029982-1030641	75	3	1
mRNA	ID=cds82	93166-94653	75	3	0
mRNA	ID=cds2986	3178443-3179603	42	3	2
mRNA	ID=cds163	189874-190599	42	3	0
mRNA	ID=cds4143	4446715-4447065	24	3	3
mRNA	ID=cds2593	2749817-2751478	20	3	1
mRNA	ID=cds3467	3683723-3687196	17	3	2
mRNA	ID=cds4136	4437610-4437816	10	3	2
mRNA	ID=cds2105	2207122-2209122	6	3	0
mRNA	ID=cds852	910405-911373	4	3	0
mRNA	ID=cds1628	1716090-1716419	3	3	2
other ncRNA		1269081-1269146	4413.56629	2.5	2.19696
mRNA	ID=cds3122	3323023-3324957	137	2.5	4
mRNA	ID=cds2549	2700503-2701408	50	2.5	0
mRNA	ID=cds3030	3229687-3231705	6	2.5	1
mgeRNA	gbkey=misc_feature	2065378-2077055	282.352916	2.333334	4.54762
mRNA	ID=cds472	506510-507304	2094	2	0
mRNA	ID=cds3930	4215132-4216436	1823	2	2
mRNA	ID=cds3799	4054648-4056057	946	2	2
mRNA	ID=cds15	16751-16960	509	2	0
mRNA	ID=cds2076	2170945-2172300	429.393044	2	7
mRNA	ID=cds1318	1395696-1396646	415	2	1
mRNA	ID=cds3657	3886753-3888168	337	2	0
mRNA	ID=cds7	8238-9191	331	2	3
other ncRNA		3192745-3192887	308.932086	2	2.778019
mgeRNA	gbkey=misc_feature	2753979-2776008	266.751977	2	10
mRNA	ID=cds3697	3931374-3931793	205.331114	2	2
other ncRNA		1762737-1762804	196.959787	2	2
mRNA	ID=cds3277	3469422-3471536	190	2	3
mRNA	ID=cds1665	1755445-1755681	187.808057	2	8
mRNA	ID=cds3743	3989176-3991722	178	2	12
mRNA	ID=cds2567	2717975-2720635	176.211325	2	3
mRNA	ID=cds1	337-2799	166	2	1
repeat_region		761985-762151	152	2	2.013654
mRNA	ID=cds3465	3680184-3681470	137	2	3
mRNA	ID=cds1868	1958086-1959819	130	2	0
mRNA	ID=cds3222	3430458-3431582	126	2	5
mRNA	ID=cds714	762237-763403	124	2	1
mRNA	ID=cds727	776963-778255	104	2	39
mRNA	ID=cds2741	2904665-2905963	103.755923	2	6
mRNA	ID=cds3278	3471564-3472103	101	2	0
mRNA	ID=cds1701	1793581-1795968	97	2	2
mRNA	ID=cds110	125695-127587	94	2	1
mRNA	ID=cds463	494344-496218	93	2	1
mRNA	ID=cds3968	4262337-4263752	92.5	2	10
mRNA	ID=cds4296	4613538-4615088	90	2	7
mRNA	ID=cds3650	3880349-3881752	86	2	2
mRNA	ID=cds3006	3199913-3201151	85	2	4
mRNA	ID=cds2556	2707459-2708034	82.5	2	3
mRNA	ID=cds2883	3070694-3071713	78	2	3
mRNA	ID=cds3563	3788343-3789368	73.287742	2	1
mRNA	ID=cds3303	3492033-3494576	73	2	1
mRNA	ID=cds1651	1739437-1740585	69	2	7
mRNA	ID=cds3114	3311364-3314036	69	2	0
mRNA	ID=cds3658	3888259-3889506	63	2	1
mRNA	ID=cds418	444526-445890	63	2	1
mRNA	ID=cds3151	3346474-3347106	61	2	2
mRNA	ID=cds1068	1148951-1149880	61	2	0
mRNA	ID=cds4094	4401323-4402327	59.193996	2	0
mRNA	ID=cds2460	2596904-2597782	56	2	1
mRNA	ID=cds959	1039840-1041138	52.992425	2	1
mRNA	ID=cds1367	1441075-1443714	50.652596	2	1
mRNA	ID=cds2586	2743392-2743940	48	2	0
mRNA	ID=cds3249	3446590-3446781	45	2	3
mRNA	ID=cds3242	3443629-3444162	43	2	2
mRNA	ID=cds3434	3641163-3643205	40.209165	2	0
mRNA	ID=cds2365	2495079-2496317	38	2	0
mRNA	ID=cds2249	2375611-2377281	37	2	1
mRNA	ID=cds474	508099-510603	36.333333	2	1
mRNA	ID=cds3857	4116868-4117353	36	2	3
mRNA	ID=cds657	694324-695499	35	2	5
mRNA	ID=cds697	742050-742793	32	2	0
mRNA	ID=cds3695	3927620-3929116	30.632215	2	1
mRNA	ID=cds4152	4455982-4457334	30	2	0
mRNA	ID=cds2550	2701405-2702085	28	2	1
mRNA	ID=cds2748	2913079-2915835	27	2	3
other ncRNA		3984455-3984626	25	2	1
mRNA	ID=cds2317	2447250-2448071	25	2	0
mRNA	ID=cds4055	4361368-4363065	24	2	2
mRNA	ID=cds2565	2716757-2717176	24	2	0
mRNA	ID=cds2831	3010636-3012261	21	2	0
mRNA	ID=cds2325	2455037-2457181	20.191943	2	1
mRNA	ID=cds3346	3538429-3540750	19.416666	2	1
mRNA	ID=cds3126	3326985-3328418	18	2	1
mRNA	ID=cds3676	3907462-3908421	16	2	2
mRNA	ID=cds3920	4198589-4199284	13	2	0
mRNA	ID=cds4291	4609419-4610024	11	2	2
mRNA	ID=cds502	539789-541090	8	2	1

mRNA	ID=cds2211	2327820-2332424	7	2	1
mRNA	ID=cds1788	1878145-1878783	7	2	0
mRNA	ID=cds2536	2685491-2686825	7	2	0
mRNA	ID=cds3953	4247577-4248497	6	2	2
mRNA	ID=cds587	621523-622773	4	2	28.25
other ncRNA		4275950-4276089	4	2	16
mRNA	ID=cds3337	3527370-3527771	4	2	0
mRNA	ID=cds2474	2611956-2612804	3	2	0
mRNA	ID=cds4033	4333717-4335054	3	2	0
mRNA	ID=cds1287	1366103-1366771	2.223607	2	1
mRNA	ID=cds1339	1416695-1417183	2	2	0
mRNA	ID=cds1995	2082491-2083549	2	2	0
mRNA	ID=cds3317	3506609-3507835	2	2	0
mRNA	ID=cds3631	3860010-3861626	2	2	0
mRNA	ID=cds3805	4062386-4063789	2	2	0
mRNA	ID=cds1491	1586877-1588103	0.5	2	0
mRNA	ID=cds1984	2075136-2075504	0.183892	2	0
mRNA	ID=cds2028	2113931-2115151	0	2	1
mRNA	ID=cds1301	1378845-1379801	0	2	0
repeat_region		507806-508041	106.907494	1.627595	2.218815
mRNA	ID=cds3276	3468167-3469351	158.400061	1.5	1
mRNA	ID=cds3898	4173967-4175151	156.599939	1.5	1
mRNA	ID=cds637	671424-674006	110.233854	1.5	5
mRNA	ID=cds455	485760-489122	46.195047	1.5	0
mRNA	ID=cds3916	4195739-4196803	26	1.5	0
mRNA	ID=cds3196	3401506-3402480	14	1.5	0
mRNA	ID=cds1086	1166822-1167361	11	1.5	0
mRNA	ID=cds2410	2546124-2547428	3	1.5	0
mRNA	ID=cds2465	2601869-2602816	1	1.5	0
repeat_region		4151370-4151715	101.304534	1.071429	12.029755
mRNA	ID=cds521	558920-561523	4.003163	1.050543	0.064333
mRNA	ID=cds2781	2957082-2960450	25.005958	1.019182	2.557397
repeat_region		757632-757749	576.050801	1.015372	1.424135
mRNA	ID=cds1221	1299206-1300837	1397.811018	1	10
mRNA	ID=cds4065	4372652-4373680	1207	1	0
mRNA	ID=cds711	756912-757628	631.293195	1	2
mRNA	ID=cds1821	1910792-1912840	570.333334	1	2
repeat_region		4051553-4051636	477.294793	1	0.020988
mRNA	ID=cds3443	3651984-3652550	424	1	7
mRNA	ID=cds4058	4364914-4366350	417.587139	1	11
other ncRNA		2812824-2812901	397	1	13
mRNA	ID=cds1478	1566978-1568513	379.894206	1	14
mRNA	ID=cds3106	3302595-3303839	373.708614	1	11
other ncRNA		3309247-3309420	356	1	1
mRNA	ID=cds2268	2395461-2398187	344.133833	1	3
mRNA	ID=cds816	877471-877854	338.896784	1	5
mRNA	ID=cds3447	3654431-3654763	336.913888	1	3
mgeRNA	gbkey=misc_feature	1630310-1650767	312.380123	1	5.214285
mRNA	ID=cds1600	1684755-1686401	261.848783	1	0
mRNA	ID=cds2570	2722470-2723768	254	1	3
mRNA	ID=cds3499	3722430-3723341	226	1	1
mRNA	ID=cds1122	1202491-1203048	197	1	16
mRNA	ID=cds174	202101-202556	189	1	0
mRNA	ID=cds1906	1995086-1995838	188	1	0
mRNA	ID=cds3823	4080795-4083845	186.067367	1	4.5
mRNA	ID=cds3906	4183373-4187596	182.392139	1	6
other ncRNA		4047922-4048030	179.957924	1	11
mRNA	ID=cds3364	3562157-3564604	177.006309	1	4
mRNA	ID=cds113	131615-134212	168.661105	1	4
mRNA	ID=cds1690	1782758-1785136	165.940991	1	2
mRNA	ID=cds3059	3256307-3257671	160	1	1
mRNA	ID=cds3721	3962388-3963653	159.75	1	11
mRNA	ID=cds1767	1860795-1861790	158	1	8
mRNA	ID=cds3482	3704121-3705728	156	1	3
mRNA	ID=cds3654	3883099-3884745	154.389182	1	2
mRNA	ID=cds1104	1186342-1187463	151	1	2
mRNA	ID=cds4075	4378533-4380341	149	1	5
mRNA	ID=cds1216	1291732-1292145	149	1	2
repeat_region		2835487-2835513	146	1	0
mRNA	ID=cds4035	4336277-4338544	144	1	1
mRNA	ID=cds1707	1798666-1800594	142	1	4
mRNA	ID=cds2267	2394487-2395464	137	1	0.5
mRNA	ID=cds2997	3189761-3189961	136	1	3
mRNA	ID=cds1060	1140405-1143590	130.666667	1	1
mRNA	ID=cds399	426871-428718	129	1	2
mRNA	ID=cds3367	3567369-3569342	127	1	0
mRNA	ID=cds719	770681-772249	126.333333	1	6
repeat_region		2346803-2346838	124	1	0
repeat_region		2137726-2137762	123.502895	1	0
mRNA	ID=cds109	123017-125680	120.5	1	2
mRNA	ID=cds3326	3515420-3516508	117	1	0
mRNA	ID=cds869	932447-936436	116.5	1	5
mRNA	ID=cds3241	3443266-3443619	115	1	0
mRNA	ID=cds1706	1798120-1798662	114	1	3
mRNA	ID=cds3686	3918973-3919212	113	1	1
mRNA	ID=cds3514	3739707-3740555	107	1	1
mRNA	ID=cds2890	3077666-3079657	102	1	1
mRNA	ID=cds2075	2169857-2170897	99.162926	1	5
mRNA	ID=cds2512	2658339-2659553	98	1	3
mRNA	ID=cds1070	1150838-1151074	97.874965	1	0
mRNA	ID=cds3822	4079880-4080782	97.028356	1	0
mRNA	ID=cds52	54755-57109	97	1	6
mRNA	ID=cds2214	2334815-2337442	95.35314	1	4
mRNA	ID=cds3687	3919259-3920074	95	1	4
mRNA	ID=cds3200	3403939-3405288	94.795876	1	0
mRNA	ID=cds3708	3948345-3948443	94	1	1
mRNA	ID=cds2277	2407542-2409374	92.758027	1	0.021828
mRNA	ID=cds2606	2763940-2765013	92	1	0
mRNA	ID=cds619	656515-656724	91	1	4
mRNA	ID=cds903	975549-980009	90.333333	1	5
mRNA	ID=cds3017	3211069-3212910	86.329217	1	4
mRNA	ID=cds1727	1819942-1820280	86	1	5
mRNA	ID=cds2555	2706776-2707426	84.685327	1	0
mRNA	ID=cds3778	4024550-4025251	79	1	1
mRNA	ID=cds3341	3532538-3533890	79	1	0
repeat_region		2302439-2303093	78.111587	1	25.701759
mRNA	ID=cds4312	4632464-4633333	76	1	5
mRNA	ID=cds3240	3442748-3443251	76	1	1
mRNA	ID=cds1206	1279087-1282830	75.5	1	3
mRNA	ID=cds2947	3139308-3141011	73	1	1
mRNA	ID=cds452	480478-483627	72.007694	1	2
mRNA	ID=cds484	518363-518989	72	1	4
mRNA	ID=cds3682	3915425-3916288	72	1	0
mRNA	ID=cds1902	1990898-1992730	71.776393	1	2
mRNA	ID=cds13	14168-15298	71	1	3
mRNA	ID=cds4089	4395435-4397282	71	1	3
mRNA	ID=cds3173	3374804-3375442	71	1	1
mRNA	ID=cds1306	1384744-1386285	71	1	0
mRNA	ID=cds3146	3342739-3344172	70.6	1	12
mRNA	ID=cds794	849673-850188	70.303868	1	3
mRNA	ID=cds3123	3325057-3325686	68	1	3
mRNA	ID=cds1276	1352529-1353494	68	1	0
mRNA	ID=cds3036	3235333-3236319	67.5	1	3
mRNA	ID=cds1224	1302778-1303791	67	1	0
mRNA	ID=cds4157	4462782-4464203	67	1	0
mRNA	ID=cds946	1028002-1029192	65	1	0
mRNA	ID=cds3190	3394348-3395817	64.639959	1	4
mRNA	ID=cds832	891190-892092	64.000459	1	1
mRNA	ID=cds3325	3514042-3515328	63.128187	1	1
mRNA	ID=cds2893	3081957-3083933	63	1	2
mRNA	ID=cds1713	1805820-1806680	62	1	0
mRNA	ID=cds400	428729-429700	62	1	0
mRNA	ID=cds3448	3655018-3655590	61.162716	1	0
mRNA	ID=cds2562	2714088-2714471	60.731384	1	5
mRNA	ID=cds87	99644-100711	60.204124	1	1
mRNA	ID=cds1833	1923132-1923362	60	1	7
mRNA	ID=cds908	985117-986205	59.930091	1	0
repeat_region		3137643-3137725	59.404033	1	1.472051

repeat_region		899972-900058	58.126235	1	2.711325
mRNA	ID=cds1770	1863750-1864496	57.732739	1	0
mRNA	ID=cds405	432679-433782	57	1	3
mRNA	ID=cds1105	1187539-1188999	56.012362	1	18
mRNA	ID=cds1189	1262937-1264193	55	1	2
mRNA	ID=cds2896	3084728-3085882	54.562015	1	0
mRNA	ID=cds3903	4178019-4178516	54	1	6
mRNA	ID=cds1602	1687876-1689384	53.185482	1	0
mRNA	ID=cds3365	3564623-3566056	53	1	2
mRNA	ID=cds3120	3320755-3322092	53	1	1
mRNA	ID=cds1225	1303788-1304792	53	1	0
mRNA	ID=cds1752	1844989-1846032	53	1	0
mRNA	ID=cds4155	4458545-4460683	53	1	0
mRNA	ID=cds3112	3309855-3310799	52	1	0
mRNA	ID=cds656	692754-694178	51	1	1
mRNA	ID=cds3892	4160193-4161293	50.723358	1	2
mRNA	ID=cds1822	1912860-1913558	50	1	2
mRNA	ID=cds3286	3475929-3476519	50	1	1
mRNA	ID=cds1731	1823164-1823649	49	1	1
mRNA	ID=cds2483	2621066-2623132	48	1	4
mRNA	ID=cds3246	3445475-3445789	48	1	1
mRNA	ID=cds1692	1786459-1787505	47	1	1
mRNA	ID=cds3934	4221851-4225534	46.646447	1	1
mRNA	ID=cds4085	4390951-4392090	45	1	13
mRNA	ID=cds2097	2191081-2192190	45	1	0
mRNA	ID=cds3652	3882516-3882875	45	1	0
mRNA	ID=cds3800	4056430-4058253	44.5927	1	0
mRNA	ID=cds2489	2628980-2630557	44.244077	1	0
mRNA	ID=cds2655	2812905-2814461	44.177518	1	3
mRNA	ID=cds1960	2042962-2050038	44	1	1
mRNA	ID=cds2778	2948657-2950483	43	1	1
other ncRNA		1768396-1768501	42.556005	1	4
mRNA	ID=cds3405	3604474-3606672	42.307318	1	1
mRNA	ID=cds871	937217-938560	42	1	4
mRNA	ID=cds3174	3375837-3376229	42	1	0
mRNA	ID=cds3987	4285787-4287223	42	1	0
mRNA	ID=cds669	710158-710688	41.521398	1	1
mRNA	ID=cds932	1014938-1015105	41	1	6
mRNA	ID=cds3244	3444601-3444906	41	1	5
mRNA	ID=cds3740	3985908-3987089	41	1	1
mRNA	ID=cds707	752408-753691	40.5	1	1
mRNA	ID=cds454	484985-485632	40	1	7
mRNA	ID=cds3180	3381352-3382290	40	1	1
mRNA	ID=cds934	1015762-1017522	39.5	1	1
mRNA	ID=cds3453	3663009-3663833	39	1	0
mRNA	ID=cds4072	4377030-4377389	39	1	0
mRNA	ID=cds1897	1986740-1987237	38.417029	1	1
mRNA	ID=cds3859	4118439-4119770	38	1	1
mRNA	ID=cds3177	3378213-3378611	38	1	0
mRNA	ID=cds3870	4131858-4134038	38	1	0
mRNA	ID=cds2757	2924330-2925694	37	1	1
mRNA	ID=cds3003	3194823-3197663	37	1	1
mRNA	ID=cds4127	4427887-4429299	37	1	1
mRNA	ID=cds3104	3300511-3301389	37	1	0
mRNA	ID=cds3235	3440137-3440493	37	1	0
mRNA	ID=cds1856	1945435-1946175	36.5	1	0
mRNA	ID=cds1581	1665368-1666588	36.469978	1	1
mRNA	ID=cds2828	3006786-3007997	36	1	1
mRNA	ID=cds740	791539-793011	36	1	0
mRNA	ID=cds1167	1234932-1236464	35	1	1
mRNA	ID=cds2695	2855115-2857676	35	1	1
mRNA	ID=cds650	686062-686970	35	1	1
mRNA	ID=cds2284	2416656-2417198	35	1	0
mRNA	ID=cds482	516649-517503	35	1	0
mRNA	ID=cds1751	1843023-1844984	34.007014	1	1
mRNA	ID=cds2869	3055200-3056432	34	1	2
mRNA	ID=cds1259	1337354-1338118	34	1	1
mRNA	ID=cds3284	3474629-3475441	34	1	1
mRNA	ID=cds1208	1284362-1285072	33.5	1	0
mRNA	ID=cds3488	3712084-3714417	33.288675	1	0
mRNA	ID=cds2132	2234765-2235775	33	1	0
mRNA	ID=cds2184	2295043-2295666	33	1	0
mRNA	ID=cds2218	2345406-2346536	33	1	0
mRNA	ID=cds3156	3352747-3357207	32.652596	1	0
mRNA	ID=cds1213	1288468-1289373	32	1	4
other ncRNA		2974326-2974407	32	1	1
mRNA	ID=cds198	231122-231922	32	1	1
mRNA	ID=cds1215	1290680-1291588	32	1	0
mRNA	ID=cds2706	2867535-2868296	32	1	0
mRNA	ID=cds3332	3519994-3520773	32	1	0
mRNA	ID=cds2881	3068187-3069266	31.695256	1	0
mRNA	ID=cds4299	4617626-4618849	31	1	3
mRNA	ID=cds1207	1282827-1284365	31	1	1
mRNA	ID=cds2490	2630626-2632092	30.5	1	1
mRNA	ID=cds160	185123-185947	30.5	1	0
mRNA	ID=cds2497	2638708-2639826	30	1	3
mRNA	ID=cds3143	3340858-3341433	30	1	0
mRNA	ID=cds4121	4423862-4424089	30	1	0
mRNA	ID=cds941	1023694-1025748	30	1	0
mRNA	ID=cds3178	3378765-3380132	29	1	1
mRNA	ID=cds3409	3608539-3609756	29	1	1
mRNA	ID=cds4149	4452634-4453632	29	1	1
mRNA	ID=cds89	102233-103153	28.811018	1	2
mRNA	ID=cds94	107705-108217	28.70244	1	2
mRNA	ID=cds1247	1322770-1324665	28.5	1	2
other ncRNA		2974124-2974211	28	1	2
mRNA	ID=cds381	406203-406394	28	1	2
mRNA	ID=cds1626	1713972-1715246	28	1	1
mRNA	ID=cds424	451294-452769	28	1	0
mRNA	ID=cds658	696736-698400	28	1	0
mRNA	ID=cds1410	1494880-1496535	27	1	1.020259
mRNA	ID=cds1269	1345002-1346936	27	1	0
mRNA	ID=cds844	902229-902957	27	1	0
repeat_region		1061630-1061763	26.186247	1	4.743505
mRNA	ID=cds23	21407-22348	26	1	1
mRNA	ID=cds1180	1250289-1252208	26	1	0
mRNA	ID=cds1254	1331879-1332853	26	1	0
mRNA	ID=cds4043	4348054-4349685	26	1	0
mRNA	ID=cds3000	3192788-3192847	25.493381	1	0.5
mRNA	ID=cds1714	1806721-1807257	25	1	2
mRNA	ID=cds698	742816-743472	25	1	2
mRNA	ID=cds2852	3038438-3038845	25	1	1
mRNA	ID=cds1691	1785469-1786302	25	1	0
mRNA	ID=cds2607	2765006-2765377	25	1	0
mRNA	ID=cds3773	4020241-4020756	25	1	0
mRNA	ID=cds3901	4176470-4176898	24.672673	1	0
mRNA	ID=cds928	1009187-1011094	24	1	2
mRNA	ID=cds2683	2842784-2844493	24	1	0
mRNA	ID=cds3655	3884851-3886215	24	1	0
mRNA	ID=cds4036	4338743-4339651	24	1	0
mRNA	ID=cds447	476291-477841	23.204124	1	0
mRNA	ID=cds1304	1382141-1383538	22.5	1	0
mRNA	ID=cds182	212331-213629	22	1	1
mRNA	ID=cds1165	1232399-1233940	22	1	0
mRNA	ID=cds1912	2000134-2001630	22	1	0
mRNA	ID=cds2853	3038826-3039092	22	1	0
mRNA	ID=cds3449	3656389-3656916	22	1	0
mRNA	ID=cds3107	3303993-3305882	21.146447	1	4
mRNA	ID=cds3043	3244674-3245450	21	1	0
mRNA	ID=cds4041	4345427-4346767	20	1	2
mRNA	ID=cds1903	1992727-1993383	20	1	0
mRNA	ID=cds3192	3396409-3396897	20	1	0
mRNA	ID=cds1460	1545425-1548472	19.902321	1	2.5
mRNA	ID=cds1820	1909719-1910600	19.5	1	5
mRNA	ID=cds1029	1113487-1114713	19	1	0
mRNA	ID=cds1035	1118691-1119809	19	1	0
mRNA	ID=cds1209	1285072-1285749	19	1	0
mRNA	ID=cds140	160149-160604	19	1	0

mRNA	ID=cds1963	2053085-2054539	19	1	0
mRNA	ID=cds2637	2796113-2796517	19	1	0
mRNA	ID=cds398	426511-426843	19	1	0
mRNA	ID=cds563	590164-592401	18.528595	1	0
mRNA	ID=cds1631	1718414-1718848	18.17854	1	2
mRNA	ID=cds937	1019633-1020142	18	1	3
mRNA	ID=cds2902	3090959-3091522	18	1	2
mRNA	ID=cds2835	3017183-3018511	18	1	1
mRNA	ID=cds2836	3018562-3019341	18	1	1
mRNA	ID=cds1075	1154985-1155989	18	1	0
mRNA	ID=cds3142	3340295-3340861	18	1	0
mRNA	ID=cds847	904136-904966	18	1	0
mRNA	ID=cds3774	4020759-4021535	17.646447	1	0
mRNA	ID=cds2538	2687693-2689120	17.5	1	1
mRNA	ID=cds1495	1590689-1596011	17	1	8
mRNA	ID=cds3217	3427788-3428045	17	1	5
mRNA	ID=cds2623	2776168-2780748	17	1	2
mRNA	ID=cds1194	1267388-1268242	17	1	0
mRNA	ID=cds120	141431-141967	17	1	0
mRNA	ID=cds420	446941-447270	17	1	0
mRNA	ID=cds870	936595-937206	16	1	2
mRNA	ID=cds2116	2217714-2220011	16	1	1
mRNA	ID=cds1274	1350660-1351652	16	1	0
mRNA	ID=cds1421	1504805-1506766	16	1	0
mRNA	ID=cds202	234816-235538	16	1	0
mRNA	ID=cds867	930308-931273	16	1	0
mRNA	ID=cds2996	3188654-3189718	15.327327	1	1
mRNA	ID=cds1624	1712401-1713006	15	1	1
mRNA	ID=cds180	209679-211820	15	1	0
mRNA	ID=cds3136	3335278-3335913	15	1	0
mRNA	ID=cds3767	4014454-4015215	15	1	0
mRNA	ID=cds746	796836-797654	14.717962	1	0
mRNA	ID=cds1080	1161470-1161847	14.353553	1	2
mRNA	ID=cds3844	4105575-4106537	14.12703	1	0
mRNA	ID=cds1365	1439345-1439767	14	1	11
mRNA	ID=cds3569	3794971-3796230	14	1	4
mRNA	ID=cds1327	1406074-1407057	14	1	0
mRNA	ID=cds1614	1702973-1703188	14	1	0
mRNA	ID=cds2475	2612842-2613903	14	1	0
mRNA	ID=cds2841	3026546-3027034	14	1	0
mRNA	ID=cds3710	3949565-3950227	14	1	0
mRNA	ID=cds3860	4119780-4120310	14	1	0
mRNA	ID=cds3896	4171105-4172070	14	1	0
mRNA	ID=cds3127	3328604-3329776	13.73178	1	7
mRNA	ID=cds891	963051-963335	13	1	2
mRNA	ID=cds153	177662-178462	13	1	1
mRNA	ID=cds3436	3644322-3645674	13	1	1
repeat_region		1927947-1928030	13	1	0
mRNA	ID=cds1848	1939675-1940607	13	1	0
mRNA	ID=cds2793	2969619-2970659	13	1	0
mRNA	ID=cds2821	2997158-2997913	13	1	0
mRNA	ID=cds2989	3181835-3182488	13	1	0
mRNA	ID=cds3258	3450981-3451292	13	1	0
mRNA	ID=cds3312	3502074-3502805	13	1	0
mRNA	ID=cds1591	1674395-1675927	12.5	1	1
mRNA	ID=cds1694	1787832-1789268	12.360041	1	0
mRNA	ID=cds4040	4343703-4345349	12.151217	1	1
mRNA	ID=cds2884	3071998-3072711	12	1	2
mRNA	ID=cds626	661602-661865	12	1	2
mRNA	ID=cds1955	2037502-2038506	12	1	1
mRNA	ID=cds1962	2051667-2052983	12	1	0
mRNA	ID=cds3401	3602416-3603012	12	1	0
mRNA	ID=cds596	631612-632700	11.5	1	0
mRNA	ID=cds1951	2033859-2034710	11	1	1
mRNA	ID=cds1030	1114885-1115805	11	1	0
mRNA	ID=cds148	171462-173444	11	1	0
mRNA	ID=cds157	182463-183620	11	1	0
mRNA	ID=cds1734	1826280-1827758	11	1	0
mRNA	ID=cds3160	3360829-3363210	11	1	0
mRNA	ID=cds4273	4591384-4592745	11	1	0
mRNA	ID=cds960	1041253-1043433	11	1	0
mRNA	ID=cds3551	3777077-3777853	10.646447	1	0
mRNA	ID=cds2322	2450378-2453023	10	1	8
mRNA	ID=cds662	702034-702834	10	1	5
mRNA	ID=cds702	745946-746992	10	1	1
mRNA	ID=cds1414	1498597-1499589	10	1	0
mRNA	ID=cds3663	3893295-3894632	10	1	0
mRNA	ID=cds386	409368-410276	10	1	0
mRNA	ID=cds632	667942-668259	10	1	0
mRNA	ID=cds2450	2585617-2588730	9.472027	1	0
mRNA	ID=cds286	306031-308556	9.429198	1	2
mRNA	ID=cds2142	2247739-2248788	9.333333	1	1
mRNA	ID=cds262	282425-284392	9	1	3
mRNA	ID=cds2215	2337589-2338311	9	1	1
mRNA	ID=cds2128	2230900-2231619	9	1	0
mRNA	ID=cds2539	2689678-2693565	9	1	0
mRNA	ID=cds2563	2714776-2715465	9	1	0
mRNA	ID=cds3313	3502957-3504042	9	1	0
mRNA	ID=cds3995	4294459-4295148	9	1	0
mRNA	ID=cds4286	4604692-4605723	9	1	0
mRNA	ID=cds493	529356-530450	9	1	0
mRNA	ID=cds1826	1918247-1919686	8.5	1	1
repeat_region		3229274-3229556	8.059595	1	0.241656
mRNA	ID=cds211	240859-243303	8	1	6
mRNA	ID=cds1519	1619356-1620543	8	1	4
mRNA	ID=cds1200	1271730-1272425	8	1	3
mRNA	ID=cds2376	2509023-2509349	8	1	1
mRNA	ID=cds3715	3954950-3955843	8	1	1
mRNA	ID=cds4120	4423543-4423857	8	1	1
mRNA	ID=cds2936	3129363-3130430	8	1	0.5
mRNA	ID=cds75	85630-87354	8	1	0.073044
mRNA	ID=cds2520	2667054-2668415	8	1	0
mRNA	ID=cds3063	3261708-3263039	8	1	0
mRNA	ID=cds3887	4154873-4156246	7	1	0.666666
mRNA	ID=cds1325	1404003-1404566	7	1	0
mRNA	ID=cds1400	1485259-1486059	7	1	0
mRNA	ID=cds1715	1807404-1808072	7	1	0
mRNA	ID=cds1765	1859726-1859998	7	1	0
mRNA	ID=cds2635	2795233-2795532	7	1	0
mRNA	ID=cds2723	2882575-2885241	7	1	0
mRNA	ID=cds324	344890-345561	7	1	0
mRNA	ID=cds3504	3725940-3727394	7	1	0
mRNA	ID=cds350	376759-377592	7	1	0
mRNA	ID=cds3559	3783283-3784827	7	1	0
mRNA	ID=cds4031	4331305-4331973	7	1	0
mRNA	ID=cds4237	4545765-4546295	7	1	0
mRNA	ID=cds565	593983-594666	7	1	0
mRNA	ID=cds615	651458-653116	7	1	0
mRNA	ID=cds1356	1427073-1430435	6.5	1	0
repeat_region		3295021-3295104	6.322838	1	0.041219
mRNA	ID=cds1226	1304845-1305174	6	1	4
mRNA	ID=cds3052	3250326-3250691	6	1	2
mRNA	ID=cds3961	4257260-4257469	6	1	2
mRNA	ID=cds4054	4360756-4361331	6	1	2
mRNA	ID=cds1220	1297821-1298468	6	1	1
mRNA	ID=cds2222	2347957-2349033	6	1	1
mRNA	ID=cds3463	3677442-3678371	6	1	1
mRNA	ID=cds3835	4096669-4097517	6	1	0
mRNA	ID=cds4258	4568185-4569597	6	1	0
mRNA	ID=cds3352	3544581-3545897	5.469668	1	0
mRNA	ID=cds2286	2417863-2418507	5.353553	1	0
mRNA	ID=cds1899	1987705-1988916	5	1	2
mRNA	ID=cds769	821721-822962	5	1	2
mRNA	ID=cds1397	1478933-1480225	5	1	0
mRNA	ID=cds1907	1995835-1996503	5	1	0
mRNA	ID=cds1966	2057988-2058938	5	1	0
mRNA	ID=cds2171	2280962-2281969	5	1	0
mRNA	ID=cds2765	2935460-2936908	5	1	0

mRNA	ID=cds612	648805-649713	5	1	0
mRNA	ID=cds1515	1617144-1617578	4	1	1
mRNA	ID=cds2000	2087235-2087489	4	1	1
mRNA	ID=cds4212	4520150-4522117	4	1	1
mRNA	ID=cds1407	1490494-1492134	4	1	0
mRNA	ID=cds1928	2014578-2015951	4	1	0
mRNA	ID=cds259	279651-279959	4	1	0
mRNA	ID=cds2876	3062824-3064302	4	1	0
mRNA	ID=cds2972	3166771-3167253	4	1	0
mRNA	ID=cds3033	3232761-3233897	4	1	0
mRNA	ID=cds3111	3309437-3309706	4	1	0
mRNA	ID=cds3387	3587205-3588050	4	1	0
mRNA	ID=cds353	379293-380066	4	1	0
mRNA	ID=cds3713	3951501-3953351	4	1	0
mRNA	ID=cds3876	4140553-4141632	4	1	0
mRNA	ID=cds4071	4375834-4376967	4	1	0
mRNA	ID=cds4232	4540060-4540656	4	1	0
mRNA	ID=cds605	641311-642549	4	1	0
mRNA	ID=cds68	77621-78799	4	1	0
mRNA	ID=cds742	793996-794145	4	1	0
mRNA	ID=cds1443	1525926-1527962	3.666667	1	0
mRNA	ID=cds1405	1489946-1490095	3	1	5
mRNA	ID=cds3388	3588047-3588934	3	1	2
mRNA	ID=cds2510	2657585-2657908	3	1	1
mRNA	ID=cds3008	3202243-3202611	3	1	1
mRNA	ID=cds1509	1610349-1611275	3	1	0
mRNA	ID=cds1523	1622797-1623315	3	1	0
mRNA	ID=cds1931	2017642-2018106	3	1	0
mRNA	ID=cds2004	2089121-2090425	3	1	0
mRNA	ID=cds2103	2203717-2205996	3	1	0
mRNA	ID=cds3096	3295120-3296160	3	1	0
mRNA	ID=cds326	346081-347667	3	1	0
mRNA	ID=cds4265	4578091-4579485	3	1	0
mRNA	ID=cds4271	4587152-4589302	3	1	0
mRNA	ID=cds855	914575-915270	3	1	0
mRNA	ID=cds961	1043453-1043899	3	1	0
mRNA	ID=cds3802	4059188-4060168	2.416666	1	0
mRNA	ID=cds2895	3084209-3084424	2	1	1
mRNA	ID=cds4131	4432136-4432516	2	1	1
mRNA	ID=cds733	784160-784540	2	1	1
mRNA	ID=cds1174	1243951-1244205	2	1	0
mRNA	ID=cds1494	1590200-1590466	2	1	0
mRNA	ID=cds2900	3089156-3089887	2	1	0
mRNA	ID=cds3029	3226910-3229261	2	1	0
mRNA	ID=cds312	331595-332683	2	1	0
mRNA	ID=cds3135	3334985-3335278	2	1	0
mRNA	ID=cds313	332725-333657	2	1	0
mRNA	ID=cds3298	3488288-3488851	2	1	0
mRNA	ID=cds3331	3519455-3519994	2	1	0
mRNA	ID=cds3507	3730224-3731765	2	1	0
mRNA	ID=cds3947	4238802-4240277	2	1	0
mRNA	ID=cds3028	3225823-3226893	1.103515	1	0
mRNA	ID=cds2032	2119633-2121003	1	1	2
mRNA	ID=cds1203	1274402-1275052	1	1	1
mRNA	ID=cds1322	1399834-1401279	1	1	0
mRNA	ID=cds1360	1432982-1433032	1	1	0
mRNA	ID=cds1413	1497493-1498473	1	1	0
mRNA	ID=cds2194	2303130-2304776	1	1	0
mRNA	ID=cds2387	2523149-2523913	1	1	0
mRNA	ID=cds2918	3103736-3104992	1	1	0
mRNA	ID=cds2951	3144472-3144759	1	1	0
mRNA	ID=cds3523	3748109-3748804	1	1	0
mRNA	ID=cds35	36271-37824	1	1	0
mRNA	ID=cds3827	4087878-4088948	1	1	0
mRNA	ID=cds2046	2134128-2135267	0.353553	1	5
mRNA	ID=cds524	563071-563703	0	1	2
mRNA	ID=cds1301	1379801-1379926	0	1	0
mRNA	ID=cds1483	1577657-1578814	0	1	0
mRNA	ID=cds1490	1586333-1586863	0	1	0
mRNA	ID=cds2358	2488278-2489972	0	1	0
mRNA	ID=cds2440	2572324-2573025	0	1	0
mRNA	ID=cds2467	2604284-2604934	0	1	0
mRNA	ID=cds2811	2989290-2989781	0	1	0
mRNA	ID=cds300	316950-317543	0	1	0
mRNA	ID=cds3309	3499290-3500312	0	1	0
mRNA	ID=cds345	371339-372148	0	1	0
mRNA	ID=cds346	372145-373095	0	1	0
mRNA	ID=cds3528	3750986-3752122	0	1	0
mRNA	ID=cds4001	4302635-4304620	0	1	0
mRNA	ID=cds507	545904-547571	0	1	0
mRNA	ID=cds537	571689-572144	0	1	0
mRNA	ID=cds609	645854-646732	0	1	0
mgeRNA	gbkey=mobile_element	2168195-2169452	19.864079	0.6	0.4
mgeRNA	gbkey=mobile_element	1093468-1094725	17.864079	0.6	0.4
mgeRNA	gbkey=mobile_element	390933-392190	17.864079	0.6	0.4
mgeRNA	gbkey=mobile_element	566000-567257	17.864079	0.6	0.4
mgeRNA	gbkey=mobile_element	314450-315707	17.543684	0.6	0.4
repeat_region		4323825-4324407	143.569814	0.58824	2.09473
other ncRNA		1269616-1269683	1573.05183	0.5	1.19696
mRNA	ID=cds956	1036963-1038507	146	0.5	6
mRNA	ID=cds2837	3019338-3022208	81.132658	0.5	2
mRNA	ID=cds1090	1169741-1173187	77.333333	0.5	0
mRNA	ID=cds2779	2950483-2954025	57	0.5	0
mRNA	ID=cds3750	3996006-3998168	51.853553	0.5	4
mRNA	ID=cds2397	2533856-2534365	44	0.5	0
mRNA	ID=cds3422	3623702-3624826	34	0.5	0
mRNA	ID=cds2772	2941359-2942564	28.5	0.5	3
mRNA	ID=cds3727	3969283-3970545	22	0.5	0
mRNA	ID=cds807	864352-865587	20	0.5	1.033652
mRNA	ID=cds2169	2278654-2280414	19.5	0.5	0
mRNA	ID=cds1675	1763653-1766709	19	0.5	0
mRNA	ID=cds3915	4194926-4195699	18	0.5	3
mRNA	ID=cds4253	4561945-4562712	16	0.5	0
mRNA	ID=cds3554	3779764-3780585	14.6331	0.5	2
mRNA	ID=cds2311	2442225-2442773	11	0.5	0
mgeRNA	gbkey=mobile_element	278387-279154	10.199277	0.5	1.166668
mgeRNA	gbkey=mobile_element	289858-290625	10.199277	0.5	1.166668
mRNA	ID=cds487	522485-526765	9.166667	0.5	1
mRNA	ID=cds1970	2062503-2063246	9	0.5	0
repeat_region		1550095-1550191	8.940509	0.5	0.547374
repeat_region		1550273-1550369	8.940509	0.5	0.432377
mRNA	ID=cds256	278402-278905	8.704994	0.5	1.000001
mRNA	ID=cds268	289873-290376	8.704994	0.5	1.000001
mRNA	ID=cds825	885354-886562	8	0.5	1
mRNA	ID=cds592	627774-628520	7	0.5	0
mRNA	ID=cds331	354146-355405	6	0.5	0
mRNA	ID=cds764	818271-818516	5	0.5	0
mRNA	ID=cds257	278824-279099	4.923364	0.5	0.166667
mRNA	ID=cds269	290295-290570	4.923364	0.5	0.166667
mRNA	ID=cds1892	1983163-1984152	4	0.5	0
mRNA	ID=cds318	337549-338967	3.062517	0.5	0
mRNA	ID=cds234	262374-262436	3	0.5	0
mRNA	ID=cds3019	3213749-3214513	3	0.5	0
mRNA	ID=cds1793	1883869-1884834	2	0.5	0
mRNA	ID=cds317	336002-337549	1	0.5	1
mRNA	ID=cds1710	1803349-1804107	1	0.5	0
mRNA	ID=cds3082	3281165-3282025	1	0.5	0
mRNA	ID=cds347	373092-374105	1	0.5	0
mRNA	ID=cds566	594823-596196	1	0.5	0
mRNA	ID=cds339	362455-365529	0.5	0.5	0
mRNA	ID=cds236	262914-263231	0	0.5	0
mRNA	ID=cds2721	2880177-2880659	0	0.5	0
mRNA	ID=cds3402	3603002-3603271	0	0.5	0
mRNA	ID=cds1006	1093498-1094364	11.8	0.4	0
mRNA	ID=cds2074	2168556-2169422	11.8	0.4	0
mRNA	ID=cds297	314811-315677	11.8	0.4	0
mRNA	ID=cds365	390963-391829	11.8	0.4	0
mRNA	ID=cds531	566361-567227	11.8	0.4	0

mRNA	ID=cds3810	4069796-4070674	2.343944	0.353553	1
other ncRNA		1286289-1286459	6.506091	0.336855	2.992067
mgeRNA	gbkey=mobile_element	1465934-1467264	9.169024	0.333334	1.54762
mgeRNA	gbkey=mobile_element	2066965-2068295	9.169024	0.333334	1.54762
mgeRNA	gbkey=mobile_element	2994383-2995713	9.169024	0.333334	1.54762
mgeRNA	gbkey=mobile_element	3184118-3185448	9.169024	0.333334	1.54762
mgeRNA	gbkey=mobile_element	380484-381814	9.169024	0.333334	1.54762
mgeRNA	gbkey=mobile_element	4496204-4497534	9.169024	0.333334	1.54762
mgeRNA	gbkey=mobile_element	2512295-2513639	57.33328	0.333333	1.999998
mgeRNA	gbkey=mobile_element	15387-16731	53.33328	0.333333	1.999998
mgeRNA	gbkey=mobile_element	607230-608574	53.33328	0.333333	1.999998
mRNA	ID=cds14	15445-16557	45.999954	0.333333	1.999998
mRNA	ID=cds2379	2512353-2513465	45.999954	0.333333	1.999998
mRNA	ID=cds577	607288-608400	45.999954	0.333333	1.999998
mRNA	ID=cds865	926697-928418	30	0.333333	1
mRNA	ID=cds745	795777-796835	24	0.333333	0
mRNA	ID=cds2674	2833195-2835447	13	0.333333	0
mgeRNA	gbkey=mobile_element	269765-270985	8.999994	0.333333	0.666666
mRNA	ID=cds249	269827-270978	7.999994	0.333333	0.666666
mRNA	ID=cds2409	2544695-2546119	7	0.333333	1
mgeRNA	gbkey=mobile_element	1467320-1468540	6.999994	0.333333	0.666666
mgeRNA	gbkey=mobile_element	4505482-4506702	6.999994	0.333333	0.666666
mRNA	ID=cds1391	1467382-1468533	6.999994	0.333333	0.666666
mRNA	ID=cds4198	4505489-4506640	6.999994	0.333333	0.666666
mRNA	ID=cds1891	1981579-1983093	6	0.333333	0
mRNA	ID=cds579	609477-611717	5	0.333333	0
tRNA	product=tRNA-Ile	2783784-2783859	0.447649	0.288675	3.616764
repeat_region		374154-374589	85.954303	0.281412	5.142701
repeat_region		4025345-4025625	56.757858	0.260506	4.205949
repeat_region		4612280-4612667	120.506192	0.260403	12.369104
other ncRNA		3193121-3193262	415.815298	0.226455	9.610626
mRNA	ID=cds1007	1094361-1094660	5.6	0.2	0.4
mRNA	ID=cds2073	2168260-2168559	5.6	0.2	0.4
mRNA	ID=cds296	314515-314814	5.6	0.2	0.4
mRNA	ID=cds366	391826-392125	5.6	0.2	0.4
mRNA	ID=cds530	566065-566364	5.6	0.2	0.4
repeat_region		2345182-2345384	41.5033	0.191844	0.463738
mRNA	ID=cds1732	1823979-1824947	3.030415	0.179878	0.086422
mRNA	ID=cds1390	1466808-1467173	4.000008	0.166667	0.500001
mRNA	ID=cds1978	2067839-2068204	4.000008	0.166667	0.500001
mRNA	ID=cds2819	2995257-2995622	4.000008	0.166667	0.500001
mRNA	ID=cds2993	3184209-3184574	4.000008	0.166667	0.500001
mRNA	ID=cds355	380575-380940	4.000008	0.166667	0.500001
mRNA	ID=cds4187	4496295-4496660	4.000008	0.166667	0.500001
mRNA	ID=cds1389	1465945-1466850	3.83568	0.166667	0.714285
mRNA	ID=cds1977	2066976-2067881	3.83568	0.166667	0.714285
mRNA	ID=cds2818	2994394-2995299	3.83568	0.166667	0.714285
mRNA	ID=cds2994	3184532-3185437	3.83568	0.166667	0.714285
mRNA	ID=cds356	380898-381803	3.83568	0.166667	0.714285
mRNA	ID=cds4188	4496618-4497523	3.83568	0.166667	0.714285
repeat_region		410302-410492	33.054576	0.145625	2.323876
repeat_region		805027-805204	16.720439	0.130253	0.150692
repeat_region		4061542-4061616	1.28822	0.130253	0.047004
mRNA	ID=cds220	250072-250827	6.039055	0.129099	0
repeat_region		2338335-2338416	13.077672	0.117648	0.218946
mRNA	ID=cds3537	3765244-3766188	2.766666	0.116473	0
mgeRNA	gbkey=mobile_element	2099773-2100967	29.494013	0.1	0.511111
mgeRNA	gbkey=mobile_element	573814-575008	24.44886	0.1	0.511111
mgeRNA	gbkey=mobile_element	3650059-3651253	24.436682	0.1	0.511111
mgeRNA	gbkey=mobile_element	3128168-3129362	24.182011	0.1	0.511111
mgeRNA	gbkey=mobile_element	687074-688268	22.850073	0.1	0.511111
mgeRNA	gbkey=mobile_element	1394068-1395262	22.25936	0.1	0.511111
mgeRNA	gbkey=mobile_element	3363578-3364772	22.25936	0.1	0.511111
mgeRNA	gbkey=mobile_element	2286941-2288135	22.146937	0.1	0.511111
mgeRNA	gbkey=mobile_element	273179-274373	22.146937	0.1	0.511111
mRNA	ID=cds2015	2099919-2100935	21.806987	0.1	0.411111
mRNA	ID=cds3442	3650205-3651221	21.602717	0.1	0.411111
mRNA	ID=cds2935	3128200-3129216	21.589887	0.1	0.411111
mRNA	ID=cds651	687220-688236	21.513797	0.1	0.411111
mRNA	ID=cds544	573960-574976	21.500366	0.1	0.411111
mRNA	ID=cds1316	1394100-1395116	21.472885	0.1	0.411111
mRNA	ID=cds3162	3363724-3364740	21.472885	0.1	0.411111
mRNA	ID=cds2175	2287087-2288103	21.463222	0.1	0.411111
mRNA	ID=cds251	273325-274341	21.463222	0.1	0.411111
mgeRNA	gbkey=mobile_element	2064183-2065377	19.576462	0.1	0.4
mRNA	ID=cds1973	2064329-2065345	19.003858	0.1	0.3
mRNA	ID=cds2195	2304994-2306637	4.119305	0.091608	0.28354
mRNA	ID=cds4204	4511429-4512331	6.042791	0.083152	0
repeat_region		2591000-2591082	23.098482	0.071429	0
repeat_region		3502808-3502843	13.885896	0.071429	0
repeat_region		3752883-3752964	5.385483	0.071429	0
repeat_region		2314856-2315052	366.522025	0.058824	0.161823
repeat_region		25710-25795	131.744052	0.058824	0.052492
repeat_region		836762-836884	89.217717	0.058824	0.218946
repeat_region		4283325-4283401	41.015095	0.058824	0.218946
repeat_region		3040343-3040497	40.44829	0.058824	0.208367
repeat_region		72136-72220	14.634189	0.058824	0.089451
repeat_region		3232660-3232745	8.17741	0.058824	0.047004
repeat_region		338981-339338	21.14187	0.046116	2.798077
mRNA	ID=cds3445	3653278-3653925	28	0.02657	0.021433
mRNA	ID=cds3421	3622401-3623537	2.083333	0.022125	0
mRNA	ID=cds1446	1529840-1530976	1.583333	0.022125	0
mRNA	ID=cds692	736048-737184	1.083333	0.022125	0
repeat_region		2234556-2234705	212.162774	0.015372	1.545906
repeat_region		631338-631371	114.66813	0.015372	0.566736
repeat_region		376559-376745	106.069585	0.015372	0.519553
repeat_region		4407176-4407273	98.605219	0.015372	1.770023
repeat_region		2943918-2944014	94.560436	0.015372	2.40553
repeat_region		3674144-3674304	86.847954	0.015372	0.414787
repeat_region		4455208-4455291	76.97188	0.015372	0.797787
repeat_region		4233444-4233520	74.986932	0.015372	0.308515
mRNA	ID=cds4294	4611504-4612283	64.626115	0.015372	2.04079
repeat_region		4216459-4216590	48.387376	0.015372	3.941026
repeat_region		2289175-2289373	34.02612	0.015372	3.847795
repeat_region		814808-814904	27.508914	0.015372	2.200607
repeat_region		2682139-2682236	26.931788	0.015372	0.238993
repeat_region		3637232-3637329	24.606976	0.015372	1.89175
repeat_region		706993-707157	23.085141	0.015372	0.813365
repeat_region		2547488-2547585	22.899563	0.015372	0.039728
repeat_region		1814209-1814397	19.675663	0.015372	4.141268
repeat_region		356708-356786	17.092027	0.015372	4.656682
repeat_region		356908-356986	17.092027	0.015372	4.656682
repeat_region		2840445-2840523	14.457237	0.015372	1.468527
repeat_region		3148576-3148830	12.274472	0.015372	4.02406
repeat_region		4125975-4126071	11.910007	0.015372	5.136727
repeat_region		698648-698745	11.909002	0.015372	0.714132
repeat_region		714487-714584	11.781462	0.015372	0.251106
repeat_region		1952453-1952550	11.450902	0.015372	0.712911
repeat_region		3875609-3875706	10.692875	0.015372	0.995341
repeat_region		609340-609437	9.54925	0.015372	0.205677
repeat_region		4552528-4552561	8.781661	0.015372	0.716106
repeat_region		3068025-3068122	7.525816	0.015372	0.634868
repeat_region		4078043-4078140	4.603224	0.015372	1.151451
repeat_region		3510533-3510622	3.994575	0.015372	0.401638
repeat_region		2712305-2712402	1.936071	0.015372	0.113195
repeat_region		3214623-3214700	1.687952	0.015372	0.35015
repeat_region		2131456-2131489	0.566066	0.015372	0.873501
mRNA	ID=cds953	1034993-1035580	168.038471	0.011354	6
mRNA	ID=cds3520	3745107-3746603	7	0.010406	0
other ncRNA		3348599-3348719	1196	0	47
mRNA	ID=cds4236	4543119-4545755	917	0	0
mRNA	ID=cds2421	2555340-2556701	857	0	2
mRNA	ID=cds2287	2418643-2419290	829	0	0
mRNA	ID=cds4233	4541138-4541686	654	0	1
mRNA	ID=cds862	922487-924763	602.582971	0	4
other ncRNA		1407153-1407274	584.959059	0	5
other ncRNA		887199-887277	574	0	7
mRNA	ID=cds710	755130-756896	431	0	3

mRNA	ID=cds1666	1755745-1756749	377	0	1
other ncRNA		2940718-2940923	373	0	73
repeat_region		1555087-1555105	369	0	0
mRNA	ID=cds2554	2705820-2706776	342	0	3
mRNA	ID=cds3929	4213501-4215102	341	0	2
mRNA	ID=cds4059	4366687-4367163	341	0	1
mRNA	ID=cds712	757929-760730	330.631367	0	7
mRNA	ID=cds3684	3917893-3918426	317	0	0
mRNA	ID=cds476	511800-513092	314.790835	0	6
other ncRNA		606957-607015	270	0	2
mRNA	ID=cds1643	1731778-1732125	270	0	0
mRNA	ID=cds2498	2639853-2640866	266.450339	0	3
mRNA	ID=cds50	52427-53416	266	0	0
mRNA	ID=cds2308	2438407-2439627	248	0	2
mRNA	ID=cds995	1074143-1078105	241.190444	0	0
mRNA	ID=cds81	91413-93179	241	0	6
mRNA	ID=cds3194	3398066-3399109	238	0	0
repeat_region		500668-500751	237.458649	0	19
mRNA	ID=cds2858	3043180-3043923	234	0	0
mRNA	ID=cds2514	2660605-2661345	232.163636	0	2
repeat_region		2660331-2660596	228.999773	0	0.9297
mRNA	ID=cds3777	4023011-4024504	224.224979	0	1
mgeRNA	gbkey=misc_feature	2464407-2474621	219.5	0	2.1066
other ncRNA		2023251-2023337	218.517238	0	63
mRNA	ID=cds3722	3963784-3964113	215	0	0
mRNA	ID=cds898	970975-971868	215	0	0
mRNA	ID=cds3251	3447204-3447905	214	0	4
mRNA	ID=cds170	196546-197898	214	0	1
mRNA	ID=cds4092	4398695-4399975	213.776393	0	2
mRNA	ID=cds2077	2172304-2172588	211	0	0
mRNA	ID=cds1319	1396798-1397550	209.915779	0	2
mRNA	ID=cds3220	3429442-3429984	208	0	2
mRNA	ID=cds3295	3484142-3484774	206	0	10
mRNA	ID=cds2945	3138333-3138821	198	0	0
mRNA	ID=cds2499	2641151-2642305	195.114184	0	1
mRNA	ID=cds3248	3446336-3446590	188	0	0
mRNA	ID=cds722	773532-773825	188	0	0
mRNA	ID=cds2281	2411492-2412694	186.80132	0	4
mRNA	ID=cds3931	4216619-4218355	183.843004	0	13
mRNA	ID=cds178	205126-208608	181.778634	0	1
other ncRNA		2698542-2698618	179	0	2
mRNA	ID=cds2647	2805154-2806146	176.514952	0	0
mRNA	ID=cds3683	3916339-3917880	176.376393	0	2
mRNA	ID=cds3193	3396897-3398000	174	0	0.5
mRNA	ID=cds3679	3911853-3913223	173	0	2
mRNA	ID=cds3905	4179268-4183296	172	0	1
other ncRNA		2151668-2151803	169.296725	0	3.278019
mRNA	ID=cds2271	2400077-2401867	169	0	8
mRNA	ID=cds3957	4252066-4254489	168.46599	0	6
mRNA	ID=cds713	760745-761962	166.92713	0	3
mRNA	ID=cds21	20815-21078	162	0	1
mRNA	ID=cds3681	3914016-3915398	160.745412	0	2
mRNA	ID=cds2703	2864581-2865573	158	0	0
mRNA	ID=cds950	1031362-1032480	152.053835	0	8.121584
mRNA	ID=cds1771	1864932-1866866	149	0	3
mRNA	ID=cds3555	3780665-3781684	149	0	0
mRNA	ID=cds421	447270-447884	148	0	1
mRNA	ID=cds2078	2172619-2173071	148	0	0
mRNA	ID=cds3933	4220827-4221651	148	0	0
repeat_region		1027589-1027612	147	0	0
mRNA	ID=cds890	961218-962891	146	0	11
repeat_region		2391153-2391188	144	0	0
mRNA	ID=cds3340	3530840-3532462	141	0	2
mRNA	ID=cds1583	1667723-1668976	138	0	1
mRNA	ID=cds3935	4225754-4227385	137.954586	0	0
mRNA	ID=cds1307	1386329-1386835	136.827356	0	11
repeat_region		2615990-2616026	136.790783	0	0
mRNA	ID=cds1026	1110086-1112629	136	0	4
mRNA	ID=cds3233	3439077-3439697	136	0	1
mRNA	ID=cds3479	3700888-3701871	135.5	0	0
mRNA	ID=cds3110	3307055-3309190	135	0	3
mRNA	ID=cds882	950495-952777	134.268616	0	2
mRNA	ID=cds3355	3551107-3553812	132.851335	0	5
mRNA	ID=cds857	917351-918343	132	0	2
mRNA	ID=cds594	629117-631222	132	0	0.5
other ncRNA		2165138-2165224	131	0	2
mRNA	ID=cds1257	1333855-1336530	130.333333	0	4
mRNA	ID=cds2882	3069481-3070644	130.333333	0	1
mRNA	ID=cds3678	3909862-3911691	130.142675	0	2
mRNA	ID=cds4317	4637613-4638329	130	0	4
mRNA	ID=cds4249	4558020-4558703	130	0	1
mRNA	ID=cds422	447874-449865	129.324536	0	0
mRNA	ID=cds3847	4108763-4109530	129	0	1
tRNA	pseudo=true	2474606-2474620	129	0	0.1066
mRNA	ID=cds25	25207-25701	129	0	0
repeat_region		4227399-4227468	126.678807	0	0.008913
mRNA	ID=cds1109	1191890-1192996	126	0	7
mRNA	ID=cds3622	3851945-3853129	125.869442	0	2
mRNA	ID=cds3685	3918441-3918911	123.75	0	0
mRNA	ID=cds2048	2137783-2139636	123	0	0
repeat_region		4413981-4414014	122.856378	0	1
mRNA	ID=cds1064	1146017-1146538	122	0	1
mRNA	ID=cds1222	1300923-1301843	121.780377	0	0
mRNA	ID=cds4140	4442135-4445914	119	0	4
mRNA	ID=cds2788	2964210-2966456	119	0	3
mRNA	ID=cds3894	4163451-4164308	119	0	1
mRNA	ID=cds3815	4074169-4075041	118	0	1
mRNA	ID=cds17	17489-18655	117	0	1
mRNA	ID=cds3852	4111749-4112495	117	0	1
mRNA	ID=cds49	51609-52430	117	0	0
mRNA	ID=cds412	439426-440325	115	0	1
mRNA	ID=cds1168	1236794-1238092	113	0	6
mRNA	ID=cds3005	3199229-3199849	113	0	3
mRNA	ID=cds872	938651-939943	112.416667	0	0
mRNA	ID=cds2659	2817403-2820033	112.353553	0	0
mRNA	ID=cds3747	3993606-3994313	112	0	0
mRNA	ID=cds2200	2311510-2314182	111.333333	0	7
mRNA	ID=cds4247	4556377-4557549	111.157337	0	1
mRNA	ID=cds3914	4194355-4194831	110.861325	0	3
mRNA	ID=cds3584	3809461-3809697	110.434525	0	0
mRNA	ID=cds3423	3624826-3627561	110	0	1
mRNA	ID=cds2202	2315049-2317898	108.358792	0	1
mRNA	ID=cds4098	4404677-4407118	108	0	3
mRNA	ID=cds460	491316-493247	108	0	2
mRNA	ID=cds3400	3600773-3602266	108	0	1
mRNA	ID=cds2445	2577658-2579661	107	0	4
mRNA	ID=cds3597	3820423-3822531	106	0	1
mRNA	ID=cds475	510865-511797	104.790835	0	3
mRNA	ID=cds3373	3575754-3576749	104.5	0	3
mRNA	ID=cds3245	3444921-3445460	103	0	7
mRNA	ID=cds955	1035972-1036829	103	0	1
repeat_region		3544244-3544279	103	0	0
mRNA	ID=cds3851	4111316-4111744	103	0	0
mRNA	ID=cds2160	2268001-2268567	102	0	0
mRNA	ID=cds3656	3886458-3886532	102	0	0
mRNA	ID=cds3989	4287831-4288502	101	0	0
mRNA	ID=cds3231	3437638-3438021	100	0	0
mRNA	ID=cds3342	3533887-3534606	98	0	12
mRNA	ID=cds3451	3658437-3661550	97.25	0	3
repeat_region		4108733-4108751	97	0	0
mRNA	ID=cds1067	1147982-1148935	96	0	3
mRNA	ID=cds171	197928-200360	96	0	2
mRNA	ID=cds90	103155-103985	96	0	1
mRNA	ID=cds3296	3484813-3486915	95.364343	0	1
mRNA	ID=cds1082	1162483-1163307	95	0	0
mRNA	ID=cds1071	1151162-1152403	94	0	2
mRNA	ID=cds4144	4447145-4447675	94	0	2
mRNA	ID=cds3883	4148470-4151121	94	0	1

mRNA	ID=cds24	22391-25207	93.467707	0	1
mRNA	ID=cds3647	3875728-3878142	93.223607	0	1
mRNA	ID=cds428	455901-456524	93	0	3
mRNA	ID=cds1700	1793277-1793576	93	0	0
mRNA	ID=cds2396	2532088-2533815	92	0	4
repeat_region		1145151-1145225	90.400299	0	0.012075
mRNA	ID=cds3893	4161662-4163506	90	0	0
mRNA	ID=cds3648	3878171-3879244	89	0	1
mRNA	ID=cds57	63429-65780	88	0	3
mRNA	ID=cds173	200971-201996	88	0	1
mRNA	ID=cds1993	2080780-2082207	88	0	1
mRNA	ID=cds3368	3569339-3571525	87.5	0	2
mRNA	ID=cds1858	1946774-1948546	87.114882	0	4
mRNA	ID=cds3975	4269072-4271894	87	0	4
mRNA	ID=cds1579	1663339-1664595	87	0	0
mRNA	ID=cds3693	3925178-3926170	86	0	9
mRNA	ID=cds1184	1258014-1258292	86	0	2
mRNA	ID=cds3189	3390480-3394280	86	0	1
mgeRNA	gbkey=misc_feature	2556721-2563483	85.021119	0	3
other ncRNA		1489467-1489530	85	0	6
mRNA	ID=cds2693	2852360-2854438	85	0	2
mRNA	ID=cds3234	3439731-3440120	85	0	2
other ncRNA		2151333-2151475	84.502892	0	1
mRNA	ID=cds3100	3297988-3298290	84	0	1
other ncRNA		1620841-1620938	84	0	0
mRNA	ID=cds1754	1846861-1848717	84	0	0
mRNA	ID=cds77	88028-89032	84	0	0
mRNA	ID=cds4048	4351223-4352740	83.697195	0	2
mRNA	ID=cds873	940182-942626	83.58803	0	0
mRNA	ID=cds3101	3298277-3298780	82	0	1
mRNA	ID=cds3446	3653989-3654315	82	0	0
mRNA	ID=cds2834	3014082-3017180	81	0	2
mRNA	ID=cds2109	2210981-2212666	81	0	1
repeat_region		2943016-2943049	81	0	0
mRNA	ID=cds2173	2282398-2284158	80.740563	0	2
repeat_region		3623612-3623693	79.902609	0	0
mRNA	ID=cds3940	4231781-4233430	79.788676	0	2.5
mRNA	ID=cds200	232597-233955	79	0	0
mRNA	ID=cds3699	3933311-3934276	79	0	0
repeat_region		1074108-1074135	78	0	0
repeat_region		4626718-4626867	77.897098	0	4.821078
mRNA	ID=cds3243	3444175-3444567	77	0	2
mRNA	ID=cds2981	3174028-3174855	76.666667	0	0
mRNA	ID=cds602	638168-638731	76.591752	0	0
repeat_region		444424-444510	76.572521	0	1.02406
mRNA	ID=cds1671	1760546-1762033	76	0	3
mRNA	ID=cds2704	2865636-2866775	76	0	3
mRNA	ID=cds429	456650-457924	76	0	2
mRNA	ID=cds846	903816-904139	76	0	1
mRNA	ID=cds1253	1329072-1331669	76	0	0
mRNA	ID=cds1223	1301858-1302766	75.766146	0	0
mRNA	ID=cds477	513217-513624	75.640789	0	13
mRNA	ID=cds3595	3819451-3820074	75	0	8
mRNA	ID=cds4066	4373722-4374288	75	0	6
mRNA	ID=cds3813	4072692-4073477	75	0	1
mRNA	ID=cds1069	1149893-1150627	75	0	0
mRNA	ID=cds164	190857-191708	75	0	0
mRNA	ID=cds2574	2734168-2734905	74.570802	0	3
mRNA	ID=cds1399	1481085-1484987	74.5	0	3.666666
mRNA	ID=cds909	986808-988208	74.211324	0	2
mRNA	ID=cds3889	4157413-4158813	74	0	6
mRNA	ID=cds933	1015175-1015693	74	0	4
mRNA	ID=cds2023	2108162-2109043	74	0	0
mRNA	ID=cds667	709423-709869	74	0	0
mRNA	ID=cds3645	3874163-3874975	73.288675	0	0
mRNA	ID=cds84	96002-97084	73	0	1
mRNA	ID=cds3432	3638885-3640354	73	0	0
mRNA	ID=cds3498	3720351-3722420	72.5	0	1.666666
mRNA	ID=cds121	142008-142670	72	0	7
mRNA	ID=cds1106	1188999-1189670	72	0	3
mRNA	ID=cds1980	2069563-2072682	72	0	3
mRNA	ID=cds2868	3054912-3054971	72	0	3
mRNA	ID=cds1508	1609990-1610349	72	0	1
mRNA	ID=cds660	699597-700817	72	0	1
mRNA	ID=cds1704	1797417-1797773	72	0	0
mRNA	ID=cds1944	2026473-2027390	72	0	0
mRNA	ID=cds2201	2314199-2318849	72	0	0
mRNA	ID=cds2588	2744456-2745817	72	0	0
mRNA	ID=cds377	402927-404042	72	0	0
mRNA	ID=cds2591	2748137-2748730	71.82257	0	0
mRNA	ID=cds957	1038519-1039655	71	0	3
mRNA	ID=cds3327	3516565-3517086	71	0	2
mRNA	ID=cds1272	1349431-1349784	71	0	1
mRNA	ID=cds51	53416-54702	71	0	1
mRNA	ID=cds1243	1319408-1320970	70.870112	0	4
mRNA	ID=cds4091	4398311-4398619	70.646447	0	4
mRNA	ID=cds4276	4595173-4597464	70	0	5
mRNA	ID=cds430	458112-460466	70	0	5
mRNA	ID=cds3254	3448565-3449386	70	0	2
mRNA	ID=cds708	754400-754789	70	0	0
mRNA	ID=cds3900	4175766-4176311	69.5	0	0
mRNA	ID=cds95	108279-110984	69.366167	0	1
mRNA	ID=cds2859	3044190-3047063	69.28242	0	0
repeat_region		1355794-1355823	69	0	2
mRNA	ID=cds2691	2850158-2851279	69	0	1
mRNA	ID=cds2051	2141290-2144607	69	0	0.5
mRNA	ID=cds2494	2635496-2636674	69	0	0
mRNA	ID=cds911	989845-992457	69	0	0
mRNA	ID=cds1363	1435284-1438808	68	0	8
mRNA	ID=cds1169	1238102-1239172	68	0	7
mRNA	ID=cds409	435813-436331	68	0	2
mRNA	ID=cds4073	4377400-4377795	68	0	1
mRNA	ID=cds952	1034289-1034996	68	0	0
mRNA	ID=cds3001	3193163-3193222	67.506619	0	4.5
mRNA	ID=cds3723	3964440-3965699	67.4	0	1
mRNA	ID=cds3124	3325812-3326105	67	0	19
mRNA	ID=cds408	434858-435835	67	0	2
mRNA	ID=cds2	2801-3733	67	0	1
mRNA	ID=cds1855	1944879-1945400	67	0	0
mRNA	ID=cds1901	1990293-1990841	66.891988	0	0
mRNA	ID=cds2517	2663457-2664737	66	0	5
mRNA	ID=cds92	105305-106456	66	0	4
mRNA	ID=cds2261	2388070-2389527	66	0	1
mRNA	ID=cds3092	3291422-3293458	65	0	4
mRNA	ID=cds79	90094-91035	65	0	3
mRNA	ID=cds866	928419-930185	65	0	2
mRNA	ID=cds4028	4328525-4330027	65	0	1
repeat_region		2407518-2407536	65	0	0
mRNA	ID=cds1434	1515906-1516355	65	0	0
mRNA	ID=cds1847	1938337-1939659	65	0	0
mRNA	ID=cds2299	2429044-2429706	64	0	0
mRNA	ID=cds2282	2412769-2414913	63.908248	0	2
mRNA	ID=cds2262	2389534-2391063	63.105794	0	1
mRNA	ID=cds3324	3513099-3513935	63	0	2
mRNA	ID=cds215	245961-246239	63	0	0
mRNA	ID=cds3223	3431712-3432221	63	0	0
mRNA	ID=cds468	500786-502462	63	0	0
mRNA	ID=cds2168	2277810-2278505	62.75	0	0
mRNA	ID=cds3888	4156513-4157430	62.730725	0	2
mRNA	ID=cds1942	2024347-2026041	62	0	3
mRNA	ID=cds2289	2419730-2420623	62	0	0
mRNA	ID=cds726	775565-776830	62	0	0
mRNA	ID=cds1630	1717900-1718367	61	0	7
mRNA	ID=cds761	816267-817256	61	0	4
mRNA	ID=cds372	398817-399029	61	0	0
mRNA	ID=cds407	434361-434780	61	0	0
mRNA	ID=cds1279	1355826-1357211	60.5	0	2
mRNA	ID=cds1066	1146844-1147914	60	0	1

mRNA	ID=cds2058	2151373-2151432	60	0	1
mRNA	ID=cds1025	1108558-1110093	60	0	0
mRNA	ID=cds2085	2180057-2180803	60	0	0
mRNA	ID=cds2789	2966469-2966999	60	0	0
mRNA	ID=cds2861	3047595-3048689	60	0	0
mRNA	ID=cds638	674241-674723	59.704196	0	3
mRNA	ID=cds1219	1294669-1297344	59.5	0	0
mRNA	ID=cds1598	1682283-1683212	59	0	3
mRNA	ID=cds2513	2659665-2660153	59	0	2
mRNA	ID=cds2594	2751627-2751968	59	0	1
other ncRNA		4188350-4188510	59	0	0
mRNA	ID=cds3384	3584966-3585406	59	0	0
mRNA	ID=cds603	638976-640541	59	0	0
mRNA	ID=cds4174	4479005-4481860	58	0	2
mRNA	ID=cds661	700826-701974	58	0	2
mRNA	ID=cds673	712781-714421	58	0	1
mRNA	ID=cds3969	4263805-4264884	58	0	0
mRNA	ID=cds4061	4368711-4369004	58	0	0
mRNA	ID=cds4093	4400061-4401320	58	0	0
mRNA	ID=cds3510	3734376-3735200	57	0	2
mRNA	ID=cds3680	3913576-3913995	57	0	2
mRNA	ID=cds2508	2655107-2656957	57	0	1
mRNA	ID=cds3129	3330884-3331141	57	0	0
mRNA	ID=cds2747	2911721-2913022	56.6151	0	1
mRNA	ID=cds3109	3306062-3306946	56.5	0	6
mRNA	ID=cds1186	1260151-1261098	56	0	2
mRNA	ID=cds2026	2111458-2112351	56	0	2
mRNA	ID=cds128	147944-148795	56	0	0
mRNA	ID=cds2250	2377370-2378665	56	0	0
mRNA	ID=cds2846	3031679-3033196	56	0	0
mRNA	ID=cds3163	3364948-3365664	56	0	0
mRNA	ID=cds427	454357-455655	56	0	0
mRNA	ID=cds907	983742-984932	56	0	0
repeat_region		2948635-2948648	55.871256	0	0
mRNA	ID=cds1112	1194346-1195596	55.823223	0	0
mRNA	ID=cds2165	2274322-2275911	55.5	0	7
mRNA	ID=cds3649	3879244-3880344	55.381573	0	3
mRNA	ID=cds2167	2276592-2277782	55.120573	0	0
other ncRNA		189712-189847	55	0	4
mRNA	ID=cds3431	3638134-3638568	55	0	1
mRNA	ID=cds2980	3173447-3174028	55	0	0
mRNA	ID=cds423	449887-450834	55	0	0
mRNA	ID=cds3224	3432236-3433183	54.5	0	4
mRNA	ID=cds4175	4481860-4482303	54	0	3
mRNA	ID=cds904	980270-982117	54	0	3
mRNA	ID=cds1575	1658580-1661003	54	0	0
mRNA	ID=cds1696	1790291-1790755	54	0	0
mRNA	ID=cds2979	3171526-3173418	54	0	0
mRNA	ID=cds2985	3177766-3178437	54	0	0
mRNA	ID=cds3653	3882839-3883096	54	0	0
mRNA	ID=cds4255	4563989-4565269	54	0	0
mRNA	ID=cds2263	2391227-2393068	53.853152	0	0
mRNA	ID=cds3450	3657255-3658412	53.718068	0	1
mRNA	ID=cds1277	1353491-1355134	53.646447	0	4
mRNA	ID=cds4057	4363495-4364796	53.583333	0	0
mRNA	ID=cds2758	2926251-2927540	53.5	0	3
mRNA	ID=cds782	836888-837148	53.240867	0	1
mRNA	ID=cds981	1062078-1062998	53	0	3
mRNA	ID=cds1235	1312044-1312682	53	0	2
mRNA	ID=cds893	965844-967592	53	0	2
mRNA	ID=cds2907	3094405-3094695	53	0	0
mRNA	ID=cds3140	3338297-3339274	53	0	0
mRNA	ID=cds901	973542-974864	53	0	0
repeat_region		213632-213668	52.877061	0	0
mRNA	ID=cds3335	3524491-3526626	52.666666	0	3
mRNA	ID=cds2950	3143165-3144283	52.473479	0	0
other ncRNA		4526000-4526089	52	0	20
mRNA	ID=cds3688	3920083-3920463	52	0	2
mRNA	ID=cds1271	1348275-1349063	52	0	1
mRNA	ID=cds3321	3510656-3511660	52	0	0
mRNA	ID=cds2406	2541854-2542645	51.5	0	0
mRNA	ID=cds664	705316-706980	51.375942	0	2
mRNA	ID=cds905	982298-982846	51	0	2
mRNA	ID=cds1845	1935673-1937115	51	0	0
mRNA	ID=cds1975	2066285-2066443	51	0	0
mRNA	ID=cds3634	3864492-3864920	51	0	0
mRNA	ID=cds48	51229-51606	51	0	0
mRNA	ID=cds3899	4175381-4175764	50.873119	0	0
repeat_region		1841748-1841840	50.077819	0	0.02406
mRNA	ID=cds984	1066087-1066314	50	0	4
mRNA	ID=cds3742	3987848-3988789	50	0	3
mRNA	ID=cds3399	3600102-3600770	50	0	2
mRNA	ID=cds4177	4484241-4485341	50	0	2
mRNA	ID=cds2864	3051537-3052862	50	0	1
mRNA	ID=cds91	103982-105244	50	0	1
mRNA	ID=cds927	1007067-1009175	50	0	0
mRNA	ID=cds1155	1224608-1225303	49.829289	0	0
mRNA	ID=cds138	157729-159126	49.697571	0	5
mRNA	ID=cds2130	2232055-2233293	49.483624	0	0
mRNA	ID=cds2443	2574120-2576399	49.176777	0	0
mRNA	ID=cds3902	4176902-4177606	49	0	4
mRNA	ID=cds2273	2402651-2403094	49	0	2
mRNA	ID=cds2383	2517279-2518694	49	0	1
mRNA	ID=cds2423	2558279-2558920	49	0	1
mRNA	ID=cds230	257829-258230	49	0	0
mRNA	ID=cds3599	3823233-3825314	49	0	0
mRNA	ID=cds2269	2398240-2399577	48.516954	0	2
mRNA	ID=cds1998	2085353-2086282	48.127294	0	0
mRNA	ID=cds1580	1664548-1665243	48	0	3
mRNA	ID=cds462	493629-494234	48	0	2
mRNA	ID=cds732	783105-784046	48	0	1
mRNA	ID=cds2134	2237372-2238370	48	0	0
mRNA	ID=cds2476	2614116-2615579	48	0	0
mRNA	ID=cds190	218887-219594	47.806004	0	0
mRNA	ID=cds2300	2429696-2430964	47.788676	0	0
repeat_region		3201166-3201290	47.540337	0	0.595808
mRNA	ID=cds3490	3715333-3716307	47.5	0	2
mRNA	ID=cds948	1029562-1029891	47.41459	0	0
mRNA	ID=cds3397	3597952-3598806	47.191984	0	1
mRNA	ID=cds954	1035577-1035975	47	0	3
mRNA	ID=cds1766	1860040-1860453	47	0	2
mRNA	ID=cds189	217057-218775	47	0	1
mRNA	ID=cds2495	2636685-2637305	47	0	1
mRNA	ID=cds419	446039-446929	47	0	1
mRNA	ID=cds1804	1894956-1896320	47	0	0
mRNA	ID=cds3250	3446781-3447191	47	0	0
mRNA	ID=cds607	643420-644226	47	0	0
mRNA	ID=cds942	1025780-1026238	47	0	0
mRNA	ID=cds3533	3757881-3759272	46.5	0	1
mRNA	ID=cds1108	1191213-1191854	46.247269	0	0
mRNA	ID=cds3247	3445800-3446171	46	0	5
mRNA	ID=cds2559	2710918-2712252	46	0	2
mRNA	ID=cds185	214291-214836	46	0	1
mRNA	ID=cds4080	4384070-4387393	46	0	1
mRNA	ID=cds1242	1317813-1319408	46	0	0
mRNA	ID=cds1797	1888596-1889291	46	0	0
mRNA	ID=cds2098	2192322-2194355	46	0	0
mRNA	ID=cds4007	4310124-4311014	46	0	0
mRNA	ID=cds4248	4557562-4558023	46	0	0
mRNA	ID=cds464	496399-497043	46	0	0
mRNA	ID=cds635	669797-670828	46	0	0
mRNA	ID=cds805	862865-863527	46	0	0
mRNA	ID=cds2959	3151585-3152244	45.711325	0	0
mRNA	ID=cds3398	3599051-3600109	45.58541	0	1
mRNA	ID=cds162	188712-189506	45.5	0	1
mRNA	ID=cds3115	3314061-3315548	45	0	4
mRNA	ID=cds1401	1486256-1487695	45	0	2
mRNA	ID=cds2496	2637323-2638597	45	0	2

mRNA	ID=cds3976	4272148-4272684	45	0	2
mRNA	ID=cds56	60358-63264	45	0	1
mRNA	ID=cds3175	3376245-3376673	45	0	0
mRNA	ID=cds4122	4424131-4424580	45	0	0
mRNA	ID=cds636	670828-671409	45	0	0
mRNA	ID=cds655	691561-692601	45	0	0
repeat_region		254217-254244	44.81239	0	0
mRNA	ID=cds3720	3960768-3962252	44.530332	0	2
mRNA	ID=cds3154	3348711-3351047	44	0	3
mRNA	ID=cds2274	2403725-2404663	44	0	2
mRNA	ID=cds3138	3336488-3337270	44	0	2
mRNA	ID=cds1317	1395389-1395646	44	0	1
mRNA	ID=cds2568	2720749-2722104	44	0	1
mRNA	ID=cds700	744388-745122	44	0	1
repeat_region		1186306-1186329	44	0	0
mRNA	ID=cds2217	2342887-2345172	44	0	0
mRNA	ID=cds2301	2431034-2431948	44	0	0
mRNA	ID=cds2746	2909439-2911673	44	0	0
mRNA	ID=cds3636	3865751-3866083	44	0	0
mRNA	ID=cds1918	2006301-2007506	43.084003	0	0
mRNA	ID=cds451	479558-479932	43	0	3
mRNA	ID=cds3429	3635665-3637164	43	0	1
mRNA	ID=cds3690	3921767-3923656	43	0	1
mRNA	ID=cds4318	4638425-4638565	43	0	1
mRNA	ID=cds1385	1461563-1462513	43	0	0
mRNA	ID=cds3481	3702794-3703813	43	0	0
mRNA	ID=cds4076	4380666-4381643	43	0	0
mRNA	ID=cds610	646707-647258	43	0	0
mRNA	ID=cds83	94650-96008	43	0	0
mRNA	ID=cds2773	2942564-2943007	42.790968	0	0
repeat_region		516593-516618	42.529308	0	0
mRNA	ID=cds883	952832-953689	42.275521	0	0
mRNA	ID=cds1673	1762958-1763146	42	0	3
mRNA	ID=cds3369	3571798-3572901	42	0	2
mRNA	ID=cds1803	1894194-1894772	42	0	1
mRNA	ID=cds3700	3934301-3935191	42	0	1
mRNA	ID=cds1084	1164366-1164908	42	0	0
mRNA	ID=cds1611	1700257-1701258	42	0	0
mRNA	ID=cds1699	1792196-1793176	42	0	0
mRNA	ID=cds2551	2702357-2703331	42	0	0
mRNA	ID=cds2982	3174880-3175302	42	0	0
mRNA	ID=cds3228	3436453-3436671	42	0	0
mRNA	ID=cds3257	3450319-3450948	42	0	0
mRNA	ID=cds3	3734-5020	42	0	0
mRNA	ID=cds4060	4367179-4368435	41.532752	0	2
mRNA	ID=cds2854	3039335-3040315	41.333333	0	3
mRNA	ID=cds432	461139-463010	41	0	3
other ncRNA		4577858-4577934	41	0	2
mRNA	ID=cds2878	3065362-3066102	41	0	2
mRNA	ID=cds3741	3987111-3987851	41	0	2
mRNA	ID=cds809	866743-868614	41	0	1
mRNA	ID=cds85	97087-98403	41	0	1
mRNA	ID=cds1782	1875739-1876764	41	0	0
mRNA	ID=cds3661	3891904-3892653	41	0	0
mRNA	ID=cds47	50380-51222	41	0	0
repeat_region		2230758-2230791	40.470024	0	0.294431
mRNA	ID=cds2901	3089900-3090850	40.347404	0	0
mRNA	ID=cds3141	3339288-3340274	40	0	3
mRNA	ID=cds1258	1336594-1337184	40	0	2
mRNA	ID=cds724	774376-775068	40	0	2
mRNA	ID=cds2865	3052888-3053466	40	0	1
mRNA	ID=cds819	879950-881152	40	0	1
repeat_region		1663160-1663190	40	0	0
repeat_region		3483394-3483415	40	0	0
mRNA	ID=cds1806	1898053-1899609	40	0	0
mRNA	ID=cds2313	2443582-2444406	40	0	0
mRNA	ID=cds2459	2595853-2596887	40	0	0
mRNA	ID=cds2638	2797186-2797635	40	0	0
mRNA	ID=cds3239	3442565-3442744	40	0	0
mRNA	ID=cds3765	4011076-4013337	40	0	0
mRNA	ID=cds3868	4127858-4130290	40	0	0
mRNA	ID=cds627	661975-663186	40	0	0
mRNA	ID=cds86	98403-99647	40	0	0
repeat_region		4106594-4106691	39.605456	0	0.353553
mRNA	ID=cds2315	2445530-2446462	39.467248	0	3
mRNA	ID=cds2552	2703347-2705146	39.235702	0	0
repeat_region		4468404-4468491	39.073119	0	0.66535
mRNA	ID=cds1846	1937246-1938217	39	0	2
mRNA	ID=cds1664	1753722-1755134	39	0	1
mRNA	ID=cds2266	2393930-2394472	39	0	1
mRNA	ID=cds771	823853-824263	39	0	1
mRNA	ID=cds1309	1387894-1388622	39	0	0
mRNA	ID=cds167	193521-194717	39	0	0
mRNA	ID=cds2131	2233287-2234522	39	0	0
mRNA	ID=cds2151	2257741-2259432	39	0	0
mRNA	ID=cds2270	2399574-2400074	39	0	0
mRNA	ID=cds2328	2459328-2460668	39	0	0
mRNA	ID=cds2553	2705344-2705823	39	0	0
mRNA	ID=cds18	18715-19620	38.752203	0	0
repeat_region		4293858-4294457	38.743489	0	1.255616
mRNA	ID=cds4088	4394088-4395425	38.646447	0	3
mRNA	ID=cds3102	3298774-3299298	38.516073	0	0
mRNA	ID=cds1831	1921743-1922615	38.047634	0	0
mRNA	ID=cds151	176610-176954	38	0	3
mRNA	ID=cds3016	3209129-3210874	38	0	3
mRNA	ID=cds396	424235-425305	38	0	2
mRNA	ID=cds766	819107-819811	38	0	2
mRNA	ID=cds2833	3013182-3013760	38	0	1
mRNA	ID=cds3226	3434540-3435916	38	0	1
repeat_region		436344-436380	38	0	0
mRNA	ID=cds1825	1915534-1918167	38	0	0
mRNA	ID=cds2381	2516489-2516833	38	0	0
mRNA	ID=cds2411	2547668-2548567	38	0	0
mRNA	ID=cds2948	3141008-3142186	38	0	0
mRNA	ID=cds3225	3433229-3434518	38	0	0
mRNA	ID=cds2911	3097704-3098750	37.5	0	1
mRNA	ID=cds3725	3967054-3968100	37.5	0	0
repeat_region		244129-244263	37.285482	0	0
mRNA	ID=cds1241	1316451-1317809	37.211324	0	0
mRNA	ID=cds3216	3427258-3427812	37	0	7
mRNA	ID=cds4082	4388480-4389532	37	0	4
mRNA	ID=cds3746	3992785-3993609	37	0	3
mRNA	ID=cds3238	3442127-3442561	37	0	2
mRNA	ID=cds2011	2095345-2096325	37	0	1
mRNA	ID=cds774	826468-828204	37	0	1
mRNA	ID=cds2707	2868277-2869326	37	0	0
mRNA	ID=cds3623	3853137-3853634	37	0	0
mRNA	ID=cds659	698797-699549	37	0	0
mRNA	ID=cds459	490636-491187	36.564274	0	0
mgeRNA	gbkey=mobile_element	4500113-4501538	36.447526	0	0
repeat_region		2537629-2537664	36.445096	0	0
mRNA	ID=cds996	1078528-1080036	36.288675	0	0
repeat_region		4251924-4252053	36.076294	0	0
repeat_region		2428951-2429034	36.014365	0	0.271403
mRNA	ID=cds2825	3002030-3003808	36	0	3
mRNA	ID=cds2389	2524968-2525966	36	0	1
mRNA	ID=cds3131	3331732-3332703	36	0	1
mRNA	ID=cds628	663325-664413	36	0	1
mRNA	ID=cds653	690129-691007	36	0	1
mRNA	ID=cds736	787020-788060	36	0	1
mRNA	ID=cds176	203348-204496	36	0	0
mRNA	ID=cds2569	2722150-2722473	36	0	0
mRNA	ID=cds3188	3388605-3390050	36	0	0
mRNA	ID=cds3466	3681653-3683641	36	0	0
mRNA	ID=cds3577	3803176-3803973	36	0	0
mRNA	ID=cds3724	3965939-3967042	36	0	0
repeat_region		2441811-2441890	35.543242	0	0.696666
mRNA	ID=cds2392	2528269-2529255	35.539358	0	1

mRNA	ID=cds2183	2294384-2295046	35.5	0	0
mRNA	ID=cds179	208621-209580	35.254308	0	0
mRNA	ID=cds2826	3004284-3005474	35.183	0	0
mRNA	ID=cds227	254259-255716	35.042076	0	0
mRNA	ID=cds2501	2643035-2645347	35	0	2
mRNA	ID=cds985	1066335-1066931	35	0	2
mRNA	ID=cds1525	1625541-1626287	35	0	1
mRNA	ID=cds1597	1680906-1682207	35	0	1
mRNA	ID=cds3253	3448270-3448548	35	0	1
mRNA	ID=cds4306	4625338-4626570	35	0	1
mRNA	ID=cds715	763403-764272	35	0	1
mRNA	ID=cds2114	2215422-2216579	35	0	0
mRNA	ID=cds2314	2444410-2445495	35	0	0
mRNA	ID=cds2461	2597928-2598500	35	0	0
mRNA	ID=cds2484	2623137-2624678	35	0	0
mRNA	ID=cds2879	3066195-3066830	35	0	0
mRNA	ID=cds3820	4078322-4079251	35	0	0
mRNA	ID=cds406	433871-434341	35	0	0
mRNA	ID=cds4074	4377806-4378540	35	0	0
mRNA	ID=cds425	452813-453391	35	0	0
mRNA	ID=cds461	493300-493629	35	0	0
repeat_region		740177-740282	34.946637	0	0.403118
mRNA	ID=cds799	854047-854967	34.5	0	4
mRNA	ID=cds1999	2086328-2087152	34	0	11
mRNA	ID=cds3745	3992545-3992748	34	0	2
mRNA	ID=cds3758	4003887-4005716	34	0	2
repeat_region		176969-176987	34	0	1
mRNA	ID=cds1650	1737935-1739146	34	0	1
mRNA	ID=cds2946	3138814-3139308	34	0	1
mRNA	ID=cds3204	3408302-3409267	34	0	1
mRNA	ID=cds3366	3566056-3567351	34	0	1
mRNA	ID=cds3991	4289535-4291193	34	0	1
mRNA	ID=cds518	556098-556964	34	0	1
mRNA	ID=cds1835	1924144-1924806	34	0	0
mRNA	ID=cds2316	2446628-2447179	34	0	0
mRNA	ID=cds2330	2462274-2463029	34	0	0
mRNA	ID=cds4246	4555401-4556312	34	0	0
repeat_region		983584-983681	33.849632	0	1.236427
mRNA	ID=cds3771	4018249-4019889	33.68761	0	2
repeat_region		3998173-3998275	33.665583	0	0.032306
mRNA	ID=cds562	587205-590177	33.5	0	1
mRNA	ID=cds3133	3333257-3334516	33.5	0	0
mRNA	ID=cds3485	3710259-3710957	33	0	4
mRNA	ID=cds1702	1795983-1796966	33	0	2
mRNA	ID=cds149	173602-174882	33	0	1
mRNA	ID=cds2503	2650516-2651361	33	0	1
mRNA	ID=cds3596	3820129-3820404	33	0	1
mRNA	ID=cds4171	4476496-4476912	33	0	1
mRNA	ID=cds166	192872-193429	33	0	0.5
mRNA	ID=cds1045	1127062-1128597	33	0	0
mRNA	ID=cds154	178455-179153	33	0	0
mRNA	ID=cds1644	1732459-1733274	33	0	0
mRNA	ID=cds1824	1914282-1915565	33	0	0
mRNA	ID=cds2162	2270386-2272200	33	0	0
mRNA	ID=cds3598	3822538-3823227	33	0	0
mRNA	ID=cds392	418815-420134	33	0	0
mRNA	ID=cds4308	4628756-4630693	33	0	0
mRNA	ID=cds1653	1741481-1742854	32.745001	0	2.5
mRNA	ID=cds1844	1934676-1935545	32	0	5
mRNA	ID=cds414	440773-442221	32	0	3
mRNA	ID=cds2118	2221960-2222892	32	0	2
mRNA	ID=cds3145	3341966-3342691	32	0	2
mRNA	ID=cds729	778821-779612	32	0	2
mRNA	ID=cds2203	2318065-2319891	32	0	1
mRNA	ID=cds2272	2401973-2402635	32	0	1
mRNA	ID=cds3566	3792010-3792942	32	0	1
repeat_region		4048056-4048134	32	0	0
repeat_region		936447-936482	32	0	0
mRNA	ID=cds1742	1834097-1835263	32	0	0
mRNA	ID=cds2891	3079935-3080693	32	0	0
mRNA	ID=cds3728	3970545-3971612	32	0	0
mRNA	ID=cds848	904963-905976	32	0	0
mRNA	ID=cds2491	2632254-2633624	31.5	0	2
mRNA	ID=cds1094	1176543-1177787	31.5	0	0
mRNA	ID=cds875	943256-944119	31.44967	0	0
mRNA	ID=cds3586	3810754-3811974	31	0	4
mRNA	ID=cds4159	4465454-4465507	31	0	4
mRNA	ID=cds759	812749-814770	31	0	4
mRNA	ID=cds3424	3627558-3628625	31	0	3
mRNA	ID=cds2573	2733053-2734033	31	0	2
mRNA	ID=cds3385	3585393-3586136	31	0	2
mRNA	ID=cds4106	4412298-4413923	31	0	1
mRNA	ID=cds772	824225-825331	31	0	1
mRNA	ID=cds859	919570-921516	31	0	1
mRNA	ID=cds1558	1645958-1646365	31	0	0
mRNA	ID=cds2872	3057775-3058668	31	0	0
mRNA	ID=cds311	328687-330720	31	0	0
mRNA	ID=cds3306	3495850-3497223	31	0	0
mRNA	ID=cds3464	3678467-3679963	31	0	0
mRNA	ID=cds3986	4283436-4285394	31	0	0
mRNA	ID=cds4293	4610434-4611507	31	0	0
mRNA	ID=cds431	460675-460947	31	0	0
mRNA	ID=cds630	665539-667440	31	0	0
mRNA	ID=cds734	784856-785908	31	0	0
mRNA	ID=cds861	922136-922456	31	0	0
repeat_region		4146453-4146534	30.990198	0	0
repeat_region		111430-111629	30.962901	0	0.013654
mRNA	ID=cds3694	3926175-3927626	30.5	0	1
mRNA	ID=cds2394	2530431-2531402	30.5	0	0
mRNA	ID=cds3821	4079248-4079883	30.5	0	0
mRNA	ID=cds2963	3155672-3156598	30.490552	0	0
mRNA	ID=cds4151	4455337-4455888	30.452522	0	2
repeat_region		1942243-1942369	30.443904	0	0.013654
mRNA	ID=cds2117	2220207-2221922	30.353553	0	0
mRNA	ID=cds1103	1185067-1186293	30.347404	0	0
repeat_region		3302526-3302554	30.078109	0	19
mRNA	ID=cds1062	1144163-1145122	30	0	2
mRNA	ID=cds2327	2458672-2458956	30	0	2
repeat_region		1734007-1734080	30	0	1.082189
mRNA	ID=cds125	145081-146310	30	0	1
mRNA	ID=cds2604	2761559-2763175	30	0	1
mRNA	ID=cds3294	3483436-3483840	30	0	1
mRNA	ID=cds88	100765-102240	30	0	1
mRNA	ID=cds1185	1258347-1260026	30	0	0
mRNA	ID=cds1342	1417789-1418265	30	0	0
mRNA	ID=cds1574	1656093-1658519	30	0	0
mRNA	ID=cds168	194903-195664	30	0	0
mRNA	ID=cds2851	3037877-3038398	30	0	0
mRNA	ID=cds3007	3201332-3202153	30	0	0
mRNA	ID=cds3792	4044989-4047775	30	0	0
mRNA	ID=cds668	709862-709948	30	0	0
mRNA	ID=cds775	828197-829195	30	0	0
mRNA	ID=cds896	969896-970078	30	0	0
repeat_region		216027-216137	29.68319	0	0.052492
mRNA	ID=cds752	805221-806504	29.530022	0	0
mRNA	ID=cds1154	1223772-1224584	29.5	0	0
mRNA	ID=cds3179	3380222-3381289	29.183892	0	1
mRNA	ID=cds1866	1956544-1957290	29	0	1
mRNA	ID=cds2067	2163692-2165053	29	0	1
mRNA	ID=cds2140	2245085-2246554	29	0	1
mRNA	ID=cds2632	2793696-2794358	29	0	1
mRNA	ID=cds141	160782-161486	29	0	0
mRNA	ID=cds142	161501-162031	29	0	0
mRNA	ID=cds1648	1735868-1736893	29	0	0
mRNA	ID=cds1654	1742895-1744151	29	0	0
mRNA	ID=cds2107	2209748-2210218	29	0	0
mRNA	ID=cds2366	2496693-2498390	29	0	0
mRNA	ID=cds3560	3784861-3786120	29	0	0

mRNA	ID=cds3567	3792952-3793998	29	0	0
mRNA	ID=cds3617	3848159-3848749	29	0	0
mRNA	ID=cds3816	4075038-4075475	29	0	0
mRNA	ID=cds397	425361-426488	29	0	0
mRNA	ID=cds3990	4288499-4289455	29	0	0
mRNA	ID=cds467	499349-500653	29	0	0
mRNA	ID=cds611	647262-648794	29	0	0
mRNA	ID=cds701	745158-745949	29	0	0
mRNA	ID=cds790	845683-846342	29	0	0
mRNA	ID=cds827	887357-889042	29	0	0
mRNA	ID=cds884	954095-955855	29	0	0
mRNA	ID=cds1862	1950726-1951469	28.684342	0	3
mRNA	ID=cds2822	2998367-3000625	28.341874	0	0
mRNA	ID=cds2661	2820730-2821791	28.333333	0	2
mRNA	ID=cds1642	1727111-1731727	28	0	2
mRNA	ID=cds1402	1487988-1488737	28	0	1
mRNA	ID=cds2572	2732325-2733056	28	0	1
mRNA	ID=cds2968	3161737-3163995	28	0	1
mRNA	ID=cds3149	3345137-3345991	28	0	1
mRNA	ID=cds496	532235-533050	28	0	1
mRNA	ID=cds150	175107-176528	28	0	0
mRNA	ID=cds1590	1672996-1674384	28	0	0
mRNA	ID=cds1703	1797250-1797294	28	0	0
mRNA	ID=cds2582	2740405-2741631	28	0	0
mRNA	ID=cds2866	3053634-3053963	28	0	0
mRNA	ID=cds3094	3293831-3294421	28	0	0
mRNA	ID=cds3256	3449703-3450308	28	0	0
mRNA	ID=cds672	712210-712755	28	0	0
mRNA	ID=cds4178	4485341-4486423	27.5	0	0
mRNA	ID=cds3139	3337278-3338087	27.387298	0	0
mRNA	ID=cds4307	4626878-4628545	27.281932	0	0
mRNA	ID=cds3252	3447923-3448255	27.103216	0	1
mRNA	ID=cds3547	3773595-3774182	27	0	5
mRNA	ID=cds1497	1598312-1599265	27	0	3
mRNA	ID=cds3891	4159794-4160153	27	0	3
mRNA	ID=cds2072	2169453-2169751	27	0	2
mRNA	ID=cds3015	3208803-3209018	27	0	2
mRNA	ID=cds3769	4016878-4017633	27	0	2
mRNA	ID=cds1227	1305209-1306669	27	0	1
mRNA	ID=cds214	245065-245805	27	0	1
mRNA	ID=cds2276	2406884-2407483	27	0	1
mRNA	ID=cds3512	3737728-3738981	27	0	1
mRNA	ID=cds4137	4437895-4439238	27	0	1
mRNA	ID=cds4139	4440405-4442138	27	0	1
mRNA	ID=cds2759	2927598-2928965	27	0	0.5
mRNA	ID=cds3333	3520893-3523445	27	0	0.5
repeat_region		1039766-1039801	27	0	0
mRNA	ID=cds1095	1177816-1178727	27	0	0
mRNA	ID=cds1968	2060415-2061347	27	0	0
mRNA	ID=cds2658	2816983-2817168	27	0	0
mRNA	ID=cds2860	3047182-3047571	27	0	0
mRNA	ID=cds3093	3293416-3293811	27	0	0
mRNA	ID=cds3255	3449404-3449706	27	0	0
mRNA	ID=cds3788	4040438-4041424	27	0	0
mRNA	ID=cds3994	4292504-4293817	27	0	0
mRNA	ID=cds404	432226-432675	27	0	0
mRNA	ID=cds894	967589-968575	27	0	0
mRNA	ID=cds3065	3264149-3265087	26.620223	0	0
mRNA	ID=cds1326	1404587-1405819	26.448022	0	0
mRNA	ID=cds2949	3142176-3143162	26.40965	0	0
mRNA	ID=cds143	162105-164534	26.341565	0	0.666666
mRNA	ID=cds681	720953-723637	26.320844	0	2
mRNA	ID=cds4086	4392089-4393636	26.25	0	1
mRNA	ID=cds699	743466-744398	26.106774	0	3
mRNA	ID=cds817	877965-879080	26	0	3
mRNA	ID=cds1040	1122630-1123277	26	0	2
mRNA	ID=cds1214	1289465-1290478	26	0	2
mRNA	ID=cds1212	1287897-1288355	26	0	1
mRNA	ID=cds1408	1492172-1493095	26	0	1
mRNA	ID=cds3602	3828480-3830189	26	0	1
mRNA	ID=cds2457	2593896-2594759	26	0	0.666666
repeat_region		3830201-3830227	26	0	0
mRNA	ID=cds1092	1174650-1175849	26	0	0
mRNA	ID=cds1601	1686600-1687775	26	0	0
mRNA	ID=cds2247	2373984-2374841	26	0	0
mRNA	ID=cds3691	3924035-3924478	26	0	0
mRNA	ID=cds369	395863-397083	26	0	0
mRNA	ID=cds380	405629-406153	26	0	0
mRNA	ID=cds3956	4251039-4251911	26	0	0
mRNA	ID=cds4254	4562722-4563873	26	0	0
mRNA	ID=cds4288	4606208-4606654	26	0	0
mRNA	ID=cds440	469860-471641	26	0	0
mRNA	ID=cds939	1020953-1023106	26	0	0
mRNA	ID=cds177	204493-205089	25.858173	0	0
mRNA	ID=cds543	575009-576048	25.751337	0	1
mRNA	ID=cds4176	4482463-4483974	25.711325	0	1
mRNA	ID=cds3181	3382725-3383195	25.646447	0	2
mRNA	ID=cds3221	3430013-3430486	25.526521	0	0
mRNA	ID=cds2478	2616097-2616798	25.5	0	0
mRNA	ID=cds2577	2735767-2736927	25.267261	0	0
mRNA	ID=cds2303	2432846-2433658	25.004996	0	0.003361
mRNA	ID=cds1361	1433209-1433643	25	0	3
mRNA	ID=cds3861	4120403-4121362	25	0	3
mRNA	ID=cds1908	1996518-1997504	25	0	2
mRNA	ID=cds3103	3299507-3300502	25	0	2
mRNA	ID=cds2862	3049137-3050339	25	0	1
mRNA	ID=cds2965	3156949-3159168	25	0	1
mRNA	ID=cds3130	3331162-3331473	25	0	1
mRNA	ID=cds3651	3882359-3882499	25	0	1
mRNA	ID=cds3761	4007193-4008215	25	0	1
mRNA	ID=cds3768	4015356-4016783	25	0	1
mRNA	ID=cds906	982873-983520	25	0	1
other ncRNA		2885376-2885431	25	0	0
mRNA	ID=cds1100	1182049-1182843	25	0	0
mRNA	ID=cds1164	1231723-1232253	25	0	0
mRNA	ID=cds1190	1264235-1265317	25	0	0
mRNA	ID=cds1261	1338582-1339751	25	0	0
mRNA	ID=cds1570	1653832-1654173	25	0	0
mRNA	ID=cds1849	1940686-1941441	25	0	0
mRNA	ID=cds2115	2216586-2217503	25	0	0
mRNA	ID=cds2163	2272201-2273295	25	0	0
mRNA	ID=cds2507	2654770-2655105	25	0	0
mRNA	ID=cds2651	2808792-2809322	25	0	0
mRNA	ID=cds2780	2954018-2956906	25	0	0
mRNA	ID=cds2797	2975659-2976921	25	0	0
mRNA	ID=cds2863	3050362-3051540	25	0	0
mRNA	ID=cds3045	3246461-3246844	25	0	0
mRNA	ID=cds3583	3809273-3809440	25	0	0
mRNA	ID=cds3918	4197527-4198117	25	0	0
mRNA	ID=cds78	89634-90092	25	0	0
mRNA	ID=cds811	870190-871110	25	0	0
mRNA	ID=cds887	958035-959318	25	0	0
mRNA	ID=cds974	1052657-1055401	25	0	0
mRNA	ID=cds3195	3399414-3401354	24.788676	0	0
repeat_region		3779721-3779749	24.75966	0	0
mgeRNA	gbkey=mobile_element	1049001-1049768	24.523054	0	0.666668
mRNA	ID=cds3564	3789378-3790574	24.5	0	0
mRNA	ID=cds2705	2866915-2867541	24.359211	0	0
mRNA	ID=cds773	825342-826475	24.19245	0	1
mRNA	ID=cds1188	1262100-1262723	24	0	4
mRNA	ID=cds1618	1704943-1707165	24	0	2
mRNA	ID=cds2533	2682276-2683529	24	0	2
mRNA	ID=cds22	21181-21399	24	0	1
mRNA	ID=cds2787	2963184-2964059	24	0	1
mRNA	ID=cds3532	3756040-3757884	24	0	1
mRNA	ID=cds3580	3806563-3807840	24	0	1
mRNA	ID=cds3726	3968156-3969286	24	0	1
mRNA	ID=cds4156	4461077-4462732	24	0	1

mRNA	ID=cds735	786066-786818	24	0	1
mRNA	ID=cds795	850237-851820	24	0	1
mRNA	ID=cds851	908554-910272	24	0	1
other ncRNA		2069339-2069542	24	0	0
mRNA	ID=cds0	190-255	24	0	0
mRNA	ID=cds1233	1310375-1310914	24	0	0
mRNA	ID=cds1240	1315246-1316439	24	0	0
mRNA	ID=cds1275	1351652-1352542	24	0	0
mRNA	ID=cds1496	1596641-1598233	24	0	0
mRNA	ID=cds1498	1599514-1601049	24	0	0
mRNA	ID=cds1578	1662530-1663144	24	0	0
mRNA	ID=cds1749	1840395-1841738	24	0	0
mRNA	ID=cds2136	2239832-2240989	24	0	0
mRNA	ID=cds2312	2442773-2443582	24	0	0
mRNA	ID=cds2511	2657925-2658311	24	0	0
mRNA	ID=cds2649	2807639-2808376	24	0	0
mRNA	ID=cds3047	3247397-3247702	24	0	0
mRNA	ID=cds46	49823-50302	24	0	0
mRNA	ID=cds842	900757-901473	24	0	0
mRNA	ID=cds3202	3405629-3407080	23.747565	0	0
mRNA	ID=cds879	947883-948791	23.695789	0	0
mRNA	ID=cds62	71351-72115	23.5	0	1
mRNA	ID=cds1187	1261249-1262100	23.5	0	0
mRNA	ID=cds1843	1932863-1934338	23.5	0	0
mRNA	ID=cds2518	2664729-2665868	23.5	0	0
mRNA	ID=cds1623	1710793-1712295	23.302805	0	1
mRNA	ID=cds1612	1701292-1702332	23.195047	0	1
mRNA	ID=cds2795	2971877-2974036	23.19245	0	2
repeat_region		3982243-3982346	23.148337	0	0.114819
mRNA	ID=cds629	664424-665536	23	0	4
mRNA	ID=cds897	970075-970821	23	0	3
mRNA	ID=cds2416	2551247-2552146	23	0	2.25
mRNA	ID=cds1941	2023535-2024350	23	0	2
mRNA	ID=cds2847	3033206-3034228	23	0	2
mRNA	ID=cds2983	3175303-3175932	23	0	2
mRNA	ID=cds4146	4449081-4450583	23	0	2
mRNA	ID=cds119	138835-141225	23	0	1
mRNA	ID=cds172	200482-200967	23	0	1
mRNA	ID=cds2776	2945779-2947032	23	0	1
mRNA	ID=cds3739	3984709-3985905	23	0	1
mRNA	ID=cds731	782389-783108	23	0	1
mRNA	ID=cds760	814962-815870	23	0	1
mRNA	ID=cds820	881199-881957	23	0	1
repeat_region		3043135-3043162	23	0	0
repeat_region		3275841-3275869	23	0	0
mRNA	ID=cds1065	1146590-1146763	23	0	0
mRNA	ID=cds1596	1680183-1680902	23	0	0
mRNA	ID=cds1795	1886085-1887770	23	0	0
mRNA	ID=cds1863	1951466-1952437	23	0	0
mRNA	ID=cds2024	2109101-2110000	23	0	0
mRNA	ID=cds2123	2227004-2227087	23	0	0
mRNA	ID=cds2164	2273295-2274320	23	0	0
mRNA	ID=cds2587	2743959-2744207	23	0	0
mRNA	ID=cds2905	3093120-3093824	23	0	0
mRNA	ID=cds2966	3159279-3160691	23	0	0
mRNA	ID=cds3938	4229382-4229654	23	0	0
mRNA	ID=cds3965	4259737-4260729	23	0	0
mRNA	ID=cds4221	4528553-4529674	23	0	0
mRNA	ID=cds788	842478-844703	23	0	0
mRNA	ID=cds3076	3275878-3276687	22.823223	0	1
mRNA	ID=cds3044	3245795-3246457	22.516376	0	0
mRNA	ID=cds107	120178-121551	22.5	0	3
mRNA	ID=cds4300	4618906-4619625	22.235702	0	0
mRNA	ID=cds2280	2410699-2411154	22	0	4
mRNA	ID=cds3147	3344195-3344482	22	0	2
mRNA	ID=cds1083	1163318-1164343	22	0	1
mRNA	ID=cds1606	1694486-1695076	22	0	1
mRNA	ID=cds1676	1767098-1768210	22	0	1
mRNA	ID=cds2444	2576688-2577638	22	0	1
mRNA	ID=cds2515	2661464-2662267	22	0	1
mRNA	ID=cds3556	3781684-3782151	22	0	1
mRNA	ID=cds3752	3999449-4000399	22	0	1
mRNA	ID=cds388	411831-414977	22	0	1
mRNA	ID=cds439	468095-469867	22	0	1
repeat_region		2438365-2438400	22	0	0
mRNA	ID=cds104	117752-118645	22	0	0
mRNA	ID=cds1166	1234161-1234880	22	0	0
mRNA	ID=cds1996	2083728-2085086	22	0	0
mRNA	ID=cds2141	2246759-2247640	22	0	0
mRNA	ID=cds2355	2482396-2485989	22	0	0
mRNA	ID=cds2493	2633906-2635378	22	0	0
mRNA	ID=cds2576	2735621-2735668	22	0	0
mRNA	ID=cds3323	3512404-3513081	22	0	0
mRNA	ID=cds3593	3816897-3817514	22	0	0
mRNA	ID=cds3770	4017647-4018252	22	0	0
mRNA	ID=cds3937	4228377-4229249	22	0	0
mRNA	ID=cds394	421742-423556	22	0	0
mRNA	ID=cds3982	4278003-4279652	22	0	0
mRNA	ID=cds4081	4387415-4388383	22	0	0
mRNA	ID=cds4125	4426102-4426740	22	0	0
mRNA	ID=cds877	945094-946242	22	0	0
mRNA	ID=cds4261	4571942-4574878	21.353553	0	0
mRNA	ID=cds723	773975-774379	21.288675	0	0
repeat_region		2833089-2833170	21.110651	0	0.595889
mRNA	ID=cds2903	3091522-3091938	21.013015	0	0
mRNA	ID=cds3856	4116538-4116783	21	0	4
mRNA	ID=cds2941	3134685-3136544	21	0	2
mRNA	ID=cds308	324801-326471	21	0	2
mRNA	ID=cds1251	1327356-1328405	21	0	1
mRNA	ID=cds2161	2268748-2270304	21	0	1
mRNA	ID=cds2710	2870531-2870842	21	0	1
mRNA	ID=cds2768	2938165-2939265	21	0	1
mRNA	ID=cds646	682700-683635	21	0	1
mRNA	ID=cds413	440325-440567	21	0	0.666666
repeat_region		2736937-2736960	21	0	0
other ncRNA		4156308-4156417	21	0	0
mRNA	ID=cds1077	1157092-1158525	21	0	0
mRNA	ID=cds1260	1338267-1338575	21	0	0
mRNA	ID=cds1305	1383535-1384596	21	0	0
mRNA	ID=cds1308	1386954-1387919	21	0	0
mRNA	ID=cds1589	1671937-1672971	21	0	0
mRNA	ID=cds1652	1740625-1741266	21	0	0
mRNA	ID=cds1890	1980578-1981564	21	0	0
mRNA	ID=cds1959	2041675-2042472	21	0	0
mRNA	ID=cds2021	2106361-2107608	21	0	0
mRNA	ID=cds2127	2229866-2230750	21	0	0
mRNA	ID=cds2305	2434737-2435873	21	0	0
mRNA	ID=cds2393	2529485-2530246	21	0	0
mRNA	ID=cds2597	2752918-2753400	21	0	0
mRNA	ID=cds2944	3137999-3138340	21	0	0
mRNA	ID=cds3150	3345988-3346260	21	0	0
mRNA	ID=cds3553	3779238-3779711	21	0	0
mRNA	ID=cds3748	3994310-3995206	21	0	0
mRNA	ID=cds3988	4287268-4287834	21	0	0
mRNA	ID=cds863	925448-925666	21	0	0
mRNA	ID=cds886	956876-957964	21	0	0
mRNA	ID=cds975	1055484-1056512	21	0	0
repeat_region		1408957-1408993	20.692494	0	0
mRNA	ID=cds3619	3849119-3850807	20.5	0	1
mRNA	ID=cds1099	1181006-1182052	20.5	0	0
mRNA	ID=cds968	1049250-1049753	20.494461	0	0.500001
mRNA	ID=cds515	553834-555219	20.312348	0	1
mRNA	ID=cds1465	1551996-1553693	20.130558	0	2
repeat_region		1041208-1041241	20.100734	0	2
mRNA	ID=cds1076	1156000-1156797	20	0	8
mRNA	ID=cds2462	2598500-2598970	20	0	2.044093
mRNA	ID=cds3187	3387542-3388471	20	0	2
mRNA	ID=cds129	148807-149601	20	0	1

mRNA	ID=cds2013	2097886-2099292	20	0	1
mRNA	ID=cds2264	2393065-2393367	20	0	1
mRNA	ID=cds2548	2699763-2700491	20	0	1
mRNA	ID=cds3148	3344600-3345091	20	0	1
mRNA	ID=cds3199	3403458-3403928	20	0	1
mRNA	ID=cds3600	3825483-3826688	20	0	1
mRNA	ID=cds6	6529-7959	20	0	1
mRNA	ID=cds850	907516-908517	20	0	1
mRNA	ID=cds930	1012482-1014122	20	0	1
mRNA	ID=cds1041	1123341-1124549	20	0	0
mRNA	ID=cds1120	1201482-1202156	20	0	0
mRNA	ID=cds1182	1255944-1257035	20	0	0
mRNA	ID=cds2120	2223823-2224401	20	0	0
mRNA	ID=cds212	243543-244121	20	0	0
mRNA	ID=cds2324	2454349-2454834	20	0	0
mRNA	ID=cds2382	2516835-2517227	20	0	0
mRNA	ID=cds2786	2962383-2963177	20	0	0
mRNA	ID=cds2919	3105042-3107177	20	0	0
mRNA	ID=cds2969	3164133-3165740	20	0	0
mRNA	ID=cds2978	3171164-3171478	20	0	0
mRNA	ID=cds3172	3374301-3374798	20	0	0
mRNA	ID=cds3322	3511653-3512411	20	0	0
mRNA	ID=cds3633	3862635-3864296	20	0	0
mRNA	ID=cds363	387977-388951	20	0	0
mRNA	ID=cds367	393730-394353	20	0	0
mRNA	ID=cds3702	3936250-3937242	20	0	0
mRNA	ID=cds373	399053-400147	20	0	0
mRNA	ID=cds3749	3995206-3995922	20	0	0
mRNA	ID=cds3781	4029184-4030515	20	0	0
mRNA	ID=cds3791	4043693-4044625	20	0	0
mRNA	ID=cds3979	4275083-4275406	20	0	0
mRNA	ID=cds402	430353-431237	20	0	0
mRNA	ID=cds4042	4347338-4348057	20	0	0
mRNA	ID=cds680	720279-720956	20	0	0
mRNA	ID=cds910	988377-989579	20	0	0
repeat_region		3672404-3672496	19.957904	0	0.047004
repeat_region		3096435-3096555	19.940487	0	0.834694
repeat_region		4482356-4482439	19.810302	0	2.332944
mRNA	ID=cds663	703167-705113	19.789513	0	0
mRNA	ID=cds725	775072-775500	19.752658	0	1
mRNA	ID=cds843	901480-902211	19.583334	0	0
mRNA	ID=cds1042	1124785-1125369	19.572138	0	2
repeat_region		173504-173588	19.509484	0	0.013654
mRNA	ID=cds3480	3701882-3702784	19.5	0	2
mRNA	ID=cds2304	2433658-2434671	19.5	0	0
mRNA	ID=cds2967	3160766-3161503	19.5	0	0
mRNA	ID=cds3696	3929339-3931207	19.5	0	0
mRNA	ID=cds3772	4019968-4020237	19.5	0	0
mRNA	ID=cds232	259612-260715	19.18102	0	1
mRNA	ID=cds800	855186-856778	19.057408	0	0
mRNA	ID=cds3766	4013377-4014192	19	0	5
mRNA	ID=cds3168	3369106-3370596	19	0	2
mRNA	ID=cds3191	3395807-3396400	19	0	2
mRNA	ID=cds3403	3603274-3603633	19	0	2
mRNA	ID=cds3459	3671385-3672398	19	0	2
mRNA	ID=cds856	915696-917354	19	0	2
mRNA	ID=cds1625	1713050-1713913	19	0	1
mRNA	ID=cds2589	2745984-2746775	19	0	1
mRNA	ID=cds2709	2869802-2870512	19	0	1
mRNA	ID=cds3283	3473740-3474462	19	0	1
mRNA	ID=cds3866	4126101-4126418	19	0	1
mgeRNA	gbkey=mobile_element	1501185-1502932	19	0	0
mRNA	ID=cds1204	1275045-1276841	19	0	0
mRNA	ID=cds1435	1516352-1516870	19	0	0
mRNA	ID=cds1592	1676451-1677395	19	0	0
mRNA	ID=cds1627	1715375-1716031	19	0	0
mRNA	ID=cds1818	1907332-1908123	19	0	0
mRNA	ID=cds1947	2028923-2030341	19	0	0
mRNA	ID=cds2449	2583753-2585453	19	0	0
mRNA	ID=cds2584	2742205-2742552	19	0	0
mRNA	ID=cds275	294920-296320	19	0	0
mRNA	ID=cds27	26277-27227	19	0	0
mRNA	ID=cds2803	2982433-2983614	19	0	0
mRNA	ID=cds2857	3041684-3043123	19	0	0
mRNA	ID=cds3048	3247705-3248109	19	0	0
mRNA	ID=cds3155	3351143-3352072	19	0	0
mRNA	ID=cds3170	3371720-3372511	19	0	0
mRNA	ID=cds3282	3473354-3473740	19	0	0
mRNA	ID=cds3862	4121454-4122479	19	0	0
mRNA	ID=cds3917	4196813-4197484	19	0	0
mRNA	ID=cds3921	4199286-4199711	19	0	0
mRNA	ID=cds4108	4414464-4414793	19	0	0
mRNA	ID=cds4180	4488164-4489162	19	0	0
mRNA	ID=cds4310	4631256-4631768	19	0	0
mRNA	ID=cds514	553166-553660	19	0	0
mRNA	ID=cds822	882896-884128	19	0	0
repeat_region		3311274-3311350	18.767638	0	0
mRNA	ID=cds2179	2290983-2292926	18.5	0	0
mRNA	ID=cds3176	3376892-3378019	18.269275	0	0
repeat_region		4616140-4616221	18.023013	0	15.025102
mRNA	ID=cds3706	3946109-3946447	18	0	4
mRNA	ID=cds2477	2615600-2615959	18	0	2
mRNA	ID=cds849	906075-907505	18	0	2
mRNA	ID=cds1191	1265317-1266150	18	0	1
mRNA	ID=cds195	222833-223408	18	0	1
mRNA	ID=cds2542	2695937-2696572	18	0	1
mRNA	ID=cds3002	3193342-3194775	18	0	1
mRNA	ID=cds3705	3945151-3945990	18	0	1
mRNA	ID=cds3996	4295242-4297389	18	0	1
mRNA	ID=cds410	436385-437359	18	0	1
mRNA	ID=cds4290	4607437-4609026	18	0	1
mRNA	ID=cds980	1061773-1062078	18	0	1
repeat_region		1862777-1862797	18	0	0
repeat_region		2796075-2796104	18	0	0
mRNA	ID=cds158	183709-184095	18	0	0
mRNA	ID=cds1616	1703791-1704372	18	0	0
mRNA	ID=cds175	202560-203348	18	0	0
mRNA	ID=cds1794	1884888-1886015	18	0	0
mRNA	ID=cds1798	1889349-1891259	18	0	0
mRNA	ID=cds1815	1906285-1906572	18	0	0
mRNA	ID=cds1851	1942370-1943380	18	0	0
mRNA	ID=cds1854	1944275-1944877	18	0	0
mRNA	ID=cds1917	2005701-2006114	18	0	0
mRNA	ID=cds213	244327-245094	18	0	0
mRNA	ID=cds2185	2295679-2296281	18	0	0
mRNA	ID=cds2451	2588829-2588888	18	0	0
mRNA	ID=cds2456	2591866-2593881	18	0	0
mRNA	ID=cds2480	2618268-2618894	18	0	0
mRNA	ID=cds2546	2698640-2699020	18	0	0
mRNA	ID=cds2738	2902769-2903440	18	0	0
mRNA	ID=cds2774	2943058-2943864	18	0	0
mRNA	ID=cds3427	3632864-3633916	18	0	0
mRNA	ID=cds3550	3775422-3777077	18	0	0
mRNA	ID=cds3591	3814699-3815562	18	0	0
mRNA	ID=cds4037	4339934-4341289	18	0	0
mRNA	ID=cds98	111856-112599	18	0	0
mRNA	ID=cds99	112599-113219	18	0	0
mRNA	ID=cds1769	1862806-1863660	17.618427	0	0
mRNA	ID=cds2590	2746796-2748082	17.5	0	0
mRNA	ID=cds2245	2371670-2373025	17.166667	0	0
repeat_region		2217647-2217680	17.101322	0	0.073238
mRNA	ID=cds161	185978-188650	17	0	6
mRNA	ID=cds3089	3286836-3289352	17	0	6
mRNA	ID=cds1599	1683209-1684612	17	0	4
mRNA	ID=cds2633	2794359-2794808	17	0	2
mRNA	ID=cds789	844964-845686	17	0	2
mRNA	ID=cds1119	1200999-1201307	17	0	1
mRNA	ID=cds1402	1487737-1487988	17	0	1

mRNA	ID=cds1609	1697379-1698971	17	0	1
mRNA	ID=cds2018	2103089-2104081	17	0	1
mRNA	ID=cds2137	2241006-2241674	17	0	1
mRNA	ID=cds2265	2393364-2393918	17	0	1
mRNA	ID=cds2796	2974621-2975652	17	0	1
mRNA	ID=cds2827	3005532-3006728	17	0	1
mRNA	ID=cds4311	4631820-4632467	17	0	1
repeat_region		3207521-3207540	17	0	0
mRNA	ID=cds1073	1153335-1154357	17	0	0
mRNA	ID=cds1093	1175842-1176543	17	0	0
mRNA	ID=cds115	134788-135582	17	0	0
mRNA	ID=cds1170	1239558-1241294	17	0	0
mRNA	ID=cds117	136570-136917	17	0	0
mRNA	ID=cds152	177001-177624	17	0	0
mRNA	ID=cds1711	1804394-1805323	17	0	0
mRNA	ID=cds1802	1892829-1894190	17	0	0
mRNA	ID=cds1836	1924803-1926863	17	0	0
mRNA	ID=cds2290	2420671-2421561	17	0	0
mRNA	ID=cds2309	2439786-2441792	17	0	0
mRNA	ID=cds2648	2806338-2807515	17	0	0
mRNA	ID=cds2870	3056688-3057347	17	0	0
mRNA	ID=cds2961	3153377-3154540	17	0	0
mRNA	ID=cds3144	3341402-3341959	17	0	0
mRNA	ID=cds3205	3409293-3409589	17	0	0
mRNA	ID=cds3301	3489747-3490319	17	0	0
mRNA	ID=cds3344	3535407-3537728	17	0	0
mRNA	ID=cds3375	3577791-3578828	17	0	0
mRNA	ID=cds3762	4008223-4009023	17	0	0
mRNA	ID=cds3960	4255765-4257144	17	0	0
mRNA	ID=cds4179	4486584-4488086	17	0	0
mRNA	ID=cds4191	4500126-4501454	17	0	0
mRNA	ID=cds4277	4597718-4598212	17	0	0
mRNA	ID=cds899	971845-972624	17	0	0
repeat_region		4604581-4604672	16.874526	0	0.223004
repeat_region		3212914-3212946	16.841886	0	0
mRNA	ID=cds2014	2100968-2101413	16.816325	0	0
mgeRNA	gbkey=mobile_element	1293649-1294628	16.552474	0	1
mRNA	ID=cds1388	1468541-1472037	16.5	0	0
mRNA	ID=cds561	586314-587204	16.478602	0	1
mRNA	ID=cds436	464836-466536	16.414858	0	2
mRNA	ID=cds1850	1941438-1942223	16.344115	0	0
mRNA	ID=cds2848	3034395-3036128	16.304211	0	1
mRNA	ID=cds4295	4612703-4613566	16.276642	0	3
mRNA	ID=cds4303	4622168-4622812	16.202861	0	2
repeat_region		489183-489280	16.123195	0	2.577781
mgeRNA	gbkey=mobile_element	1425623-1426818	16.109988	0	0
mRNA	ID=cds1526	1626376-1627062	16	0	2
mRNA	ID=cds1571	1654208-1654768	16	0	2
mRNA	ID=cds1586	1669984-1670805	16	0	2
mRNA	ID=cds2071	2166736-2167635	16	0	2
mRNA	ID=cds2112	2213767-2214498	16	0	2
mRNA	ID=cds229	256527-257771	16	0	2
mRNA	ID=cds126	146314-146694	16	0	1
mRNA	ID=cds1524	1623359-1625404	16	0	1
mRNA	ID=cds1808	1901106-1901906	16	0	1
mRNA	ID=cds2059	2151705-2151761	16	0	1
mRNA	ID=cds3689	3921080-3921703	16	0	1
mRNA	ID=cds3701	3935317-3936246	16	0	1
mRNA	ID=cds416	442828-443739	16	0	1
mRNA	ID=cds892	963543-965807	16	0	1
mRNA	ID=cds1217	1292750-1293367	16	0	0
mRNA	ID=cds1229	1307040-1308293	16	0	0
mRNA	ID=cds1246	1322122-1322742	16	0	0
mRNA	ID=cds1425	1508027-1509433	16	0	0
mRNA	ID=cds1500	1602071-1603063	16	0	0
mRNA	ID=cds1577	1661633-1662487	16	0	0
mRNA	ID=cds1649	1736890-1737822	16	0	0
mRNA	ID=cds1753	1846149-1846700	16	0	0
mRNA	ID=cds1812	1904275-1905084	16	0	0
mRNA	ID=cds1857	1946204-1946656	16	0	0
mRNA	ID=cds2020	2105250-2106353	16	0	0
mRNA	ID=cds2246	2373022-2373984	16	0	0
mRNA	ID=cds2307	2436964-2438142	16	0	0
mRNA	ID=cds2509	2656974-2657489	16	0	0
mRNA	ID=cds2547	2699020-2699751	16	0	0
mRNA	ID=cds2581	2739897-2740415	16	0	0
mRNA	ID=cds2603	2759373-2761562	16	0	0
mRNA	ID=cds2804	2983869-2985098	16	0	0
mRNA	ID=cds3074	3275024-3275359	16	0	0
mRNA	ID=cds3164	3365849-3366976	16	0	0
mRNA	ID=cds3290	3479311-3481224	16	0	0
mRNA	ID=cds3338	3527796-3528674	16	0	0
mRNA	ID=cds3351	3543646-3544221	16	0	0
mRNA	ID=cds3444	3652706-3653236	16	0	0
mRNA	ID=cds3483	3706807-3708498	16	0	0
mRNA	ID=cds3703	3937208-3938635	16	0	0
mRNA	ID=cds3787	4040092-4040361	16	0	0
mRNA	ID=cds3932	4218324-4220510	16	0	0
mRNA	ID=cds4260	4570437-4571939	16	0	0
mRNA	ID=cds426	453696-454013	16	0	0
mRNA	ID=cds453	483650-484843	16	0	0
mRNA	ID=cds573	604741-605109	16	0	0
mRNA	ID=cds709	754783-755130	16	0	0
mRNA	ID=cds777	830095-831459	16	0	0
mRNA	ID=cds868	931818-932312	16	0	0
mRNA	ID=cds466	498238-499197	15.591752	0	2
mRNA	ID=cds2775	2944103-2945200	15.532293	0	0
mRNA	ID=cds2337	2469099-2469539	15.5	0	0
mRNA	ID=cds2728	2890679-2891950	15.361954	0	1
mRNA	ID=cds1102	1183681-1184817	15.333333	0	1
repeat_region		1937123-1937152	15.031886	0	0
mRNA	ID=cds2375	2507652-2508908	15	0	14
mRNA	ID=cds4218	4526134-4526940	15	0	3
mRNA	ID=cds623	658474-659439	15	0	3
mRNA	ID=cds2133	2235791-2237311	15	0	2
mRNA	ID=cds2708	2869323-2869802	15	0	2
mRNA	ID=cds1433	1515672-1515905	15	0	1
mRNA	ID=cds1634	1720145-1722157	15	0	1
mRNA	ID=cds2585	2742594-2743361	15	0	1
mRNA	ID=cds3157	3357220-3358638	15	0	1
mRNA	ID=cds4135	4436731-4437285	15	0	1
mRNA	ID=cds465	497279-498241	15	0	1
mRNA	ID=cds5	5683-6459	15	0	1
mRNA	ID=cds738	789206-790252	15	0	1
mRNA	ID=cds983	1064808-1066049	15	0	1
repeat_region		2221927-2221954	15	0	0
repeat_region		464037-464061	15	0	0
repeat_region		50327-50363	15	0	0
repeat_region		685973-685997	15	0	0
mRNA	ID=cds1013	1099519-1100010	15	0	0
mRNA	ID=cds1234	1310944-1311687	15	0	0
mRNA	ID=cds1409	1493312-1494655	15	0	0
mRNA	ID=cds1550	1643143-1643298	15	0	0
mRNA	ID=cds1629	1716517-1717626	15	0	0
mRNA	ID=cds1636	1722760-1723656	15	0	0
mRNA	ID=cds1738	1831425-1832135	15	0	0
mRNA	ID=cds1809	1901919-1902770	15	0	0
mRNA	ID=cds1813	1905250-1905459	15	0	0
mRNA	ID=cds1852	1943389-1944000	15	0	0
mRNA	ID=cds1888	1978212-1979636	15	0	0
mRNA	ID=cds2106	2209247-2209708	15	0	0
mRNA	ID=cds2108	2210265-2210984	15	0	0
mRNA	ID=cds2204	2319888-2321273	15	0	0
mRNA	ID=cds2377	2509490-2510728	15	0	0
mRNA	ID=cds2537	2686815-2687528	15	0	0
mRNA	ID=cds2689	2849023-2849895	15	0	0
mRNA	ID=cds2767	2937390-2938121	15	0	0
mRNA	ID=cds2829	3008050-3009435	15	0	0

mRNA	ID=cds3105	3301470-3302477	15	0	0
mRNA	ID=cds3534	3759370-3759978	15	0	0
mRNA	ID=cds3561	3786124-3787083	15	0	0
mRNA	ID=cds379	404988-405446	15	0	0
mRNA	ID=cds4184	4492646-4493209	15	0	0
mRNA	ID=cds435	464076-464771	15	0	0
mRNA	ID=cds695	738730-740148	15	0	0
mRNA	ID=cds900	972760-973545	15	0	0
mRNA	ID=cds3535	3760206-3764339	14.951462	0	0.5
repeat_region		1999992-2000090	14.745401	0	1.114819
mRNA	ID=cds687	728806-732999	14.647064	0	2
mRNA	ID=cds378	404059-404868	14.646447	0	0
mRNA	ID=cds1131	1207355-1207768	14.5	0	0
mRNA	ID=cds1181	1252308-1255175	14.5	0	0
mRNA	ID=cds762	817278-817790	14.5	0	0
repeat_region		2250818-2250908	14.428462	0	1
mRNA	ID=cds3116	3315576-3316028	14.132288	0	0
mRNA	ID=cds3128	3329792-3330757	14.126881	0	0
mRNA	ID=cds1355	1425770-1426750	14.109988	0	0
repeat_region		1532907-1532981	14.107417	0	0.079063
mRNA	ID=cds798	852870-853988	14.101138	0	0
mRNA	ID=cds2915	3101035-3102087	14	0	6
mRNA	ID=cds2368	2499152-2500009	14	0	4
mRNA	ID=cds2174	2284412-2286936	14	0	2
mRNA	ID=cds2564	2715513-2716550	14	0	2
mRNA	ID=cds1527	1627239-1627442	14	0	1
mRNA	ID=cds1573	1655589-1655894	14	0	1
mRNA	ID=cds1607	1695297-1696064	14	0	1
mRNA	ID=cds1807	1900072-1901043	14	0	1
mRNA	ID=cds2302	2432104-2432763	14	0	1
mRNA	ID=cds2395	2531786-2532043	14	0	1
mRNA	ID=cds2579	2738102-2739172	14	0	1
mRNA	ID=cds2640	2798156-2798497	14	0	1
mRNA	ID=cds2662	2821871-2822368	14	0	1
mRNA	ID=cds2792	2969293-2969511	14	0	1
mRNA	ID=cds2830	3009483-3010415	14	0	1
mRNA	ID=cds3334	3523611-3524171	14	0	1
mRNA	ID=cds3677	3908508-3909548	14	0	1
mRNA	ID=cds3884	4151719-4152870	14	0	1
mRNA	ID=cds4269	4585932-4586888	14	0	1
mRNA	ID=cds479	514080-514997	14	0	1
mRNA	ID=cds776	829195-829866	14	0	1
mRNA	ID=cds881	949563-950303	14	0	1
mRNA	ID=cds3895	4170080-4171108	14	0	0.016434
mRNA	ID=cds1027	1112802-1113029	14	0	0
mRNA	ID=cds1028	1113030-1113404	14	0	0
mRNA	ID=cds1037	1120465-1120710	14	0	0
mRNA	ID=cds1038	1120784-1121830	14	0	0
mRNA	ID=cds1039	1121936-1122496	14	0	0
mRNA	ID=cds106	119281-120135	14	0	0
mRNA	ID=cds1096	1178854-1179582	14	0	0
mRNA	ID=cds1717	1808958-1810349	14	0	0
mRNA	ID=cds1814	1905472-1905615	14	0	0
mRNA	ID=cds2275	2405583-2406800	14	0	0
mRNA	ID=cds2354	2481777-2482391	14	0	0
mRNA	ID=cds2367	2498405-2499139	14	0	0
mRNA	ID=cds2380	2513665-2515854	14	0	0
mRNA	ID=cds2422	2556880-2558088	14	0	0
mRNA	ID=cds2933	3126294-3127058	14	0	0
mRNA	ID=cds3004	3197686-3198987	14	0	0
mRNA	ID=cds3153	3347828-3348481	14	0	0
mRNA	ID=cds3288	3476824-3478629	14	0	0
mRNA	ID=cds3452	3661913-3662641	14	0	0
mRNA	ID=cds3574	3800062-3801081	14	0	0
mRNA	ID=cds368	394354-395511	14	0	0
mRNA	ID=cds3710	3948583-3949566	14	0	0
mRNA	ID=cds3716	3955993-3957468	14	0	0
mRNA	ID=cds3794	4049370-4049879	14	0	0
mRNA	ID=cds3936	4227476-4228165	14	0	0
mRNA	ID=cds3958	4254660-4255028	14	0	0
mRNA	ID=cds3972	4267437-4268150	14	0	0
mRNA	ID=cds3984	4281276-4282925	14	0	0
mRNA	ID=cds4099	4407298-4408029	14	0	0
mRNA	ID=cds4154	4457923-4458387	14	0	0
mRNA	ID=cds4278	4598261-4598998	14	0	0
mRNA	ID=cds4305	4623935-4625317	14	0	0
mRNA	ID=cds470	504138-505790	14	0	0
mRNA	ID=cds535	570677-571474	14	0	0
mRNA	ID=cds570	601182-602558	14	0	0
mRNA	ID=cds595	631405-631602	14	0	0
mRNA	ID=cds737	788054-789202	14	0	0
mRNA	ID=cds791	846481-847227	14	0	0
mRNA	ID=cds796	851894-852163	14	0	0
mRNA	ID=cds1023	1105578-1106999	13.89072	0	0
mRNA	ID=cds3354	3548102-3550495	13.874503	0	0
repeat_region		3703986-3704083	13.816192	0	0.025102
repeat_region		4101530-4101613	13.713457	0	0.055917
mRNA	ID=cds3038	3237966-3239210	13.655885	0	0
repeat_region		4315940-4316022	13.545594	0	0.047004
mRNA	ID=cds1074	1154347-1154988	13.5	0	0
mRNA	ID=cds3782	4030515-4031129	13.5	0	0
mRNA	ID=cds3863	4122635-4124833	13.5	0	0
repeat_region		4631114-4631253	13.33932	0	0.7698
mRNA	ID=cds3414	3614205-3615038	13.122939	0	1
mRNA	ID=cds478	513625-514083	13	0	6
mRNA	ID=cds1171	1241389-1242303	13	0	2
mRNA	ID=cds926	1005714-1006823	13	0	2
mRNA	ID=cds1101	1182840-1183667	13	0	1
mRNA	ID=cds1619	1707166-1708224	13	0	1
mRNA	ID=cds1720	1811891-1814152	13	0	1
mRNA	ID=cds2390	2525963-2526181	13	0	1
mRNA	ID=cds2596	2752310-2752786	13	0	1
mRNA	ID=cds2670	2827835-2828800	13	0	1
mRNA	ID=cds2676	2836276-2837286	13	0	1
mRNA	ID=cds2850	3036869-3037765	13	0	1
mRNA	ID=cds4189	4497616-4498557	13	0	1
mRNA	ID=cds4302	4621124-4622140	13	0	1
mRNA	ID=cds444	474603-475175	13	0	1
mRNA	ID=cds3734	3976624-3977976	13	0	0.25
repeat_region		141978-141998	13	0	0
repeat_region		881163-881189	13	0	0
mRNA	ID=cds1079	1161108-1161467	13	0	0
mRNA	ID=cds1085	1165308-1166612	13	0	0
mRNA	ID=cds1193	1266543-1267352	13	0	0
mRNA	ID=cds124	144577-145017	13	0	0
mRNA	ID=cds1314	1391251-1392864	13	0	0
mRNA	ID=cds1450	1532989-1533882	13	0	0
mRNA	ID=cds1468	1554649-1555080	13	0	0
mRNA	ID=cds1615	1703274-1703714	13	0	0
mRNA	ID=cds1640	1725861-1726268	13	0	0
mRNA	ID=cds1657	1746771-1747583	13	0	0
mRNA	ID=cds169	195677-196534	13	0	0
mRNA	ID=cds1741	1833539-1834087	13	0	0
mRNA	ID=cds1823	1913655-1914152	13	0	0
mRNA	ID=cds1981	2072803-2074335	13	0	0
mRNA	ID=cds1991	2078813-2079286	13	0	0
mRNA	ID=cds2025	2110000-2111085	13	0	0
mRNA	ID=cds2084	2179118-2180083	13	0	0
mRNA	ID=cds2157	2264267-2265733	13	0	0
mRNA	ID=cds2189	2298289-2300775	13	0	0
mRNA	ID=cds2333	2465877-2466239	13	0	0
mRNA	ID=cds2680	2841058-2841468	13	0	0
mRNA	ID=cds2681	2841465-2842232	13	0	0
mRNA	ID=cds2794	2970691-2971884	13	0	0
mRNA	ID=cds2877	3064299-3065195	13	0	0
mRNA	ID=cds2906	3093842-3094408	13	0	0
mRNA	ID=cds2912	3098926-3099645	13	0	0
mRNA	ID=cds2953	3145919-3146959	13	0	0

mRNA	ID=cds2973	3167306-3167698	13	0	0
mRNA	ID=cds3014	3207552-3208565	13	0	0
mRNA	ID=cds3137	3335932-3336483	13	0	0
mRNA	ID=cds3203	3407092-3407973	13	0	0
mRNA	ID=cds3279	3472200-3472574	13	0	0
mRNA	ID=cds3347	3540750-3540986	13	0	0
mRNA	ID=cds3408	3607978-3608535	13	0	0
mRNA	ID=cds3430	3637408-3637743	13	0	0
mRNA	ID=cds3616	3846657-3848159	13	0	0
mRNA	ID=cds3729	3971631-3972512	13	0	0
mRNA	ID=cds3797	4051892-4053301	13	0	0
mRNA	ID=cds3873	4135955-4137058	13	0	0
mRNA	ID=cds3967	4261271-4262254	13	0	0
mRNA	ID=cds3973	4268261-4268677	13	0	0
mRNA	ID=cds4315	4634719-4636143	13	0	0
mRNA	ID=cds445	475206-475595	13	0	0
mRNA	ID=cds66	75644-77299	13	0	0
mRNA	ID=cds818	879077-879703	13	0	0
mRNA	ID=cds8	9306-9893	13	0	0
repeat_region		4448967-4449051	12.929555	0	0.073238
other ncRNA		3698159-3698224	12.695872	0	2.409121
mRNA	ID=cds3919	4198304-4198576	12.68586	0	0
repeat_region		430192-430289	12.545749	0	2.214239
mRNA	ID=cds3853	4112592-4113602	12.528595	0	0
mRNA	ID=cds3546	3772447-3773595	12.5	0	1
mRNA	ID=cds4266	4579482-4581071	12.333333	0	0
mRNA	ID=cds3428	3634231-3635433	12.223607	0	0
mRNA	ID=cds3049	3248099-3248398	12.080845	0	0
repeat_region		2892830-2892896	12.072284	0	0.008913
repeat_region		2509374-2509461	12.066512	0	0.116202
mRNA	ID=cds3814	4073576-4074175	12	0	3
mRNA	ID=cds2061	2153287-2156409	12	0	2
mRNA	ID=cds2306	2435972-2436967	12	0	2
mRNA	ID=cds2771	2940940-2941167	12	0	2
mRNA	ID=cds3353	3546008-3548092	12	0	2
mRNA	ID=cds1329	1409037-1409972	12	0	1
mRNA	ID=cds1965	2056227-2057714	12	0	1
mRNA	ID=cds2003	2088216-2089115	12	0	1
mRNA	ID=cds2110	2212888-2213619	12	0	1
mRNA	ID=cds2248	2374856-2375614	12	0	1
mRNA	ID=cds2251	2378744-2379049	12	0	1
mRNA	ID=cds3046	3246991-3247359	12	0	1
mRNA	ID=cds3590	3813886-3814572	12	0	1
mRNA	ID=cds3783	4031168-4032619	12	0	1
mRNA	ID=cds4097	4404213-4404638	12	0	1
mRNA	ID=cds4301	4619792-4621123	12	0	1
mRNA	ID=cds61	70387-71265	12	0	1
repeat_region		1114849-1114875	12	0	0
repeat_region		3334941-3334974	12	0	0
repeat_region		4595120-4595158	12	0	0
other ncRNA		1403676-1403833	12	0	0
mRNA	ID=cds100	113444-114487	12	0	0
mRNA	ID=cds108	122092-122856	12	0	0
mRNA	ID=cds1202	1273007-1274401	12	0	0
mRNA	ID=cds1211	1287005-1287847	12	0	0
mRNA	ID=cds1245	1321244-1322125	12	0	0
mRNA	ID=cds1250	1326378-1327136	12	0	0
mRNA	ID=cds1267	1342781-1344766	12	0	0
mRNA	ID=cds1418	1501741-1502889	12	0	0
mRNA	ID=cds1510	1611339-1612727	12	0	0
mRNA	ID=cds1594	1679000-1679722	12	0	0
mRNA	ID=cds1645	1733402-1733983	12	0	0
mRNA	ID=cds1656	1745155-1746759	12	0	0
mRNA	ID=cds1698	1791582-1792133	12	0	0
mRNA	ID=cds1796	1887975-1888556	12	0	0
mRNA	ID=cds1834	1923464-1924120	12	0	0
mRNA	ID=cds1841	1930139-1930780	12	0	0
mRNA	ID=cds194	221614-222645	12	0	0
mRNA	ID=cds2047	2135926-2137509	12	0	0
mRNA	ID=cds2113	2214503-2215429	12	0	0
mRNA	ID=cds2154	2261885-2263066	12	0	0
mRNA	ID=cds2334	2466236-2467156	12	0	0
mRNA	ID=cds2482	2620256-2620894	12	0	0
mRNA	ID=cds2657	2814959-2815525	12	0	0
mRNA	ID=cds2669	2827069-2827842	12	0	0
mRNA	ID=cds2679	2840595-2841065	12	0	0
mRNA	ID=cds2697	2858489-2859256	12	0	0
mRNA	ID=cds2743	2907916-2908707	12	0	0
mRNA	ID=cds2855	3040511-3041170	12	0	0
mRNA	ID=cds3009	3202716-3203333	12	0	0
mRNA	ID=cds3040	3239849-3241336	12	0	0
mRNA	ID=cds3061	3258146-3260440	12	0	0
mRNA	ID=cds3416	3615799-3616605	12	0	0
mRNA	ID=cds3704	3938658-3939350	12	0	0
mRNA	ID=cds3733	3975548-3976627	12	0	0
mRNA	ID=cds375	400971-402386	12	0	0
mRNA	ID=cds3784	4032631-4033176	12	0	0
mRNA	ID=cds3854	4113737-4115245	12	0	0
mRNA	ID=cds3923	4201343-4202668	12	0	0
mRNA	ID=cds4038	4341392-4342813	12	0	0
mRNA	ID=cds4129	4430114-4431079	12	0	0
mRNA	ID=cds4210	4517361-4518347	12	0	0
mRNA	ID=cds4289	4606669-4607346	12	0	0
mRNA	ID=cds483	517564-518373	12	0	0
mRNA	ID=cds486	519640-522054	12	0	0
mRNA	ID=cds864	925951-926655	12	0	0
mRNA	ID=cds929	1011224-1012477	12	0	0
mRNA	ID=cds3418	3617215-3621450	11.951462	0	0.5
mRNA	ID=cds2540	2693823-2695379	11.76822	0	0
mRNA	ID=cds1622	1709547-1710182	11.646447	0	0
mRNA	ID=cds1745	1837491-1838798	11.528595	0	1
mRNA	ID=cds874	942637-943254	11.5	0	0
repeat_region		3523500-3523597	11.484745	0	0
mRNA	ID=cds1426	1509678-1510823	11.483046	0	0
repeat_region		1112640-1112737	11.475956	0	0.107527
mRNA	ID=cds2957	3149272-3150006	11.424684	0	1
mRNA	ID=cds3795	4050068-4051441	11.353553	0	0
mRNA	ID=cds878	946452-947882	11.19868	0	0
mRNA	ID=cds2135	2238650-2239690	11.176777	0	1
mRNA	ID=cds393	420210-421583	11.108012	0	0
mRNA	ID=cds1889	1979611-1980411	11	0	9
mRNA	ID=cds3456	3667615-3669264	11	0	6
mRNA	ID=cds1436	1517051-1518088	11	0	2
mRNA	ID=cds1504	1605370-1606128	11	0	2
mRNA	ID=cds3744	3991762-3992082	11	0	2
mRNA	ID=cds3855	4115268-4116113	11	0	2
mRNA	ID=cds387	410521-411705	11	0	2
mRNA	ID=cds389	414974-416176	11	0	2
mRNA	ID=cds204	236067-236798	11	0	1
mRNA	ID=cds2297	2426743-2428260	11	0	1
mRNA	ID=cds2378	2511064-2512266	11	0	1
mRNA	ID=cds2415	2550374-2551243	11	0	1
mRNA	ID=cds2770	2939672-2940589	11	0	1
mRNA	ID=cds2840	3025143-3026510	11	0	1
mRNA	ID=cds2885	3072708-3073217	11	0	1
mRNA	ID=cds3095	3294431-3295006	11	0	1
mRNA	ID=cds3477	3698586-3699857	11	0	1
mRNA	ID=cds3545	3770304-3772217	11	0	1
mRNA	ID=cds3736	3978910-3980295	11	0	1
mRNA	ID=cds3841	4102995-4103693	11	0	1
mRNA	ID=cds3939	4229907-4231256	11	0	1
mRNA	ID=cds564	592551-593993	11	0	1
mRNA	ID=cds747	797809-798804	11	0	1
mRNA	ID=cds779	832293-834443	11	0	1
repeat_region		1487707-1487728	11	0	0
repeat_region		1655912-1655932	11	0	0
repeat_region		4330037-4330068	11	0	0
mRNA	ID=cds105	118733-119284	11	0	0

mRNA	ID=cds1172	1242403-1243014	11	0	0
mRNA	ID=cds1244	1321062-1321106	11	0	0
mRNA	ID=cds1416	1500481-1501149	11	0	0
mRNA	ID=cds1448	1531306-1531875	11	0	0
mRNA	ID=cds1505	1606132-1607046	11	0	0
mRNA	ID=cds1617	1704372-1704950	11	0	0
mRNA	ID=cds165	191855-192580	11	0	0
mRNA	ID=cds1695	1789331-1790044	11	0	0
mRNA	ID=cds1705	1797826-1798023	11	0	0
mRNA	ID=cds1718	1810353-1811156	11	0	0
mRNA	ID=cds1740	1832832-1833539	11	0	0
mRNA	ID=cds1746	1838807-1839427	11	0	0
mRNA	ID=cds1893	1984949-1985452	11	0	0
mRNA	ID=cds1909	1997609-1998409	11	0	0
mRNA	ID=cds192	220113-220928	11	0	0
mRNA	ID=cds193	220968-221621	11	0	0
mRNA	ID=cds2166	2275915-2276259	11	0	0
mRNA	ID=cds2170	2280539-2280823	11	0	0
mRNA	ID=cds2172	2282151-2282378	11	0	0
mRNA	ID=cds2177	2289380-2290432	11	0	0
mRNA	ID=cds2198	2308501-2309556	11	0	0
mRNA	ID=cds2233	2360453-2361655	11	0	0
mRNA	ID=cds2288	2419347-2419709	11	0	0
mRNA	ID=cds2310	2441913-2442191	11	0	0
mRNA	ID=cds2348	2474332-2474532	11	0	0
mRNA	ID=cds2506	2654558-2654758	11	0	0
mRNA	ID=cds2653	2810638-2812176	11	0	0
mRNA	ID=cds3119	3320195-3320527	11	0	0
mRNA	ID=cds3121	3322085-3322933	11	0	0
mRNA	ID=cds3289	3478629-3479183	11	0	0
mRNA	ID=cds3291	3481224-3482246	11	0	0
mRNA	ID=cds3359	3557870-3558628	11	0	0
mRNA	ID=cds3620	3850913-3851011	11	0	0
mRNA	ID=cds3629	3858306-3859199	11	0	0
mRNA	ID=cds3637	3866085-3867332	11	0	0
mRNA	ID=cds3858	4117446-4118372	11	0	0
mRNA	ID=cds3922	4199949-4201346	11	0	0
mRNA	ID=cds3928	4212303-4213232	11	0	0
mRNA	ID=cds3959	4255138-4255746	11	0	0
mRNA	ID=cds4087	4393608-4394069	11	0	0
mRNA	ID=cds4245	4555016-4555408	11	0	0
mRNA	ID=cds598	633970-634599	11	0	0
mRNA	ID=cds617	653806-655191	11	0	0
mRNA	ID=cds739	790262-791278	11	0	0
mRNA	ID=cds770	822959-823720	11	0	0
mRNA	ID=cds787	841555-842481	11	0	0
mRNA	ID=cds812	871113-872024	11	0	0
mRNA	ID=cds958	1039668-1039760	11	0	0
mRNA	ID=cds2500	2642455-2642886	10.775021	0	0
mRNA	ID=cds4263	4575981-4577360	10.521392	0	0
mRNA	ID=cds3732	3974301-3975551	10.5	0	1
mRNA	ID=cds1668	1757327-1758547	10.5	0	0
mRNA	ID=cds2652	2809449-2810621	10.5	0	0
mRNA	ID=cds4132	4432645-4434588	10.5	0	0
mRNA	ID=cds2062	2156410-2159487	10.359812	0	1
mRNA	ID=cds3780	4026805-4028994	10.166667	0	0
mRNA	ID=cds2685	2845437-2847263	10.120573	0	0
repeat_region		3734177-3734347	10.095261	0	0.223004
mRNA	ID=cds2100	2198301-2201933	10.062517	0	2
repeat_region		3010423-3010626	10.05079	0	2
repeat_region		680908-680937	10.028509	0	0
mRNA	ID=cds309	326485-327957	10.007175	0	2
mRNA	ID=cds116	135598-136464	10	0	3
mRNA	ID=cds370	397096-398190	10	0	3
mRNA	ID=cds1647	1735480-1735569	10	0	2
mRNA	ID=cds2656	2814534-2814962	10	0	2
mRNA	ID=cds2692	2851276-2852286	10	0	2
mRNA	ID=cds3897	4172099-4173049	10	0	2
mRNA	ID=cds3978	4273494-4275080	10	0	2
mRNA	ID=cds815	875933-877258	10	0	2
mgeRNA	gbkey=rep_origin	3923767-3923998	10	0	1
mRNA	ID=cds1819	1908300-1909673	10	0	1
mRNA	ID=cds1887	1977777-1978205	10	0	1
mRNA	ID=cds2239	2366061-2368043	10	0	1
mRNA	ID=cds2278	2409461-2410111	10	0	1
mRNA	ID=cds2486	2627312-2627503	10	0	1
mRNA	ID=cds2832	3012309-3013079	10	0	1
mRNA	ID=cds2843	3029389-3030837	10	0	1
mRNA	ID=cds3075	3275359-3275823	10	0	1
mRNA	ID=cds3218	3428042-3428860	10	0	1
mRNA	ID=cds3410	3609888-3610937	10	0	1
mRNA	ID=cds3411	3610992-3611579	10	0	1
mRNA	ID=cds3530	3752996-3754534	10	0	1
mRNA	ID=cds781	835574-836659	10	0	1
repeat_region		1164962-1165036	10	0	0
repeat_region		1166625-1166654	10	0	0
repeat_region		1930110-1930128	10	0	0
repeat_region		473495-473517	10	0	0
mRNA	ID=cds1248	1324876-1325751	10	0	0
mRNA	ID=cds1350	1421806-1423263	10	0	0
mRNA	ID=cds1403	1488926-1489456	10	0	0
mRNA	ID=cds1943	2026212-2026394	10	0	0
mRNA	ID=cds1986	2075964-2076131	10	0	0
mRNA	ID=cds2002	2088020-2088070	10	0	0
mRNA	ID=cds2049	2139658-2140239	10	0	0
mRNA	ID=cds2053	2145698-2147050	10	0	0
mRNA	ID=cds2143	2248862-2249719	10	0	0
mRNA	ID=cds2182	2293605-2294342	10	0	0
mRNA	ID=cds2216	2338439-2342191	10	0	0
mRNA	ID=cds2384	2519615-2520499	10	0	0
mRNA	ID=cds2535	2685092-2685430	10	0	0
mRNA	ID=cds2663	2822513-2823598	10	0	0
mRNA	ID=cds2682	2842232-2842774	10	0	0
mRNA	ID=cds2777	2947264-2948595	10	0	0
mRNA	ID=cds2914	3100155-3100874	10	0	0
mRNA	ID=cds3081	3279998-3281152	10	0	0
mRNA	ID=cds3184	3384243-3386210	10	0	0
mRNA	ID=cds3336	3526691-3527359	10	0	0
mRNA	ID=cds3362	3560036-3561541	10	0	0
mRNA	ID=cds3473	3694481-3696052	10	0	0
mRNA	ID=cds3475	3696237-3697916	10	0	0
mRNA	ID=cds3509	3733002-3734180	10	0	0
mRNA	ID=cds3565	3790849-3791706	10	0	0
mRNA	ID=cds3635	3865032-3865445	10	0	0
mRNA	ID=cds3662	3892675-3893241	10	0	0
mRNA	ID=cds3789	4041441-4042067	10	0	0
mRNA	ID=cds3850	4110990-4111289	10	0	0
mRNA	ID=cds3865	4125309-4125917	10	0	0
mRNA	ID=cds4050	4354493-4356640	10	0	0
mRNA	ID=cds417	443907-444398	10	0	0
mRNA	ID=cds4217	4525572-4526003	10	0	0
mRNA	ID=cds4256	4565310-4566542	10	0	0
mRNA	ID=cds4268	4584972-4585886	10	0	0
mRNA	ID=cds450	479314-479532	10	0	0
mRNA	ID=cds473	507442-507783	10	0	0
mRNA	ID=cds485	518957-519643	10	0	0
mRNA	ID=cds575	605488-606606	10	0	0
mRNA	ID=cds622	658170-658373	10	0	0
mRNA	ID=cds793	848433-849320	10	0	0
mRNA	ID=cds797	852406-852873	10	0	0
repeat_region		5565-5669	9.856805	0	0.146394
mgeRNA	gbkey=mobile_element	1976527-1977294	9.823834	0	0.666668
mgeRNA	gbkey=mobile_element	19796-20563	9.823834	0	0.666668
mgeRNA	gbkey=mobile_element	3581451-3582218	9.823834	0	0.666668
repeat_region		3960723-3960758	9.677725	0	0
mRNA	ID=cds4069	4374898-4375215	9.646447	0	1
repeat_region		4458438-4458521	9.526735	0	0.047004
mRNA	ID=cds3719	3958700-3960721	9.5	0	1

mRNA	ID=cds3433	3640403-3641155	9.5	0	0
mRNA	ID=cds3970	4265137-4266330	9.390095	0	0
mRNA	ID=cds489	527176-527883	9.284612	0	0
repeat_region		2579670-2579738	9.09407	0	1
mRNA	ID=cds1239	1314440-1315246	9.017572	0	0
mRNA	ID=cds4298	4616252-4617574	9.017551	0	0
repeat_region		550577-550733	9.017017	0	0.079063
mRNA	ID=cds4070	4375212-4375745	9	0	7
mRNA	ID=cds2592	2748853-2749731	9	0	3
mRNA	ID=cds826	886646-887182	9	0	3
mRNA	ID=cds3843	4104492-4105394	9	0	2
mRNA	ID=cds721	773419-773532	9	0	2
mRNA	ID=cds1364	1439082-1439348	9	0	1
mRNA	ID=cds145	167484-169727	9	0	1
mRNA	ID=cds1805	1896451-1898049	9	0	1
mRNA	ID=cds1916	2004180-2005667	9	0	1
mRNA	ID=cds2374	2506483-2507448	9	0	1
mRNA	ID=cds2580	2739382-2739747	9	0	1
mRNA	ID=cds2715	2874603-2875640	9	0	1
mRNA	ID=cds2908	3094703-3095296	9	0	1
mRNA	ID=cds2958	3150258-3151445	9	0	1
mRNA	ID=cds3262	3454399-3456351	9	0	1
mRNA	ID=cds3339	3528737-3530461	9	0	1
mRNA	ID=cds3406	3606774-3607019	9	0	1
mRNA	ID=cds3493	3718072-3718284	9	0	1
mRNA	ID=cds3882	4146555-4148288	9	0	1
mRNA	ID=cds4239	4546831-4547733	9	0	1
mRNA	ID=cds4243	4552599-4553372	9	0	1
mRNA	ID=cds4304	4622918-4623886	9	0	1
mRNA	ID=cds634	669154-669795	9	0	1
mRNA	ID=cds80	91032-91397	9	0	1
mRNA	ID=cds931	1014119-1014682	9	0	1
repeat_region		1386296-1386320	9	0	0
repeat_region		2553214-2553237	9	0	0
repeat_region		2715480-2715505	9	0	0
repeat_region		3678390-3678465	9	0	0
repeat_region		4130370-4130403	9	0	0
mRNA	ID=cds1043	1125380-1126027	9	0	0
mRNA	ID=cds1087	1167423-1168055	9	0	0
mRNA	ID=cds1107	1189839-1191209	9	0	0
mRNA	ID=cds118	137083-138633	9	0	0
mRNA	ID=cds127	146968-147870	9	0	0
mRNA	ID=cds1384	1460149-1461462	9	0	0
mRNA	ID=cds139	159186-160112	9	0	0
mRNA	ID=cds1502	1604124-1604999	9	0	0
mRNA	ID=cds1507	1608931-1609878	9	0	0
mRNA	ID=cds1661	1749752-1751854	9	0	0
mRNA	ID=cds1744	1836771-1837424	9	0	0
mRNA	ID=cds1781	1875302-1875556	9	0	0
mRNA	ID=cds1789	1878910-1879833	9	0	0
mRNA	ID=cds1832	1922619-1922993	9	0	0
mRNA	ID=cds187	215269-215979	9	0	0
mRNA	ID=cds1900	1988978-1989643	9	0	0
mRNA	ID=cds1946	2028472-2028942	9	0	0
mRNA	ID=cds1989	2077056-2077385	9	0	0
mRNA	ID=cds2063	2159488-2160903	9	0	0
mRNA	ID=cds2176	2288522-2289169	9	0	0
mRNA	ID=cds2191	2301025-2301519	9	0	0
mRNA	ID=cds2363	2493667-2494587	9	0	0
mRNA	ID=cds2446	2579756-2580799	9	0	0
mRNA	ID=cds2453	2589629-2590756	9	0	0
mRNA	ID=cds253	275939-276871	9	0	0
mRNA	ID=cds2566	2717245-2717943	9	0	0
mRNA	ID=cds2654	2812240-2812755	9	0	0
mRNA	ID=cds270	290628-291455	9	0	0
mRNA	ID=cds2745	2909113-2909361	9	0	0
mRNA	ID=cds2750	2917428-2918768	9	0	0
mRNA	ID=cds2790	2967684-2968373	9	0	0
mRNA	ID=cds2838	3022373-3023773	9	0	0
mRNA	ID=cds2913	3099829-3100155	9	0	0
mRNA	ID=cds2916	3102115-3102390	9	0	0
mRNA	ID=cds2922	3109150-3110112	9	0	0
mRNA	ID=cds2942	3136749-3137615	9	0	0
mRNA	ID=cds2960	3152284-3153240	9	0	0
mRNA	ID=cds2975	3168506-3169855	9	0	0
mRNA	ID=cds2998	3190230-3190859	9	0	0
mRNA	ID=cds3021	3215578-3217098	9	0	0
mRNA	ID=cds3039	3239215-3239766	9	0	0
mRNA	ID=cds3091	3290497-3291357	9	0	0
mRNA	ID=cds3118	3318010-3319635	9	0	0
mRNA	ID=cds3219	3428865-3429437	9	0	0
mRNA	ID=cds3236	3440640-3440756	9	0	0
mRNA	ID=cds3273	3464271-3464747	9	0	0
mRNA	ID=cds3350	3542904-3543587	9	0	0
mRNA	ID=cds3412	3611690-3613264	9	0	0
mRNA	ID=cds341	366811-367644	9	0	0
mRNA	ID=cds3501	3723910-3724905	9	0	0
mRNA	ID=cds3557	3782214-3782465	9	0	0
mRNA	ID=cds3607	3836271-3837194	9	0	0
mRNA	ID=cds3613	3841987-3843753	9	0	0
mRNA	ID=cds3643	3872494-3873183	9	0	0
mRNA	ID=cds3731	3973169-3974299	9	0	0
mRNA	ID=cds3735	3977979-3978719	9	0	0
mRNA	ID=cds3757	4002885-4003754	9	0	0
mRNA	ID=cds3779	4025632-4026795	9	0	0
mRNA	ID=cds3817	4075472-4076461	9	0	0
mRNA	ID=cds4024	4323321-4323764	9	0	0
mRNA	ID=cds4107	4414040-4414315	9	0	0
mRNA	ID=cds4126	4426958-4427578	9	0	0
mRNA	ID=cds4138	4439561-4440199	9	0	0
mRNA	ID=cds4161	4468550-4468936	9	0	0
mRNA	ID=cds4235	4542327-4543052	9	0	0
mRNA	ID=cds4250	4558953-4559507	9	0	0
mRNA	ID=cds448	478005-478475	9	0	0
mRNA	ID=cds64	72911-74521	9	0	0
mRNA	ID=cds654	691097-691564	9	0	0
mRNA	ID=cds69	78848-79453	9	0	0
mRNA	ID=cds784	837753-838430	9	0	0
mRNA	ID=cds841	900089-900757	9	0	0
repeat_region		3099718-3099796	8.863903	0	0
repeat_region		4264911-4265007	8.823223	0	0
mRNA	ID=cds443	473525-474385	8.696132	0	1
repeat_region		2116490-2116675	8.672342	0	0.170883
repeat_region		39151-39232	8.634018	0	0.041219
repeat_region		1067577-1067674	8.573307	0	0.091615
mRNA	ID=cds4119	4423141-4423536	8.566987	0	2
mRNA	ID=cds438	467607-468065	8.551978	0	1
mRNA	ID=cds340	365652-366734	8.5	0	6
mRNA	ID=cds2917	3102455-3103534	8.5	0	0
mRNA	ID=cds3885	4153024-4154028	8.5	0	0
mRNA	ID=cds1885	1976542-1977045	8.246218	0	0.500001
mRNA	ID=cds19	19811-20314	8.246218	0	0.500001
mRNA	ID=cds3380	3581700-3582203	8.246218	0	0.500001
mRNA	ID=cds1299	1375908-1378175	8.235702	0	0
mRNA	ID=cds858	918458-919573	8.195047	0	0
repeat_region		2652992-2653088	8.188838	0	0.047004
mRNA	ID=cds895	968612-969844	8.131556	0	0
repeat_region		695530-695600	8.081786	0	0
repeat_region		4054494-4054578	8.01211	0	1
mRNA	ID=cds4109	4414975-4415724	8.004905	0	0
mRNA	ID=cds2971	3166270-3166566	8	0	3
mRNA	ID=cds3578	3803966-3805090	8	0	3
mRNA	ID=cds156	180884-182308	8	0	2
mRNA	ID=cds2505	2653097-2654380	8	0	2
mRNA	ID=cds3558	3782607-3783038	8	0	2
mRNA	ID=cds3594	3817511-3819193	8	0	2
mRNA	ID=cds391	417113-418408	8	0	2
mRNA	ID=cds469	502700-503920	8	0	2

mRNA	ID=cds1024	1107007-1108164	8	0	1
mRNA	ID=cds1118	1200720-1201061	8	0	1
mRNA	ID=cds1192	1266147-1266539	8	0	1
mRNA	ID=cds1518	1618262-1619161	8	0	1
mRNA	ID=cds1669	1758544-1759815	8	0	1
mRNA	ID=cds1898	1987275-1987514	8	0	1
mRNA	ID=cds1948	2030408-2031103	8	0	1
mRNA	ID=cds1969	2061412-2062491	8	0	1
mRNA	ID=cds2019	2104084-2105250	8	0	1
mRNA	ID=cds2469	2606509-2608176	8	0	1
mRNA	ID=cds305	321562-322989	8	0	1
mRNA	ID=cds3117	3316659-3318002	8	0	1
mRNA	ID=cds3435	3643408-3644250	8	0	1
mRNA	ID=cds3513	3739132-3739605	8	0	1
mRNA	ID=cds718	767201-769834	8	0	1
mRNA	ID=cds902	974845-975549	8	0	1
mRNA	ID=cds925	1005175-1005717	8	0	1
mRNA	ID=cds4186	4494698-4495963	8	0	0.5
mRNA	ID=cds2331	2463323-2464255	8	0	0.41833
repeat_region		3813120-3813141	8	0	0
repeat_region		497140-497157	8	0	0
mgeRNA	gbkey=mobile_element	3718656-3720098	8	0	0
other ncRNA		1435145-1435253	8	0	0
mRNA	ID=cds1010	1097109-1098047	8	0	0
mRNA	ID=cds1011	1098102-1098839	8	0	0
mRNA	ID=cds1012	1098863-1099417	8	0	0
mRNA	ID=cds1135	1209569-1210402	8	0	0
mRNA	ID=cds1145	1216551-1218074	8	0	0
mRNA	ID=cds1205	1277180-1278571	8	0	0
mRNA	ID=cds1228	1306812-1306985	8	0	0
mRNA	ID=cds1328	1407535-1408908	8	0	0
mRNA	ID=cds1366	1439878-1440867	8	0	0
mRNA	ID=cds137	157253-157732	8	0	0
mRNA	ID=cds1392	1472245-1473105	8	0	0
mRNA	ID=cds1415	1499586-1500179	8	0	0
mRNA	ID=cds1566	1649575-1650732	8	0	0
mRNA	ID=cds1662	1751875-1752501	8	0	0
mRNA	ID=cds1670	1759790-1760536	8	0	0
mRNA	ID=cds1737	1830452-1831258	8	0	0
mRNA	ID=cds1755	1848884-1849900	8	0	0
mRNA	ID=cds1799	1891391-1891735	8	0	0
mRNA	ID=cds1859	1948856-1949422	8	0	0
mRNA	ID=cds1867	1957304-1957870	8	0	0
mRNA	ID=cds1976	2068294-2068498	8	0	0
mRNA	ID=cds2016	2101415-2102533	8	0	0
mRNA	ID=cds2017	2102518-2103108	8	0	0
mRNA	ID=cds2054	2147063-2149009	8	0	0
mRNA	ID=cds2155	2263063-2263317	8	0	0
mRNA	ID=cds2243	2370914-2371300	8	0	0
mRNA	ID=cds2255	2382017-2383744	8	0	0
mRNA	ID=cds2388	2523952-2524878	8	0	0
mRNA	ID=cds2391	2526183-2528198	8	0	0
mRNA	ID=cds2504	2652179-2652955	8	0	0
mRNA	ID=cds2650	2808366-2808701	8	0	0
mRNA	ID=cds265	287628-288386	8	0	0
mRNA	ID=cds2798	2977043-2977978	8	0	0
mRNA	ID=cds2842	3027034-3028953	8	0	0
mRNA	ID=cds2880	3066969-3067829	8	0	0
mRNA	ID=cds2988	3180572-3181345	8	0	0
mRNA	ID=cds3054	3251340-3252236	8	0	0
mRNA	ID=cds3070	3269889-3270773	8	0	0
mRNA	ID=cds310	327971-328558	8	0	0
mRNA	ID=cds3461	3674313-3676373	8	0	0
mRNA	ID=cds3552	3777850-3779040	8	0	0
mRNA	ID=cds3572	3798290-3798988	8	0	0
mRNA	ID=cds3709	3948530-3948580	8	0	0
mRNA	ID=cds3717	3957555-3957836	8	0	0
mRNA	ID=cds3864	4125036-4125248	8	0	0
mRNA	ID=cds3867	4126695-4127855	8	0	0
mRNA	ID=cds3955	4250529-4251026	8	0	0
mRNA	ID=cds4234	4541751-4542290	8	0	0
mRNA	ID=cds4251	4559520-4560698	8	0	0
mRNA	ID=cds4313	4633544-4634017	8	0	0
mRNA	ID=cds481	515807-516586	8	0	0
mRNA	ID=cds511	550750-551817	8	0	0
mRNA	ID=cds716	764376-765098	8	0	0
mRNA	ID=cds728	778290-778811	8	0	0
mRNA	ID=cds763	817793-818278	8	0	0
mRNA	ID=cds806	863603-864352	8	0	0
mRNA	ID=cds810	868634-870172	8	0	0
mRNA	ID=cds889	960424-961107	8	0	0
mRNA	ID=cds940	1023125-1023571	8	0	0
mRNA	ID=cds976	1056485-1057177	8	0	0
mRNA	ID=cds994	1073465-1074103	8	0	0
repeat_region		4342838-4342932	7.676216	0	0
mRNA	ID=cds3491	3716357-3717067	7.668705	0	2
repeat_region		3080712-3080858	7.60283	0	2.109727
mRNA	ID=cds686	728357-728563	7.559041	0	0
mRNA	ID=cds1729	1821539-1822426	7.5	0	0
mRNA	ID=cds2417	2552152-2553204	7.5	0	0
mRNA	ID=cds3386	3586133-3587203	7.5	0	0
mRNA	ID=cds741	793079-793867	7.5	0	0
mRNA	ID=cds1427	1510841-1511854	7.42515	0	1
mRNA	ID=cds3878	4142018-4144315	7.408248	0	0
mRNA	ID=cds2909	3095289-3096425	7.288675	0	0
other ncRNA		3851141-3851280	7.246808	0	0
mRNA	ID=cds2292	2422539-2423255	7.152372	0	0
repeat_region		4092339-4092740	7.064975	0	0.182128
mRNA	ID=cds1952	2034818-2036176	7.049556	0	0
mRNA	ID=cds2420	2554432-2555319	7.029769	0	0
mRNA	ID=cds3358	3556290-3557888	7.004626	0	1
mRNA	ID=cds3437	3645728-3645856	7	0	6
mRNA	ID=cds3275	3465182-3467875	7	0	5
mRNA	ID=cds1689	1781055-1782701	7	0	3
mRNA	ID=cds31	30817-34038	7	0	2
repeat_region		3588956-3589021	7	0	1
mRNA	ID=cds1022	1105043-1105576	7	0	1
mRNA	ID=cds1063	1145234-1145818	7	0	1
mRNA	ID=cds1072	1152523-1153332	7	0	1
mRNA	ID=cds114	134388-134750	7	0	1
mRNA	ID=cds1236	1312742-1313248	7	0	1
mRNA	ID=cds1291	1367713-1368027	7	0	1
mRNA	ID=cds1353	1424478-1425410	7	0	1
mRNA	ID=cds1572	1654771-1655481	7	0	1
mRNA	ID=cds1790	1879936-1881021	7	0	1
mRNA	ID=cds1837	1927072-1927731	7	0	1
mRNA	ID=cds2001	2087486-2087737	7	0	1
mRNA	ID=cds2081	2175534-2176586	7	0	1
mRNA	ID=cds2753	2921024-2921806	7	0	1
mRNA	ID=cds3022	3217516-3218895	7	0	1
mRNA	ID=cds3913	4192227-4194122	7	0	1
mRNA	ID=cds4153	4457513-4457878	7	0	1
mRNA	ID=cds4287	4605826-4606239	7	0	1
mRNA	ID=cds559	583903-584856	7	0	1
mRNA	ID=cds703	747144-748205	7	0	1
mRNA	ID=cds4160	4465648-4468344	7	0	0.030907
repeat_region		2163041-2163120	7	0	0
repeat_region		2929839-2929868	7	0	0
repeat_region		4585895-4585921	7	0	0
repeat_region		710131-710163	7	0	0
mgeRNA	gbkey=misc_feature	2165222-2165850	7	0	0
other ncRNA		1647406-1647458	7	0	0
other ncRNA		2651877-2652180	7	0	0
mRNA	ID=cds1031	1116030-1117082	7	0	0
mRNA	ID=cds1046	1128637-1129053	7	0	0
mRNA	ID=cds1081	1161861-1162502	7	0	0
mRNA	ID=cds1140	1213487-1214698	7	0	0
mRNA	ID=cds1179	1248991-1250061	7	0	0

mRNA	ID=cds122	142779-143705	7	0	0
mRNA	ID=cds1262	1339945-1340682	7	0	0
mRNA	ID=cds1266	1342460-1342633	7	0	0
mRNA	ID=cds1370	1444402-1445307	7	0	0
mRNA	ID=cds1438	1518987-1521089	7	0	0
mRNA	ID=cds1501	1603075-1604097	7	0	0
mRNA	ID=cds1613	1702575-1702700	7	0	0
mRNA	ID=cds1641	1726371-1727018	7	0	0
mRNA	ID=cds1684	1776414-1777325	7	0	0
mRNA	ID=cds1697	1790833-1791582	7	0	0
mRNA	ID=cds1774	1870065-1871555	7	0	0
mRNA	ID=cds1780	1874933-1875280	7	0	0
mRNA	ID=cds1839	1928481-1928771	7	0	0
mRNA	ID=cds1930	2016410-2017537	7	0	0
mRNA	ID=cds1992	2079405-2080571	7	0	0
mRNA	ID=cds199	231926-232549	7	0	0
mRNA	ID=cds203	235535-236002	7	0	0
mRNA	ID=cds2052	2144716-2145564	7	0	0
mRNA	ID=cds2064	2160900-2162303	7	0	0
mRNA	ID=cds2156	2263472-2264044	7	0	0
mRNA	ID=cds2213	2332978-2334666	7	0	0
mRNA	ID=cds2293	2423252-2423938	7	0	0
mRNA	ID=cds2294	2424028-2424810	7	0	0
mRNA	ID=cds2412	2548663-2549238	7	0	0
mRNA	ID=cds2418	2553250-2553750	7	0	0
mRNA	ID=cds2447	2580925-2581500	7	0	0
mRNA	ID=cds2744	2908778-2909113	7	0	0
mRNA	ID=cds2756	2923370-2924218	7	0	0
mRNA	ID=cds282	302215-302829	7	0	0
mRNA	ID=cds3024	3219488-3220471	7	0	0
mRNA	ID=cds3169	3370705-3371598	7	0	0
mRNA	ID=cds3185	3386216-3387148	7	0	0
mRNA	ID=cds321	342108-343157	7	0	0
mRNA	ID=cds3293	3482512-3483381	7	0	0
mRNA	ID=cds3360	3558645-3559475	7	0	0
mRNA	ID=cds3404	3603774-3604400	7	0	0
mRNA	ID=cds3497	3719221-3720072	7	0	0
mRNA	ID=cds349	375996-376535	7	0	0
mRNA	ID=cds3508	3731743-3732924	7	0	0
mRNA	ID=cds3646	3875090-3875488	7	0	0
mRNA	ID=cds3714	3953354-3954898	7	0	0
mRNA	ID=cds3759	4005780-4006400	7	0	0
mRNA	ID=cds3798	4053313-4054362	7	0	0
mRNA	ID=cds3869	4130639-4131529	7	0	0
mRNA	ID=cds390	416366-417055	7	0	0
mRNA	ID=cds3925	4203966-4205555	7	0	0
mRNA	ID=cds3993	4291566-4292162	7	0	0
mRNA	ID=cds4063	4370832-4371185	7	0	0
mRNA	ID=cds4133	4434778-4435518	7	0	0
mRNA	ID=cds4158	4464322-4465269	7	0	0
mRNA	ID=cds4172	4477057-4477560	7	0	0
mRNA	ID=cds458	490106-490483	7	0	0
mRNA	ID=cds53	57364-58179	7	0	0
mRNA	ID=cds600	635939-636841	7	0	0
mRNA	ID=cds631	667471-667938	7	0	0
mRNA	ID=cds808	865791-866756	7	0	0
mRNA	ID=cds813	872202-874550	7	0	0
mRNA	ID=cds831	890407-891129	7	0	0
mRNA	ID=cds979	1061022-1061621	7	0	0
repeat_region		3253239-3253341	6.95498	0	0
repeat_region		831512-831609	6.878096	0	0.657238
mRNA	ID=cds4030	4330204-4331295	6.657337	0	0
mRNA	ID=cds72	81958-83529	6.612702	0	0
mgeRNA	gbkey=mobile_element	4516495-4517262	6.606935	0	1
mRNA	ID=cds1811	1903712-1904278	6.55033	0	3.5
mRNA	ID=cds2099	2194496-2197342	6.5	0	0
mRNA	ID=cds987	1067734-1069062	6.5	0	0
repeat_region		3738987-3739084	6.427597	0	0.012075
repeat_region		3328482-3328579	6.426302	0	0.047004
mRNA	ID=cds3210	3413055-3416159	6.369306	0	0
mRNA	ID=cds1722	1815172-1816524	6.353553	0	0
mRNA	ID=cds3730	3972490-3973164	6.288675	0	0
repeat_region		3596420-3596518	6.279645	0	0.047004
mRNA	ID=cds2126	2229041-2229736	6.276112	0	0
mRNA	ID=cds2671	2828797-2830311	6.247797	0	0
mRNA	ID=cds3630	3859196-3860010	6.235702	0	3
mRNA	ID=cds134	152829-155426	6.235702	0	2
repeat_region		3906410-3906473	6.156633	0	0.424024
repeat_region		4547806-4547841	6.154751	0	0
repeat_region		1506815-1506847	6.145907	0	0
repeat_region		408276-408312	6.143021	0	0
repeat_region		4567969-4568067	6.031283	0	0
repeat_region		785958-785992	6.008288	0	0
mRNA	ID=cds2761	2929887-2931035	6	0	3
mRNA	ID=cds2006	2091492-2092559	6	0	2
mRNA	ID=cds2088	2182535-2183323	6	0	2
mRNA	ID=cds3042	3243126-3244544	6	0	2
mRNA	ID=cds3543	3768266-3769402	6	0	2
mRNA	ID=cds694	738224-738733	6	0	2
mRNA	ID=cds1000	1084215-1085279	6	0	1
mRNA	ID=cds1428	1511855-1512796	6	0	1
mRNA	ID=cds1728	1820482-1821309	6	0	1
mRNA	ID=cds1827	1919804-1920040	6	0	1
mRNA	ID=cds2060	2152040-2153287	6	0	1
mRNA	ID=cds2138	2241932-2242768	6	0	1
mRNA	ID=cds2241	2368930-2370582	6	0	1
mRNA	ID=cds2369	2500012-2502507	6	0	1
mRNA	ID=cds2578	2736970-2738091	6	0	1
mRNA	ID=cds266	288525-289529	6	0	1
mRNA	ID=cds2791	2968442-2969155	6	0	1
mRNA	ID=cds279	297960-300158	6	0	1
mRNA	ID=cds2954	3146999-3147493	6	0	1
mRNA	ID=cds3051	3249046-3250032	6	0	1
mRNA	ID=cds3125	3326261-3326737	6	0	1
mRNA	ID=cds3413	3613264-3614208	6	0	1
mRNA	ID=cds3776	4022356-4022844	6	0	1
mRNA	ID=cds3793	4048156-4048788	6	0	1
mRNA	ID=cds3824	4084039-4084872	6	0	1
mRNA	ID=cds4083	4389627-4390172	6	0	1
mRNA	ID=cds4314	4634030-4634719	6	0	1
mRNA	ID=cds778	831691-832173	6	0	1
mRNA	ID=cds4309	4630783-4631109	6	0	0.5
mRNA	ID=cds4032	4331970-4333613	6	0	0.158968
repeat_region		2167674-2167707	6	0	0
repeat_region		3091988-3092112	6	0	0
repeat_region		442242-442273	6	0	0
repeat_region		791487-791520	6	0	0
mgeRNA	gbkey=mobile_element	269430-269764	6	0	0
mRNA	ID=cds1004	1089089-1091512	6	0	0
mRNA	ID=cds1177	1246919-1248337	6	0	0
mRNA	ID=cds1230	1308593-1308889	6	0	0
mRNA	ID=cds1320	1397745-1398260	6	0	0
mRNA	ID=cds1388	1463416-1465928	6	0	0
mRNA	ID=cds1430	1513602-1515026	6	0	0
mRNA	ID=cds1449	1532048-1532893	6	0	0
mRNA	ID=cds1477	1565528-1566847	6	0	0
mRNA	ID=cds1480	1570431-1573226	6	0	0
mRNA	ID=cds1514	1616267-1616932	6	0	0
mRNA	ID=cds1547	1640513-1641265	6	0	0
mRNA	ID=cds1595	1679719-1680054	6	0	0
mRNA	ID=cds159	184257-185069	6	0	0
mRNA	ID=cds1621	1708852-1709547	6	0	0
mRNA	ID=cds1743	1835236-1836771	6	0	0
mRNA	ID=cds1768	1861874-1862758	6	0	0
mRNA	ID=cds1778	1872779-1873600	6	0	0
mRNA	ID=cds1945	2027563-2028483	6	0	0
mRNA	ID=cds1950	2032075-2032737	6	0	0
mRNA	ID=cds1971	2063243-2063788	6	0	0

mRNA	ID=cds197	229967-230881	6	0	0
mRNA	ID=cds2050	2140331-2140972	6	0	0
mRNA	ID=cds2111	2213679-2213786	6	0	0
mRNA	ID=cds2129	2231622-2231861	6	0	0
mRNA	ID=cds2197	2307363-2308427	6	0	0
mRNA	ID=cds2219	2346536-2346790	6	0	0
mRNA	ID=cds2252	2379104-2379565	6	0	0
mRNA	ID=cds2253	2379630-2380547	6	0	0
mRNA	ID=cds228	255977-256435	6	0	0
mRNA	ID=cds2295	2425031-2425813	6	0	0
mRNA	ID=cds2362	2493072-2493314	6	0	0
mRNA	ID=cds2398	2534408-2535259	6	0	0
mRNA	ID=cds2473	2609922-2611934	6	0	0
mRNA	ID=cds248	269502-269759	6	0	0
mRNA	ID=cds2519	2666028-2666918	6	0	0
mRNA	ID=cds2524	2670069-2671271	6	0	0
mRNA	ID=cds2544	2697685-2697945	6	0	0
mRNA	ID=cds261	281502-282410	6	0	0
mRNA	ID=cds2628	2788004-2789272	6	0	0
mRNA	ID=cds2629	2789295-2790743	6	0	0
mRNA	ID=cds26	25826-26275	6	0	0
mRNA	ID=cds2717	2876877-2877794	6	0	0
mRNA	ID=cds2729	2891941-2892201	6	0	0
mRNA	ID=cds2730	2892218-2892793	6	0	0
mRNA	ID=cds2763	2932257-2933573	6	0	0
mRNA	ID=cds2764	2933606-2935381	6	0	0
mRNA	ID=cds2769	2939258-2939653	6	0	0
mRNA	ID=cds2799	2977965-2978657	6	0	0
mRNA	ID=cds2800	2978786-2980204	6	0	0
mRNA	ID=cds2849	3036134-3036844	6	0	0
mRNA	ID=cds2955	3147684-3148568	6	0	0
mRNA	ID=cds2987	3179641-3180429	6	0	0
mRNA	ID=cds3020	3214801-3215424	6	0	0
mRNA	ID=cds3025	3220655-3223747	6	0	0
mRNA	ID=cds32	34300-34695	6	0	0
mRNA	ID=cds3343	3534834-3535310	6	0	0
mRNA	ID=cds3349	3542096-3542866	6	0	0
mRNA	ID=cds3417	3616611-3617012	6	0	0
mRNA	ID=cds3568	3794002-3794961	6	0	0
mRNA	ID=cds3601	3826968-3828359	6	0	0
mRNA	ID=cds3611	3839973-3840425	6	0	0
mRNA	ID=cds3675	3906572-3907462	6	0	0
mRNA	ID=cds384	407833-408174	6	0	0
mRNA	ID=cds3875	4137743-4140244	6	0	0
mRNA	ID=cds3881	4145489-4146340	6	0	0
mRNA	ID=cds3946	4238348-4238758	6	0	0
mRNA	ID=cds4025	4324422-4324757	6	0	0
mRNA	ID=cds4039	4342952-4343581	6	0	0
mRNA	ID=cds4052	4358419-4359957	6	0	0
mRNA	ID=cds4141	4445917-4446258	6	0	0
mRNA	ID=cds4211	4518694-4520043	6	0	0
mRNA	ID=cds4240	4547976-4549319	6	0	0
mRNA	ID=cds4272	4589680-4591335	6	0	0
mRNA	ID=cds4279	4599001-4599540	6	0	0
mRNA	ID=cds513	552441-553163	6	0	0
mRNA	ID=cds525	564038-565201	6	0	0
mRNA	ID=cds572	603994-604647	6	0	0
mRNA	ID=cds597	632809-633969	6	0	0
mRNA	ID=cds604	640662-641090	6	0	0
mRNA	ID=cds606	642780-643190	6	0	0
mRNA	ID=cds633	668519-669130	6	0	0
mRNA	ID=cds744	795085-795774	6	0	0
mRNA	ID=cds821	882015-882611	6	0	0
mRNA	ID=cds839	897741-898868	6	0	0
mRNA	ID=cds853	911385-913037	6	0	0
mRNA	ID=cds876	944154-944780	6	0	0
mRNA	ID=cds3441	3651254-3651736	5.755074	0	0
repeat_region		4371220-4371318	5.708886	0	0.047004
repeat_region		2280909-2280942	5.656755	0	0
mRNA	ID=cds3581	3807848-3808327	5.646447	0	0
mRNA	ID=cds3478	3699887-3700891	5.53401	0	0
repeat_region		818981-819007	5.515255	0	0
mRNA	ID=cds2867	3054263-3054811	5.5	0	0
mRNA	ID=cds2956	3148840-3149265	5.5	0	0
mRNA	ID=cds4252	4560766-4561626	5.5	0	0
mRNA	ID=cds3356	3553855-3554871	5.353553	0	0
mRNA	ID=cds3644	3873461-3874117	5.333333	0	0
mRNA	ID=cds1620	1708228-1708848	5.303488	0	0
mRNA	ID=cds4008	4311373-4311822	5.25	0	0
mRNA	ID=cds3911	4190844-4191599	5.188982	0	1
mRNA	ID=cds1419	1502929-1504104	5.176777	0	2
repeat_region		4243115-4243240	5.125428	0	0.667832
repeat_region		2915910-2916004	5.107198	0	0
mRNA	ID=cds3659	3889638-3890813	5.057408	0	0
repeat_region		3908466-3908500	5.015283	0	0
mRNA	ID=cds2193	2301927-2302415	5	0	3
mRNA	ID=cds2856	3041334-3041645	5	0	3
mRNA	ID=cds45	47769-49631	5	0	3
mRNA	ID=cds1044	1126029-1126952	5	0	1
mRNA	ID=cds1198	1269972-1271072	5	0	1
mRNA	ID=cds1201	1272469-1272822	5	0	1
mRNA	ID=cds1638	1724047-1724646	5	0	1
mRNA	ID=cds1800	1892157-1892456	5	0	1
mRNA	ID=cds2119	2223066-2223653	5	0	1
mRNA	ID=cds216	246242-246502	5	0	1
mRNA	ID=cds2332	2464567-2465724	5	0	1
mRNA	ID=cds2471	2608728-2609486	5	0	1
mRNA	ID=cds2598	2754181-2755422	5	0	1
mRNA	ID=cds2711	2871036-2871359	5	0	1
mRNA	ID=cds2823	3000636-3001514	5	0	1
mRNA	ID=cds2930	3122258-3123481	5	0	1
mRNA	ID=cds3274	3464819-3465013	5	0	1
mRNA	ID=cds364	392194-393642	5	0	1
mRNA	ID=cds3907	4187809-4188348	5	0	1
mRNA	ID=cds4049	4352977-4354434	5	0	1
repeat_region		348829-349193	5	0	0.286516
repeat_region		3058682-3058702	5	0	0
repeat_region		3231721-3231741	5	0	0
repeat_region		346048-346077	5	0	0
repeat_region		3896654-3896679	5	0	0
other ncRNA		1145812-1145980	5	0	0
mRNA	ID=cds1005	1092099-1093457	5	0	0
mRNA	ID=cds1019	1103670-1104125	5	0	0
mRNA	ID=cds1061	1143725-1144045	5	0	0
mRNA	ID=cds1111	1193521-1194174	5	0	0
mRNA	ID=cds112	129407-131260	5	0	0
mRNA	ID=cds1147	1218824-1221471	5	0	0
mRNA	ID=cds1153	1223502-1223768	5	0	0
mRNA	ID=cds1160	1228038-1228499	5	0	0
mRNA	ID=cds1176	1244902-1246599	5	0	0
mRNA	ID=cds1252	1328441-1328692	5	0	0
mRNA	ID=cds1255	1333184-1333312	5	0	0
mRNA	ID=cds1310	1388749-1388886	5	0	0
mRNA	ID=cds1335	1412810-1415410	5	0	0
mRNA	ID=cds1383	1458917-1460122	5	0	0
mRNA	ID=cds1424	1507532-1507948	5	0	0
mRNA	ID=cds147	170575-171465	5	0	0
mRNA	ID=cds155	179237-180754	5	0	0
mRNA	ID=cds1576	1661014-1661631	5	0	0
mRNA	ID=cds1635	1722158-1722679	5	0	0
mRNA	ID=cds1658	1747587-1748372	5	0	0
mRNA	ID=cds1730	1822386-1822961	5	0	0
mRNA	ID=cds2158	2265851-2266837	5	0	0
mRNA	ID=cds2180	2292923-2293402	5	0	0
mRNA	ID=cds2188	2297587-2298282	5	0	0
mRNA	ID=cds2279	2410122-2410616	5	0	0
mRNA	ID=cds2296	2426079-2426648	5	0	0
mRNA	ID=cds2400	2535771-2536505	5	0	0

mRNA	ID=cds2402	2537739-2538836	5	0	0
mRNA	ID=cds2448	2581568-2583547	5	0	0
mRNA	ID=cds246	267321-268184	5	0	0
mRNA	ID=cds2541	2695376-2695879	5	0	0
mRNA	ID=cds2583	2741647-2742129	5	0	0
mRNA	ID=cds2595	2752030-2752320	5	0	0
mRNA	ID=cds2602	2758569-2759195	5	0	0
mRNA	ID=cds2673	2831934-2833067	5	0	0
mRNA	ID=cds2675	2835600-2836127	5	0	0
mRNA	ID=cds2684	2844511-2845434	5	0	0
mRNA	ID=cds2752	2920557-2921006	5	0	0
mRNA	ID=cds2760	2929077-2929832	5	0	0
mRNA	ID=cds278	296994-297950	5	0	0
mRNA	ID=cds2873	3058872-3061016	5	0	0
mRNA	ID=cds2874	3061009-3062004	5	0	0
mRNA	ID=cds2897	3086306-3087700	5	0	0
mRNA	ID=cds2920	3107575-3108282	5	0	0
mRNA	ID=cds2990	3182862-3183152	5	0	0
mRNA	ID=cds29	28374-29195	5	0	0
mRNA	ID=cds3041	3241351-3242763	5	0	0
mRNA	ID=cds3090	3289363-3290454	5	0	0
mRNA	ID=cds3113	3310799-3311200	5	0	0
mRNA	ID=cds3132	3332931-3333209	5	0	0
mRNA	ID=cds3161	3363207-3363573	5	0	0
mRNA	ID=cds3270	3461944-3463107	5	0	0
mRNA	ID=cds3320	3509461-3510366	5	0	0
mRNA	ID=cds332	355395-356678	5	0	0
mRNA	ID=cds3395	3596007-3596390	5	0	0
mRNA	ID=cds3460	3672809-3674131	5	0	0
mRNA	ID=cds3471	3693256-3694008	5	0	0
mRNA	ID=cds3492	3717501-3717791	5	0	0
mRNA	ID=cds3505	3727466-3728788	5	0	0
mRNA	ID=cds3526	3749151-3749891	5	0	0
mRNA	ID=cds3570	3796262-3797335	5	0	0
mRNA	ID=cds3579	3805087-3806121	5	0	0
mRNA	ID=cds3587	3811955-3812410	5	0	0
mRNA	ID=cds3610	3838572-3839762	5	0	0
mRNA	ID=cds3624	3853631-3853993	5	0	0
mRNA	ID=cds3627	3854934-3856427	5	0	0
mRNA	ID=cds3673	3904876-3905601	5	0	0
mRNA	ID=cds3763	4009099-4009998	5	0	0
mRNA	ID=cds3764	4009886-4010839	5	0	0
mRNA	ID=cds3908	4188758-4189891	5	0	0
mRNA	ID=cds3950	4243252-4244442	5	0	0
mRNA	ID=cds4002	4304893-4305822	5	0	0
mRNA	ID=cds4005	4307471-4309003	5	0	0
mRNA	ID=cds4148	4451606-4452601	5	0	0
mRNA	ID=cds415	442275-442865	5	0	0
mRNA	ID=cds4216	4524928-4525560	5	0	0
mRNA	ID=cds4297	4615346-4616125	5	0	0
mRNA	ID=cds4319	4638965-4639651	5	0	0
mRNA	ID=cds437	466636-467454	5	0	0
mRNA	ID=cds44	47246-47776	5	0	0
mRNA	ID=cds517	555884-556096	5	0	0
mRNA	ID=cds55	59687-60346	5	0	0
mRNA	ID=cds571	602639-603886	5	0	0
mRNA	ID=cds574	605174-605422	5	0	0
mRNA	ID=cds614	650021-651079	5	0	0
mRNA	ID=cds63	72229-72927	5	0	0
mRNA	ID=cds647	683753-684478	5	0	0
mRNA	ID=cds648	684478-685152	5	0	0
mRNA	ID=cds649	685152-685892	5	0	0
mRNA	ID=cds730	781308-782351	5	0	0
mRNA	ID=cds755	808567-809607	5	0	0
mRNA	ID=cds833	892180-892656	5	0	0
mRNA	ID=cds935	1017708-1018160	5	0	0
mRNA	ID=cds938	1020361-1020990	5	0	0
mRNA	ID=cds943	1026334-1026996	5	0	0
mRNA	ID=cds944	1027169-1027582	5	0	0
mRNA	ID=cds947	1029287-1029565	5	0	0
mRNA	ID=cds1210	1286310-1286399	4.951129	0	0.333333
repeat_region		2671798-2671831	4.828677	0	0.404989
repeat_region		4439416-4439513	4.809052	0	0
repeat_region		3590587-3590685	4.807283	0	0.047004
mRNA	ID=cds620	656778-657161	4.711325	0	0
mRNA	ID=cds3790	4042222-4043652	4.707826	0	1
mRNA	ID=cds1303	1380987-1381985	4.666666	0	0
mRNA	ID=cds1454	1536874-1540614	4.5	0	3
mRNA	ID=cds2144	2249722-2250810	4.5	0	0
mRNA	ID=cds267	289653-289857	4.5	0	0
mRNA	ID=cds2749	2916067-2917407	4.5	0	0
mRNA	ID=cds3785	4038929-4039456	4.5	0	0
mRNA	ID=cds3786	4039438-4040022	4.5	0	0
mRNA	ID=cds382	406652-407329	4.5	0	0
mRNA	ID=cds385	408332-409243	4.5	0	0
mRNA	ID=cds2713	2872014-2873441	4.412861	0	0
mRNA	ID=cds4209	4516744-4517247	4.356935	0	1
mRNA	ID=cds2931	3123492-3124544	4.331295	0	0
mRNA	ID=cds2139	2242800-2244791	4.325016	0	0
repeat_region		2536557-2536654	4.321956	0	0.047004
mRNA	ID=cds2008	2093149-2093886	4.302429	0	0
mRNA	ID=cds2534	2683857-2685047	4.254588	0	0
repeat_region		3215456-3215544	4.091088	0	0
repeat_region		2806153-2806236	4.086651	0	0
mRNA	ID=cds645	680946-682616	4.067954	0	0
mRNA	ID=cds967	1049056-1049331	4.0388	0	0.166667
mRNA	ID=cds2285	2417256-2417810	4	0	2
mRNA	ID=cds284	303719-304429	4	0	2
mRNA	ID=cds4147	4450594-4451619	4	0	2
mRNA	ID=cds1263	1340682-1341008	4	0	1
mRNA	ID=cds1828	1920145-1920336	4	0	1
mRNA	ID=cds1838	1928058-1928414	4	0	1
mRNA	ID=cds184	213925-214125	4	0	1
mRNA	ID=cds1864	1952602-1955031	4	0	1
mRNA	ID=cds1990	2077557-2078615	4	0	1
mRNA	ID=cds2224	2350669-2352297	4	0	1
mRNA	ID=cds28	27293-28207	4	0	1
mRNA	ID=cds3069	3268647-3269792	4	0	1
mRNA	ID=cds336	359216-360370	4	0	1
mRNA	ID=cds3660	3890788-3891747	4	0	1
mRNA	ID=cds371	398249-398557	4	0	1
mRNA	ID=cds3839	4100845-4101519	4	0	1
mRNA	ID=cds3924	4202665-4203954	4	0	1
mRNA	ID=cds671	711261-712025	4	0	1
mRNA	ID=cds783	837413-837679	4	0	1
mRNA	ID=cds998	1081466-1082593	4	0	1
mRNA	ID=cds38	40417-41931	4	0	0.5
mRNA	ID=cds1660	1749101-1749748	4	0	0.012714
repeat_region		1189803-1189825	4	0	0
repeat_region		1325761-1325779	4	0	0
repeat_region		2406814-2406832	4	0	0
repeat_region		2854448-2854471	4	0	0
repeat_region		3179612-3179635	4	0	0
repeat_region		421596-421628	4	0	0
repeat_region		4309037-4309118	4	0	0
repeat_region		555230-555255	4	0	0
repeat_region		653775-653797	4	0	0
other ncRNA		852175-852263	4	0	0
mRNA	ID=cds1003	1087062-1089080	4	0	0
mRNA	ID=cds1014	1100074-1100907	4	0	0
mRNA	ID=cds1036	1119924-1120178	4	0	0
mRNA	ID=cds1163	1230409-1231677	4	0	0
mRNA	ID=cds1173	1243016-1243750	4	0	0
mRNA	ID=cds1256	1333315-1333482	4	0	0
mRNA	ID=cds1315	1392915-1393946	4	0	0
mRNA	ID=cds1324	1402765-1403673	4	0	0
mRNA	ID=cds1371	1445543-1447042	4	0	0
mRNA	ID=cds1379	1455521-1456288	4	0	0

mRNA	ID=cds1422	1506858-1507088	4	0	0
mRNA	ID=cds1471	1557038-1557934	4	0	0
mRNA	ID=cds1506	1607253-1608704	4	0	0
mRNA	ID=cds1511	1612828-1613709	4	0	0
mRNA	ID=cds1513	1615052-1616242	4	0	0
mRNA	ID=cds1567	1650920-1651939	4	0	0
mRNA	ID=cds1593	1677581-1678963	4	0	0
mRNA	ID=cds1655	1744724-1745029	4	0	0
mRNA	ID=cds1674	1763246-1763656	4	0	0
mRNA	ID=cds1681	1772710-1773468	4	0	0
mRNA	ID=cds1683	1775220-1776371	4	0	0
mRNA	ID=cds1736	1828786-1830006	4	0	0
mRNA	ID=cds1739	1832140-1832817	4	0	0
mRNA	ID=cds1757	1850645-1852003	4	0	0
mRNA	ID=cds1764	1858280-1859356	4	0	0
mRNA	ID=cds1775	1871598-1872101	4	0	0
mRNA	ID=cds1777	1872376-1872822	4	0	0
mRNA	ID=cds1883	1975290-1975868	4	0	0
mRNA	ID=cds1884	1975871-1976221	4	0	0
mRNA	ID=cds1904	1993842-1994066	4	0	0
mRNA	ID=cds1910	1998497-1999048	4	0	0
mRNA	ID=cds1938	2021992-2022615	4	0	0
mRNA	ID=cds1940	2023010-2023237	4	0	0
mRNA	ID=cds1953	2036176-2036847	4	0	0
mRNA	ID=cds1956	2038507-2039142	4	0	0
mRNA	ID=cds1967	2059040-2059957	4	0	0
mRNA	ID=cds196	229167-229970	4	0	0
mRNA	ID=cds2009	2093868-2094644	4	0	0
mRNA	ID=cds2087	2181738-2182538	4	0	0
mRNA	ID=cds2145	2250917-2252167	4	0	0
mRNA	ID=cds2181	2293399-2293608	4	0	0
mRNA	ID=cds2238	2365093-2366061	4	0	0
mRNA	ID=cds2283	2415103-2416623	4	0	0
mRNA	ID=cds2326	2457181-2458491	4	0	0
mRNA	ID=cds2349	2474716-2475651	4	0	0
mRNA	ID=cds2356	2486045-2487190	4	0	0
mRNA	ID=cds2413	2549299-2549748	4	0	0
mRNA	ID=cds2468	2604939-2606519	4	0	0
mRNA	ID=cds2479	2616893-2618182	4	0	0
mRNA	ID=cds2481	2619219-2620256	4	0	0
mRNA	ID=cds2485	2624717-2626960	4	0	0
mRNA	ID=cds2492	2633621-2633836	4	0	0
mRNA	ID=cds2532	2680885-2682078	4	0	0
mRNA	ID=cds2560	2712461-2713342	4	0	0
mRNA	ID=cds2643	2799370-2801514	4	0	0
mRNA	ID=cds2645	2802837-2804039	4	0	0
mRNA	ID=cds2672	2830498-2831937	4	0	0
mRNA	ID=cds2678	2839012-2840436	4	0	0
mRNA	ID=cds2690	2849886-2850158	4	0	0
mRNA	ID=cds2699	2860357-2861523	4	0	0
mRNA	ID=cds2702	2863123-2864487	4	0	0
mRNA	ID=cds2737	2899918-2901396	4	0	0
mRNA	ID=cds274	294363-294803	4	0	0
mRNA	ID=cds2892	3080899-3081819	4	0	0
mRNA	ID=cds2904	3092122-3093102	4	0	0
mRNA	ID=cds2926	3112572-3117134	4	0	0
mRNA	ID=cds2928	3119656-3121827	4	0	0
mRNA	ID=cds2970	3165873-3166268	4	0	0
mRNA	ID=cds3057	3253363-3254673	4	0	0
mRNA	ID=cds3099	3297494-3297937	4	0	0
mRNA	ID=cds3198	3402754-3403053	4	0	0
mRNA	ID=cds3230	3437163-3437531	4	0	0
mRNA	ID=cds3261	3453600-3454415	4	0	0
mRNA	ID=cds3297	3486982-3488202	4	0	0
mRNA	ID=cds3299	3488883-3489485	4	0	0
mRNA	ID=cds3305	3495025-3495831	4	0	0
mRNA	ID=cds3357	3554875-3556101	4	0	0
mRNA	ID=cds3389	3589032-3590348	4	0	0
mRNA	ID=cds3455	3665814-3667211	4	0	0
mRNA	ID=cds3615	3845328-3846647	4	0	0
mRNA	ID=cds3671	3901743-3903620	4	0	0
mRNA	ID=cds3755	4001311-4002201	4	0	0
mRNA	ID=cds3775	4021577-4022359	4	0	0
mRNA	ID=cds37	39244-40386	4	0	0
mRNA	ID=cds3806	4063832-4065217	4	0	0
mRNA	ID=cds3846	4107953-4108708	4	0	0
mRNA	ID=cds3871	4134131-4135036	4	0	0
mRNA	ID=cds3949	4241554-4243098	4	0	0
mRNA	ID=cds3974	4268681-4269037	4	0	0
mRNA	ID=cds3999	4301101-4302132	4	0	0
mRNA	ID=cds4010	4312367-4313125	4	0	0
mRNA	ID=cds4051	4356720-4358054	4	0	0
mRNA	ID=cds4110	4415721-4416476	4	0	0
mRNA	ID=cds4163	4469483-4470418	4	0	0
mRNA	ID=cds4181	4489229-4490548	4	0	0
mRNA	ID=cds4223	4530460-4531206	4	0	0
mRNA	ID=cds4225	4531819-4532076	4	0	0
mRNA	ID=cds4242	4550924-4552384	4	0	0
mRNA	ID=cds4283	4602183-4602860	4	0	0
mRNA	ID=cds456	489334-489495	4	0	0
mRNA	ID=cds582	613380-617261	4	0	0
mRNA	ID=cds588	622777-623733	4	0	0
mRNA	ID=cds608	644340-645803	4	0	0
mRNA	ID=cds639	674793-675770	4	0	0
mRNA	ID=cds65	74497-75480	4	0	0
mRNA	ID=cds704	748202-748930	4	0	0
mRNA	ID=cds765	818518-818970	4	0	0
mRNA	ID=cds768	820765-821721	4	0	0
mRNA	ID=cds780	834471-835433	4	0	0
mRNA	ID=cds785	838472-840754	4	0	0
mRNA	ID=cds801	857019-858284	4	0	0
mRNA	ID=cds828	889312-889689	4	0	0
mRNA	ID=cds829	889719-889976	4	0	0
mRNA	ID=cds838	897212-897700	4	0	0
mRNA	ID=cds840	899067-899798	4	0	0
mRNA	ID=cds845	903175-903690	4	0	0
mRNA	ID=cds854	913181-914080	4	0	0
mRNA	ID=cds880	948891-949481	4	0	0
mRNA	ID=cds888	959487-960251	4	0	0
mRNA	ID=cds973	1051512-1052585	4	0	0
mRNA	ID=cds97	111649-111846	4	0	0
mRNA	ID=cds9	9928-10494	4	0	0
mRNA	ID=cds1886	1976964-1977239	3.921156	0	0.166667
mRNA	ID=cds20	20233-20508	3.921156	0	0.166667
mRNA	ID=cds3379	3581506-3581781	3.921156	0	0.166667
repeat_region		66565-66812	3.89086	0	0.069969
repeat_region		898919-899016	3.64543	0	0.047004
repeat_region		1133809-1133906	3.568785	0	1.114819
repeat_region		248147-248334	3.508211	0	0.188151
mRNA	ID=cds1054	1133952-1134734	3.5	0	0
mRNA	ID=cds2124	2227460-2228407	3.5	0	0
mRNA	ID=cds221	250898-251953	3.5	0	0
mRNA	ID=cds2601	2757007-2758416	3.5	0	0
mRNA	ID=cds3612	3840478-3841812	3.5	0	0
mRNA	ID=cds4056	4363041-4363379	3.5	0	0
mRNA	ID=cds552	579474-579668	3.5	0	0
mRNA	ID=cds2438	2570511-2571527	3.353553	0	0
mRNA	ID=cds3037	3236602-3237567	3.353553	0	0
mRNA	ID=cds3737	3980981-3982216	3.353553	0	0
mRNA	ID=cds2934	3127065-3128165	3.337979	0	0
repeat_region		4247344-4247468	3.324832	0	0.055917
mRNA	ID=cds3487	3711675-3712115	3.148366	0	0
repeat_region		769898-769996	3.111078	0	0.156062
mRNA	ID=cds2352	2478660-2480198	3.110818	0	0
repeat_region		4006420-4006453	3.110248	0	0.032306
repeat_region		4038887-4038922	3.006157	0	0
mRNA	ID=cds1659	1748369-1749037	3	0	4
mgeRNA	gbkey=mobile_element	279338-279649	3	0	2

mRNA	ID=cds1139	1212551-1213282	3	0	2
mRNA	ID=cds1289	1367049-1367408	3	0	2
mRNA	ID=cds1546	1639879-1640091	3	0	2
mRNA	ID=cds1816	1906647-1906790	3	0	2
mRNA	ID=cds3097	3296233-3296868	3	0	2
mRNA	ID=cds3229	3436727-3437152	3	0	2
mRNA	ID=cds3962	4257511-4258026	3	0	2
mRNA	ID=cds4195	4504649-4504879	3	0	2
repeat_region		3884801-3884829	3	0	1
repeat_region		398204-398238	3	0	1
mRNA	ID=cds1441	1524271-1524888	3	0	1
mRNA	ID=cds1633	1719288-1720145	3	0	1
mRNA	ID=cds1842	1930817-1932628	3	0	1
mRNA	ID=cds2121	2224531-2225292	3	0	1
mRNA	ID=cds2153	2260387-2261517	3	0	1
mRNA	ID=cds2346	2473895-2474200	3	0	1
mRNA	ID=cds2351	2477224-2478552	3	0	1
mRNA	ID=cds2401	2536694-2537605	3	0	1
mRNA	ID=cds2458	2594927-2595640	3	0	1
mRNA	ID=cds2634	2794892-2795050	3	0	1
mRNA	ID=cds2677	2837546-2839003	3	0	1
mRNA	ID=cds2962	3154645-3155472	3	0	1
mRNA	ID=cds3010	3203346-3204278	3	0	1
mRNA	ID=cds3166	3367497-3368372	3	0	1
mRNA	ID=cds3182	3383560-3383823	3	0	1
mRNA	ID=cds3207	3410643-3410822	3	0	1
mRNA	ID=cds3292	3482240-3482458	3	0	1
mRNA	ID=cds3348	3541189-3542067	3	0	1
mRNA	ID=cds3370	3573094-3573687	3	0	1
mRNA	ID=cds3642	3871619-3872497	3	0	1
mRNA	ID=cds3848	4109638-4110237	3	0	1
mRNA	ID=cds471	505827-506306	3	0	1
mRNA	ID=cds921	1002112-1002654	3	0	1
mRNA	ID=cds3383	3583104-3584846	3	0	0.5
mRNA	ID=cds101	114522-115724	3	0	0.360041
repeat_region		129347-129361	3	0	0
repeat_region		1731738-1731761	3	0	0
repeat_region		3491780-3491805	3	0	0
repeat_region		3553818-3553841	3	0	0
repeat_region		4100719-4100744	3	0	0
repeat_region		4160164-4160185	3	0	0
repeat_region		4384042-4384061	3	0	0
repeat_region		4476962-4476995	3	0	0
repeat_region		496225-496250	3	0	0
mRNA	ID=cds102	115714-117099	3	0	0
mRNA	ID=cds1078	1158585-1160774	3	0	0
mRNA	ID=cds1088	1168296-1168553	3	0	0
mRNA	ID=cds1089	1168635-1169597	3	0	0
mRNA	ID=cds1127	1204954-1205365	3	0	0
mRNA	ID=cds1134	1208908-1209462	3	0	0
mRNA	ID=cds1218	1294215-1294364	3	0	0
mRNA	ID=cds1231	1309113-1309832	3	0	0
mRNA	ID=cds1237	1313294-1313794	3	0	0
mRNA	ID=cds1249	1325791-1326381	3	0	0
mRNA	ID=cds1270	1347004-1348131	3	0	0
mRNA	ID=cds1273	1349852-1350658	3	0	0
mRNA	ID=cds1281	1359144-1359908	3	0	0
mRNA	ID=cds1282	1359935-1360492	3	0	0
mRNA	ID=cds1285	1363574-1364839	3	0	0
mRNA	ID=cds1286	1364959-1365936	3	0	0
mRNA	ID=cds1294	1371246-1372127	3	0	0
mRNA	ID=cds130	149715-150953	3	0	0
mRNA	ID=cds1313	1390015-1390914	3	0	0
mRNA	ID=cds1344	1418708-1419130	3	0	0
mRNA	ID=cds1348	1421225-1421668	3	0	0
mRNA	ID=cds1412	1496962-1497501	3	0	0
mRNA	ID=cds1466	1553850-1553987	3	0	0
mRNA	ID=cds1493	1588878-1590200	3	0	0
mRNA	ID=cds1499	1601043-1602071	3	0	0
mRNA	ID=cds1516	1617598-1617981	3	0	0
mRNA	ID=cds1537	1635633-1635806	3	0	0
mRNA	ID=cds1548	1641279-1642328	3	0	0
mRNA	ID=cds1549	1642675-1642926	3	0	0
mRNA	ID=cds1551	1643370-1643657	3	0	0
mRNA	ID=cds1587	1670844-1671173	3	0	0
mRNA	ID=cds1605	1692284-1694095	3	0	0
mRNA	ID=cds1672	1762042-1762410	3	0	0
mRNA	ID=cds1735	1827755-1828789	3	0	0
mRNA	ID=cds1747	1839514-1839921	3	0	0
mRNA	ID=cds1748	1839887-1840159	3	0	0
mRNA	ID=cds1750	1841855-1842895	3	0	0
mRNA	ID=cds1756	1849911-1850552	3	0	0
mRNA	ID=cds1758	1852120-1852878	3	0	0
mRNA	ID=cds1779	1873697-1874878	3	0	0
mRNA	ID=cds1829	1920337-1920993	3	0	0
mRNA	ID=cds183	213678-213932	3	0	0
mRNA	ID=cds1869	1959996-1960484	3	0	0
mRNA	ID=cds186	214833-215255	3	0	0
mRNA	ID=cds1895	1985897-1985983	3	0	0
mRNA	ID=cds1905	1994134-1994856	3	0	0
mRNA	ID=cds1939	2022659-2022847	3	0	0
mRNA	ID=cds1982	2074332-2074778	3	0	0
mRNA	ID=cds2010	2094638-2095249	3	0	0
mRNA	ID=cds2012	2096471-2097637	3	0	0
mRNA	ID=cds2037	2125217-2126338	3	0	0
mRNA	ID=cds2091	2184982-2185320	3	0	0
mRNA	ID=cds2096	2190537-2190818	3	0	0
mRNA	ID=cds210	240343-240816	3	0	0
mRNA	ID=cds2125	2228646-2229044	3	0	0
mRNA	ID=cds2147	2253377-2254036	3	0	0
mRNA	ID=cds219	248358-250070	3	0	0
mRNA	ID=cds2212	2332358-2332981	3	0	0
mRNA	ID=cds2226	2353543-2354733	3	0	0
mRNA	ID=cds2235	2362576-2363001	3	0	0
mRNA	ID=cds2244	2371294-2371560	3	0	0
mRNA	ID=cds2291	2421758-2422531	3	0	0
mRNA	ID=cds2298	2428297-2428785	3	0	0
mRNA	ID=cds2335	2467153-2468484	3	0	0
mRNA	ID=cds2414	2549735-2550160	3	0	0
mRNA	ID=cds243	265777-265998	3	0	0
mRNA	ID=cds2454	2590784-2590984	3	0	0
mRNA	ID=cds2488	2628348-2628887	3	0	0
mRNA	ID=cds2543	2696781-2697629	3	0	0
mRNA	ID=cds2557	2708442-2710064	3	0	0
mRNA	ID=cds2558	2710049-2710786	3	0	0
mRNA	ID=cds2616	2771340-2773043	3	0	0
mRNA	ID=cds2688	2848669-2849019	3	0	0
mRNA	ID=cds2694	2854475-2854828	3	0	0
mRNA	ID=cds2712	2871409-2872014	3	0	0
mRNA	ID=cds2755	2922757-2923302	3	0	0
mRNA	ID=cds2766	2936910-2937332	3	0	0
mRNA	ID=cds2839	3023788-3025107	3	0	0
mRNA	ID=cds2886	3073239-3074204	3	0	0
mRNA	ID=cds2898	3087777-3088274	3	0	0
mRNA	ID=cds289	309970-310560	3	0	0
mRNA	ID=cds298	315710-316393	3	0	0
mRNA	ID=cds2999	3190886-3192547	3	0	0
mRNA	ID=cds3018	3212989-3213495	3	0	0
mRNA	ID=cds3031	3231750-3232166	3	0	0
mRNA	ID=cds3034	3233982-3234485	3	0	0
mRNA	ID=cds3072	3271595-3272929	3	0	0
mRNA	ID=cds3208	3410825-3411487	3	0	0
mRNA	ID=cds320	340349-341731	3	0	0
mRNA	ID=cds3211	3416412-3416633	3	0	0
mRNA	ID=cds3259	3451530-3451949	3	0	0
mRNA	ID=cds3281	3472995-3473354	3	0	0
mRNA	ID=cds3285	3475662-3475880	3	0	0
mRNA	ID=cds3361	3559520-3559846	3	0	0

mRNA	ID=cds3415	3615038-3615802	3	0	0
mRNA	ID=cds3425	3628991-3630613	3	0	0
mRNA	ID=cds3470	3690641-3693259	3	0	0
mRNA	ID=cds3474	3696049-3696240	3	0	0
mRNA	ID=cds3496	3718703-3719224	3	0	0
mRNA	ID=cds3527	3750015-3750989	3	0	0
mRNA	ID=cds3575	3801081-3802160	3	0	0
mRNA	ID=cds3576	3802204-3803139	3	0	0
mRNA	ID=cds3582	3808366-3809175	3	0	0
mRNA	ID=cds3588	3812517-3813113	3	0	0
mRNA	ID=cds358	383283-383840	3	0	0
mRNA	ID=cds3603	3830242-3832560	3	0	0
mRNA	ID=cds3614	3843799-3845190	3	0	0
mRNA	ID=cds3632	3861922-3862638	3	0	0
mRNA	ID=cds3665	3895529-3895996	3	0	0
mRNA	ID=cds3674	3905616-3906389	3	0	0
mRNA	ID=cds3692	3924568-3925026	3	0	0
mRNA	ID=cds3707	3946472-3947992	3	0	0
mRNA	ID=cds3712	3950507-3951436	3	0	0
mRNA	ID=cds3718	3958035-3958277	3	0	0
mRNA	ID=cds3756	4002253-4002720	3	0	0
mRNA	ID=cds376	402505-402825	3	0	0
mRNA	ID=cds3849	4110338-4110778	3	0	0
mRNA	ID=cds3992	4291186-4291569	3	0	0
mRNA	ID=cds4009	4311891-4312220	3	0	0
mRNA	ID=cds4026	4325158-4327386	3	0	0
mRNA	ID=cds403	431536-432075	3	0	0
mRNA	ID=cds4064	4371388-4372257	3	0	0
mRNA	ID=cds4079	4383727-4384041	3	0	0
mRNA	ID=cds4118	4422539-4422814	3	0	0
mRNA	ID=cds4130	4431187-4432047	3	0	0
mRNA	ID=cds4183	4491398-4492429	3	0	0
mRNA	ID=cds4219	4526953-4528266	3	0	0
mRNA	ID=cds4270	4586899-4587102	3	0	0
mRNA	ID=cds4281	4600111-4600881	3	0	0
mRNA	ID=cds442	472190-473476	3	0	0
mRNA	ID=cds446	475896-476249	3	0	0
mRNA	ID=cds480	515143-515820	3	0	0
mRNA	ID=cds569	597937-601080	3	0	0
mRNA	ID=cds59	66835-68337	3	0	0
mRNA	ID=cds616	653085-653765	3	0	0
mRNA	ID=cds621	657254-657448	3	0	0
mRNA	ID=cds624	659648-660601	3	0	0
mRNA	ID=cds625	660860-661501	3	0	0
mRNA	ID=cds641	676638-678065	3	0	0
mRNA	ID=cds670	710828-711121	3	0	0
mRNA	ID=cds682	723630-724202	3	0	0
mRNA	ID=cds74	84368-85312	3	0	0
mRNA	ID=cds758	811493-812170	3	0	0
mRNA	ID=cds814	874558-875886	3	0	0
mRNA	ID=cds823	884169-884453	3	0	0
mRNA	ID=cds919	998439-1001039	3	0	0
mRNA	ID=cds924	1003991-1005001	3	0	0
mRNA	ID=cds965	1047911-1048555	3	0	0
mRNA	ID=cds970	1050684-1050896	3	0	0
mRNA	ID=cds978	1058479-1061025	3	0	0
mgeRNA	gbkey=mobile_element	1648867-1649572	2.98591	0	0.714285
mRNA	ID=cds1565	1648869-1649561	2.98591	0	0.714285
mRNA	ID=cds2626	2784419-2786671	2.898862	0	0
repeat_region		1083905-1083990	2.767461	0	0
repeat_region		2175430-2175506	2.76445	0	0
mRNA	ID=cds3519	3744117-3745103	2.752731	0	0
repeat_region		844854-844952	2.656048	0	0.674099
repeat_region		138699-138796	2.652643	0	0.499468
repeat_region		1821355-1821452	2.552345	0	0.296056
repeat_region		3112400-3112567	2.528554	0	0.073238
mRNA	ID=cds2636	2795542-2796066	2.5	0	1
mRNA	ID=cds1531	1631646-1632236	2.5	0	0.5
mRNA	ID=cds3073	3273304-3274875	2.5	0	0.5
mgeRNA	gbkey=mobile_element	270986-271414	2.5	0	0
mRNA	ID=cds1110	1193050-1193511	2.5	0	0
mRNA	ID=cds1265	1341621-1342370	2.5	0	0
mRNA	ID=cds2022	2107605-2108162	2.5	0	0
mRNA	ID=cds248	270988-271413	2.5	0	0
mRNA	ID=cds252	274549-275952	2.5	0	0
mRNA	ID=cds3809	4068538-4069779	2.5	0	0
mRNA	ID=cds3826	4086130-4087878	2.5	0	0
mRNA	ID=cds505	543281-544516	2.5	0	0
mRNA	ID=cds3606	3834976-3836160	2.471136	0	0
mRNA	ID=cds3315	3505370-3505723	2.433013	0	0
mRNA	ID=cds2431	2563503-2564906	2.3669	0	0
repeat_region		2566161-2566343	2.362023	0	0.196009
mRNA	ID=cds333	357015-357914	2.353553	0	0
mRNA	ID=cds4208	4516550-4516825	2.351015	0	0
mRNA	ID=cds1950	2032737-2033267	2.264024	0	0
mRNA	ID=cds1830	1921389-1921730	2.235702	0	0
repeat_region		3537768-3537801	2.206427	0	0.003013
mRNA	ID=cds1396	1477162-1478919	2.197203	0	0
repeat_region		3954907-3954932	2.187024	0	0
mRNA	ID=cds689	733356-734876	2.183333	0	0
mRNA	ID=cds3302	3490590-3491771	2.152753	0	0
mRNA	ID=cds2812	2990116-2991492	2.125	0	0
mRNA	ID=cds743	794312-795085	2.086112	0	1
repeat_region		2886356-2886392	2.085465	0	0
repeat_region		4432519-4432629	2.077358	0	0
repeat_region		353823-353993	2.062113	0	1.013654
repeat_region		3648735-3648834	2.051885	0	0.693213
mRNA	ID=cds3012	3205393-3205998	2.042944	0	1
mRNA	ID=cds2531	2677486-2680767	2	0	3
other ncRNA		77367-77593	2	0	2
mRNA	ID=cds1915	2003737-2004102	2	0	2
mRNA	ID=cds258	279338-279586	2	0	2
mRNA	ID=cds271	291546-292172	2	0	2
mRNA	ID=cds3027	3224256-3225689	2	0	2
mRNA	ID=cds3260	3451951-3453420	2	0	2
mRNA	ID=cds395	423561-424142	2	0	2
mRNA	ID=cds4077	4381862-4383364	2	0	2
mRNA	ID=cds1018	1103174-1103629	2	0	1
mRNA	ID=cds1052	1131797-1133005	2	0	1
mRNA	ID=cds1439	1521331-1522392	2	0	1
mRNA	ID=cds1608	1696176-1697204	2	0	1
mRNA	ID=cds1785	1877031-1877279	2	0	1
mRNA	ID=cds1840	1928905-1930083	2	0	1
mRNA	ID=cds1871	1960996-1963074	2	0	1
mRNA	ID=cds1913	2001896-2003302	2	0	1
mRNA	ID=cds2845	3031087-3031635	2	0	1
mRNA	ID=cds306	322982-323677	2	0	1
mRNA	ID=cds3511	3735520-3737550	2	0	1
mRNA	ID=cds3952	4245994-4247334	2	0	1
mRNA	ID=cds4185	4493213-4494232	2	0	1
mRNA	ID=cds4285	4603827-4604063	2	0	1
mRNA	ID=cds4292	4610151-4610312	2	0	1
mRNA	ID=cds534	570116-570667	2	0	1
mRNA	ID=cds586	620408-621412	2	0	1
mRNA	ID=cds963	1045072-1047168	2	0	1
mRNA	ID=cds803	859397-861829	2	0	0.5
mRNA	ID=cds263	284619-286001	2	0	0.25
repeat_region		1117091-1117111	2	0	0
repeat_region		3161666-3161695	2	0	0
repeat_region		3218902-3218932	2	0	0
repeat_region		4113700-4113729	2	0	0
repeat_region		4534428-4534453	2	0	0
repeat_region		505800-505819	2	0	0
repeat_region		863556-863593	2	0	0
mgeRNA	gbkey=mobile_element	279155-279335	2	0	0
mgeRNA	gbkey=mobile_element	4505148-4505481	2	0	0
mRNA	ID=cds1016	1101375-1101764	2	0	0
mRNA	ID=cds1033	1117703-1118269	2	0	0

mRNA	ID=cds1053	1133025-1133780	2	0	0
mRNA	ID=cds1055	1134787-1135485	2	0	0
mRNA	ID=cds1148	1221528-1221857	2	0	0
mRNA	ID=cds1157	1226294-1226695	2	0	0
mRNA	ID=cds1159	1227302-1227961	2	0	0
mRNA	ID=cds1162	1229990-1230409	2	0	0
mRNA	ID=cds1183	1257152-1257736	2	0	0
mRNA	ID=cds1232	1309872-1310270	2	0	0
mRNA	ID=cds123	143702-144472	2	0	0
mRNA	ID=cds1323	1401279-1402589	2	0	0
mRNA	ID=cds133	152243-152812	2	0	0
mRNA	ID=cds1351	1423401-1423664	2	0	0
mRNA	ID=cds1369	1443904-1444230	2	0	0
mRNA	ID=cds1372	1447100-1449373	2	0	0
mRNA	ID=cds1378	1454454-1455524	2	0	0
mRNA	ID=cds1386	1462495-1463085	2	0	0
mRNA	ID=cds1398	1480279-1480884	2	0	0
mRNA	ID=cds1420	1504196-1504732	2	0	0
mRNA	ID=cds1429	1512786-1513580	2	0	0
mRNA	ID=cds1431	1515123-1515218	2	0	0
mRNA	ID=cds1437	1518286-1518951	2	0	0
mRNA	ID=cds1447	1531076-1531309	2	0	0
mRNA	ID=cds1455	1540696-1542084	2	0	0
mRNA	ID=cds1472	1557931-1558953	2	0	0
mRNA	ID=cds1474	1560519-1561100	2	0	0
mRNA	ID=cds1476	1563782-1565164	2	0	0
mRNA	ID=cds1481	1573271-1575643	2	0	0
mRNA	ID=cds1482	1575681-1577366	2	0	0
mRNA	ID=cds1487	1582231-1584510	2	0	0
mRNA	ID=cds1512	1613787-1614902	2	0	0
mRNA	ID=cds1517	1618013-1618231	2	0	0
mRNA	ID=cds1552	1643657-1643896	2	0	0
mRNA	ID=cds1553	1643921-1644226	2	0	0
mRNA	ID=cds1582	1666723-1667616	2	0	0
mRNA	ID=cds1584	1669400-1669708	2	0	0
mRNA	ID=cds1588	1671160-1671525	2	0	0
mRNA	ID=cds1637	1723705-1723944	2	0	0
mRNA	ID=cds1646	1734145-1735314	2	0	0
mRNA	ID=cds1663	1752956-1753165	2	0	0
mRNA	ID=cds1667	1756898-1757314	2	0	0
mRNA	ID=cds1724	1817479-1817829	2	0	0
mRNA	ID=cds1773	1868409-1869884	2	0	0
mRNA	ID=cds181	211877-212266	2	0	0
mRNA	ID=cds1861	1950290-1950685	2	0	0
mRNA	ID=cds1870	1960604-1960996	2	0	0
mRNA	ID=cds1880	1971384-1973348	2	0	0
mRNA	ID=cds188	216179-217003	2	0	0
mRNA	ID=cds1894	1985531-1985782	2	0	0
mRNA	ID=cds191	219591-219995	2	0	0
mRNA	ID=cds1920	2007845-2008513	2	0	0
mRNA	ID=cds1925	2011253-2012911	2	0	0
mRNA	ID=cds1932	2018111-2019115	2	0	0
mRNA	ID=cds1934	2019528-2019893	2	0	0
mRNA	ID=cds1935	2019893-2020630	2	0	0
mRNA	ID=cds1964	2054882-2055598	2	0	0
mRNA	ID=cds2007	2092559-2093149	2	0	0
mRNA	ID=cds2014	2099420-2099768	2	0	0
mRNA	ID=cds201	234027-234782	2	0	0
mRNA	ID=cds2027	2112526-2113920	2	0	0
mRNA	ID=cds2066	2163213-2163545	2	0	0
mRNA	ID=cds2068	2165326-2165544	2	0	0
mRNA	ID=cds2090	2183939-2184763	2	0	0
mRNA	ID=cds2093	2186452-2188932	2	0	0
mRNA	ID=cds209	239419-240189	2	0	0
mRNA	ID=cds2148	2254107-2255357	2	0	0
mRNA	ID=cds2152	2259449-2260387	2	0	0
mRNA	ID=cds2178	2290429-2290986	2	0	0
mRNA	ID=cds2190	2300772-2301035	2	0	0
mRNA	ID=cds2205	2321469-2322131	2	0	0
mRNA	ID=cds2210	2326170-2327819	2	0	0
mRNA	ID=cds2223	2349038-2350396	2	0	0
mRNA	ID=cds2228	2355838-2356023	2	0	0
mRNA	ID=cds2229	2356064-2356867	2	0	0
mRNA	ID=cds222	252005-252298	2	0	0
mRNA	ID=cds2232	2359451-2360233	2	0	0
mRNA	ID=cds2234	2361755-2362297	2	0	0
mRNA	ID=cds2237	2363950-2365089	2	0	0
mRNA	ID=cds2240	2368040-2368930	2	0	0
mRNA	ID=cds2242	2370579-2370914	2	0	0
mRNA	ID=cds2257	2384956-2385459	2	0	0
mRNA	ID=cds2323	2453105-2453668	2	0	0
mRNA	ID=cds2347	2474203-2474253	2	0	0
mRNA	ID=cds2361	2492720-2492995	2	0	0
mRNA	ID=cds239	263972-264430	2	0	0
mRNA	ID=cds241	264844-265311	2	0	0
mRNA	ID=cds2428	2561614-2561991	2	0	0
mRNA	ID=cds2429	2562002-2562394	2	0	0
mRNA	ID=cds2430	2562545-2563354	2	0	0
mRNA	ID=cds2432	2564903-2566129	2	0	0
mRNA	ID=cds2442	2573492-2573827	2	0	0
mRNA	ID=cds2455	2591094-2591792	2	0	0
mRNA	ID=cds2464	2599840-2601858	2	0	0
mRNA	ID=cds2529	2674872-2676383	2	0	0
mRNA	ID=cds2599	2755666-2756622	2	0	0
mRNA	ID=cds260	280053-281207	2	0	0
mRNA	ID=cds2610	2767725-2768426	2	0	0
mRNA	ID=cds2614	2770024-2770176	2	0	0
mRNA	ID=cds2615	2770189-2771088	2	0	0
mRNA	ID=cds2625	2781660-2783033	2	0	0
mRNA	ID=cds2631	2792275-2793675	2	0	0
mRNA	ID=cds2642	2798987-2799397	2	0	0
mRNA	ID=cds2687	2847996-2848457	2	0	0
mRNA	ID=cds2696	2857782-2858438	2	0	0
mRNA	ID=cds2698	2859452-2860360	2	0	0
mRNA	ID=cds2719	2878396-2879070	2	0	0
mRNA	ID=cds2725	2886409-2888121	2	0	0
mRNA	ID=cds2731	2892941-2893801	2	0	0
mRNA	ID=cds273	293169-294023	2	0	0
mRNA	ID=cds2754	2921806-2922135	2	0	0
mRNA	ID=cds2785	2961729-2962199	2	0	0
mRNA	ID=cds2805	2985558-2986190	2	0	0
mRNA	ID=cds2809	2987957-2988382	2	0	0
mRNA	ID=cds2847	3034230-3034304	2	0	0
mRNA	ID=cds2888	3075493-3076881	2	0	0
mRNA	ID=cds2910	3096580-3097587	2	0	0
mRNA	ID=cds2927	3117619-3119301	2	0	0
mRNA	ID=cds2932	3124544-3126043	2	0	0
mRNA	ID=cds2937	3130476-3131234	2	0	0
mRNA	ID=cds2943	3137738-3137986	2	0	0
mRNA	ID=cds2952	3144878-3145294	2	0	0
mRNA	ID=cds2952	3145294-3145713	2	0	0
mRNA	ID=cds2974	3167850-3168509	2	0	0
mRNA	ID=cds2977	3170552-3171133	2	0	0
mRNA	ID=cds2992	3185443-3187887	2	0	0
mRNA	ID=cds3050	3248584-3248976	2	0	0
mRNA	ID=cds3055	3252341-3253042	2	0	0
mRNA	ID=cds3064	3263061-3264050	2	0	0
mRNA	ID=cds3077	3276936-3278216	2	0	0
mRNA	ID=cds3080	3279093-3279647	2	0	0
mRNA	ID=cds3098	3296996-3297514	2	0	0
mRNA	ID=cds3165	3367036-3367500	2	0	0
mRNA	ID=cds3212	3417129-3418088	2	0	0
mRNA	ID=cds3215	3420458-3421216	2	0	0
mRNA	ID=cds323	344628-344873	2	0	0
mRNA	ID=cds3264	3457839-3459035	2	0	0
mRNA	ID=cds3272	3463565-3464242	2	0	0
mRNA	ID=cds3300	3489475-3489642	2	0	0
mRNA	ID=cds330	351930-353816	2	0	0

mRNA	ID=cds3372	3575088-3575615	2	0	0
mRNA	ID=cds3392	3592226-3593503	2	0	0
mRNA	ID=cds3393	3593500-3594426	2	0	0
mRNA	ID=cds3396	3596578-3597681	2	0	0
mRNA	ID=cds3440	3648260-3648685	2	0	0
mRNA	ID=cds344	370448-371329	2	0	0
mRNA	ID=cds3484	3708822-3710030	2	0	0
mRNA	ID=cds3486	3711115-3711678	2	0	0
mRNA	ID=cds351	377686-378795	2	0	0
mRNA	ID=cds3536	3764360-3765202	2	0	0
mRNA	ID=cds3548	3774194-3774403	2	0	0
mRNA	ID=cds3549	3774688-3775050	2	0	0
mRNA	ID=cds354	380068-380483	2	0	0
mRNA	ID=cds3562	3787070-3788104	2	0	0
mRNA	ID=cds3573	3799006-3800022	2	0	0
mRNA	ID=cds3608	3837198-3838016	2	0	0
mRNA	ID=cds3626	3854438-3854887	2	0	0
mRNA	ID=cds3666	3896045-3896632	2	0	0
mRNA	ID=cds3718	3958277-3958483	2	0	0
mRNA	ID=cds374	400610-400870	2	0	0
mRNA	ID=cds3836	4097514-4098548	2	0	0
mRNA	ID=cds3838	4099713-4100696	2	0	0
mRNA	ID=cds3872	4135063-4135680	2	0	0
mRNA	ID=cds3874	4137069-4137731	2	0	0
mRNA	ID=cds3909	4189888-4190658	2	0	0
mRNA	ID=cds3912	4191592-4192227	2	0	0
mRNA	ID=cds3926	4211257-4211640	2	0	0
mRNA	ID=cds3927	4211703-4212146	2	0	0
mRNA	ID=cds3944	4235657-4237753	2	0	0
mRNA	ID=cds3951	4244807-4245922	2	0	0
mRNA	ID=cds3966	4260863-4261105	2	0	0
mRNA	ID=cds3977	4272783-4273064	2	0	0
mRNA	ID=cds3980	4275492-4275956	2	0	0
mRNA	ID=cds3983	4279806-4281098	2	0	0
mRNA	ID=cds3985	4282922-4283236	2	0	0
mRNA	ID=cds4003	4305806-4306501	2	0	0
mRNA	ID=cds4004	4306512-4307492	2	0	0
mRNA	ID=cds4012	4313548-4314105	2	0	0
mRNA	ID=cds4044	4349866-4350096	2	0	0
mRNA	ID=cds4068	4374576-4374722	2	0	0
mRNA	ID=cds4095	4402409-4402606	2	0	0
mRNA	ID=cds4111	4416584-4417648	2	0	0
mRNA	ID=cds4124	4425717-4426118	2	0	0
mRNA	ID=cds4134	4435730-4436668	2	0	0
mRNA	ID=cds4162	4469009-4469470	2	0	0
mRNA	ID=cds4164	4470422-4470556	2	0	0
mRNA	ID=cds4192	4502081-4503298	2	0	0
mRNA	ID=cds4196	4504884-4505132	2	0	0
mRNA	ID=cds4197	4505220-4505474	2	0	0
mRNA	ID=cds4202	4509481-4510437	2	0	0
mRNA	ID=cds4205	4512376-4514700	2	0	0
mRNA	ID=cds4231	4538980-4539582	2	0	0
mRNA	ID=cds4241	4549659-4550843	2	0	0
mRNA	ID=cds4257	4567021-4567941	2	0	0
mRNA	ID=cds4262	4574935-4575981	2	0	0
mRNA	ID=cds4284	4602898-4603686	2	0	0
mRNA	ID=cds490	527864-528354	2	0	0
mRNA	ID=cds499	535810-536688	2	0	0
mRNA	ID=cds4	5234-5530	2	0	0
mRNA	ID=cds523	562553-563068	2	0	0
mRNA	ID=cds529	567258-567470	2	0	0
mRNA	ID=cds54	59052-59228	2	0	0
mRNA	ID=cds556	581375-582029	2	0	0
mRNA	ID=cds557	582176-582358	2	0	0
mRNA	ID=cds560	585370-586131	2	0	0
mRNA	ID=cds568	596702-597925	2	0	0
mRNA	ID=cds578	608682-609302	2	0	0
mRNA	ID=cds585	619419-620411	2	0	0
mRNA	ID=cds58	65855-66550	2	0	0
mRNA	ID=cds601	637050-637796	2	0	0
mRNA	ID=cds618	655780-656340	2	0	0
mRNA	ID=cds640	675934-676641	2	0	0
mRNA	ID=cds665	707557-708963	2	0	0
mRNA	ID=cds674	714635-715129	2	0	0
mRNA	ID=cds678	717485-719683	2	0	0
mRNA	ID=cds67	77388-77519	2	0	0
mRNA	ID=cds696	740298-741779	2	0	0
mRNA	ID=cds706	751452-752018	2	0	0
mRNA	ID=cds73	83622-83708	2	0	0
mRNA	ID=cds753	806656-807132	2	0	0
mRNA	ID=cds754	807191-808480	2	0	0
mRNA	ID=cds757	810745-811500	2	0	0
mRNA	ID=cds824	884539-885354	2	0	0
mRNA	ID=cds836	895357-896310	2	0	0
mRNA	ID=cds915	995208-996167	2	0	0
mRNA	ID=cds920	1001030-1002100	2	0	0
mRNA	ID=cds923	1003170-1003880	2	0	0
mRNA	ID=cds982	1063259-1064515	2	0	0
mRNA	ID=cds997	1080683-1081408	2	0	0
mRNA	ID=cds999	1082599-1083870	2	0	0
mRNA	ID=cds1461	1548485-1549369	1.971644	0	0
repeat_region		3561605-3561702	1.948762	0	1.181164
repeat_region		4135812-4135909	1.919608	0	0.025102
mRNA	ID=cds2082	2176843-2178120	1.585272	0	0
repeat_region		856839-856936	1.526495	0	3.025102
mRNA	ID=cds2665	2824414-2825373	1.5	0	4
mRNA	ID=cds2371	2503569-2504654	1.5	0	1
mRNA	ID=cds3011	3204485-3205396	1.5	0	1
mRNA	ID=cds1358	1431108-1431698	1.5	0	0.5
mRNA	ID=cds1195	1268391-1268498	1.5	0	0
mRNA	ID=cds1197	1269461-1269568	1.5	0	0
mRNA	ID=cds1377	1453949-1454446	1.5	0	0
mRNA	ID=cds1452	1534638-1535333	1.5	0	0
mRNA	ID=cds1533	1632909-1633871	1.5	0	0
mRNA	ID=cds1872	1963067-1964215	1.5	0	0
mRNA	ID=cds2527	2672722-2673783	1.5	0	0
mRNA	ID=cds4201	4508713-4509480	1.5	0	0
repeat_region		542311-542407	1.457672	0	0.5
mRNA	ID=cds3328	3517487-3518725	1.4	0	0
mRNA	ID=cds1009	1095066-1096052	1.360041	0	0
mRNA	ID=cds3167	3368369-3369058	1.353553	0	0
mRNA	ID=cds3263	3456361-3457842	1.333333	0	0
mRNA	ID=cds3812	4071762-4072658	1.333333	0	0
mRNA	ID=cds70	79464-80864	1.333333	0	0
repeat_region		3085934-3085959	1.312522	0	0.207332
repeat_region		247458-247541	1.297786	0	0.104217
mRNA	ID=cds3035	3234562-3235254	1.295804	0	0
mRNA	ID=cds43	45807-47138	1.288675	0	0
mRNA	ID=cds748	798845-799798	1.259437	0	0
repeat_region		3772259-3772357	1.251315	0	0
mRNA	ID=cds693	737315-738076	1.25	0	0
mRNA	ID=cds2630	2790757-2792037	1.199007	0	0
mRNA	ID=cds677	716169-717488	1.18885	0	0
mRNA	ID=cds750	801110-802543	1.170783	0	1
mRNA	ID=cds912	992500-993267	1.167332	0	0
mRNA	ID=cds2751	2918770-2920122	1.162221	0	0
repeat_region		3851735-3851770	1.121641	0	0
repeat_region		276899-276965	1.068011	0	0
repeat_region		2792050-2792185	1.06717	0	0.04596
mRNA	ID=cds217	246712-247461	1.041824	0	0
repeat_region		705120-705155	1.010997	0	0
mRNA	ID=cds1123	1203045-1203383	1	0	2
mRNA	ID=cds1469	1555136-1556062	1	0	2
mRNA	ID=cds751	802726-804987	1	0	2
mRNA	ID=cds977	1057307-1058479	1	0	2
mRNA	ID=cds3751	3998315-3999079	1	0	1.639959
mRNA	ID=cds1141	1215012-1215248	1	0	1
mRNA	ID=cds1445	1528610-1529347	1	0	1

mRNA	ID=cds1464	1550852-1551862	1	0	1
mRNA	ID=cds1559	1646532-1646687	1	0	1
mRNA	ID=cds1569	1653371-1653697	1	0	1
mRNA	ID=cds1865	1955056-1956156	1	0	1
mRNA	ID=cds2031	2118184-2119578	1	0	1
mRNA	ID=cds2405	2540534-2541550	1	0	1
mRNA	ID=cds2605	2763535-2763798	1	0	1
mRNA	ID=cds2608	2765732-2766595	1	0	1
mRNA	ID=cds2627	2787007-2787984	1	0	1
mRNA	ID=cds2732	2893798-2894577	1	0	1
mRNA	ID=cds3062	3260474-3261682	1	0	1
mRNA	ID=cds3280	3472700-3472987	1	0	1
mRNA	ID=cds329	350439-351890	1	0	1
mRNA	ID=cds3639	3868461-3869753	1	0	1
mRNA	ID=cds3981	4276502-4277851	1	0	1
mRNA	ID=cds4206	4514787-4515740	1	0	1
mRNA	ID=cds4228	4534637-4535617	1	0	1
mRNA	ID=cds494	530519-531445	1	0	1
mRNA	ID=cds621	657448-658041	1	0	1
mRNA	ID=cds1604	1690914-1692287	1	0	0.5
repeat_region		1011142-1011170	1	0	0
repeat_region		1080335-1080368	1	0	0
repeat_region		1100044-1100062	1	0	0
repeat_region		167281-167305	1	0	0
repeat_region		1850586-1850621	1	0	0
repeat_region		1866916-1866954	1	0	0
repeat_region		1964346-1964408	1	0	0
repeat_region		2039176-2039209	1	0	0
repeat_region		258240-258260	1	0	0
repeat_region		3117583-3117609	1	0	0
repeat_region		3358710-3358808	1	0	0
repeat_region		34727-34762	1	0	0
repeat_region		3775087-3775120	1	0	0
repeat_region		3825361-3825439	1	0	0
repeat_region		4478962-4478979	1	0	0
repeat_region		688487-688520	1	0	0
repeat_region		835445-835468	1	0	0
mgeRNA	gbkey=mobile_element	4506703-4507029	1	0	0
mRNA	ID=cds1002	1085744-1087069	1	0	0
mRNA	ID=cds1017	1101769-1102419	1	0	0
mRNA	ID=cds1021	1104637-1104948	1	0	0
mRNA	ID=cds1034	1118530-1118670	1	0	0
mRNA	ID=cds103	117109-117549	1	0	0
mRNA	ID=cds1047	1129058-1129351	1	0	0
mRNA	ID=cds1049	1130241-1130657	1	0	0
mRNA	ID=cds1050	1130661-1131065	1	0	0
mRNA	ID=cds1056	1135497-1136594	1	0	0
mRNA	ID=cds1057	1136594-1137535	1	0	0
mRNA	ID=cds1058	1137601-1139244	1	0	0
mRNA	ID=cds1059	1139256-1140209	1	0	0
mRNA	ID=cds1091	1173315-1174388	1	0	0
mRNA	ID=cds1097	1179702-1180490	1	0	0
mRNA	ID=cds10	10643-11356	1	0	0
mRNA	ID=cds1114	1196756-1197460	1	0	0
mRNA	ID=cds1116	1198902-1200029	1	0	0
mRNA	ID=cds1117	1200010-1200255	1	0	0
mRNA	ID=cds1121	1202247-1202447	1	0	0
mRNA	ID=cds1125	1203627-1204760	1	0	0
mRNA	ID=cds1130	1206724-1207353	1	0	0
mRNA	ID=cds1133	1208342-1208842	1	0	0
mRNA	ID=cds1138	1211926-1212330	1	0	0
mRNA	ID=cds1143	1215592-1215858	1	0	0
mRNA	ID=cds1146	1218206-1218424	1	0	0
mRNA	ID=cds1175	1244383-1244823	1	0	0
mRNA	ID=cds1199	1271342-1271572	1	0	0
mRNA	ID=cds11	11382-11786	1	0	0
mRNA	ID=cds1238	1313880-1314059	1	0	0
mRNA	ID=cds1278	1355447-1355692	1	0	0
mRNA	ID=cds1280	1357514-1358932	1	0	0
mRNA	ID=cds1283	1360767-1362254	1	0	0
mRNA	ID=cds1288	1366825-1367049	1	0	0
mRNA	ID=cds1290	1367417-1367638	1	0	0
mRNA	ID=cds1297	1374058-1374846	1	0	0
mRNA	ID=cds1298	1374856-1375911	1	0	0
mRNA	ID=cds1300	1378172-1378831	1	0	0
mRNA	ID=cds1321	1398271-1399797	1	0	0
mRNA	ID=cds1336	1415512-1415787	1	0	0
mRNA	ID=cds1337	1415862-1416032	1	0	0
mRNA	ID=cds1341	1417346-1417480	1	0	0
mRNA	ID=cds1345	1419143-1420000	1	0	0
mRNA	ID=cds1349	1421424-1421609	1	0	0
mRNA	ID=cds1352	1423654-1424106	1	0	0
mRNA	ID=cds1381	1457078-1458505	1	0	0
mRNA	ID=cds1387	1463189-1463254	1	0	0
mRNA	ID=cds1393	1473168-1475474	1	0	0
mRNA	ID=cds1411	1496675-1496899	1	0	0
mRNA	ID=cds1442	1524964-1525176	1	0	0
mRNA	ID=cds1444	1527946-1528428	1	0	0
mRNA	ID=cds1459	1544312-1545193	1	0	0
mRNA	ID=cds1463	1550422-1550706	1	0	0
mRNA	ID=cds1473	1558955-1560505	1	0	0
mRNA	ID=cds1484	1578866-1580548	1	0	0
mRNA	ID=cds1492	1588358-1588450	1	0	0
mRNA	ID=cds1528	1627477-1628937	1	0	0
mRNA	ID=cds1529	1629026-1630309	1	0	0
mRNA	ID=cds1544	1638394-1638609	1	0	0
mRNA	ID=cds1545	1639363-1639578	1	0	0
mRNA	ID=cds1556	1645382-1645660	1	0	0
mRNA	ID=cds1557	1645644-1645874	1	0	0
mRNA	ID=cds1603	1689610-1690875	1	0	0
mRNA	ID=cds1632	1719049-1719285	1	0	0
mRNA	ID=cds1639	1724683-1725780	1	0	0
mRNA	ID=cds1677	1768639-1768995	1	0	0
mRNA	ID=cds1680	1771813-1772679	1	0	0
mRNA	ID=cds1686	1778425-1779363	1	0	0
mRNA	ID=cds1687	1779419-1780708	1	0	0
mRNA	ID=cds1693	1787637-1787828	1	0	0
mRNA	ID=cds1712	1805424-1805714	1	0	0
mRNA	ID=cds1719	1811445-1811687	1	0	0
mRNA	ID=cds1721	1814410-1815159	1	0	0
mRNA	ID=cds1733	1824940-1826283	1	0	0
mRNA	ID=cds1759	1853015-1853995	1	0	0
mRNA	ID=cds1762	1855814-1856857	1	0	0
mRNA	ID=cds1783	1876797-1876895	1	0	0
mRNA	ID=cds1784	1876898-1876972	1	0	0
mRNA	ID=cds1786	1877427-1877609	1	0	0
mRNA	ID=cds1801	1892576-1892755	1	0	0
mRNA	ID=cds1810	1902825-1903283	1	0	0
mRNA	ID=cds1853	1944139-1944204	1	0	0
mRNA	ID=cds1860	1949419-1950237	1	0	0
mRNA	ID=cds1873	1964417-1965061	1	0	0
mRNA	ID=cds1874	1965072-1965461	1	0	0
mRNA	ID=cds1877	1967407-1969008	1	0	0
mRNA	ID=cds1878	1969054-1970715	1	0	0
mRNA	ID=cds1879	1970860-1971363	1	0	0
mRNA	ID=cds1881	1973353-1974279	1	0	0
mRNA	ID=cds1911	1999094-1999813	1	0	0
mRNA	ID=cds1919	2007503-2007736	1	0	0
mRNA	ID=cds1923	2010526-2010687	1	0	0
mRNA	ID=cds1924	2010724-2011038	1	0	0
mRNA	ID=cds1926	2012904-2013899	1	0	0
mRNA	ID=cds1929	2015970-2016413	1	0	0
mRNA	ID=cds1933	2019112-2019525	1	0	0
mRNA	ID=cds1954	2036980-2037393	1	0	0
mRNA	ID=cds1957	2039399-2040049	1	0	0
mRNA	ID=cds1961	2050300-2051352	1	0	0
mRNA	ID=cds1976	2066659-2066962	1	0	0
mRNA	ID=cds1979	2068684-2069235	1	0	0

mRNA	ID=cds1985	2075593-2075967	1	0	0
mRNA	ID=cds1987	2076573-2076701	1	0	0
mRNA	ID=cds2033	2121108-2122544	1	0	0
mRNA	ID=cds2034	2122547-2123770	1	0	0
mRNA	ID=cds2039	2126928-2127674	1	0	0
mRNA	ID=cds2045	2133679-2134122	1	0	0
mRNA	ID=cds2057	2150493-2151152	1	0	0
mRNA	ID=cds2083	2178117-2179121	1	0	0
mRNA	ID=cds2089	2183546-2183818	1	0	0
mRNA	ID=cds208	239106-239378	1	0	0
mRNA	ID=cds2094	2188948-2189667	1	0	0
mRNA	ID=cds2099	2197344-2198291	1	0	0
mRNA	ID=cds2122	2225345-2226631	1	0	0
mRNA	ID=cds2146	2252267-2253208	1	0	0
mRNA	ID=cds2149	2255451-2256389	1	0	0
mRNA	ID=cds2159	2266876-2267589	1	0	0
mRNA	ID=cds2186	2296291-2296740	1	0	0
mRNA	ID=cds218	247637-248134	1	0	0
mRNA	ID=cds2196	2306713-2307363	1	0	0
mRNA	ID=cds2209	2325389-2326165	1	0	0
mRNA	ID=cds2225	2352287-2353546	1	0	0
mRNA	ID=cds2231	2358231-2359436	1	0	0
mRNA	ID=cds224	252709-253161	1	0	0
mRNA	ID=cds2318	2448073-2448612	1	0	0
mRNA	ID=cds231	258269-259324	1	0	0
mRNA	ID=cds2329	2461034-2462092	1	0	0
mRNA	ID=cds2345	2473533-2473895	1	0	0
mRNA	ID=cds2350	2475869-2477206	1	0	0
mRNA	ID=cds2353	2480198-2481361	1	0	0
mRNA	ID=cds2364	2494943-2495014	1	0	0
mRNA	ID=cds2403	2538826-2539701	1	0	0
mRNA	ID=cds2407	2542774-2543631	1	0	0
mRNA	ID=cds2408	2543795-2544691	1	0	0
mRNA	ID=cds2419	2553763-2554422	1	0	0
mRNA	ID=cds2427	2560546-2561139	1	0	0
mRNA	ID=cds2433	2566346-2567533	1	0	0
mRNA	ID=cds2435	2568370-2569773	1	0	0
mRNA	ID=cds2439	2571524-2572327	1	0	0
mRNA	ID=cds2441	2573000-2573479	1	0	0
mRNA	ID=cds245	266408-267229	1	0	0
mRNA	ID=cds2466	2602833-2604272	1	0	0
mRNA	ID=cds247	268513-269406	1	0	0
mRNA	ID=cds2487	2627814-2628332	1	0	0
mRNA	ID=cds2516	2662412-2663266	1	0	0
mRNA	ID=cds2521	2668412-2668930	1	0	0
mRNA	ID=cds2528	2673849-2674847	1	0	0
mRNA	ID=cds254	276980-278026	1	0	0
mRNA	ID=cds2561	2713445-2714032	1	0	0
mRNA	ID=cds2600	2756666-2756878	1	0	0
mRNA	ID=cds2611	2768467-2768703	1	0	0
mRNA	ID=cds2618	2773941-2774399	1	0	0
mRNA	ID=cds2624	2781087-2781326	1	0	0
mRNA	ID=cds2639	2797672-2798016	1	0	0
mRNA	ID=cds2646	2804032-2805096	1	0	0
mRNA	ID=cds2660	2820161-2820661	1	0	0
mRNA	ID=cds2664	2823854-2824417	1	0	0
mRNA	ID=cds2667	2825759-2826538	1	0	0
mRNA	ID=cds2668	2826643-2827002	1	0	0
mRNA	ID=cds2686	2847260-2847871	1	0	0
mRNA	ID=cds2700	2861615-2862253	1	0	0
mRNA	ID=cds2714	2873443-2874351	1	0	0
mRNA	ID=cds2718	2877810-2878409	1	0	0
mRNA	ID=cds2726	2888121-2889920	1	0	0
mRNA	ID=cds2727	2890236-2890601	1	0	0
mRNA	ID=cds272	292444-293142	1	0	0
mRNA	ID=cds2733	2894555-2895892	1	0	0
mRNA	ID=cds277	296938-297015	1	0	0
mRNA	ID=cds2784	2961175-2961738	1	0	0
mRNA	ID=cds2814	2991961-2992404	1	0	0
mRNA	ID=cds2817	2993984-2994382	1	0	0
mRNA	ID=cds281	301108-301797	1	0	0
mRNA	ID=cds2820	2995714-2996850	1	0	0
mRNA	ID=cds2824	3001511-3001990	1	0	0
mRNA	ID=cds2844	3030839-3030964	1	0	0
mRNA	ID=cds2875	3062015-3062800	1	0	0
mRNA	ID=cds287	308582-309250	1	0	0
mRNA	ID=cds2894	3083942-3084073	1	0	0
mRNA	ID=cds2921	3108612-3109148	1	0	0
mRNA	ID=cds292	311738-312001	1	0	0
mRNA	ID=cds2938	3131266-3131979	1	0	0
mRNA	ID=cds2939	3132153-3132845	1	0	0
mRNA	ID=cds293	312365-312466	1	0	0
mRNA	ID=cds2940	3132894-3134393	1	0	0
mRNA	ID=cds2964	3156649-3156906	1	0	0
mRNA	ID=cds2995	3187903-3188652	1	0	0
mRNA	ID=cds3032	3232163-3232477	1	0	0
mRNA	ID=cds303	319451-320305	1	0	0
mRNA	ID=cds304	320832-321551	1	0	0
mRNA	ID=cds3058	3254701-3256032	1	0	0
mRNA	ID=cds3066	3265402-3265620	1	0	0
mRNA	ID=cds3067	3265876-3266415	1	0	0
mRNA	ID=cds3068	3266437-3267624	1	0	0
mRNA	ID=cds3078	3278239-3278712	1	0	0
mRNA	ID=cds3084	3282707-3283510	1	0	0
mRNA	ID=cds3086	3284292-3285047	1	0	0
mRNA	ID=cds3087	3285448-3286032	1	0	0
mRNA	ID=cds3108	3305875-3305955	1	0	0
mRNA	ID=cds3134	3334571-3334825	1	0	0
mRNA	ID=cds314	333749-334246	1	0	0
mRNA	ID=cds3158	3359198-3359962	1	0	0
mRNA	ID=cds3171	3372891-3374258	1	0	0
mRNA	ID=cds3186	3387156-3387359	1	0	0
mRNA	ID=cds3201	3405397-3405639	1	0	0
mRNA	ID=cds3214	3419347-3420450	1	0	0
mRNA	ID=cds322	343400-344215	1	0	0
mRNA	ID=cds325	345708-345983	1	0	0
mRNA	ID=cds3265	3459045-3459482	1	0	0
mRNA	ID=cds3266	3459490-3459999	1	0	0
mRNA	ID=cds3267	3459996-3460373	1	0	0
mRNA	ID=cds3269	3460946-3461929	1	0	0
mRNA	ID=cds3271	3463104-3463565	1	0	0
mRNA	ID=cds327	347906-348796	1	0	0
mRNA	ID=cds3287	3476614-3476814	1	0	0
mRNA	ID=cds3310	3500362-3501192	1	0	0
mRNA	ID=cds3314	3504054-3505358	1	0	0
mRNA	ID=cds3316	3505734-3506612	1	0	0
mRNA	ID=cds3329	3518637-3519041	1	0	0
mRNA	ID=cds334	358023-358682	1	0	0
mRNA	ID=cds335	358713-359183	1	0	0
mRNA	ID=cds3371	3573744-3575084	1	0	0
mRNA	ID=cds3376	3579161-3579649	1	0	0
mRNA	ID=cds337	360473-361084	1	0	0
mRNA	ID=cds3381	3582427-3582582	1	0	0
mRNA	ID=cds338	361150-362403	1	0	0
mRNA	ID=cds33	34781-35371	1	0	0
mRNA	ID=cds3407	3607240-3607905	1	0	0
mRNA	ID=cds3426	3631288-3632481	1	0	0
mRNA	ID=cds343	369501-370445	1	0	0
mRNA	ID=cds3457	3669315-3669917	1	0	0
mRNA	ID=cds3458	3670437-3671336	1	0	0
mRNA	ID=cds3462	3676443-3677210	1	0	0
mRNA	ID=cds3468	3687178-3688284	1	0	0
mRNA	ID=cds3472	3694020-3694208	1	0	0
mRNA	ID=cds3476	3698003-3698110	1	0	0
mRNA	ID=cds3489	3714570-3715229	1	0	0
mRNA	ID=cds348	374683-375894	1	0	0
mRNA	ID=cds3503	3725430-3725771	1	0	0
mRNA	ID=cds3506	3729154-3730146	1	0	0

mRNA	ID=cds3515	3740756-3741754	1	0	0
mRNA	ID=cds3522	3747255-3748115	1	0	0
mRNA	ID=cds3524	3748836-3748937	1	0	0
mRNA	ID=cds3529	3752128-3752451	1	0	0
mRNA	ID=cds3585	3809914-3810582	1	0	0
mRNA	ID=cds3592	3815783-3816607	1	0	0
mRNA	ID=cds3609	3838238-3838531	1	0	0
mRNA	ID=cds3618	3848825-3849115	1	0	0
mRNA	ID=cds3625	3853983-3854330	1	0	0
mRNA	ID=cds3628	3856424-3858139	1	0	0
mRNA	ID=cds362	387019-387870	1	0	0
mRNA	ID=cds3638	3867400-3868464	1	0	0
mRNA	ID=cds364	389475-390932	1	0	0
mRNA	ID=cds3664	3894797-3895462	1	0	0
mRNA	ID=cds3669	3898627-3900243	1	0	0
mRNA	ID=cds36	37898-39115	1	0	0
mRNA	ID=cds3760	4006462-4007082	1	0	0
mRNA	ID=cds3796	4051670-4051780	1	0	0
mRNA	ID=cds3801	4058470-4059180	1	0	0
mRNA	ID=cds3803	4060270-4061535	1	0	0
mRNA	ID=cds3807	4065263-4067299	1	0	0
mRNA	ID=cds3811	4070698-4071594	1	0	0
mRNA	ID=cds3819	4077774-4077992	1	0	0
mRNA	ID=cds3879	4144281-4145159	1	0	0
mRNA	ID=cds3886	4154036-4154812	1	0	0
mRNA	ID=cds3910	4190660-4190860	1	0	0
mRNA	ID=cds3954	4248726-4248908	1	0	0
mRNA	ID=cds3954	4248984-4250306	1	0	0
mRNA	ID=cds3998	4299050-4301101	1	0	0
mRNA	ID=cds4006	4309130-4310065	1	0	0
mRNA	ID=cds4011	4313127-4313561	1	0	0
mRNA	ID=cds4014	4315238-4315918	1	0	0
mRNA	ID=cds4019	4319267-4319719	1	0	0
mRNA	ID=cds401	429829-430176	1	0	0
mRNA	ID=cds4020	4319720-4320445	1	0	0
mRNA	ID=cds4045	4350108-4350380	1	0	0
mRNA	ID=cds4084	4390915-4390968	1	0	0
mRNA	ID=cds4116	4420869-4421723	1	0	0
mRNA	ID=cds4128	4429344-4430006	1	0	0
mRNA	ID=cds4166	4471363-4472076	1	0	0
mRNA	ID=cds4167	4472147-4472740	1	0	0
mRNA	ID=cds4168	4472885-4473337	1	0	0
mRNA	ID=cds4169	4473460-4475274	1	0	0
mRNA	ID=cds4170	4475330-4476334	1	0	0
mRNA	ID=cds4173	4477753-4478949	1	0	0
mRNA	ID=cds4182	4490610-4491374	1	0	0
mRNA	ID=cds4189	4498557-4498814	1	0	0
mRNA	ID=cds4199	4506699-4506965	1	0	0
mRNA	ID=cds4203	4510434-4511432	1	0	0
mRNA	ID=cds4215	4524129-4524911	1	0	0
mRNA	ID=cds4220	4528278-4528556	1	0	0
mRNA	ID=cds4222	4530073-4530333	1	0	0
mRNA	ID=cds4224	4531262-4531807	1	0	0
mRNA	ID=cds4227	4533026-4534054	1	0	0
mRNA	ID=cds4229	4535682-4536788	1	0	0
mRNA	ID=cds4230	4536808-4537524	1	0	0
mRNA	ID=cds4238	4546308-4546811	1	0	0
mRNA	ID=cds4259	4569774-4569938	1	0	0
mRNA	ID=cds4264	4577522-4577863	1	0	0
mRNA	ID=cds4280	4599647-4600120	1	0	0
mRNA	ID=cds457	489509-490036	1	0	0
mRNA	ID=cds491	528724-528816	1	0	0
mRNA	ID=cds492	528884-529240	1	0	0
mRNA	ID=cds501	538371-539732	1	0	0
mRNA	ID=cds512	551814-552323	1	0	0
mRNA	ID=cds519	557435-557977	1	0	0
mRNA	ID=cds520	558197-558889	1	0	0
mRNA	ID=cds532	567538-567870	1	0	0
mRNA	ID=cds541	572953-573093	1	0	0
mRNA	ID=cds543	573752-573809	1	0	0
mRNA	ID=cds546	576836-577333	1	0	0
mRNA	ID=cds549	577823-578116	1	0	0
mRNA	ID=cds54	58474-59052	1	0	0
mRNA	ID=cds550	578407-578817	1	0	0
mRNA	ID=cds551	579103-579309	1	0	0
mRNA	ID=cds554	580577-580885	1	0	0
mRNA	ID=cds589	624108-625283	1	0	0
mRNA	ID=cds590	625293-626903	1	0	0
mRNA	ID=cds593	628523-628936	1	0	0
mRNA	ID=cds599	634572-635792	1	0	0
mRNA	ID=cds613	649710-650006	1	0	0
mRNA	ID=cds642	678075-678629	1	0	0
mRNA	ID=cds666	709013-709339	1	0	0
mRNA	ID=cds676	715944-716093	1	0	0
mRNA	ID=cds683	724211-726259	1	0	0
mRNA	ID=cds717	765207-767183	1	0	0
mRNA	ID=cds71	80867-81958	1	0	0
mRNA	ID=cds756	809604-810758	1	0	0
mRNA	ID=cds786	841019-841279	1	0	0
mRNA	ID=cds802	858436-859251	1	0	0
mRNA	ID=cds830	890136-890423	1	0	0
mRNA	ID=cds834	893007-894119	1	0	0
mRNA	ID=cds835	894214-895347	1	0	0
mRNA	ID=cds969	1050186-1050398	1	0	0
mRNA	ID=cds986	1067304-1067477	1	0	0
mRNA	ID=cds988	1069083-1069577	1	0	0
mRNA	ID=cds990	1070188-1070988	1	0	0
mRNA	ID=cds991	1070996-1071382	1	0	0
mRNA	ID=cds992	1071394-1072086	1	0	0
mRNA	ID=cds237	263250-263471	0.918644	0	0
mRNA	ID=cds2620	2774899-2775099	0.918644	0	0
repeat_region		2224407-2224490	0.738471	0	0
repeat_region		4372521-4372619	0.709129	0	0
mRNA	ID=cds302	317900-319225	0.666666	0	0
repeat_region		3267744-3267841	0.567933	0	0.1685
repeat_region		1689449-1689545	0.507467	0	0.047004
mRNA	ID=cds1875	1965476-1966525	0.5	0	1
mgeRNA	gbkey=mobile_element	4507030-4507824	0.5	0	0
mRNA	ID=cds1152	1222894-1223130	0.5	0	0
mRNA	ID=cds1374	1451951-1452880	0.5	0	0
mRNA	ID=cds1376	1453188-1453934	0.5	0	0
mRNA	ID=cds146	169778-170575	0.5	0	0
mRNA	ID=cds1534	1633864-1634391	0.5	0	0
mRNA	ID=cds1535	1634780-1635013	0.5	0	0
mRNA	ID=cds1679	1770536-1771801	0.5	0	0
mRNA	ID=cds2029	2115148-2116428	0.5	0	0
mRNA	ID=cds2320	2449094-2449606	0.5	0	0
mRNA	ID=cds2359	2490026-2491276	0.5	0	0
mRNA	ID=cds2666	2825384-2825755	0.5	0	0
mRNA	ID=cds2739	2903579-2903719	0.5	0	0
mRNA	ID=cds3997	4297587-4299053	0.5	0	0
mRNA	ID=cds4197	4507032-4507816	0.5	0	0
mRNA	ID=cds433	463161-463532	0.5	0	0
mRNA	ID=cds553	580057-580602	0.5	0	0
mRNA	ID=cds583	617477-618610	0.5	0	0
repeat_region		360432-360460	0.414237	0	0
mRNA	ID=cds1156	1225823-1226191	0.379777	0	0
repeat_region		2254060-2254096	0.353553	0	0
mRNA	ID=cds206	238257-238736	0.353553	0	0
mRNA	ID=cds3394	3594474-3595583	0.353553	0	0
mRNA	ID=cds40	43188-44129	0.353553	0	0
mRNA	ID=cds360	385431-386198	0.333333	0	11
mRNA	ID=cds2619	2774408-2774890	0.333333	0	0
mRNA	ID=cds4275	4594013-4595035	0.299572	0	0
mRNA	ID=cds1678	1769095-1770309	0.29756	0	0
mRNA	ID=cds3013	3206046-3207509	0.291386	0	0
mRNA	ID=cds522	561559-562542	0.288675	0	0
mRNA	ID=cds2236	2363040-2363642	0.282038	0	0

mRNA	ID=cds643	678731-679438	0.25	0	0
mRNA	ID=cds2256	2383882-2384853	0.233974	0	0
mRNA	ID=cds316	335149-336012	0.188982	0	0
mRNA	ID=cds2887	3074201-3075478	0.181818	0	0
repeat_region		3486925-3486980	0.176777	0	0
mRNA	ID=cds1362	1433784-1434917	0.166181	0	1
mRNA	ID=cds1688	1780705-1780998	0.132686	0	0
mRNA	ID=cds4021	4320828-4321253	0.109812	0	0
repeat_region		1179577-1179672	0.097632	0	0
mRNA	ID=cds675	715170-715820	0.090963	0	0
mRNA	ID=cds3161	3364773-3364951	0.070923	0	0
repeat_region		1380891-1380964	0.062075	0	0
mRNA	ID=cds4207	4515737-4516258	0.05844	0	0
repeat_region		2595695-2595740	0.054466	0	0.20935
repeat_region		4486528-4486563	0.053645	0	0
repeat_region		2144624-2144707	0.048171	0	0
repeat_region		3250087-3250184	0.044878	0	0
mRNA	ID=cds3833	4094002-4095471	0.040213	0	0
repeat_region		4321269-4321355	0.030646	0	0
repeat_region		2826583-2826611	0.022503	0	0
repeat_region		361107-361140	0.011331	0	0
repeat_region		3706295-3706380	0.007289	0	0.008913
repeat_region		1355383-1355416	0.002282	0	1.020031
mRNA	ID=cds60	68348-70048	0	0	4
mRNA	ID=cds1368	1443711-1443896	0	0	2
mRNA	ID=cds1896	1986246-1986569	0	0	2
mRNA	ID=cds1792	1882689-1883813	0	0	1.5
mRNA	ID=cds1113	1196090-1196755	0	0	1
mRNA	ID=cds1115	1197918-1198811	0	0	1
mRNA	ID=cds1128	1205366-1206145	0	0	1
mRNA	ID=cds1218	1293649-1294215	0	0	1
mRNA	ID=cds1264	1341134-1341352	0	0	1
mRNA	ID=cds1302	1379971-1380876	0	0	1
mRNA	ID=cds1470	1556055-1557041	0	0	1
mRNA	ID=cds1725	1817880-1819238	0	0	1
mRNA	ID=cds1760	1854005-1854952	0	0	1
mRNA	ID=cds2042	2130091-2130579	0	0	1
mRNA	ID=cds2044	2131514-2133676	0	0	1
mRNA	ID=cds240	264528-264767	0	0	1
mRNA	ID=cds250	272071-273178	0	0	1
mRNA	ID=cds2720	2879073-2880164	0	0	1
mRNA	ID=cds3053	3250933-3251289	0	0	1
mRNA	ID=cds3085	3283500-3284291	0	0	1
mRNA	ID=cds4104	4410410-4411048	0	0	1
mRNA	ID=cds4105	4411051-4412214	0	0	1
mRNA	ID=cds4274	4592960-4593874	0	0	1
mRNA	ID=cds449	478591-479142	0	0	1
repeat_region		3839899-3839967	0	0	0.256495
repeat_region		3279724-3279805	0	0	0.071629
mRNA	ID=cds2434	2567523-2568359	0	0	0.015848
