# Supplementary material for: The extracellular RNA complement of Escherichia coli
Source: Microbiologyopen. 2015 Jan 21;4(2):252–66. doi: 10.1002/mbo3.235 (PMC4398507; doi:10.1002/mbo3.235)
Supplement: Supplementary file 5 — Table S4. Unique biotypes represented by RNAex. [file mbo30004-0252-sd5.pdf]

Supplementary table S4: Unique biotypes represented by RNA_{ex}

RNA biotype	Product information	Genomic coordinates	RNA_{exOMV} (Read Counts)	$RNA_{exOMV-f}$ (Read Counts)
mRNA	ID=cds705	748945-751392	4.5	0
mRNA	ID=cds1301	1378845-1379801	2	0
mRNA	ID=cds1301	1379801-1379926	1	0
mRNA	ID=cds1483	1577657-1578814	1	0
mRNA	ID=cds1490	1586333-1586863	1	0
mRNA	ID=cds2358	2488278-2489972	1	0
mRNA	ID=cds2440	2572324-2573025	1	0
mRNA	ID=cds2467	2604284-2604934	1	0
mRNA	ID=cds2811	2989290-2989781	1	0
mRNA	ID=cds300	316950-317543	1	0
mRNA	ID=cds3309	3499290-3500312	1	0
mRNA	ID=cds345	371339-372148	1	0
mRNA	ID=cds346	372145-373095	1	0
mRNA	ID=cds3528	3750986-3752122	1	0
mRNA	ID=cds4001	4302635-4304620	1	0
mRNA	ID=cds507	545904-547571	1	0
mRNA	ID=cds537	571689-572144	1	0
mRNA	ID=cds609	645854-646732	1	0
mRNA	ID=cds236	262914-263231	0.5	0
mRNA	ID=cds2721	2880177-2880659	0.5	0
mRNA	ID=cds3402	3603002-3603271	0.5	0
mRNA	ID=cds60	68348-70048	0	4
mRNA	ID=cds1368	1443711-1443896	0	2
mRNA	ID=cds1896	1986246-1986569	0	2
mRNA	ID=cds1792	1882689-1883813	0	1.5
mRNA	ID=cds1113	1196090-1196755	0	1
mRNA	ID=cds1115	1197918-1198811	0	1
mRNA	ID=cds1128	1205366-1206145	0	1
mRNA	ID=cds1218	1293649-1294215	0	1
mRNA	ID=cds1264	1341134-1341352	0	1
mRNA	ID=cds1302	1379971-1380876	0	1
mRNA	ID=cds1470	1556055-1557041	0	1
mRNA	ID=cds1725	1817880-1819238	0	1
mRNA	ID=cds1760	1854005-1854952	0	1
mRNA	ID=cds2042	2130091-2130579	0	1
mRNA	ID=cds2044	2131514-2133676	0	1
mRNA	ID=cds240	264528-264767	0	1
mRNA	ID=cds250	272071-273178	0	1
mRNA	ID=cds2720	2879073-2880164	0	1
mRNA	ID=cds3053	3250933-3251289	0	1
mRNA	ID=cds3085	3283500-3284291	0	1
mRNA	ID=cds4104	4410410-4411048	0	1
mRNA	ID=cds4105	4411051-4412214	0	1

mRNA	ID=cds4274	4592960-4593874	0	1
mRNA	ID=cds449	478591-479142	0	1
repeat_region		3839899-3839967	0	0.256495
repeat_region		3279724-3279805	0	0.071629
mRNA	ID=cds2434	2567523-2568359	0	0.015848
