# Supplementary material for: The extracellular RNA complement of Escherichia coli
Source: Microbiologyopen. 2015 Jan 21;4(2):252–66. doi: 10.1002/mbo3.235 (PMC4398507; doi:10.1002/mbo3.235)
Supplement: Supplementary file 6 — Table S5. Unique biotypes represented by RNAexOMV in comparison with RNAexOMV-f. [file mbo30004-0252-sd6.pdf]

Supplementary table S5: Unique biotypes represented by RNA_{exOMV} in comparision to $RNA_{exOMV-f}$

RNA biotype	Product information	Genomic coordinates	RNA_{exOMV} (Read Counts)
mRNA	ID=cds4150	4453808-4455181	57
mRNA	ID=cds285	304398-306041	42
mRNA	ID=cds2065	2162300-2163022	31
mRNA	ID=cds3738	3982375-3984030	30
mRNA	ID=cds411	437539-439401	15
mRNA	ID=cds4244	4553513-4554343	13
mRNA	ID=cds3544	3769405-3769767	7
mRNA	ID=cds1772	1866979-1868262	6
mRNA	ID=cds144	164730-167264	5
mRNA	ID=cds705	748945-751392	4.5
mRNA	ID=cds233	260727-261980	4
mRNA	ID=cds3469	3688291-3690630	4
Other ncRNA		16952-17006	3
mRNA	ID=cds82	93166-94653	3
mRNA	ID=cds163	189874-190599	3
mRNA	ID=cds2105	2207122-2209122	3
mRNA	ID=cds852	910405-911373	3
mRNA	ID=cds2549	2700503-2701408	2.5
mRNA	ID=cds472	506510-507304	2
mRNA	ID=cds15	16751-16960	2
mRNA	ID=cds3657	3886753-3888168	2
mRNA	ID=cds1868	1958086-1959819	2
mRNA	ID=cds3278	3471564-3472103	2
mRNA	ID=cds3114	3311364-3314036	2
mRNA	ID=cds1068	1148951-1149880	2
mRNA	ID=cds4094	4401323-4402327	2
mRNA	ID=cds2586	2743392-2743940	2
mRNA	ID=cds3434	3641163-3643205	2
mRNA	ID=cds2365	2495079-2496317	2
mRNA	ID=cds697	742050-742793	2
mRNA	ID=cds4152	4455982-4457334	2
mRNA	ID=cds2317	2447250-2448071	2
mRNA	ID=cds2565	2716757-2717176	2
mRNA	ID=cds2831	3010636-3012261	2
mRNA	ID=cds3920	4198589-4199284	2
mRNA	ID=cds1788	1878145-1878783	2
mRNA	ID=cds2536	2685491-2686825	2
mRNA	ID=cds3337	3527370-3527771	2
mRNA	ID=cds2474	2611956-2612804	2
mRNA	ID=cds4033	4333717-4335054	2
mRNA	ID=cds1339	1416695-1417183	2
mRNA	ID=cds1995	2082491-2083549	2
mRNA	ID=cds3317	3506609-3507835	2
mRNA	ID=cds3631	3860010-3861626	2
mRNA	ID=cds3805	4062386-4063789	2
mRNA	ID=cds1491	1586877-1588103	2
mRNA	ID=cds1984	2075136-2075504	2
mRNA	ID=cds1301	1378845-1379801	2
mRNA	ID=cds455	485760-489122	1.5
mRNA	ID=cds3916	4195739-4196803	1.5
mRNA	ID=cds3196	3401506-3402480	1.5
mRNA	ID=cds1086	1166822-1167361	1.5
mRNA	ID=cds2410	2546124-2547428	1.5
mRNA	ID=cds2465	2601869-2602816	1.5
mRNA	ID=cds4065	4372652-4373680	1
mRNA	ID=cds1600	1684755-1686401	1
mRNA	ID=cds174	202101-202556	1
mRNA	ID=cds1906	1995086-1995838	1
repeat_region		2835487-2835513	1
mRNA	ID=cds3367	3567369-3569342	1
repeat_region		2346803-2346838	1
repeat_region		2137726-2137762	1
mRNA	ID=cds3326	3515420-3516508	1
mRNA	ID=cds3241	3443266-3443619	1
mRNA	ID=cds1070	1150838-1151074	1
mRNA	ID=cds3822	4079880-4080782	1
mRNA	ID=cds3200	3403939-3405288	1
mRNA	ID=cds2606	2763940-2765013	1

mRNA	ID=cds2555	2706776-2707426	1
mRNA	ID=cds3341	3532538-3533890	1
mRNA	ID=cds3682	3915425-3916288	1
mRNA	ID=cds1306	1384744-1386285	1
mRNA	ID=cds1276	1352529-1353494	1
mRNA	ID=cds1224	1302778-1303791	1
mRNA	ID=cds4157	4462782-4464203	1
mRNA	ID=cds946	1028002-1029192	1
mRNA	ID=cds1713	1805820-1806680	1
mRNA	ID=cds400	428729-429700	1
mRNA	ID=cds3448	3655018-3655590	1
mRNA	ID=cds908	985117-986205	1
mRNA	ID=cds1770	1863750-1864496	1
mRNA	ID=cds2896	3084728-3085882	1
mRNA	ID=cds1602	1687876-1689384	1
mRNA	ID=cds1225	1303788-1304792	1
mRNA	ID=cds1752	1844989-1846032	1
mRNA	ID=cds4155	4458545-4460683	1
mRNA	ID=cds3112	3309855-3310799	1
mRNA	ID=cds2097	2191081-2192190	1
mRNA	ID=cds3652	3882516-3882875	1
mRNA	ID=cds3800	4056430-4058253	1
mRNA	ID=cds2489	2628980-2630557	1
mRNA	ID=cds3174	3375837-3376229	1
mRNA	ID=cds3987	4285787-4287223	1
mRNA	ID=cds3453	3663009-3663833	1
mRNA	ID=cds4072	4377030-4377389	1
mRNA	ID=cds3177	3378213-3378611	1
mRNA	ID=cds3870	4131858-4134038	1
mRNA	ID=cds3104	3300511-3301389	1
mRNA	ID=cds3235	3440137-3440493	1
mRNA	ID=cds1856	1945435-1946175	1
mRNA	ID=cds740	791539-793011	1
mRNA	ID=cds2284	2416656-2417198	1
mRNA	ID=cds482	516649-517503	1
mRNA	ID=cds1208	1284362-1285072	1
mRNA	ID=cds3488	3712084-3714417	1
mRNA	ID=cds2132	2234765-2235775	1
mRNA	ID=cds2184	2295043-2295666	1
mRNA	ID=cds2218	2345406-2346536	1
mRNA	ID=cds3156	3352747-3357207	1
mRNA	ID=cds1215	1290680-1291588	1
mRNA	ID=cds2706	2867535-2868296	1
mRNA	ID=cds3332	3519994-3520773	1
mRNA	ID=cds2881	3068187-3069266	1
mRNA	ID=cds160	185123-185947	1
mRNA	ID=cds3143	3340858-3341433	1
mRNA	ID=cds4121	4423862-4424089	1
mRNA	ID=cds941	1023694-1025748	1
mRNA	ID=cds424	451294-452769	1
mRNA	ID=cds658	696736-698400	1
mRNA	ID=cds1269	1345002-1346936	1
mRNA	ID=cds844	902229-902957	1
mRNA	ID=cds1180	1250289-1252208	1
mRNA	ID=cds1254	1331879-1332853	1
mRNA	ID=cds4043	4348054-4349685	1
mRNA	ID=cds1691	1785469-1786302	1
mRNA	ID=cds2607	2765006-2765377	1
mRNA	ID=cds3773	4020241-4020756	1
mRNA	ID=cds3901	4176470-4176898	1
mRNA	ID=cds2683	2842784-2844493	1
mRNA	ID=cds3655	3884851-3886215	1
mRNA	ID=cds4036	4338743-4339651	1
mRNA	ID=cds447	476291-477841	1
mRNA	ID=cds1304	1382141-1383538	1
mRNA	ID=cds1165	1232399-1233940	1
mRNA	ID=cds1912	2000134-2001630	1
mRNA	ID=cds2853	3038826-3039092	1
mRNA	ID=cds3449	3656389-3656916	1
mRNA	ID=cds3043	3244674-3245450	1
mRNA	ID=cds1903	1992727-1993383	1

mRNA	ID=cds3192	3396409-3396897	1
mRNA	ID=cds1029	1113487-1114713	1
mRNA	ID=cds1035	1118691-1119809	1
mRNA	ID=cds1209	1285072-1285749	1
mRNA	ID=cds140	160149-160604	1
mRNA	ID=cds1963	2053085-2054539	1
mRNA	ID=cds2637	2796113-2796517	1
mRNA	ID=cds398	426511-426843	1
mRNA	ID=cds563	590164-592401	1
mRNA	ID=cds1075	1154985-1155989	1
mRNA	ID=cds3142	3340295-3340861	1
mRNA	ID=cds847	904136-904966	1
mRNA	ID=cds3774	4020759-4021535	1
mRNA	ID=cds1194	1267388-1268242	1
mRNA	ID=cds120	141431-141967	1
mRNA	ID=cds420	446941-447270	1
mRNA	ID=cds1274	1350660-1351652	1
mRNA	ID=cds1421	1504805-1506766	1
mRNA	ID=cds202	234816-235538	1
mRNA	ID=cds867	930308-931273	1
mRNA	ID=cds180	209679-211820	1
mRNA	ID=cds3136	3335278-3335913	1
mRNA	ID=cds3767	4014454-4015215	1
mRNA	ID=cds746	796836-797654	1
mRNA	ID=cds3844	4105575-4106537	1
mRNA	ID=cds1327	1406074-1407057	1
mRNA	ID=cds1614	1702973-1703188	1
mRNA	ID=cds2475	2612842-2613903	1
mRNA	ID=cds2841	3026546-3027034	1
mRNA	ID=cds3710	3949565-3950227	1
mRNA	ID=cds3860	4119780-4120310	1
mRNA	ID=cds3896	4171105-4172070	1
repeat_region		1927947-1928030	1
mRNA	ID=cds1848	1939675-1940607	1
mRNA	ID=cds2793	2969619-2970659	1
mRNA	ID=cds2821	2997158-2997913	1
mRNA	ID=cds2989	3181835-3182488	1
mRNA	ID=cds3258	3450981-3451292	1
mRNA	ID=cds3312	3502074-3502805	1
mRNA	ID=cds1694	1787832-1789268	1
mRNA	ID=cds1962	2051667-2052983	1
mRNA	ID=cds3401	3602416-3603012	1
mRNA	ID=cds596	631612-632700	1
mRNA	ID=cds1030	1114885-1115805	1
mRNA	ID=cds148	171462-173444	1
mRNA	ID=cds157	182463-183620	1
mRNA	ID=cds1734	1826280-1827758	1
mRNA	ID=cds3160	3360829-3363210	1
mRNA	ID=cds4273	4591384-4592745	1
mRNA	ID=cds960	1041253-1043433	1
mRNA	ID=cds3551	3777077-3777853	1
mRNA	ID=cds1414	1498597-1499589	1
mRNA	ID=cds3663	3893295-3894632	1
mRNA	ID=cds386	409368-410276	1
mRNA	ID=cds632	667942-668259	1
mRNA	ID=cds2450	2585617-2588730	1
mRNA	ID=cds2128	2230900-2231619	1
mRNA	ID=cds2539	2689678-2693565	1
mRNA	ID=cds2563	2714776-2715465	1
mRNA	ID=cds3313	3502957-3504042	1
mRNA	ID=cds3995	4294459-4295148	1
mRNA	ID=cds4286	4604692-4605723	1
mRNA	ID=cds493	529356-530450	1
mRNA	ID=cds2520	2667054-2668415	1
mRNA	ID=cds3063	3261708-3263039	1
mRNA	ID=cds1325	1404003-1404566	1
mRNA	ID=cds1400	1485259-1486059	1
mRNA	ID=cds1715	1807404-1808072	1
mRNA	ID=cds1765	1859726-1859998	1
mRNA	ID=cds2635	2795233-2795532	1
mRNA	ID=cds2723	2882575-2885241	1

mRNA	ID=cds324	344890-345561	1
mRNA	ID=cds3504	3725940-3727394	1
mRNA	ID=cds350	376759-377592	1
mRNA	ID=cds3559	3783283-3784827	1
mRNA	ID=cds4031	4331305-4331973	1
mRNA	ID=cds4237	4545765-4546295	1
mRNA	ID=cds565	593983-594666	1
mRNA	ID=cds615	651458-653116	1
mRNA	ID=cds1356	1427073-1430435	1
mRNA	ID=cds3835	4096669-4097517	1
mRNA	ID=cds4258	4568185-4569597	1
mRNA	ID=cds3352	3544581-3545897	1
mRNA	ID=cds2286	2417863-2418507	1
mRNA	ID=cds1397	1478933-1480225	1
mRNA	ID=cds1907	1995835-1996503	1
mRNA	ID=cds1966	2057988-2058938	1
mRNA	ID=cds2171	2280962-2281969	1
mRNA	ID=cds2765	2935460-2936908	1
mRNA	ID=cds612	648805-649713	1
mRNA	ID=cds1407	1490494-1492134	1
mRNA	ID=cds1928	2014578-2015951	1
mRNA	ID=cds259	279651-279959	1
mRNA	ID=cds2876	3062824-3064302	1
mRNA	ID=cds2972	3166771-3167253	1
mRNA	ID=cds3033	3232761-3233897	1
mRNA	ID=cds3111	3309437-3309706	1
mRNA	ID=cds3387	3587205-3588050	1
mRNA	ID=cds353	379293-380066	1
mRNA	ID=cds3713	3951501-3953351	1
mRNA	ID=cds3876	4140553-4141632	1
mRNA	ID=cds4071	4375834-4376967	1
mRNA	ID=cds4232	4540060-4540656	1
mRNA	ID=cds605	641311-642549	1
mRNA	ID=cds68	77621-78799	1
mRNA	ID=cds742	793996-794145	1
mRNA	ID=cds1443	1525926-1527962	1
mRNA	ID=cds1509	1610349-1611275	1
mRNA	ID=cds1523	1622797-1623315	1
mRNA	ID=cds1931	2017642-2018106	1
mRNA	ID=cds2004	2089121-2090425	1
mRNA	ID=cds2103	2203717-2205996	1
mRNA	ID=cds3096	3295120-3296160	1
mRNA	ID=cds326	346081-347667	1
mRNA	ID=cds4265	4578091-4579485	1
mRNA	ID=cds4271	4587152-4589302	1
mRNA	ID=cds855	914575-915270	1
mRNA	ID=cds961	1043453-1043899	1
mRNA	ID=cds3802	4059188-4060168	1
mRNA	ID=cds1174	1243951-1244205	1
mRNA	ID=cds1494	1590200-1590466	1
mRNA	ID=cds2900	3089156-3089887	1
mRNA	ID=cds3029	3226910-3229261	1
mRNA	ID=cds312	331595-332683	1
mRNA	ID=cds3135	3334985-3335278	1
mRNA	ID=cds313	332725-333657	1
mRNA	ID=cds3298	3488288-3488851	1
mRNA	ID=cds3331	3519455-3519994	1
mRNA	ID=cds3507	3730224-3731765	1
mRNA	ID=cds3947	4238802-4240277	1
mRNA	ID=cds3028	3225823-3226893	1
mRNA	ID=cds1322	1399834-1401279	1
mRNA	ID=cds1360	1432982-1433032	1
mRNA	ID=cds1413	1497493-1498473	1
mRNA	ID=cds2194	2303130-2304776	1
mRNA	ID=cds2387	2523149-2523913	1
mRNA	ID=cds2918	3103736-3104992	1
mRNA	ID=cds2951	3144472-3144759	1
mRNA	ID=cds3523	3748109-3748804	1
mRNA	ID=cds35	36271-37824	1
mRNA	ID=cds3827	4087878-4088948	1
mRNA	ID=cds1301	1379801-1379926	1

mRNA	ID=cds1483	1577657-1578814	1
mRNA	ID=cds1490	1586333-1586863	1
mRNA	ID=cds2358	2488278-2489972	1
mRNA	ID=cds2440	2572324-2573025	1
mRNA	ID=cds2467	2604284-2604934	1
mRNA	ID=cds2811	2989290-2989781	1
mRNA	ID=cds300	316950-317543	1
mRNA	ID=cds3309	3499290-3500312	1
mRNA	ID=cds345	371339-372148	1
mRNA	ID=cds346	372145-373095	1
mRNA	ID=cds3528	3750986-3752122	1
mRNA	ID=cds4001	4302635-4304620	1
mRNA	ID=cds507	545904-547571	1
mRNA	ID=cds537	571689-572144	1
mRNA	ID=cds609	645854-646732	1
mRNA	ID=cds1090	1169741-1173187	0.5
mRNA	ID=cds2779	2950483-2954025	0.5
mRNA	ID=cds2397	2533856-2534365	0.5
mRNA	ID=cds3422	3623702-3624826	0.5
mRNA	ID=cds3727	3969283-3970545	0.5
mRNA	ID=cds2169	2278654-2280414	0.5
mRNA	ID=cds1675	1763653-1766709	0.5
mRNA	ID=cds4253	4561945-4562712	0.5
mRNA	ID=cds2311	2442225-2442773	0.5
mRNA	ID=cds1970	2062503-2063246	0.5
mRNA	ID=cds592	627774-628520	0.5
mRNA	ID=cds331	354146-355405	0.5
mRNA	ID=cds764	818271-818516	0.5
mRNA	ID=cds1892	1983163-1984152	0.5
mRNA	ID=cds318	337549-338967	0.5
mRNA	ID=cds234	262374-262436	0.5
mRNA	ID=cds3019	3213749-3214513	0.5
mRNA	ID=cds1793	1883869-1884834	0.5
mRNA	ID=cds1710	1803349-1804107	0.5
mRNA	ID=cds3082	3281165-3282025	0.5
mRNA	ID=cds347	373092-374105	0.5
mRNA	ID=cds566	594823-596196	0.5
mRNA	ID=cds339	362455-365529	0.5
mRNA	ID=cds236	262914-263231	0.5
mRNA	ID=cds2721	2880177-2880659	0.5
mRNA	ID=cds3402	3603002-3603271	0.5
mRNA	ID=cds1006	1093498-1094364	0.4
mRNA	ID=cds2074	2168556-2169422	0.4
mRNA	ID=cds297	314811-315677	0.4
mRNA	ID=cds365	390963-391829	0.4
mRNA	ID=cds531	566361-567227	0.4
mRNA	ID=cds745	795777-796835	0.333333
mRNA	ID=cds2674	2833195-2835447	0.333333
mRNA	ID=cds1891	1981579-1983093	0.333333
mRNA	ID=cds579	609477-611717	0.333333
mRNA	ID=cds220	250072-250827	0.129099
mRNA	ID=cds3537	3765244-3766188	0.116473
mRNA	ID=cds4204	4511429-4512331	0.083152
repeat_region		2591000-2591082	0.071429
repeat_region		3502808-3502843	0.071429
repeat_region		3752883-3752964	0.071429
mRNA	ID=cds3421	3622401-3623537	0.022125
mRNA	ID=cds1446	1529840-1530976	0.022125
mRNA	ID=cds692	736048-737184	0.022125
mRNA	ID=cds3520	3745107-3746603	0.010406
