# Supplementary material for: The extracellular RNA complement of Escherichia coli
Source: Microbiologyopen. 2015 Jan 21;4(2):252–66. doi: 10.1002/mbo3.235 (PMC4398507; doi:10.1002/mbo3.235)
Supplement: Supplementary file 7 — Table S6. Unique biotypes represented by RNAexOMV-f in comparison with RNAexOMV. [file mbo30004-0252-sd7.pdf]

Supplementary table S6: Unique biotypes represented by $RNA_{exOMV\text{-}f}$ in comparision to RNA_{exOMV}

RNA biotype	Product information	Genomic coordinates	RNA_{exOMV} (Read Counts)
Other ncRNA		2940718-2940923	73
Other ncRNA		2023251-2023337	63
Other ncRNA		3348599-3348719	47
Other ncRNA		4526000-4526089	20
repeat_region		500668-500751	19
mRNA	ID=cds3124	3325812-3326105	19
repeat_region		3302526-3302554	19
repeat_region		4616140-4616221	15.025102
mRNA	ID=cds2375	2507652-2508908	14
mRNA	ID=cds3931	4216619-4218355	13
mRNA	ID=cds477	513217-513624	13
mRNA	ID=cds3342	3533887-3534606	12
mRNA	ID=cds890	961218-962891	11
mRNA	ID=cds1307	1386329-1386835	11
mRNA	ID=cds1999	2086328-2087152	11
mRNA	ID=cds360	385431-386198	11
mRNA	ID=cds3295	3484142-3484774	10
mRNA	ID=cds3693	3925178-3926170	9
mRNA	ID=cds1889	1979611-1980411	9
mRNA	ID=cds950	1031362-1032480	8.121584
mRNA	ID=cds2271	2400077-2401867	8
mRNA	ID=cds3595	3819451-3820074	8
mRNA	ID=cds1363	1435284-1438808	8
mRNA	ID=cds1076	1156000-1156797	8
Other ncRNA		887199-887277	7
mRNA	ID=cds712	757929-760730	7
mRNA	ID=cds1109	1191890-1192996	7
mRNA	ID=cds2200	2311510-2314182	7
mRNA	ID=cds3245	3444921-3445460	7
mRNA	ID=cds121	142008-142670	7
mRNA	ID=cds1169	1238102-1239172	7
mRNA	ID=cds1630	1717900-1718367	7
mRNA	ID=cds2165	2274322-2275911	7
mRNA	ID=cds3216	3427258-3427812	7
mRNA	ID=cds4070	4375212-4375745	7
mRNA	ID=cds476	511800-513092	6
mRNA	ID=cds81	91413-93179	6
mRNA	ID=cds3957	4252066-4254489	6
mRNA	ID=cds1168	1236794-1238092	6
Other ncRNA		1489467-1489530	6
mRNA	ID=cds4066	4373722-4374288	6
mRNA	ID=cds3889	4157413-4158813	6
mRNA	ID=cds3109	3306062-3306946	6
mRNA	ID=cds161	185978-188650	6
mRNA	ID=cds3089	3286836-3289352	6
mRNA	ID=cds2915	3101035-3102087	6
mRNA	ID=cds478	513625-514083	6
mRNA	ID=cds3456	3667615-3669264	6
mRNA	ID=cds340	365652-366734	6
mRNA	ID=cds3437	3645728-3645856	6
Other ncRNA		1407153-1407274	5
mRNA	ID=cds3355	3551107-3553812	5
mRNA	ID=cds4276	4595173-4597464	5
mRNA	ID=cds430	458112-460466	5
mRNA	ID=cds2517	2663457-2664737	5
mRNA	ID=cds138	157729-159126	5
mRNA	ID=cds3247	3445800-3446171	5
mRNA	ID=cds1844	1934676-1935545	5
mRNA	ID=cds3547	3773595-3774182	5
mRNA	ID=cds3766	4013377-4014192	5
mRNA	ID=cds3275	3465182-3467875	5
repeat_region		4626718-4626867	4.821078
mRNA	ID=cds3001	3193163-3193222	4.5
mRNA	ID=cds862	922487-924763	4
mRNA	ID=cds3251	3447204-3447905	4
mRNA	ID=cds2281	2411492-2412694	4
mRNA	ID=cds1026	1110086-1112629	4
mRNA	ID=cds1257	1333855-1336530	4

mRNA	ID=cds4317	4637613-4638329	4
mRNA	ID=cds4140	4442135-4445914	4
mRNA	ID=cds2445	2577658-2579661	4
mRNA	ID=cds2396	2532088-2533815	4
mRNA	ID=cds1858	1946774-1948546	4
mRNA	ID=cds3975	4269072-4271894	4
mRNA	ID=cds933	1015175-1015693	4
mRNA	ID=cds1243	1319408-1320970	4
mRNA	ID=cds4091	4398311-4398619	4
mRNA	ID=cds92	105305-106456	4
mRNA	ID=cds3092	3291422-3293458	4
mRNA	ID=cds761	816267-817256	4
Other ncRNA		189712-189847	4
mRNA	ID=cds3224	3432236-3433183	4
mRNA	ID=cds1277	1353491-1355134	4
mRNA	ID=cds984	1066087-1066314	4
mRNA	ID=cds3902	4176902-4177606	4
mRNA	ID=cds3115	3314061-3315548	4
mRNA	ID=cds4082	4388480-4389532	4
mRNA	ID=cds799	854047-854967	4
mRNA	ID=cds3485	3710259-3710957	4
mRNA	ID=cds3586	3810754-3811974	4
mRNA	ID=cds4159	4465454-4465507	4
mRNA	ID=cds759	812749-814770	4
mRNA	ID=cds1188	1262100-1262723	4
mRNA	ID=cds629	664424-665536	4
mRNA	ID=cds2280	2410699-2411154	4
mRNA	ID=cds3856	4116538-4116783	4
mRNA	ID=cds3706	3946109-3946447	4
mRNA	ID=cds1599	1683209-1684612	4
mRNA	ID=cds2368	2499152-2500009	4
mRNA	ID=cds1659	1748369-1749037	4
mRNA	ID=cds2665	2824414-2825373	4
mRNA	ID=cds60	68348-70048	4
mRNA	ID=cds1399	1481085-1484987	3.666666
mRNA	ID=cds1811	1903712-1904278	3.5
Other ncRNA		2151668-2151803	3.278019
repeat_region		856839-856936	3.025102
mRNA	ID=cds710	755130-756896	3
mRNA	ID=cds2554	2705820-2706776	3
mRNA	ID=cds2498	2639853-2640866	3
mRNA	ID=cds713	760745-761962	3
mRNA	ID=cds1771	1864932-1866866	3
mRNA	ID=cds3110	3307055-3309190	3
mRNA	ID=cds2788	2964210-2966456	3
mRNA	ID=cds3005	3199229-3199849	3
mRNA	ID=cds3914	4194355-4194831	3
mRNA	ID=cds4098	4404677-4407118	3
mRNA	ID=cds475	510865-511797	3
mRNA	ID=cds3373	3575754-3576749	3
mRNA	ID=cds3451	3658437-3661550	3
mRNA	ID=cds1067	1147982-1148935	3
mRNA	ID=cds428	455901-456524	3
mRNA	ID=cds57	63429-65780	3
mgeRNA	gbkey=misc_feature	2556721-2563483	3
mRNA	ID=cds1671	1760546-1762033	3
mRNA	ID=cds2704	2865636-2866775	3
mRNA	ID=cds2574	2734168-2734905	3
mRNA	ID=cds1106	1188999-1189670	3
mRNA	ID=cds1980	2069563-2072682	3
mRNA	ID=cds2868	3054912-3054971	3
mRNA	ID=cds957	1038519-1039655	3
mRNA	ID=cds79	90094-91035	3
mRNA	ID=cds1942	2024347-2026041	3
mRNA	ID=cds638	674241-674723	3
mRNA	ID=cds1598	1682283-1683212	3
mRNA	ID=cds3649	3879244-3880344	3
mRNA	ID=cds4175	4481860-4482303	3
mRNA	ID=cds904	980270-982117	3
mRNA	ID=cds2758	2926251-2927540	3
mRNA	ID=cds981	1062078-1062998	3

mRNA	ID=cds3335	3524491-3526626	3
mRNA	ID=cds3742	3987848-3988789	3
mRNA	ID=cds1580	1664548-1665243	3
mRNA	ID=cds954	1035577-1035975	3
mRNA	ID=cds3154	3348711-3351047	3
mRNA	ID=cds451	479558-479932	3
mRNA	ID=cds1673	1762958-1763146	3
mRNA	ID=cds2854	3039335-3040315	3
mRNA	ID=cds432	461139-463010	3
mRNA	ID=cds3141	3339288-3340274	3
mRNA	ID=cds2315	2445530-2446462	3
mRNA	ID=cds4088	4394088-4395425	3
mRNA	ID=cds151	176610-176954	3
mRNA	ID=cds3016	3209129-3210874	3
mRNA	ID=cds3746	3992785-3993609	3
mRNA	ID=cds2825	3002030-3003808	3
mRNA	ID=cds414	440773-442221	3
mRNA	ID=cds3424	3627558-3628625	3
mRNA	ID=cds1862	1950726-1951469	3
mRNA	ID=cds1497	1598312-1599265	3
mRNA	ID=cds3891	4159794-4160153	3
mRNA	ID=cds699	743466-744398	3
mRNA	ID=cds817	877965-879080	3
mRNA	ID=cds1361	1433209-1433643	3
mRNA	ID=cds3861	4120403-4121362	3
mRNA	ID=cds897	970075-970821	3
mRNA	ID=cds107	120178-121551	3
mRNA	ID=cds4295	4612703-4613566	3
mRNA	ID=cds4218	4526134-4526940	3
mRNA	ID=cds623	658474-659439	3
mRNA	ID=cds3814	4073576-4074175	3
mRNA	ID=cds116	135598-136464	3
mRNA	ID=cds370	397096-398190	3
mRNA	ID=cds2592	2748853-2749731	3
mRNA	ID=cds826	886646-887182	3
mRNA	ID=cds2971	3166270-3166566	3
mRNA	ID=cds3578	3803966-3805090	3
mRNA	ID=cds1689	1781055-1782701	3
mRNA	ID=cds3630	3859196-3860010	3
mRNA	ID=cds2761	2929887-2931035	3
mRNA	ID=cds2193	2301927-2302415	3
mRNA	ID=cds2856	3041334-3041645	3
mRNA	ID=cds45	47769-49631	3
mRNA	ID=cds1454	1536874-1540614	3
mRNA	ID=cds2531	2677486-2680767	3
repeat_region		489183-489280	2.577781
mRNA	ID=cds3940	4231781-4233430	2.5
mRNA	ID=cds1653	1741481-1742854	2.5
Other ncRNA		3698159-3698224	2.409121
repeat_region		4482356-4482439	2.332944
mRNA	ID=cds2416	2551247-2552146	2.25
repeat_region		430192-430289	2.214239
repeat_region		3080712-3080858	2.109727
mgeRNA	gbkey=misc_feature	2464407-2474621	2.1066
mRNA	ID=cds2462	2598500-2598970	2.044093
mRNA	ID=cds2421	2555340-2556701	2
mRNA	ID=cds3929	4213501-4215102	2
Other ncRNA		606957-607015	2
mRNA	ID=cds2308	2438407-2439627	2
mRNA	ID=cds2514	2660605-2661345	2
mRNA	ID=cds4092	4398695-4399975	2
mRNA	ID=cds1319	1396798-1397550	2
mRNA	ID=cds3220	3429442-3429984	2
Other ncRNA		2698542-2698618	2
mRNA	ID=cds3683	3916339-3917880	2
mRNA	ID=cds3679	3911853-3913223	2
mRNA	ID=cds3681	3914016-3915398	2
mRNA	ID=cds3340	3530840-3532462	2
mRNA	ID=cds882	950495-952777	2
mRNA	ID=cds857	917351-918343	2
Other ncRNA		2165138-2165224	2

mRNA	ID=cds3678	3909862-3911691	2
mRNA	ID=cds3622	3851945-3853129	2
mRNA	ID=cds460	491316-493247	2
mRNA	ID=cds171	197928-200360	2
mRNA	ID=cds1071	1151162-1152403	2
mRNA	ID=cds4144	4447145-4447675	2
mRNA	ID=cds3368	3569339-3571525	2
mRNA	ID=cds1184	1258014-1258292	2
mRNA	ID=cds2693	2852360-2854438	2
mRNA	ID=cds3234	3439731-3440120	2
mRNA	ID=cds4048	4351223-4352740	2
mRNA	ID=cds2834	3014082-3017180	2
mRNA	ID=cds2173	2282398-2284158	2
mRNA	ID=cds3243	3444175-3444567	2
mRNA	ID=cds429	456650-457924	2
mRNA	ID=cds909	986808-988208	2
mRNA	ID=cds3327	3516565-3517086	2
mRNA	ID=cds3254	3448565-3449386	2
repeat_region		1355794-1355823	2
mRNA	ID=cds409	435813-436331	2
mRNA	ID=cds408	434858-435835	2
mRNA	ID=cds866	928419-930185	2
mRNA	ID=cds2282	2412769-2414913	2
mRNA	ID=cds3324	3513099-3513935	2
mRNA	ID=cds3888	4156513-4157430	2
mRNA	ID=cds1279	1355826-1357211	2
mRNA	ID=cds2513	2659665-2660153	2
mRNA	ID=cds4174	4479005-4481860	2
mRNA	ID=cds661	700826-701974	2
mRNA	ID=cds3510	3734376-3735200	2
mRNA	ID=cds3680	3913576-3913995	2
mRNA	ID=cds1186	1260151-1261098	2
mRNA	ID=cds2026	2111458-2112351	2
mRNA	ID=cds1235	1312044-1312682	2
mRNA	ID=cds893	965844-967592	2
mRNA	ID=cds3688	3920083-3920463	2
mRNA	ID=cds664	705316-706980	2
mRNA	ID=cds905	982298-982846	2
mRNA	ID=cds3399	3600102-3600770	2
mRNA	ID=cds4177	4484241-4485341	2
mRNA	ID=cds2273	2402651-2403094	2
mRNA	ID=cds2269	2398240-2399577	2
mRNA	ID=cds462	493629-494234	2
mRNA	ID=cds3490	3715333-3716307	2
mRNA	ID=cds1766	1860040-1860453	2
mRNA	ID=cds2559	2710918-2712252	2
mRNA	ID=cds1401	1486256-1487695	2
mRNA	ID=cds2496	2637323-2638597	2
mRNA	ID=cds3976	4272148-4272684	2
mRNA	ID=cds3720	3960768-3962252	2
mRNA	ID=cds2274	2403725-2404663	2
mRNA	ID=cds3138	3336488-3337270	2
mRNA	ID=cds3369	3571798-3572901	2
mRNA	ID=cds4060	4367179-4368435	2
Other ncRNA		4577858-4577934	2
mRNA	ID=cds2878	3065362-3066102	2
mRNA	ID=cds3741	3987111-3987851	2
mRNA	ID=cds1258	1336594-1337184	2
mRNA	ID=cds724	774376-775068	2
mRNA	ID=cds1846	1937246-1938217	2
mRNA	ID=cds396	424235-425305	2
mRNA	ID=cds766	819107-819811	2
mRNA	ID=cds3238	3442127-3442561	2
mRNA	ID=cds2501	2643035-2645347	2
mRNA	ID=cds985	1066335-1066931	2
mRNA	ID=cds3745	3992545-3992748	2
mRNA	ID=cds3758	4003887-4005716	2
mRNA	ID=cds3771	4018249-4019889	2
mRNA	ID=cds1702	1795983-1796966	2
mRNA	ID=cds2118	2221960-2222892	2
mRNA	ID=cds3145	3341966-3342691	2

mRNA	ID=cds729	778821-779612	2
mRNA	ID=cds2491	2632254-2633624	2
mRNA	ID=cds2573	2733053-2734033	2
mRNA	ID=cds3385	3585393-3586136	2
mRNA	ID=cds4151	4455337-4455888	2
mRNA	ID=cds1062	1144163-1145122	2
mRNA	ID=cds2327	2458672-2458956	2
mRNA	ID=cds2661	2820730-2821791	2
mRNA	ID=cds1642	1727111-1731727	2
mRNA	ID=cds2072	2169453-2169751	2
mRNA	ID=cds3015	3208803-3209018	2
mRNA	ID=cds3769	4016878-4017633	2
mRNA	ID=cds681	720953-723637	2
mRNA	ID=cds1040	1122630-1123277	2
mRNA	ID=cds1214	1289465-1290478	2
mRNA	ID=cds3181	3382725-3383195	2
mRNA	ID=cds1908	1996518-1997504	2
mRNA	ID=cds3103	3299507-3300502	2
mRNA	ID=cds1618	1704943-1707165	2
mRNA	ID=cds2533	2682276-2683529	2
mRNA	ID=cds2795	2971877-2974036	2
mRNA	ID=cds1941	2023535-2024350	2
mRNA	ID=cds2847	3033206-3034228	2
mRNA	ID=cds2983	3175303-3175932	2
mRNA	ID=cds4146	4449081-4450583	2
mRNA	ID=cds3147	3344195-3344482	2
mRNA	ID=cds2941	3134685-3136544	2
mRNA	ID=cds308	324801-326471	2
mRNA	ID=cds1465	1551996-1553693	2
repeat_region		1041208-1041241	2
mRNA	ID=cds3187	3387542-3388471	2
mRNA	ID=cds1042	1124785-1125369	2
mRNA	ID=cds3480	3701882-3702784	2
mRNA	ID=cds3168	3369106-3370596	2
mRNA	ID=cds3191	3395807-3396400	2
mRNA	ID=cds3403	3603274-3603633	2
mRNA	ID=cds3459	3671385-3672398	2
mRNA	ID=cds856	915696-917354	2
mRNA	ID=cds2477	2615600-2615959	2
mRNA	ID=cds849	906075-907505	2
mRNA	ID=cds2633	2794359-2794808	2
mRNA	ID=cds789	844964-845686	2
mRNA	ID=cds436	464836-466536	2
mRNA	ID=cds4303	4622168-4622812	2
mRNA	ID=cds1526	1626376-1627062	2
mRNA	ID=cds1571	1654208-1654768	2
mRNA	ID=cds1586	1669984-1670805	2
mRNA	ID=cds2071	2166736-2167635	2
mRNA	ID=cds2112	2213767-2214498	2
mRNA	ID=cds229	256527-257771	2
mRNA	ID=cds466	498238-499197	2
mRNA	ID=cds2133	2235791-2237311	2
mRNA	ID=cds2708	2869323-2869802	2
mRNA	ID=cds687	728806-732999	2
mRNA	ID=cds2174	2284412-2286936	2
mRNA	ID=cds2564	2715513-2716550	2
mRNA	ID=cds1171	1241389-1242303	2
mRNA	ID=cds926	1005714-1006823	2
mRNA	ID=cds2061	2153287-2156409	2
mRNA	ID=cds2306	2435972-2436967	2
mRNA	ID=cds2771	2940940-2941167	2
mRNA	ID=cds3353	3546008-3548092	2
mRNA	ID=cds1436	1517051-1518088	2
mRNA	ID=cds1504	1605370-1606128	2
mRNA	ID=cds3744	3991762-3992082	2
mRNA	ID=cds3855	4115268-4116113	2
mRNA	ID=cds387	410521-411705	2
mRNA	ID=cds389	414974-416176	2
mRNA	ID=cds2100	2198301-2201933	2
repeat_region		3010423-3010626	2
mRNA	ID=cds309	326485-327957	2

mRNA	ID=cds1647	1735480-1735569	2
mRNA	ID=cds2656	2814534-2814962	2
mRNA	ID=cds2692	2851276-2852286	2
mRNA	ID=cds3897	4172099-4173049	2
mRNA	ID=cds3978	4273494-4275080	2
mRNA	ID=cds815	875933-877258	2
mRNA	ID=cds3843	4104492-4105394	2
mRNA	ID=cds721	773419-773532	2
mRNA	ID=cds4119	4423141-4423536	2
mRNA	ID=cds156	180884-182308	2
mRNA	ID=cds2505	2653097-2654380	2
mRNA	ID=cds3558	3782607-3783038	2
mRNA	ID=cds3594	3817511-3819193	2
mRNA	ID=cds391	417113-418408	2
mRNA	ID=cds469	502700-503920	2
mRNA	ID=cds3491	3716357-3717067	2
mRNA	ID=cds31	30817-34038	2
mRNA	ID=cds134	152829-155426	2
mRNA	ID=cds2006	2091492-2092559	2
mRNA	ID=cds2088	2182535-2183323	2
mRNA	ID=cds3042	3243126-3244544	2
mRNA	ID=cds3543	3768266-3769402	2
mRNA	ID=cds694	738224-738733	2
mRNA	ID=cds1419	1502929-1504104	2
mRNA	ID=cds2285	2417256-2417810	2
mRNA	ID=cds284	303719-304429	2
mRNA	ID=cds4147	4450594-4451619	2
mgeRNA	gbkey=mobile_element	279338-279649	2
mRNA	ID=cds1139	1212551-1213282	2
mRNA	ID=cds1289	1367049-1367408	2
mRNA	ID=cds1546	1639879-1640091	2
mRNA	ID=cds1816	1906647-1906790	2
mRNA	ID=cds3097	3296233-3296868	2
mRNA	ID=cds3229	3436727-3437152	2
mRNA	ID=cds3962	4257511-4258026	2
mRNA	ID=cds4195	4504649-4504879	2
Other ncRNA		77367-77593	2
mRNA	ID=cds1915	2003737-2004102	2
mRNA	ID=cds258	279338-279586	2
mRNA	ID=cds271	291546-292172	2
mRNA	ID=cds3027	3224256-3225689	2
mRNA	ID=cds3260	3451951-3453420	2
mRNA	ID=cds395	423561-424142	2
mRNA	ID=cds4077	4381862-4383364	2
mRNA	ID=cds1123	1203045-1203383	2
mRNA	ID=cds1469	1555136-1556062	2
mRNA	ID=cds751	802726-804987	2
mRNA	ID=cds977	1057307-1058479	2
mRNA	ID=cds1368	1443711-1443896	2
mRNA	ID=cds1896	1986246-1986569	2
mRNA	ID=cds3498	3720351-3722420	1.666666
mRNA	ID=cds3751	3998315-3999079	1.639959
mRNA	ID=cds1792	1882689-1883813	1.5
repeat_region		4293858-4294457	1.255616
repeat_region		983584-983681	1.236427
repeat_region		3561605-3561702	1.181164
repeat_region		1999992-2000090	1.114819
repeat_region		1133809-1133906	1.114819
repeat_region		1734007-1734080	1.082189
repeat_region		444424-444510	1.02406
repeat_region		1355383-1355416	1.020031
repeat_region		353823-353993	1.013654
mRNA	ID=cds4233	4541138-4541686	1
mRNA	ID=cds1666	1755745-1756749	1
mRNA	ID=cds4059	4366687-4367163	1
mRNA	ID=cds3777	4023011-4024504	1
mRNA	ID=cds170	196546-197898	1
mRNA	ID=cds2499	2641151-2642305	1
mRNA	ID=cds178	205126-208608	1
mRNA	ID=cds3905	4179268-4183296	1
mRNA	ID=cds21	20815-21078	1

mRNA	ID=cds421	447270-447884	1
mRNA	ID=cds1583	1667723-1668976	1
mRNA	ID=cds3233	3439077-3439697	1
mRNA	ID=cds2882	3069481-3070644	1
mRNA	ID=cds4249	4558020-4558703	1
mRNA	ID=cds3847	4108763-4109530	1
repeat_region		4413981-4414014	1
mRNA	ID=cds1064	1146017-1146538	1
mRNA	ID=cds3894	4163451-4164308	1
mRNA	ID=cds3815	4074169-4075041	1
mRNA	ID=cds17	17489-18655	1
mRNA	ID=cds3852	4111749-4112495	1
mRNA	ID=cds412	439426-440325	1
mRNA	ID=cds4247	4556377-4557549	1
mRNA	ID=cds3423	3624826-3627561	1
mRNA	ID=cds2202	2315049-2317898	1
mRNA	ID=cds3400	3600773-3602266	1
mRNA	ID=cds3597	3820423-3822531	1
mRNA	ID=cds955	1035972-1036829	1
mRNA	ID=cds90	103155-103985	1
mRNA	ID=cds3296	3484813-3486915	1
mRNA	ID=cds3883	4148470-4151121	1
mRNA	ID=cds24	22391-25207	1
mRNA	ID=cds3647	3875728-3878142	1
mRNA	ID=cds3648	3878171-3879244	1
mRNA	ID=cds173	200971-201996	1
mRNA	ID=cds1993	2080780-2082207	1
mRNA	ID=cds3189	3390480-3394280	1
Other ncRNA		2151333-2151475	1
mRNA	ID=cds3100	3297988-3298290	1
mRNA	ID=cds3101	3298277-3298780	1
mRNA	ID=cds2109	2210981-2212666	1
mRNA	ID=cds846	903816-904139	1
mRNA	ID=cds3813	4072692-4073477	1
mRNA	ID=cds84	96002-97084	1
mRNA	ID=cds1508	1609990-1610349	1
mRNA	ID=cds660	699597-700817	1
mRNA	ID=cds1272	1349431-1349784	1
mRNA	ID=cds51	53416-54702	1
mRNA	ID=cds95	108279-110984	1
mRNA	ID=cds2691	2850158-2851279	1
mRNA	ID=cds4073	4377400-4377795	1
mRNA	ID=cds3723	3964440-3965699	1
mRNA	ID=cds2	2801-3733	1
mRNA	ID=cds2261	2388070-2389527	1
mRNA	ID=cds4028	4328525-4330027	1
mRNA	ID=cds2262	2389534-2391063	1
mRNA	ID=cds1066	1146844-1147914	1
mRNA	ID=cds2058	2151373-2151432	1
mRNA	ID=cds2594	2751627-2751968	1
mRNA	ID=cds673	712781-714421	1
mRNA	ID=cds2508	2655107-2656957	1
mRNA	ID=cds2747	2911721-2913022	1
mRNA	ID=cds3431	3638134-3638568	1
mRNA	ID=cds3450	3657255-3658412	1
mRNA	ID=cds782	836888-837148	1
mRNA	ID=cds1271	1348275-1349063	1
mRNA	ID=cds2864	3051537-3052862	1
mRNA	ID=cds91	103982-105244	1
mRNA	ID=cds2383	2517279-2518694	1
mRNA	ID=cds2423	2558279-2558920	1
mRNA	ID=cds732	783105-784046	1
mRNA	ID=cds3397	3597952-3598806	1
mRNA	ID=cds189	217057-218775	1
mRNA	ID=cds2495	2636685-2637305	1
mRNA	ID=cds419	446039-446929	1
mRNA	ID=cds3533	3757881-3759272	1
mRNA	ID=cds185	214291-214836	1
mRNA	ID=cds4080	4384070-4387393	1
mRNA	ID=cds3398	3599051-3600109	1
mRNA	ID=cds162	188712-189506	1

mRNA	ID=cds56	60358-63264	1
mRNA	ID=cds1317	1395389-1395646	1
mRNA	ID=cds2568	2720749-2722104	1
mRNA	ID=cds700	744388-745122	1
mRNA	ID=cds3429	3635665-3637164	1
mRNA	ID=cds3690	3921767-3923656	1
mRNA	ID=cds4318	4638425-4638565	1
mRNA	ID=cds1803	1894194-1894772	1
mRNA	ID=cds3700	3934301-3935191	1
mRNA	ID=cds809	866743-868614	1
mRNA	ID=cds85	97087-98403	1
mRNA	ID=cds2865	3052888-3053466	1
mRNA	ID=cds819	879950-881152	1
mRNA	ID=cds1664	1753722-1755134	1
mRNA	ID=cds2266	2393930-2394472	1
mRNA	ID=cds771	823853-824263	1
mRNA	ID=cds2833	3013182-3013760	1
mRNA	ID=cds3226	3434540-3435916	1
mRNA	ID=cds2911	3097704-3098750	1
mRNA	ID=cds2011	2095345-2096325	1
mRNA	ID=cds774	826468-828204	1
mRNA	ID=cds2389	2524968-2525966	1
mRNA	ID=cds3131	3331732-3332703	1
mRNA	ID=cds628	663325-664413	1
mRNA	ID=cds653	690129-691007	1
mRNA	ID=cds736	787020-788060	1
mRNA	ID=cds2392	2528269-2529255	1
mRNA	ID=cds1525	1625541-1626287	1
mRNA	ID=cds1597	1680906-1682207	1
mRNA	ID=cds3253	3448270-3448548	1
mRNA	ID=cds4306	4625338-4626570	1
mRNA	ID=cds715	763403-764272	1
repeat_region		176969-176987	1
mRNA	ID=cds1650	1737935-1739146	1
mRNA	ID=cds2946	3138814-3139308	1
mRNA	ID=cds3204	3408302-3409267	1
mRNA	ID=cds3366	3566056-3567351	1
mRNA	ID=cds3991	4289535-4291193	1
mRNA	ID=cds518	556098-556964	1
mRNA	ID=cds562	587205-590177	1
mRNA	ID=cds149	173602-174882	1
mRNA	ID=cds2503	2650516-2651361	1
mRNA	ID=cds3596	3820129-3820404	1
mRNA	ID=cds4171	4476496-4476912	1
mRNA	ID=cds2203	2318065-2319891	1
mRNA	ID=cds2272	2401973-2402635	1
mRNA	ID=cds3566	3792010-3792942	1
mRNA	ID=cds4106	4412298-4413923	1
mRNA	ID=cds772	824225-825331	1
mRNA	ID=cds859	919570-921516	1
mRNA	ID=cds3694	3926175-3927626	1
mRNA	ID=cds125	145081-146310	1
mRNA	ID=cds2604	2761559-2763175	1
mRNA	ID=cds3294	3483436-3483840	1
mRNA	ID=cds88	100765-102240	1
mRNA	ID=cds3179	3380222-3381289	1
mRNA	ID=cds1866	1956544-1957290	1
mRNA	ID=cds2067	2163692-2165053	1
mRNA	ID=cds2140	2245085-2246554	1
mRNA	ID=cds2632	2793696-2794358	1
mRNA	ID=cds1402	1487988-1488737	1
mRNA	ID=cds2572	2732325-2733056	1
mRNA	ID=cds2968	3161737-3163995	1
mRNA	ID=cds3149	3345137-3345991	1
mRNA	ID=cds496	532235-533050	1
mRNA	ID=cds3252	3447923-3448255	1
mRNA	ID=cds1227	1305209-1306669	1
mRNA	ID=cds214	245065-245805	1
mRNA	ID=cds2276	2406884-2407483	1
mRNA	ID=cds3512	3737728-3738981	1
mRNA	ID=cds4137	4437895-4439238	1

mRNA	ID=cds4139	4440405-4442138	1
mRNA	ID=cds4086	4392089-4393636	1
mRNA	ID=cds1212	1287897-1288355	1
mRNA	ID=cds1408	1492172-1493095	1
mRNA	ID=cds3602	3828480-3830189	1
mRNA	ID=cds543	575009-576048	1
mRNA	ID=cds4176	4482463-4483974	1
mRNA	ID=cds2862	3049137-3050339	1
mRNA	ID=cds2965	3156949-3159168	1
mRNA	ID=cds3130	3331162-3331473	1
mRNA	ID=cds3651	3882359-3882499	1
mRNA	ID=cds3761	4007193-4008215	1
mRNA	ID=cds3768	4015356-4016783	1
mRNA	ID=cds906	982873-983520	1
mRNA	ID=cds773	825342-826475	1
mRNA	ID=cds22	21181-21399	1
mRNA	ID=cds2787	2963184-2964059	1
mRNA	ID=cds3532	3756040-3757884	1
mRNA	ID=cds3580	3806563-3807840	1
mRNA	ID=cds3726	3968156-3969286	1
mRNA	ID=cds4156	4461077-4462732	1
mRNA	ID=cds735	786066-786818	1
mRNA	ID=cds795	850237-851820	1
mRNA	ID=cds851	908554-910272	1
mRNA	ID=cds62	71351-72115	1
mRNA	ID=cds1623	1710793-1712295	1
mRNA	ID=cds1612	1701292-1702332	1
mRNA	ID=cds119	138835-141225	1
mRNA	ID=cds172	200482-200967	1
mRNA	ID=cds2776	2945779-2947032	1
mRNA	ID=cds3739	3984709-3985905	1
mRNA	ID=cds731	782389-783108	1
mRNA	ID=cds760	814962-815870	1
mRNA	ID=cds820	881199-881957	1
mRNA	ID=cds3076	3275878-3276687	1
mRNA	ID=cds1083	1163318-1164343	1
mRNA	ID=cds1606	1694486-1695076	1
mRNA	ID=cds1676	1767098-1768210	1
mRNA	ID=cds2444	2576688-2577638	1
mRNA	ID=cds2515	2661464-2662267	1
mRNA	ID=cds3556	3781684-3782151	1
mRNA	ID=cds3752	3999449-4000399	1
mRNA	ID=cds388	411831-414977	1
mRNA	ID=cds439	468095-469867	1
mRNA	ID=cds1251	1327356-1328405	1
mRNA	ID=cds2161	2268748-2270304	1
mRNA	ID=cds2710	2870531-2870842	1
mRNA	ID=cds2768	2938165-2939265	1
mRNA	ID=cds646	682700-683635	1
mRNA	ID=cds3619	3849119-3850807	1
mRNA	ID=cds515	553834-555219	1
mRNA	ID=cds129	148807-149601	1
mRNA	ID=cds2013	2097886-2099292	1
mRNA	ID=cds2264	2393065-2393367	1
mRNA	ID=cds2548	2699763-2700491	1
mRNA	ID=cds3148	3344600-3345091	1
mRNA	ID=cds3199	3403458-3403928	1
mRNA	ID=cds3600	3825483-3826688	1
mRNA	ID=cds6	6529-7959	1
mRNA	ID=cds850	907516-908517	1
mRNA	ID=cds930	1012482-1014122	1
mRNA	ID=cds725	775072-775500	1
mRNA	ID=cds232	259612-260715	1
mRNA	ID=cds1625	1713050-1713913	1
mRNA	ID=cds2589	2745984-2746775	1
mRNA	ID=cds2709	2869802-2870512	1
mRNA	ID=cds3283	3473740-3474462	1
mRNA	ID=cds3866	4126101-4126418	1
mRNA	ID=cds1191	1265317-1266150	1
mRNA	ID=cds195	222833-223408	1
mRNA	ID=cds2542	2695937-2696572	1

mRNA	ID=cds3002	3193342-3194775	1
mRNA	ID=cds3705	3945151-3945990	1
mRNA	ID=cds3996	4295242-4297389	1
mRNA	ID=cds410	436385-437359	1
mRNA	ID=cds4290	4607437-4609026	1
mRNA	ID=cds980	1061773-1062078	1
mRNA	ID=cds1119	1200999-1201307	1
mRNA	ID=cds1402	1487737-1487988	1
mRNA	ID=cds1609	1697379-1698971	1
mRNA	ID=cds2018	2103089-2104081	1
mRNA	ID=cds2137	2241006-2241674	1
mRNA	ID=cds2265	2393364-2393918	1
mRNA	ID=cds2796	2974621-2975652	1
mRNA	ID=cds2827	3005532-3006728	1
mRNA	ID=cds4311	4631820-4632467	1
mgeRNA	gbkey=mobile_element	1293649-1294628	1
mRNA	ID=cds561	586314-587204	1
mRNA	ID=cds2848	3034395-3036128	1
mRNA	ID=cds126	146314-146694	1
mRNA	ID=cds1524	1623359-1625404	1
mRNA	ID=cds1808	1901106-1901906	1
mRNA	ID=cds2059	2151705-2151761	1
mRNA	ID=cds3689	3921080-3921703	1
mRNA	ID=cds3701	3935317-3936246	1
mRNA	ID=cds416	442828-443739	1
mRNA	ID=cds892	963543-965807	1
mRNA	ID=cds2728	2890679-2891950	1
mRNA	ID=cds1102	1183681-1184817	1
mRNA	ID=cds1433	1515672-1515905	1
mRNA	ID=cds1634	1720145-1722157	1
mRNA	ID=cds2585	2742594-2743361	1
mRNA	ID=cds3157	3357220-3358638	1
mRNA	ID=cds4135	4436731-4437285	1
mRNA	ID=cds465	497279-498241	1
mRNA	ID=cds5	5683-6459	1
mRNA	ID=cds738	789206-790252	1
mRNA	ID=cds983	1064808-1066049	1
repeat_region		2250818-2250908	1
mRNA	ID=cds1527	1627239-1627442	1
mRNA	ID=cds1573	1655589-1655894	1
mRNA	ID=cds1607	1695297-1696064	1
mRNA	ID=cds1807	1900072-1901043	1
mRNA	ID=cds2302	2432104-2432763	1
mRNA	ID=cds2395	2531786-2532043	1
mRNA	ID=cds2579	2738102-2739172	1
mRNA	ID=cds2640	2798156-2798497	1
mRNA	ID=cds2662	2821871-2822368	1
mRNA	ID=cds2792	2969293-2969511	1
mRNA	ID=cds2830	3009483-3010415	1
mRNA	ID=cds3334	3523611-3524171	1
mRNA	ID=cds3677	3908508-3909548	1
mRNA	ID=cds3884	4151719-4152870	1
mRNA	ID=cds4269	4585932-4586888	1
mRNA	ID=cds479	514080-514997	1
mRNA	ID=cds776	829195-829866	1
mRNA	ID=cds881	949563-950303	1
mRNA	ID=cds3414	3614205-3615038	1
mRNA	ID=cds1101	1182840-1183667	1
mRNA	ID=cds1619	1707166-1708224	1
mRNA	ID=cds1720	1811891-1814152	1
mRNA	ID=cds2390	2525963-2526181	1
mRNA	ID=cds2596	2752310-2752786	1
mRNA	ID=cds2670	2827835-2828800	1
mRNA	ID=cds2676	2836276-2837286	1
mRNA	ID=cds2850	3036869-3037765	1
mRNA	ID=cds4189	4497616-4498557	1
mRNA	ID=cds4302	4621124-4622140	1
mRNA	ID=cds444	474603-475175	1
mRNA	ID=cds3546	3772447-3773595	1
mRNA	ID=cds1329	1409037-1409972	1
mRNA	ID=cds1965	2056227-2057714	1

mRNA	ID=cds2003	2088216-2089115	1
mRNA	ID=cds2110	2212888-2213619	1
mRNA	ID=cds2248	2374856-2375614	1
mRNA	ID=cds2251	2378744-2379049	1
mRNA	ID=cds3046	3246991-3247359	1
mRNA	ID=cds3590	3813886-3814572	1
mRNA	ID=cds3783	4031168-4032619	1
mRNA	ID=cds4097	4404213-4404638	1
mRNA	ID=cds4301	4619792-4621123	1
mRNA	ID=cds61	70387-71265	1
mRNA	ID=cds1745	1837491-1838798	1
mRNA	ID=cds2957	3149272-3150006	1
mRNA	ID=cds2135	2238650-2239690	1
mRNA	ID=cds204	236067-236798	1
mRNA	ID=cds2297	2426743-2428260	1
mRNA	ID=cds2378	2511064-2512266	1
mRNA	ID=cds2415	2550374-2551243	1
mRNA	ID=cds2770	2939672-2940589	1
mRNA	ID=cds2840	3025143-3026510	1
mRNA	ID=cds2885	3072708-3073217	1
mRNA	ID=cds3095	3294431-3295006	1
mRNA	ID=cds3477	3698586-3699857	1
mRNA	ID=cds3545	3770304-3772217	1
mRNA	ID=cds3736	3978910-3980295	1
mRNA	ID=cds3841	4102995-4103693	1
mRNA	ID=cds3939	4229907-4231256	1
mRNA	ID=cds564	592551-593993	1
mRNA	ID=cds747	797809-798804	1
mRNA	ID=cds779	832293-834443	1
mRNA	ID=cds3732	3974301-3975551	1
mRNA	ID=cds2062	2156410-2159487	1
mgeRNA	gbkey=rep_origin	3923767-3923998	1
mRNA	ID=cds1819	1908300-1909673	1
mRNA	ID=cds1887	1977777-1978205	1
mRNA	ID=cds2239	2366061-2368043	1
mRNA	ID=cds2278	2409461-2410111	1
mRNA	ID=cds2486	2627312-2627503	1
mRNA	ID=cds2832	3012309-3013079	1
mRNA	ID=cds2843	3029389-3030837	1
mRNA	ID=cds3075	3275359-3275823	1
mRNA	ID=cds3218	3428042-3428860	1
mRNA	ID=cds3410	3609888-3610937	1
mRNA	ID=cds3411	3610992-3611579	1
mRNA	ID=cds3530	3752996-3754534	1
mRNA	ID=cds781	835574-836659	1
mRNA	ID=cds4069	4374898-4375215	1
mRNA	ID=cds3719	3958700-3960721	1
repeat_region		2579670-2579738	1
mRNA	ID=cds1364	1439082-1439348	1
mRNA	ID=cds145	167484-169727	1
mRNA	ID=cds1805	1896451-1898049	1
mRNA	ID=cds1916	2004180-2005667	1
mRNA	ID=cds2374	2506483-2507448	1
mRNA	ID=cds2580	2739382-2739747	1
mRNA	ID=cds2715	2874603-2875640	1
mRNA	ID=cds2908	3094703-3095296	1
mRNA	ID=cds2958	3150258-3151445	1
mRNA	ID=cds3262	3454399-3456351	1
mRNA	ID=cds3339	3528737-3530461	1
mRNA	ID=cds3406	3606774-3607019	1
mRNA	ID=cds3493	3718072-3718284	1
mRNA	ID=cds3882	4146555-4148288	1
mRNA	ID=cds4239	4546831-4547733	1
mRNA	ID=cds4243	4552599-4553372	1
mRNA	ID=cds4304	4622918-4623886	1
mRNA	ID=cds634	669154-669795	1
mRNA	ID=cds80	91032-91397	1
mRNA	ID=cds931	1014119-1014682	1
mRNA	ID=cds443	473525-474385	1
mRNA	ID=cds438	467607-468065	1
repeat_region		4054494-4054578	1

mRNA	ID=cds1024	1107007-1108164	1
mRNA	ID=cds1118	1200720-1201061	1
mRNA	ID=cds1192	1266147-1266539	1
mRNA	ID=cds1518	1618262-1619161	1
mRNA	ID=cds1669	1758544-1759815	1
mRNA	ID=cds1898	1987275-1987514	1
mRNA	ID=cds1948	2030408-2031103	1
mRNA	ID=cds1969	2061412-2062491	1
mRNA	ID=cds2019	2104084-2105250	1
mRNA	ID=cds2469	2606509-2608176	1
mRNA	ID=cds305	321562-322989	1
mRNA	ID=cds3117	3316659-3318002	1
mRNA	ID=cds3435	3643408-3644250	1
mRNA	ID=cds3513	3739132-3739605	1
mRNA	ID=cds718	767201-769834	1
mRNA	ID=cds902	974845-975549	1
mRNA	ID=cds925	1005175-1005717	1
mRNA	ID=cds1427	1510841-1511854	1
mRNA	ID=cds3358	3556290-3557888	1
repeat_region		3588956-3589021	1
mRNA	ID=cds1022	1105043-1105576	1
mRNA	ID=cds1063	1145234-1145818	1
mRNA	ID=cds1072	1152523-1153332	1
mRNA	ID=cds114	134388-134750	1
mRNA	ID=cds1236	1312742-1313248	1
mRNA	ID=cds1291	1367713-1368027	1
mRNA	ID=cds1353	1424478-1425410	1
mRNA	ID=cds1572	1654771-1655481	1
mRNA	ID=cds1790	1879936-1881021	1
mRNA	ID=cds1837	1927072-1927731	1
mRNA	ID=cds2001	2087486-2087737	1
mRNA	ID=cds2081	2175534-2176586	1
mRNA	ID=cds2753	2921024-2921806	1
mRNA	ID=cds3022	3217516-3218895	1
mRNA	ID=cds3913	4192227-4194122	1
mRNA	ID=cds4153	4457513-4457878	1
mRNA	ID=cds4287	4605826-4606239	1
mRNA	ID=cds559	583903-584856	1
mRNA	ID=cds703	747144-748205	1
mgeRNA	gbkey=mobile_element	4516495-4517262	1
mRNA	ID=cds1000	1084215-1085279	1
mRNA	ID=cds1428	1511855-1512796	1
mRNA	ID=cds1728	1820482-1821309	1
mRNA	ID=cds1827	1919804-1920040	1
mRNA	ID=cds2060	2152040-2153287	1
mRNA	ID=cds2138	2241932-2242768	1
mRNA	ID=cds2241	2368930-2370582	1
mRNA	ID=cds2369	2500012-2502507	1
mRNA	ID=cds2578	2736970-2738091	1
mRNA	ID=cds266	288525-289529	1
mRNA	ID=cds2791	2968442-2969155	1
mRNA	ID=cds279	297960-300158	1
mRNA	ID=cds2954	3146999-3147493	1
mRNA	ID=cds3051	3249046-3250032	1
mRNA	ID=cds3125	3326261-3326737	1
mRNA	ID=cds3413	3613264-3614208	1
mRNA	ID=cds3776	4022356-4022844	1
mRNA	ID=cds3793	4048156-4048788	1
mRNA	ID=cds3824	4084039-4084872	1
mRNA	ID=cds4083	4389627-4390172	1
mRNA	ID=cds4314	4634030-4634719	1
mRNA	ID=cds778	831691-832173	1
mRNA	ID=cds3911	4190844-4191599	1
mRNA	ID=cds1044	1126029-1126952	1
mRNA	ID=cds1198	1269972-1271072	1
mRNA	ID=cds1201	1272469-1272822	1
mRNA	ID=cds1638	1724047-1724646	1
mRNA	ID=cds1800	1892157-1892456	1
mRNA	ID=cds2119	2223066-2223653	1
mRNA	ID=cds216	246242-246502	1
mRNA	ID=cds2332	2464567-2465724	1

mRNA	ID=cds2471	2608728-2609486	1
mRNA	ID=cds2598	2754181-2755422	1
mRNA	ID=cds2711	2871036-2871359	1
mRNA	ID=cds2823	3000636-3001514	1
mRNA	ID=cds2930	3122258-3123481	1
mRNA	ID=cds3274	3464819-3465013	1
mRNA	ID=cds364	392194-393642	1
mRNA	ID=cds3907	4187809-4188348	1
mRNA	ID=cds4049	4352977-4354434	1
mRNA	ID=cds3790	4042222-4043652	1
mRNA	ID=cds4209	4516744-4517247	1
mRNA	ID=cds1263	1340682-1341008	1
mRNA	ID=cds1828	1920145-1920336	1
mRNA	ID=cds1838	1928058-1928414	1
mRNA	ID=cds184	213925-214125	1
mRNA	ID=cds1864	1952602-1955031	1
mRNA	ID=cds1990	2077557-2078615	1
mRNA	ID=cds2224	2350669-2352297	1
mRNA	ID=cds28	27293-28207	1
mRNA	ID=cds3069	3268647-3269792	1
mRNA	ID=cds336	359216-360370	1
mRNA	ID=cds3660	3890788-3891747	1
mRNA	ID=cds371	398249-398557	1
mRNA	ID=cds3839	4100845-4101519	1
mRNA	ID=cds3924	4202665-4203954	1
mRNA	ID=cds671	711261-712025	1
mRNA	ID=cds783	837413-837679	1
mRNA	ID=cds998	1081466-1082593	1
repeat_region		3884801-3884829	1
repeat_region		398204-398238	1
mRNA	ID=cds1441	1524271-1524888	1
mRNA	ID=cds1633	1719288-1720145	1
mRNA	ID=cds1842	1930817-1932628	1
mRNA	ID=cds2121	2224531-2225292	1
mRNA	ID=cds2153	2260387-2261517	1
mRNA	ID=cds2346	2473895-2474200	1
mRNA	ID=cds2351	2477224-2478552	1
mRNA	ID=cds2401	2536694-2537605	1
mRNA	ID=cds2458	2594927-2595640	1
mRNA	ID=cds2634	2794892-2795050	1
mRNA	ID=cds2677	2837546-2839003	1
mRNA	ID=cds2962	3154645-3155472	1
mRNA	ID=cds3010	3203346-3204278	1
mRNA	ID=cds3166	3367497-3368372	1
mRNA	ID=cds3182	3383560-3383823	1
mRNA	ID=cds3207	3410643-3410822	1
mRNA	ID=cds3292	3482240-3482458	1
mRNA	ID=cds3348	3541189-3542067	1
mRNA	ID=cds3370	3573094-3573687	1
mRNA	ID=cds3642	3871619-3872497	1
mRNA	ID=cds3848	4109638-4110237	1
mRNA	ID=cds471	505827-506306	1
mRNA	ID=cds921	1002112-1002654	1
mRNA	ID=cds2636	2795542-2796066	1
mRNA	ID=cds743	794312-795085	1
mRNA	ID=cds3012	3205393-3205998	1
mRNA	ID=cds1018	1103174-1103629	1
mRNA	ID=cds1052	1131797-1133005	1
mRNA	ID=cds1439	1521331-1522392	1
mRNA	ID=cds1608	1696176-1697204	1
mRNA	ID=cds1785	1877031-1877279	1
mRNA	ID=cds1840	1928905-1930083	1
mRNA	ID=cds1871	1960996-1963074	1
mRNA	ID=cds1913	2001896-2003302	1
mRNA	ID=cds2845	3031087-3031635	1
mRNA	ID=cds306	322982-323677	1
mRNA	ID=cds3511	3735520-3737550	1
mRNA	ID=cds3952	4245994-4247334	1
mRNA	ID=cds4185	4493213-4494232	1
mRNA	ID=cds4285	4603827-4604063	1
mRNA	ID=cds4292	4610151-4610312	1

mRNA	ID=cds534	570116-570667	1
mRNA	ID=cds586	620408-621412	1
mRNA	ID=cds963	1045072-1047168	1
mRNA	ID=cds2371	2503569-2504654	1
mRNA	ID=cds3011	3204485-3205396	1
mRNA	ID=cds750	801110-802543	1
mRNA	ID=cds1141	1215012-1215248	1
mRNA	ID=cds1445	1528610-1529347	1
mRNA	ID=cds1464	1550852-1551862	1
mRNA	ID=cds1559	1646532-1646687	1
mRNA	ID=cds1569	1653371-1653697	1
mRNA	ID=cds1865	1955056-1956156	1
mRNA	ID=cds2031	2118184-2119578	1
mRNA	ID=cds2405	2540534-2541550	1
mRNA	ID=cds2605	2763535-2763798	1
mRNA	ID=cds2608	2765732-2766595	1
mRNA	ID=cds2627	2787007-2787984	1
mRNA	ID=cds2732	2893798-2894577	1
mRNA	ID=cds3062	3260474-3261682	1
mRNA	ID=cds3280	3472700-3472987	1
mRNA	ID=cds329	350439-351890	1
mRNA	ID=cds3639	3868461-3869753	1
mRNA	ID=cds3981	4276502-4277851	1
mRNA	ID=cds4206	4514787-4515740	1
mRNA	ID=cds4228	4534637-4535617	1
mRNA	ID=cds494	530519-531445	1
mRNA	ID=cds621	657448-658041	1
mRNA	ID=cds1875	1965476-1966525	1
mRNA	ID=cds1362	1433784-1434917	1
mRNA	ID=cds1113	1196090-1196755	1
mRNA	ID=cds1115	1197918-1198811	1
mRNA	ID=cds1128	1205366-1206145	1
mRNA	ID=cds1218	1293649-1294215	1
mRNA	ID=cds1264	1341134-1341352	1
mRNA	ID=cds1302	1379971-1380876	1
mRNA	ID=cds1470	1556055-1557041	1
mRNA	ID=cds1725	1817880-1819238	1
mRNA	ID=cds1760	1854005-1854952	1
mRNA	ID=cds2042	2130091-2130579	1
mRNA	ID=cds2044	2131514-2133676	1
mRNA	ID=cds240	264528-264767	1
mRNA	ID=cds250	272071-273178	1
mRNA	ID=cds2720	2879073-2880164	1
mRNA	ID=cds3053	3250933-3251289	1
mRNA	ID=cds3085	3283500-3284291	1
mRNA	ID=cds4104	4410410-4411048	1
mRNA	ID=cds4105	4411051-4412214	1
mRNA	ID=cds4274	4592960-4593874	1
mRNA	ID=cds449	478591-479142	1
repeat_region		2660331-2660596	0.9297
repeat_region		3096435-3096555	0.834694
repeat_region		4631114-4631253	0.7698
mgeRNA	gbkey=mobile_element	1648867-1649572	0.714285
mRNA	ID=cds1565	1648869-1649561	0.714285
repeat_region		2441811-2441890	0.696666
repeat_region		3648735-3648834	0.693213
repeat_region		844854-844952	0.674099
repeat_region		4243115-4243240	0.667832
mgeRNA	gbkey=mobile_element	1049001-1049768	0.666668
mgeRNA	gbkey=mobile_element	1976527-1977294	0.666668
mgeRNA	gbkey=mobile_element	19796-20563	0.666668
mgeRNA	gbkey=mobile_element	3581451-3582218	0.666668
mRNA	ID=cds143	162105-164534	0.666666
mRNA	ID=cds2457	2593896-2594759	0.666666
mRNA	ID=cds413	440325-440567	0.666666
repeat_region		4468404-4468491	0.66535
repeat_region		831512-831609	0.657238
repeat_region		2833089-2833170	0.595889
repeat_region		3201166-3201290	0.595808
mRNA	ID=cds968	1049250-1049753	0.500001
mRNA	ID=cds1885	1976542-1977045	0.500001

mRNA	ID=cds19	19811-20314	0.500001
mRNA	ID=cds3380	3581700-3582203	0.500001
mRNA	ID=cds3193	3396897-3398000	0.5
mRNA	ID=cds594	629117-631222	0.5
mRNA	ID=cds2051	2141290-2144607	0.5
mRNA	ID=cds166	192872-193429	0.5
mRNA	ID=cds2759	2927598-2928965	0.5
mRNA	ID=cds3333	3520893-3523445	0.5
mRNA	ID=cds3535	3760206-3764339	0.5
mRNA	ID=cds3418	3617215-3621450	0.5
mRNA	ID=cds4186	4494698-4495963	0.5
mRNA	ID=cds4309	4630783-4631109	0.5
mRNA	ID=cds38	40417-41931	0.5
mRNA	ID=cds3383	3583104-3584846	0.5
mRNA	ID=cds1531	1631646-1632236	0.5
mRNA	ID=cds3073	3273304-3274875	0.5
mRNA	ID=cds803	859397-861829	0.5
mRNA	ID=cds1358	1431108-1431698	0.5
repeat_region		542311-542407	0.5
mRNA	ID=cds1604	1690914-1692287	0.5
repeat_region		138699-138796	0.499468
repeat_region		3906410-3906473	0.424024
mRNA	ID=cds2331	2463323-2464255	0.41833
repeat_region		2671798-2671831	0.404989
repeat_region		740177-740282	0.403118
mRNA	ID=cds101	114522-115724	0.360041
repeat_region		4106594-4106691	0.353553
mRNA	ID=cds1210	1286310-1286399	0.333333
repeat_region		1821355-1821452	0.296056
repeat_region		2230758-2230791	0.294431
repeat_region		348829-349193	0.286516
repeat_region		2428951-2429034	0.271403
repeat_region		3839899-3839967	0.256495
mRNA	ID=cds3734	3976624-3977976	0.25
mRNA	ID=cds263	284619-286001	0.25
repeat_region		4604581-4604672	0.223004
repeat_region		3734177-3734347	0.223004
repeat_region		2595695-2595740	0.20935
repeat_region		3085934-3085959	0.207332
repeat_region		2566161-2566343	0.196009
repeat_region		248147-248334	0.188151
repeat_region		4092339-4092740	0.182128
repeat_region		2116490-2116675	0.170883
repeat_region		3267744-3267841	0.1685
mRNA	ID=cds967	1049056-1049331	0.166667
mRNA	ID=cds1886	1976964-1977239	0.166667
mRNA	ID=cds20	20233-20508	0.166667
mRNA	ID=cds3379	3581506-3581781	0.166667
mRNA	ID=cds4032	4331970-4333613	0.158968
repeat_region		769898-769996	0.156062
repeat_region		5565-5669	0.146394
repeat_region		2509374-2509461	0.116202
repeat_region		3982243-3982346	0.114819
repeat_region		1112640-1112737	0.107527
tRNA	pseudo=true	2474606-2474620	0.1066
repeat_region		247458-247541	0.104217
repeat_region		1067577-1067674	0.091615
repeat_region		1532907-1532981	0.079063
repeat_region		550577-550733	0.079063
repeat_region		2217647-2217680	0.073238
repeat_region		4448967-4449051	0.073238
repeat_region		3112400-3112567	0.073238
repeat_region		3279724-3279805	0.071629
repeat_region		66565-66812	0.069969
repeat_region		4101530-4101613	0.055917
repeat_region		4247344-4247468	0.055917
repeat_region		216027-216137	0.052492
repeat_region		3672404-3672496	0.047004
repeat_region		4315940-4316022	0.047004
repeat_region		4458438-4458521	0.047004
repeat_region		2652992-2653088	0.047004

repeat_region		3328482-3328579	0.047004
repeat_region		3596420-3596518	0.047004
repeat_region		4371220-4371318	0.047004
repeat_region		3590587-3590685	0.047004
repeat_region		2536557-2536654	0.047004
repeat_region		898919-899016	0.047004
repeat_region		1689449-1689545	0.047004
repeat_region		2792050-2792185	0.04596
repeat_region		39151-39232	0.041219
repeat_region		3998173-3998275	0.032306
repeat_region		4006420-4006453	0.032306
mRNA	ID=cds4160	4465648-4468344	0.030907
repeat_region		3703986-3704083	0.025102
repeat_region		4135812-4135909	0.025102
repeat_region		1841748-1841840	0.02406
mRNA	ID=cds3895	4170080-4171108	0.016434
mRNA	ID=cds2434	2567523-2568359	0.015848
repeat_region		111430-111629	0.013654
repeat_region		1942243-1942369	0.013654
repeat_region		173504-173588	0.013654
mRNA	ID=cds1660	1749101-1749748	0.012714
repeat_region		1145151-1145225	0.012075
repeat_region		3738987-3739084	0.012075
repeat_region		4227399-4227468	0.008913
repeat_region		2892830-2892896	0.008913
repeat_region		3706295-3706380	0.008913
mRNA	ID=cds2303	2432846-2433658	0.003361
repeat_region		3537768-3537801	0.003013
