# Supplementary material for: The extracellular RNA complement of Escherichia coli
Source: Microbiologyopen. 2015 Jan 21;4(2):252–66. doi: 10.1002/mbo3.235 (PMC4398507; doi:10.1002/mbo3.235)
Supplement: Supplementary file 8 — Table S7. Genomic features and corresponding read counts in low density fractions (LDFs). [file mbo30004-0252-sd8.pdf]

Supplementary table S7 Genomic features and corresponding read counts in low density fractions (LDFs)

RNA Biotype	Product Information	Genomic Coordinates	Weighted Read Counts
tRNA	product=tRNA-Trp	3944980-3945055	1207
rRNA	product=23S ribosomal RNA of rrnA operon	4035542-4038446	745.555396
rRNA	product=23S ribosomal RNA of rrnH operon	225759-228662	732.438644
rRNA	product=23S ribosomal RNA of rrnG operon	2724303-2727206	730.357972
rRNA	product=23S ribosomal RNA of rrnB operon	4166664-4169567	730.357972
rRNA	product=23S ribosomal RNA of rrnE operon	4208066-4210969	729.129285
rRNA	product=23S ribosomal RNA of rrnC operon	3941727-3944630	728.620543
rRNA	product=23S ribosomal RNA of rrnD operon	3421902-3424805	701.412548
tRNA	product=tRNA-Thr	4173411-4173486	591
rRNA	product=16S ribosomal RNA of rrnH operon	223771-225312	380.60308
rRNA	product=16S ribosomal RNA of rrnA operon	4033554-4035095	354.393045
rRNA	product=16S ribosomal RNA of rrnG operon	2727638-2729179	351.845992
rRNA	product=16S ribosomal RNA of rrnC operon	3939831-3941372	350.739052
rRNA	product=16S ribosomal RNA of rrnD operon	3425243-3426784	350.596119
rRNA	product=16S ribosomal RNA of rrnB operon	4164682-4166223	349.209345
rRNA	product=16S ribosomal RNA of rrnE operon	4206170-4207711	349.195288
uncharacterized			248.198891
other ncRNA		475672-475785	175
tRNA	product=tRNA-Pro	3980758-3980834	152
rRNA	product=5S ribosomal RNA of rrnD operon	3421445-3421564	143.425118
tRNA	product=tRNA-Pro	2284233-2284309	140
tRNA	product=tRNA-Thr	3421602-3421677	114.0765
tRNA	product=tRNA-His	3980532-3980608	102.907608
tmRNA		2753615-2753977	96
mRNA	ID=cds2749	2916067-2917407	94
mgeRNA	gbkey=misc_feature	262182-296489	93.743655
tRNA	product=tRNA-Ala	3424980-3425055	93.419914
tRNA	product=tRNA-Ala	4035283-4035358	93.419914
tRNA	product=tRNA-Ala	225500-225575	93.419914
tRNA	product=tRNA-Ile	4035164-4035240	92.333241
tRNA	product=tRNA-Ile	3425098-3425174	92.333241
tRNA	product=tRNA-Ile	225381-225457	92.333241
mRNA	ID=cds2	2801-3733	85
mRNA	ID=cds1	337-2799	85
tRNA	product=tRNA-Val	1744540-1744616	81.394659
tRNA	product=tRNA-Cys	1989938-1990011	80.904029
mRNA	ID=cds44	47246-47776	80
tRNA	product=tRNA-Sec	3834245-3834339	78
tRNA	product=tRNA-Met	3316235-3316311	75.498545
mRNA	ID=cds3320	3509461-3510366	71
rRNA	product=5S ribosomal RNA of rrnA operon	4038540-4038659	67.605598
mRNA	ID=cds2505	2653097-2654380	67
tRNA	product=tRNA-Glu	4207797-4207872	65.25
tRNA	product=tRNA-Glu	3941458-3941533	65.25
tRNA	product=tRNA-Glu	2727391-2727466	65.25
tRNA	product=tRNA-Glu	4166395-4166470	65.25
rRNA	product=5S ribosomal RNA of rrnB operon	4169660-4169779	61.603593
rRNA	product=5S ribosomal RNA of rrnE operon	4211063-4211182	61.603593
rRNA	product=5S ribosomal RNA of rrnD operon	3421690-3421809	57.18672
rRNA	product=5S ribosomal RNA of rrnC operon	3944723-3944842	57.18672
rRNA	product=5S ribosomal RNA of rrnG operon	2724091-2724210	57.18672
rRNA	product=5S ribosomal RNA of rrnH operon	228756-228875	57.18672
tRNA	product=tRNA-Arg	2816495-2816571	57.071428
tRNA	product=tRNA-Arg	2816220-2816296	57.071428
tRNA	product=tRNA-Arg	2816081-2816157	57.071428
tRNA	product=tRNA-Arg	2815806-2815882	57.071428
tRNA	product=tRNA-Leu	3320094-3320180	57
tRNA	product=tRNA-Thr	262095-262170	56.445171
tRNA	product=tRNA-Met	2945409-2945485	54.16714
tRNA	product=tRNA-Met	2945519-2945595	54.16714
tRNA	product=tRNA-Met	2945629-2945705	54.16714
tRNA	product=tRNA-Leu	4604223-4604309	51.541425
tRNA	product=tRNA-Ser	1096788-1096875	50.9
tRNA	product=tRNA-Ser	925107-925194	50.9
tRNA	product=tRNA-Val	1744459-1744535	50.735252

mRNA	ID=cds4052	4358419-4359957	50
mRNA	ID=cds628	663325-664413	47
mRNA	ID=cds996	1078528-1080036	47
mRNA	ID=cds4257	4567021-4567941	46
mRNA	ID=cds283	303077-303406	46
tRNA	product=tRNA-Arg	3980398-3980474	46
tRNA	pseudo=true	296430-296478	44.850514
mRNA	ID=cds1707	1798666-1800594	44
mRNA	ID=cds1925	2011253-2012911	43
mRNA	ID=cds2380	2513665-2515854	43
tRNA	product=tRNA-Met	696280-696356	41.325551
tRNA	product=tRNA-Thr	4173777-4173852	40.754776
mRNA	ID=cds790	845683-846342	38
tRNA	product=tRNA-Gly	2997006-2997079	37
mRNA	ID=cds275	294920-296320	36
tRNA	product=tRNA-Asn	2060284-2060359	33.75
tRNA	product=tRNA-Asn	2042573-2042648	33.75
tRNA	product=tRNA-Asn	2057875-2057950	33.75
tRNA	product=tRNA-Asn	2056051-2056126	33.75
mRNA	ID=cds70	79464-80864	33.3333
mRNA	ID=cds1257	1333855-1336530	33.3333
mRNA	ID=cds3812	4071762-4072658	33.3333
mRNA	ID=cds2031	2118184-2119578	33
tRNA	product=tRNA-Leu	4604102-4604188	31.819508
tRNA	product=tRNA-Leu	4604338-4604424	31.819508
tRNA	product=tRNA-Leu	3980629-3980715	31.819508
tRNA	product=tRNA-Ser	2816575-2816667	29
mRNA	ID=cds471	505827-506306	28
tRNA	product=tRNA-Gly	4390383-4390458	27.058203
tRNA	product=tRNA-Gly	4390495-4390570	27.058203
tRNA	product=tRNA-Gly	1990066-1990141	27.058203
tRNA	product=tRNA-Gly	4390606-4390681	27.058203
tRNA	product=tRNA-Phe	3108388-3108463	26.304989
mRNA	ID=cds1109	1191890-1192996	26
tRNA	product=tRNA-Leu	1989839-1989925	25
tRNA	product=tRNA-Gly	4173696-4173770	25
tRNA	product=tRNA-Phe	4360574-4360649	24.695011
mRNA	ID=cds3799	4054648-4056057	24
tRNA	product=tRNA-Ala	2516063-2516138	23.370002
tRNA	product=tRNA-Ala	2516178-2516253	23.370002
mRNA	ID=cds4267	4581272-4584784	23
mRNA	ID=cds3184	3384243-3386210	23
tRNA	product=tRNA-Leu	4494428-4494512	23
tRNA	product=tRNA-Ser	1030848-1030935	21
tRNA	product=tRNA-Pro	3706639-3706715	20
mRNA	ID=cds4031	4331305-4331973	19
mRNA	ID=cds3386	3586133-3587203	19
mRNA	ID=cds684	726282-727955	19
mRNA	ID=cds960	1041253-1043433	18
mRNA	ID=cds3574	3800062-3801081	18
mRNA	ID=cds2147	2253377-2254036	18
other ncRNA		3268238-3268614	17
mRNA	ID=cds1738	1831425-1832135	16
tRNA	product=tRNA-Met	695887-695963	15.544549
mRNA	ID=cds2961	3153377-3154540	15
mRNA	ID=cds2984	3176137-3177618	15
mRNA	ID=cds28	27293-28207	15
mRNA	ID=cds3055	3252341-3253042	15
mRNA	ID=cds3277	3469422-3471536	14.5
mRNA	ID=cds3807	4065263-4067299	14.254023
tRNA	product=tRNA-Val	779988-780063	13.221062
mRNA	ID=cds340	365652-366734	13
mRNA	ID=cds1297	1374058-1374846	13
mRNA	ID=cds3237	3440788-3442119	12
tRNA	product=tRNA-Tyr	4173495-4173579	12
tRNA	product=tRNA-Ser	2041492-2041581	12
tRNA	product=tRNA-Asp	3944895-3944971	11.818043
tRNA	product=tRNA-Asp	228928-229004	11.818043

tRNA	product=tRNA-Asp	236931-237007	11.363885
tRNA	product=tRNA-Val	2518953-2519028	11.301138
tRNA	product=tRNA-Val	2519195-2519270	11.159266
tRNA	product=tRNA-Val	2519073-2519148	11.159266
tRNA	product=tRNA-Val	780291-780366	11.159266
mRNA	ID=cds3209	3411886-3413043	11
mRNA	ID=cds939	1020953-1023106	11
tRNA	product=tRNA-Leu	696186-696270	11
mRNA	ID=cds3155	3351143-3352072	10
mRNA	ID=cds2567	2717975-2720635	10
mRNA	ID=cds2960	3152284-3153240	10
mRNA	ID=cds1376	1453188-1453934	10
mRNA	ID=cds163	189874-190599	10
mRNA	ID=cds264	286013-287623	9.953748
mgeRNA	gbkey=misc_feature	1409923-1432982	9.272727
mRNA	ID=cds3884	4151719-4152870	9
mRNA	ID=cds1942	2024347-2026041	9
mRNA	ID=cds2869	3055200-3056432	8
mRNA	ID=cds3232	3438062-3439051	8
mRNA	ID=cds4154	4457923-4458387	8
mRNA	ID=cds1495	1590689-1596011	8
mRNA	ID=cds3473	3694481-3696052	7.5
tRNA	product=tRNA-Arg	563946-564022	7.205344
mRNA	ID=cds2026	2111458-2112351	7
mRNA	ID=cds2072	2167717-2168191	7
mRNA	ID=cds298	315710-316393	7
mRNA	ID=cds4075	4378533-4380341	7
mRNA	ID=cds3464	3678467-3679963	7
mRNA	ID=cds3303	3492033-3494576	7
mRNA	ID=cds1619	1707166-1708224	7
mRNA	ID=cds168	194903-195664	7
mRNA	ID=cds903	975549-980009	7
mRNA	ID=cds3905	4179268-4183296	7
mRNA	ID=cds3064	3263061-3264050	7
other ncRNA		2940718-2940923	7
tRNA	product=tRNA-Lys	780066-780141	6.576881
mRNA	ID=cds594	629117-631222	6.046252
mRNA	ID=cds4140	4442135-4445914	6
mRNA	ID=cds2689	2849023-2849895	6
mRNA	ID=cds2013	2097886-2099292	6
mRNA	ID=cds60	68348-70048	6
mRNA	ID=cds951	1032477-1034270	6
mRNA	ID=cds178	205126-208608	6
mRNA	ID=cds3517	3742351-3742824	6
mRNA	ID=cds3450	3657255-3658412	6
mRNA	ID=cds779	832293-834443	6
mRNA	ID=cds3276	3468167-3469351	6
mRNA	ID=cds3898	4173967-4175151	6
mRNA	ID=cds890	961218-962891	6
mRNA	ID=cds2078	2172619-2173071	6
mRNA	ID=cds2659	2817403-2820033	6
mgeRNA	gbkey=misc_feature	2065378-2077055	5
mRNA	ID=cds3103	3299507-3300502	5
mRNA	ID=cds1479	1568669-1570069	5
mRNA	ID=cds1701	1793581-1795968	5
mRNA	ID=cds287	308582-309250	5
mRNA	ID=cds1675	1763653-1766709	5
mRNA	ID=cds852	910405-911373	5
mRNA	ID=cds871	937217-938560	5
mRNA	ID=cds3238	3442127-3442561	5
mRNA	ID=cds2580	2739382-2739747	5
mRNA	ID=cds2277	2407542-2409374	5
mRNA	ID=cds1460	1545425-1548472	5
mRNA	ID=cds3622	3851945-3853129	5
mRNA	ID=cds2643	2799370-2801514	5
mRNA	ID=cds4060	4367179-4368435	5
mRNA	ID=cds2981	3174028-3174855	5
mRNA	ID=cds2116	2217714-2220011	5

mRNA	ID=cds1504	1605370-1606128	5
mRNA	ID=cds2464	2599840-2601858	5
mRNA	ID=cds3673	3904876-3905601	5
mRNA	ID=cds3120	3320755-3322092	5
mRNA	ID=cds627	661975-663186	5
mRNA	ID=cds83	94650-96008	5
mRNA	ID=cds2076	2170945-2172300	5
mRNA	ID=cds3994	4292504-4293817	5
repeat_region		34727-34762	5
tRNA	product=tRNA-Lys	2519275-2519350	4.981149
tRNA	product=tRNA-Lys	779777-779852	4.981149
tRNA	product=tRNA-Lys	780370-780445	4.981149
tRNA	product=tRNA-Lys	780592-780667	4.981149
tRNA	product=tRNA-Lys	780800-780875	4.981149
mgeRNA	gbkey=misc_feature	564025-585326	4.967383
mRNA	ID=cds720	772265-773404	4.795875
mRNA	ID=cds2571	2729622-2732195	4.5
mRNA	ID=cds2781	2957082-2960450	4.014095
mRNA	ID=cds3902	4176902-4177606	4
mRNA	ID=cds339	362455-365529	4
mRNA	ID=cds3666	3896045-3896632	4
mRNA	ID=cds3063	3261708-3263039	4
mRNA	ID=cds2318	2448073-2448612	4
mRNA	ID=cds3242	3443629-3444162	4
mRNA	ID=cds2158	2265851-2266837	4
mRNA	ID=cds1664	1753722-1755134	4
mRNA	ID=cds286	306031-308556	4
mRNA	ID=cds3808	4067498-4068424	4
mRNA	ID=cds1350	1421806-1423263	4
mRNA	ID=cds3567	3792952-3793998	4
mRNA	ID=cds3725	3967054-3968100	4
mRNA	ID=cds7	8238-9191	4
mRNA	ID=cds1959	2041675-2042472	4
mRNA	ID=cds2852	3038438-3038845	4
mRNA	ID=cds3262	3454399-3456351	4
mRNA	ID=cds1206	1279087-1282830	4
mRNA	ID=cds718	767201-769834	4
mRNA	ID=cds3930	4215132-4216436	4
mRNA	ID=cds2269	2398240-2399577	4
mRNA	ID=cds2415	2550374-2551243	4
mRNA	ID=cds3061	3258146-3260440	4
mRNA	ID=cds911	989845-992457	4
mRNA	ID=cds2494	2635496-2636674	4
mRNA	ID=cds399	426871-428718	4
mRNA	ID=cds4097	4404213-4404638	4
mRNA	ID=cds4123	4424651-4425445	4
mRNA	ID=cds656	692754-694178	4
mRNA	ID=cds2498	2639853-2640866	4
mRNA	ID=cds619	656515-656724	4
mRNA	ID=cds3353	3546008-3548092	4
mRNA	ID=cds3951	4244807-4245922	4
mRNA	ID=cds973	1051512-1052585	4
mRNA	ID=cds1711	1804394-1805323	4
mRNA	ID=cds475	510865-511797	4
mRNA	ID=cds2853	3038826-3039092	4
mRNA	ID=cds3657	3886753-3888168	4
other ncRNA		3054871-3055010	4
tRNA	product=tRNA-Arg	2464331-2464405	4
mRNA	ID=cds1222	1300923-1301843	3.5
tRNA	product=tRNA-Tyr	1286467-1286551	3.5
tRNA	product=tRNA-Tyr	1286761-1286845	3.5
mRNA	ID=cds4098	4404677-4407118	3.333333
mRNA	ID=cds3890	4159147-4159794	3
mRNA	ID=cds947	1029287-1029565	3
mRNA	ID=cds3133	3333257-3334516	3
mRNA	ID=cds3507	3730224-3731765	3
mRNA	ID=cds3435	3643408-3644250	3
mRNA	ID=cds713	760745-761962	3

mRNA	ID=cds4058	4364914-4366350	3
mRNA	ID=cds1170	1239558-1241294	3
mRNA	ID=cds1478	1566978-1568513	3
mRNA	ID=cds1807	1900072-1901043	3
mRNA	ID=cds3428	3634231-3635433	3
mRNA	ID=cds3873	4135955-4137058	3
mRNA	ID=cds4203	4510434-4511432	3
mRNA	ID=cds128	147944-148795	3
mRNA	ID=cds3597	3820423-3822531	3
mRNA	ID=cds595	631405-631602	3
mRNA	ID=cds3296	3484813-3486915	3
mRNA	ID=cds518	556098-556964	3
mRNA	ID=cds157	182463-183620	3
mRNA	ID=cds4150	4453808-4455181	3
mRNA	ID=cds2365	2495079-2496317	3
mRNA	ID=cds752	805221-806504	3
mRNA	ID=cds3245	3444921-3445460	3
mRNA	ID=cds1169	1238102-1239172	3
mRNA	ID=cds2282	2412769-2414913	3
mRNA	ID=cds4234	4541751-4542290	3
mRNA	ID=cds2896	3084728-3085882	3
mRNA	ID=cds3146	3342739-3344172	3
mRNA	ID=cds3530	3752996-3754534	3
mRNA	ID=cds1715	1807404-1808072	3
mRNA	ID=cds866	928419-930185	3
mRNA	ID=cds3416	3615799-3616605	3
mRNA	ID=cds3742	3987848-3988789	3
mRNA	ID=cds4092	4398695-4399975	3
mRNA	ID=cds1700	1793277-1793576	3
mRNA	ID=cds1309	1387894-1388622	3
mRNA	ID=cds3231	3437638-3438021	3
mRNA	ID=cds3872	4135063-4135680	3
mRNA	ID=cds3984	4281276-4282925	3
mRNA	ID=cds388	411831-414977	3
mRNA	ID=cds3586	3810754-3811974	3
mRNA	ID=cds3431	3638134-3638568	3
mRNA	ID=cds2986	3178443-3179603	3
mRNA	ID=cds3247	3445800-3446171	3
mRNA	ID=cds3354	3548102-3550495	3
mRNA	ID=cds1665	1755445-1755681	3
mRNA	ID=cds4110	4415721-4416476	3
mRNA	ID=cds4240	4547976-4549319	3
mRNA	ID=cds3750	3996006-3998168	3
mRNA	ID=cds2538	2687693-2689120	3
mRNA	ID=cds1642	1727111-1731727	3
mRNA	ID=cds3679	3911853-3913223	3
mRNA	ID=cds1733	1824940-1826283	3
mRNA	ID=cds2566	2717245-2717943	3
mRNA	ID=cds2934	3127065-3128165	3
mRNA	ID=cds2707	2868277-2869326	3
mRNA	ID=cds2314	2444410-2445495	3
mRNA	ID=cds2586	2743392-2743940	3
mRNA	ID=cds817	877965-879080	3
mRNA	ID=cds952	1034289-1034996	3
mRNA	ID=cds2645	2802837-2804039	3
mRNA	ID=cds2507	2654770-2655105	3
mRNA	ID=cds171	197928-200360	3
mRNA	ID=cds1661	1749752-1751854	3
mRNA	ID=cds3327	3516565-3517086	3
mRNA	ID=cds2758	2926251-2927540	3
mRNA	ID=cds2100	2198301-2201933	3
mRNA	ID=cds1656	1745155-1746759	3
mRNA	ID=cds1611	1700257-1701258	3
mRNA	ID=cds2051	2141290-2144607	3
mRNA	ID=cds950	1031362-1032480	3
mRNA	ID=cds3203	3407092-3407973	3
mRNA	ID=cds405	432679-433782	3
mRNA	ID=cds3544	3769405-3769767	3

mRNA	ID=cds1980	2069563-2072682	3
mRNA	ID=cds3454	3664203-3665603	3
mRNA	ID=cds3233	3439077-3439697	3
mRNA	ID=cds3166	3367497-3368372	3
mRNA	ID=cds3338	3527796-3528674	3
mRNA	ID=cds1966	2057988-2058938	3
mRNA	ID=cds4089	4395435-4397282	3
mRNA	ID=cds2130	2232055-2233293	3
mRNA	ID=cds34	35377-36162	3
mRNA	ID=cds3336	3526691-3527359	3
mRNA	ID=cds4096	4402710-4404008	3
mRNA	ID=cds869	932447-936436	3
mRNA	ID=cds3774	4020759-4021535	3
mRNA	ID=cds814	874558-875886	3
mRNA	ID=cds2404	2539701-2540534	3
mRNA	ID=cds1774	1870065-1871555	3
mRNA	ID=cds3112	3309855-3310799	3
mRNA	ID=cds3773	4020241-4020756	3
mRNA	ID=cds113	131615-134212	3
other ncRNA		3054005-3054187	3
tRNA	product=tRNA-Gln	696088-696162	2.75
tRNA	product=tRNA-Gln	695979-696053	2.75
repeat_region		3545909-3545995	2.711009
mRNA	ID=cds3770	4017647-4018252	2.5
mRNA	ID=cds3614	3843799-3845190	2.5
tRNA	product=tRNA-Gln	695765-695839	2.25
tRNA	product=tRNA-Gln	695653-695727	2.25
mRNA	ID=cds949	1029982-1030641	2.204125
mRNA	ID=cds3355	3551107-3553812	2.010879
mgeRNA	gbkey=misc_feature	1630310-1650767	2
mgeRNA	gbkey=mobile_element	4505148-4505481	2
mgeRNA	gbkey=mobile_element	1501185-1502932	2
mRNA	ID=cds1843	1932863-1934338	2
mRNA	ID=cds4010	4312367-4313125	2
mRNA	ID=cds2262	2389534-2391063	2
mRNA	ID=cds3648	3878171-3879244	2
mRNA	ID=cds285	304398-306041	2
mRNA	ID=cds2691	2850158-2851279	2
mRNA	ID=cds2153	2260387-2261517	2
mRNA	ID=cds3241	3443266-3443619	2
mRNA	ID=cds119	138835-141225	2
mRNA	ID=cds3459	3671385-3672398	2
mRNA	ID=cds472	506510-507304	2
mRNA	ID=cds3863	4122635-4124833	2
mRNA	ID=cds3080	3279093-3279647	2
mRNA	ID=cds3875	4137743-4140244	2
mRNA	ID=cds2421	2555340-2556701	2
mRNA	ID=cds3040	3239849-3241336	2
mRNA	ID=cds3798	4053313-4054362	2
mRNA	ID=cds2840	3025143-3026510	2
mRNA	ID=cds2117	2220207-2221922	2
mRNA	ID=cds3761	4007193-4008215	2
mRNA	ID=cds3556	3781684-3782151	2
mRNA	ID=cds3467	3683723-3687196	2
mRNA	ID=cds3526	3749151-3749891	2
mRNA	ID=cds2778	2948657-2950483	2
mRNA	ID=cds2893	3081957-3083933	2
mRNA	ID=cds4301	4619792-4621123	2
mRNA	ID=cds2709	2869802-2870512	2
mRNA	ID=cds667	709423-709869	2
mRNA	ID=cds1612	1701292-1702332	2
mRNA	ID=cds3352	3544581-3545897	2
mRNA	ID=cds792	847631-848134	2
mRNA	ID=cds3052	3250326-3250691	2
mRNA	ID=cds2205	2321469-2322131	2
mRNA	ID=cds1788	1878145-1878783	2
mRNA	ID=cds3456	3667615-3669264	2
mRNA	ID=cds1029	1113487-1114713	2

mRNA	ID=cds4074	4377806-4378540	2
mRNA	ID=cds394	421742-423556	2
mRNA	ID=cds2950	3143165-3144283	2
mRNA	ID=cds2811	2989290-2989781	2
mRNA	ID=cds1494	1590200-1590466	2
mRNA	ID=cds2290	2420671-2421561	2
mRNA	ID=cds1354	1425413-1425622	2
mRNA	ID=cds2225	2352287-2353546	2
mRNA	ID=cds21	20815-21078	2
mRNA	ID=cds211	240859-243303	2
mRNA	ID=cds3157	3357220-3358638	2
mRNA	ID=cds110	125695-127587	2
mRNA	ID=cds3154	3348711-3351047	2
mRNA	ID=cds1385	1461563-1462513	2
mRNA	ID=cds3258	3450981-3451292	2
mRNA	ID=cds2064	2160900-2162303	2
mRNA	ID=cds1996	2083728-2085086	2
mRNA	ID=cds1491	1586877-1588103	2
mRNA	ID=cds3288	3476824-3478629	2
mRNA	ID=cds678	717485-719683	2
mRNA	ID=cds3210	3413055-3416159	2
mRNA	ID=cds2862	3049137-3050339	2
mRNA	ID=cds3138	3336488-3337270	2
mRNA	ID=cds3256	3449703-3450308	2
mRNA	ID=cds4263	4575981-4577360	2
mRNA	ID=cds3179	3380222-3381289	2
mRNA	ID=cds3964	4258622-4259329	2
mRNA	ID=cds4235	4542327-4543052	2
mRNA	ID=cds2593	2749817-2751478	2
mRNA	ID=cds3405	3604474-3606672	2
mRNA	ID=cds668	709862-709948	2
mRNA	ID=cds4104	4410410-4411048	2
mRNA	ID=cds3510	3734376-3735200	2
mRNA	ID=cds3214	3419347-3420450	2
mRNA	ID=cds422	447874-449865	2
mRNA	ID=cds2496	2637323-2638597	2
mRNA	ID=cds3626	3854438-3854887	2
mRNA	ID=cds3409	3608539-3609756	2
mRNA	ID=cds187	215269-215979	2
mRNA	ID=cds657	694324-695499	2
mRNA	ID=cds1590	1672996-1674384	2
mRNA	ID=cds3252	3447923-3448255	2
mRNA	ID=cds227	254259-255716	2
mRNA	ID=cds936	1018236-1019276	2
mRNA	ID=cds4048	4351223-4352740	2
mRNA	ID=cds3397	3597952-3598806	2
mRNA	ID=cds1791	1881212-1882657	2
mRNA	ID=cds3326	3515420-3516508	2
mRNA	ID=cds278	296994-297950	2
mRNA	ID=cds2928	3119656-3121827	2
mRNA	ID=cds30	29651-30799	2
mRNA	ID=cds1586	1669984-1670805	2
mRNA	ID=cds2833	3013182-3013760	2
mRNA	ID=cds2996	3188654-3189718	2
mRNA	ID=cds1778	1872779-1873600	2
mRNA	ID=cds1692	1786459-1787505	2
mRNA	ID=cds719	770681-772249	2
mRNA	ID=cds486	519640-522054	2
mRNA	ID=cds4158	4464322-4465269	2
mRNA	ID=cds308	324801-326471	2
mRNA	ID=cds3652	3882516-3882875	2
mRNA	ID=cds3929	4213501-4215102	2
mRNA	ID=cds4138	4439561-4440199	2
mRNA	ID=cds4197	4505220-4505474	2
mRNA	ID=cds1984	2075136-2075504	2
mRNA	ID=cds3906	4183373-4187596	2
mRNA	ID=cds2217	2342887-2345172	2
mRNA	ID=cds3246	3445475-3445789	2

mRNA	ID=cds2174	2284412-2286936	2
mRNA	ID=cds4186	4494698-4495963	2
mRNA	ID=cds1164	1231723-1232253	2
mRNA	ID=cds3627	3854934-3856427	2
mRNA	ID=cds1180	1250289-1252208	2
mRNA	ID=cds3368	3569339-3571525	2
mRNA	ID=cds2227	2354926-2355825	2
mRNA	ID=cds12	12163-14079	2
mRNA	ID=cds2079	2173081-2174343	2
mRNA	ID=cds3180	3381352-3382290	2
mRNA	ID=cds664	705316-706980	2
mRNA	ID=cds3057	3253363-3254673	2
mRNA	ID=cds4081	4387415-4388383	2
mRNA	ID=cds1379	1455521-1456288	2
mRNA	ID=cds2462	2598500-2598970	2
mRNA	ID=cds4065	4372652-4373680	2
mRNA	ID=cds3447	3654431-3654763	2
mRNA	ID=cds2080	2174372-2175226	2
mRNA	ID=cds1165	1232399-1233940	2
mRNA	ID=cds1826	1918247-1919686	2
mRNA	ID=cds3693	3925178-3926170	2
mRNA	ID=cds4169	4473460-4475274	2
mRNA	ID=cds3279	3472200-3472574	2
mRNA	ID=cds2199	2309668-2310771	2
mRNA	ID=cds1462	1549362-1550015	2
mRNA	ID=cds2918	3103736-3104992	2
mRNA	ID=cds443	473525-474385	2
mRNA	ID=cds1279	1355826-1357211	2
mRNA	ID=cds957	1038519-1039655	2
mRNA	ID=cds1918	2006301-2007506	2
mRNA	ID=cds1399	1481085-1484987	2
mRNA	ID=cds2189	2298289-2300775	2
mRNA	ID=cds2190	2300772-2301035	2
mRNA	ID=cds1064	1146017-1146538	2
mRNA	ID=cds3805	4062386-4063789	2
mRNA	ID=cds1633	1719288-1720145	2
mRNA	ID=cds3089	3286836-3289352	2
mRNA	ID=cds1388	1468541-1472037	2
mRNA	ID=cds2456	2591866-2593881	2
mRNA	ID=cds901	973542-974864	2
mRNA	ID=cds4095	4402409-4402606	2
mRNA	ID=cds2584	2742205-2742552	2
mRNA	ID=cds3248	3446336-3446590	2
mRNA	ID=cds116	135598-136464	2
mRNA	ID=cds3072	3271595-3272929	2
mRNA	ID=cds1299	1375908-1378175	2
mRNA	ID=cds4035	4336277-4338544	2
mRNA	ID=cds2131	2233287-2234522	2
mRNA	ID=cds3243	3444175-3444567	2
mRNA	ID=cds1644	1732459-1733274	2
mRNA	ID=cds3171	3372891-3374258	2
mRNA	ID=cds3649	3879244-3880344	2
mRNA	ID=cds4012	4313548-4314105	2
mRNA	ID=cds816	877471-877854	2
mRNA	ID=cds3335	3524491-3526626	2
mRNA	ID=cds161	185978-188650	2
mRNA	ID=cds4181	4489229-4490548	2
mRNA	ID=cds210	240343-240816	2
mRNA	ID=cds3286	3475929-3476519	2
mRNA	ID=cds703	747144-748205	2
mRNA	ID=cds870	936595-937206	2
mRNA	ID=cds2248	2374856-2375614	2
mRNA	ID=cds1254	1331879-1332853	2
mRNA	ID=cds4041	4345427-4346767	2
mRNA	ID=cds2867	3054263-3054811	2
mRNA	ID=cds584	618607-619422	2
mRNA	ID=cds4316	4636201-4637553	2
mRNA	ID=cds2499	2641151-2642305	2

mRNA	ID=cds4299	4617626-4618849	2
mRNA	ID=cds635	669797-670828	2
mRNA	ID=cds1363	1435284-1438808	2
mRNA	ID=cds605	641311-642549	2
mRNA	ID=cds882	950495-952777	2
mRNA	ID=cds523	562553-563068	2
mRNA	ID=cds3161	3363207-3363573	2
mRNA	ID=cds987	1067734-1069062	2
mRNA	ID=cds3901	4176470-4176898	2
mRNA	ID=cds4057	4363495-4364796	2
mRNA	ID=cds1514	1616267-1616932	2
mRNA	ID=cds857	917351-918343	2
mRNA	ID=cds835	894214-895347	2
mRNA	ID=cds1418	1501741-1502889	2
mRNA	ID=cds3683	3916339-3917880	2
mRNA	ID=cds51	53416-54702	2
mRNA	ID=cds1610	1698981-1700153	2
other ncRNA		2732175-2732317	2
repeat_region		3982243-3982346	2
repeat_region		4407176-4407273	2
tRNA	pseudo=true	585280-585324	1.794656
mRNA	ID=cds3792	4044989-4047775	1.646447
mRNA	ID=cds331	354146-355405	1.5
mRNA	ID=cds2524	2670069-2671271	1.5
mRNA	ID=cds109	123017-125680	1.5
mRNA	ID=cds3373	3575754-3576749	1.5
mRNA	ID=cds534	570116-570667	1.5
mRNA	ID=cds106	119281-120135	1.5
mRNA	ID=cds111	127912-129336	1.333333
repeat_region		489183-489280	1.308599
mRNA	ID=cds2303	2432846-2433658	1.03969
mRNA	ID=cds3520	3745107-3746603	1.010312
mgeRNA	gbkey=misc_feature	1195443-1210635	1
mgeRNA	gbkey=misc_feature	2556721-2563483	1
mRNA	ID=cds1384	1460149-1461462	1
mRNA	ID=cds2469	2606509-2608176	1
mRNA	ID=cds3867	4126695-4127855	1
mRNA	ID=cds84	96002-97084	1
mRNA	ID=cds90	103155-103985	1
mRNA	ID=cds3703	3937208-3938635	1
mRNA	ID=cds3647	3875728-3878142	1
mRNA	ID=cds2651	2808792-2809322	1
mRNA	ID=cds2416	2551247-2552146	1
mRNA	ID=cds1069	1149893-1150627	1
mRNA	ID=cds768	820765-821721	1
mRNA	ID=cds460	491316-493247	1
mRNA	ID=cds2484	2623137-2624678	1
mRNA	ID=cds1947	2028923-2030341	1
mRNA	ID=cds2493	2633906-2635378	1
mRNA	ID=cds2958	3150258-3151445	1
mRNA	ID=cds2200	2311510-2314182	1
mRNA	ID=cds750	801110-802543	1
mRNA	ID=cds2246	2373022-2373984	1
mRNA	ID=cds954	1035577-1035975	1
mRNA	ID=cds1574	1656093-1658519	1
mRNA	ID=cds3172	3374301-3374798	1
mRNA	ID=cds1694	1787832-1789268	1
mRNA	ID=cds347	373092-374105	1
mRNA	ID=cds3525	3748941-3749132	1
mRNA	ID=cds2685	2845437-2847263	1
mRNA	ID=cds2587	2743959-2744207	1
mRNA	ID=cds2242	2370579-2370914	1
mRNA	ID=cds3834	4095759-4096595	1
mRNA	ID=cds1067	1147982-1148935	1
mRNA	ID=cds3787	4040092-4040361	1
mRNA	ID=cds3022	3217516-3218895	1
mRNA	ID=cds1243	1319408-1320970	1
mRNA	ID=cds3550	3775422-3777077	1

mRNA	ID=cds2426	2560133-2560549	1
mRNA	ID=cds491	528724-528816	1
mRNA	ID=cds2539	2689678-2693565	1
mRNA	ID=cds3227	3436046-3436456	1
mRNA	ID=cds1207	1282827-1284365	1
mRNA	ID=cds2476	2614116-2615579	1
mRNA	ID=cds3145	3341966-3342691	1
mRNA	ID=cds430	458112-460466	1
mRNA	ID=cds785	838472-840754	1
mRNA	ID=cds5	5683-6459	1
mRNA	ID=cds1766	1860040-1860453	1
mRNA	ID=cds2900	3089156-3089887	1
mRNA	ID=cds3366	3566056-3567351	1
mRNA	ID=cds2377	2509490-2510728	1
mRNA	ID=cds144	164730-167264	1
mRNA	ID=cds1090	1169741-1173187	1
mRNA	ID=cds2443	2574120-2576399	1
mRNA	ID=cds3025	3220655-3223747	1
mRNA	ID=cds615	651458-653116	1
mRNA	ID=cds3110	3307055-3309190	1
mRNA	ID=cds2548	2699763-2700491	1
mRNA	ID=cds2698	2859452-2860360	1
mRNA	ID=cds2965	3156949-3159168	1
mRNA	ID=cds716	764376-765098	1
mRNA	ID=cds2398	2534408-2535259	1
mRNA	ID=cds3716	3955993-3957468	1
mRNA	ID=cds2457	2593896-2594759	1
mRNA	ID=cds3016	3209129-3210874	1
mRNA	ID=cds1836	1924803-1926863	1
mRNA	ID=cds3427	3632864-3633916	1
mRNA	ID=cds4189	4498557-4498814	1
mRNA	ID=cds1330	1410024-1411259	1
mRNA	ID=cds3200	3403939-3405288	1
mRNA	ID=cds2863	3050362-3051540	1
mRNA	ID=cds3858	4117446-4118372	1
mRNA	ID=cds425	452813-453391	1
mRNA	ID=cds1216	1291732-1292145	1
mRNA	ID=cds3883	4148470-4151121	1
mRNA	ID=cds65	74497-75480	1
mRNA	ID=cds1856	1945435-1946175	1
mRNA	ID=cds1669	1758544-1759815	1
mRNA	ID=cds1471	1557038-1557934	1
mRNA	ID=cds777	830095-831459	1
mRNA	ID=cds712	757929-760730	1
mRNA	ID=cds776	829195-829866	1
mRNA	ID=cds4152	4455982-4457334	1
mRNA	ID=cds3669	3898627-3900243	1
mRNA	ID=cds2263	2391227-2393068	1
mRNA	ID=cds4284	4602898-4603686	1
mRNA	ID=cds3549	3774688-3775050	1
mRNA	ID=cds4157	4462782-4464203	1
mRNA	ID=cds2271	2400077-2401867	1
mRNA	ID=cds3560	3784861-3786120	1
mRNA	ID=cds2249	2375611-2377281	1
mRNA	ID=cds3842	4103843-4104343	1
mRNA	ID=cds680	720279-720956	1
mRNA	ID=cds3254	3448565-3449386	1
mRNA	ID=cds990	1070188-1070988	1
mRNA	ID=cds262	282425-284392	1
mRNA	ID=cds2682	2842232-2842774	1
mRNA	ID=cds3658	3888259-3889506	1
mRNA	ID=cds3715	3954950-3955843	1
mRNA	ID=cds632	667942-668259	1
mRNA	ID=cds683	724211-726259	1
mRNA	ID=cds1179	1248991-1250061	1
mRNA	ID=cds1714	1806721-1807257	1
mRNA	ID=cds1646	1734145-1735314	1
mRNA	ID=cds3371	3573744-3575084	1

mRNA	ID=cds2897	3086306-3087700	1
mRNA	ID=cds4132	4432645-4434588	1
mRNA	ID=cds2028	2113931-2115151	1
mRNA	ID=cds2631	2792275-2793675	1
mRNA	ID=cds3934	4221851-4225534	1
mRNA	ID=cds89	102233-103153	1
mRNA	ID=cds4231	4538980-4539582	1
mRNA	ID=cds3136	3335278-3335913	1
mRNA	ID=cds2356	2486045-2487190	1
mRNA	ID=cds1291	1367713-1368027	1
mRNA	ID=cds3475	3696237-3697916	1
mRNA	ID=cds1666	1755745-1756749	1
mRNA	ID=cds3041	3241351-3242763	1
mRNA	ID=cds3195	3399414-3401354	1
mRNA	ID=cds3868	4127858-4130290	1
mRNA	ID=cds3290	3479311-3481224	1
mRNA	ID=cds329	350439-351890	1
mRNA	ID=cds1366	1439878-1440867	1
mRNA	ID=cds3734	3976624-3977976	1
mRNA	ID=cds4093	4400061-4401320	1
mRNA	ID=cds3043	3244674-3245450	1
mRNA	ID=cds3271	3463104-3463565	1
mRNA	ID=cds3701	3935317-3936246	1
mRNA	ID=cds2202	2315049-2317898	1
mRNA	ID=cds1072	1152523-1153332	1
mRNA	ID=cds444	474603-475175	1
mRNA	ID=cds2038	2126364-2126912	1
mRNA	ID=cds677	716169-717488	1
mRNA	ID=cds3062	3260474-3261682	1
mRNA	ID=cds4086	4392089-4393636	1
mRNA	ID=cds3484	3708822-3710030	1
mRNA	ID=cds2187	2296737-2297600	1
mRNA	ID=cds1457	1542782-1543771	1
mRNA	ID=cds1019	1103670-1104125	1
mRNA	ID=cds3471	3693256-3694008	1
mRNA	ID=cds3582	3808366-3809175	1
mRNA	ID=cds2173	2282398-2284158	1
mRNA	ID=cds1580	1664548-1665243	1
mRNA	ID=cds3297	3486982-3488202	1
mRNA	ID=cds3600	3825483-3826688	1
mRNA	ID=cds513	552441-553163	1
mRNA	ID=cds899	971845-972624	1
mRNA	ID=cds3913	4192227-4194122	1
mRNA	ID=cds2826	3004284-3005474	1
mRNA	ID=cds1671	1760546-1762033	1
mRNA	ID=cds928	1009187-1011094	1
mRNA	ID=cds2988	3180572-3181345	1
mRNA	ID=cds2594	2751627-2751968	1
mRNA	ID=cds2834	3014082-3017180	1
mRNA	ID=cds3537	3765244-3766188	1
mRNA	ID=cds3610	3838572-3839762	1
mRNA	ID=cds1524	1623359-1625404	1
mRNA	ID=cds3839	4100845-4101519	1
mRNA	ID=cds2949	3142176-3143162	1
mRNA	ID=cds3278	3471564-3472103	1
mRNA	ID=cds1686	1778425-1779363	1
mRNA	ID=cds3291	3481224-3482246	1
mRNA	ID=cds3272	3463565-3464242	1
mRNA	ID=cds4178	4485341-4486423	1
mRNA	ID=cds1416	1500481-1501149	1
mRNA	ID=cds3225	3433229-3434518	1
mRNA	ID=cds428	455901-456524	1
mRNA	ID=cds2864	3051537-3052862	1
mRNA	ID=cds2798	2977043-2977978	1
mRNA	ID=cds1840	1928905-1930083	1
mRNA	ID=cds43	45807-47138	1
mRNA	ID=cds1327	1406074-1407057	1
mRNA	ID=cds1767	1860795-1861790	1

mRNA	ID=cds2541	2695376-2695879	1
mRNA	ID=cds1706	1798120-1798662	1
mRNA	ID=cds1070	1150838-1151074	1
mRNA	ID=cds1916	2004180-2005667	1
mRNA	ID=cds186	214833-215255	1
mRNA	ID=cds3548	3774194-3774403	1
mRNA	ID=cds2411	2547668-2548567	1
mRNA	ID=cds3767	4014454-4015215	1
mRNA	ID=cds2478	2616097-2616798	1
mRNA	ID=cds2146	2252267-2253208	1
mRNA	ID=cds602	638168-638731	1
mRNA	ID=cds306	322982-323677	1
mRNA	ID=cds1111	1193521-1194174	1
mRNA	ID=cds740	791539-793011	1
mRNA	ID=cds2975	3168506-3169855	1
mRNA	ID=cds2854	3039335-3040315	1
mRNA	ID=cds3601	3826968-3828359	1
mRNA	ID=cds1468	1554649-1555080	1
mRNA	ID=cds977	1057307-1058479	1
mRNA	ID=cds1702	1795983-1796966	1
mRNA	ID=cds2004	2089121-2090425	1
mRNA	ID=cds3498	3720351-3722420	1
mRNA	ID=cds2953	3145919-3146959	1
mRNA	ID=cds4274	4592960-4593874	1
mRNA	ID=cds4304	4622918-4623886	1
mRNA	ID=cds883	952832-953689	1
mRNA	ID=cds3790	4042222-4043652	1
mRNA	ID=cds3811	4070698-4071594	1
mRNA	ID=cds2959	3151585-3152244	1
mRNA	ID=cds3173	3374804-3375442	1
mRNA	ID=cds3684	3917893-3918426	1
mRNA	ID=cds407	434361-434780	1
mRNA	ID=cds2667	2825759-2826538	1
mRNA	ID=cds1819	1908300-1909673	1
mRNA	ID=cds2264	2393065-2393367	1
mRNA	ID=cds313	332725-333657	1
mRNA	ID=cds2880	3066969-3067829	1
mRNA	ID=cds200	232597-233955	1
mRNA	ID=cds4122	4424131-4424580	1
mRNA	ID=cds3168	3369106-3370596	1
mRNA	ID=cds2482	2620256-2620894	1
mRNA	ID=cds2245	2371670-2373025	1
mRNA	ID=cds3781	4029184-4030515	1
mRNA	ID=cds828	889312-889689	1
mRNA	ID=cds79	90094-91035	1
mRNA	ID=cds3850	4110990-4111289	1
mRNA	ID=cds2788	2964210-2966456	1
mRNA	ID=cds2703	2864581-2865573	1
mRNA	ID=cds3826	4086130-4087878	1
mRNA	ID=cds3565	3790849-3791706	1
mRNA	ID=cds2224	2350669-2352297	1
mRNA	ID=cds1653	1741481-1742854	1
mRNA	ID=cds763	817793-818278	1
mRNA	ID=cds2133	2235791-2237311	1
mRNA	ID=cds3640	3869873-3871021	1
mRNA	ID=cds3576	3802204-3803139	1
mRNA	ID=cds1667	1756898-1757314	1
mRNA	ID=cds1571	1654208-1654768	1
mRNA	ID=cds3820	4078322-4079251	1
mRNA	ID=cds3687	3919259-3920074	1
mRNA	ID=cds1897	1986740-1987237	1
mRNA	ID=cds1891	1981579-1983093	1
mRNA	ID=cds2485	2624717-2626960	1
mRNA	ID=cds1226	1304845-1305174	1
mRNA	ID=cds637	671424-674006	1
mRNA	ID=cds3924	4202665-4203954	1
mRNA	ID=cds4161	4468550-4468936	1
mRNA	ID=cds630	665539-667440	1

mRNA	ID=cds3367	3567369-3569342	1
mRNA	ID=cds219	248358-250070	1
mRNA	ID=cds4145	4447985-4448941	1
mRNA	ID=cds981	1062078-1062998	1
mRNA	ID=cds2531	2677486-2680767	1
mRNA	ID=cds107	120178-121551	1
mRNA	ID=cds3124	3325812-3326105	1
mRNA	ID=cds3127	3328604-3329776	1
mRNA	ID=cds3957	4252066-4254489	1
mRNA	ID=cds1751	1843023-1844984	1
mRNA	ID=cds2972	3166771-3167253	1
mRNA	ID=cds963	1045072-1047168	1
mRNA	ID=cds2488	2628348-2628887	1
mRNA	ID=cds452	480478-483627	1
mRNA	ID=cds3533	3757881-3759272	1
mRNA	ID=cds408	434858-435835	1
mRNA	ID=cds1045	1127062-1128597	1
mRNA	ID=cds1493	1588878-1590200	1
mRNA	ID=cds1435	1516352-1516870	1
mRNA	ID=cds2144	2249722-2250810	1
mRNA	ID=cds2546	2698640-2699020	1
mRNA	ID=cds1945	2027563-2028483	1
mRNA	ID=cds270	290628-291455	1
mRNA	ID=cds760	814962-815870	1
mRNA	ID=cds2021	2106361-2107608	1
mRNA	ID=cds2486	2627312-2627503	1
mRNA	ID=cds92	105305-106456	1
mRNA	ID=cds4067	4374340-4374465	1
mRNA	ID=cds77	88028-89032	1
mRNA	ID=cds1231	1309113-1309832	1
mRNA	ID=cds2093	2186452-2188932	1
mRNA	ID=cds3185	3386216-3387148	1
mRNA	ID=cds3226	3434540-3435916	1
mRNA	ID=cds2272	2401973-2402635	1
mRNA	ID=cds3429	3635665-3637164	1
mRNA	ID=cds572	603994-604647	1
mRNA	ID=cds2890	3077666-3079657	1
mRNA	ID=cds1913	2001896-2003302	1
mRNA	ID=cds1640	1725861-1726268	1
mRNA	ID=cds3071	3270809-3271579	1
mRNA	ID=cds2564	2715513-2716550	1
mRNA	ID=cds1506	1607253-1608704	1
mRNA	ID=cds2022	2107605-2108162	1
mRNA	ID=cds2962	3154645-3155472	1
mRNA	ID=cds784	837753-838430	1
mRNA	ID=cds1454	1536874-1540614	1
mRNA	ID=cds2513	2659665-2660153	1
mRNA	ID=cds3909	4189888-4190658	1
mRNA	ID=cds4175	4481860-4482303	1
mRNA	ID=cds3794	4049370-4049879	1
mRNA	ID=cds1405	1489946-1490095	1
mRNA	ID=cds4232	4540060-4540656	1
mRNA	ID=cds3857	4116868-4117353	1
mRNA	ID=cds181	211877-212266	1
mRNA	ID=cds88	100765-102240	1
mRNA	ID=cds3853	4112592-4113602	1
mRNA	ID=cds4120	4423543-4423857	1
mRNA	ID=cds2289	2419730-2420623	1
mRNA	ID=cds3743	3989176-3991722	1
mRNA	ID=cds821	882015-882611	1
mRNA	ID=cds2989	3181835-3182488	1
mRNA	ID=cds3775	4021577-4022359	1
mRNA	ID=cds3257	3450319-3450948	1
mRNA	ID=cds4021	4320828-4321253	1
mRNA	ID=cds972	1051290-1051463	1
mRNA	ID=cds1745	1837491-1838798	1
mRNA	ID=cds364	392194-393642	1
mRNA	ID=cds3815	4074169-4075041	1

mRNA	ID=cds3654	3883099-3884745	1
mRNA	ID=cds2779	2950483-2954025	1
mRNA	ID=cds2061	2153287-2156409	1
mRNA	ID=cds2508	2655107-2656957	1
mRNA	ID=cds1498	1599514-1601049	1
mRNA	ID=cds3983	4279806-4281098	1
mRNA	ID=cds4064	4371388-4372257	1
mRNA	ID=cds3374	3576973-3577668	1
mRNA	ID=cds3004	3197686-3198987	1
mRNA	ID=cds369	395863-397083	1
mRNA	ID=cds3194	3398066-3399109	1
mRNA	ID=cds849	906075-907505	1
mRNA	ID=cds3682	3915425-3916288	1
mRNA	ID=cds2247	2373984-2374841	1
mRNA	ID=cds3925	4203966-4205555	1
mRNA	ID=cds437	466636-467454	1
mRNA	ID=cds2870	3056688-3057347	1
mRNA	ID=cds2152	2259449-2260387	1
mRNA	ID=cds173	200971-201996	1
mRNA	ID=cds1147	1218824-1221471	1
mRNA	ID=cds3030	3229687-3231705	1
mRNA	ID=cds2017	2102518-2103108	1
mRNA	ID=cds295	313581-314452	1
mRNA	ID=cds1529	1629026-1630309	1
mRNA	ID=cds3273	3464271-3464747	1
mRNA	ID=cds440	469860-471641	1
mRNA	ID=cds2881	3068187-3069266	1
mRNA	ID=cds2954	3146999-3147493	1
mRNA	ID=cds4174	4479005-4481860	1
mRNA	ID=cds4205	4512376-4514700	1
mRNA	ID=cds2574	2734168-2734905	1
mRNA	ID=cds24	22391-25207	1
mRNA	ID=cds3156	3352747-3357207	1
mRNA	ID=cds2160	2268001-2268567	1
mRNA	ID=cds2874	3061009-3062004	1
mRNA	ID=cds3424	3627558-3628625	1
mRNA	ID=cds1672	1762042-1762410	1
mRNA	ID=cds1294	1371246-1372127	1
mRNA	ID=cds3892	4160193-4161293	1
mRNA	ID=cds4080	4384070-4387393	1
mRNA	ID=cds773	825342-826475	1
mRNA	ID=cds3433	3640403-3641155	1
mRNA	ID=cds3295	3484142-3484774	1
mRNA	ID=cds537	571689-572144	1
mRNA	ID=cds4308	4628756-4630693	1
other ncRNA		1768396-1768501	1
other ncRNA		2974124-2974211	1
other ncRNA		3656009-3656077	1
repeat_region		421596-421628	1
repeat_region		4323825-4324407	1
repeat_region		1555087-1555105	1
repeat_region		2438365-2438400	1
repeat_region		4626718-4626867	1
mRNA	ID=cds3535	3760206-3764339	0.5
mRNA	ID=cds3961	4257260-4257469	0.5
mRNA	ID=cds1203	1274402-1275052	0.5
mRNA	ID=cds3079	3278723-3279097	0.5
mRNA	ID=cds2108	2210265-2210984	0.5
mRNA	ID=cds3536	3764360-3765202	0.5
mRNA	ID=cds704	748202-748930	0.5
mRNA	ID=cds3299	3488883-3489485	0.5
mRNA	ID=cds2747	2911721-2913022	0.5
mRNA	ID=cds101	114522-115724	0.5
mRNA	ID=cds4210	4517361-4518347	0.5
mRNA	ID=cds862	922487-924763	0.5
mRNA	ID=cds687	728806-732999	0.5
mRNA	ID=cds1442	1524964-1525176	0.5
mRNA	ID=cds3401	3602416-3603012	0.5

mRNA	ID=cds138	157729-159126	0.5
mRNA	ID=cds2179	2290983-2292926	0.5
mRNA	ID=cds3916	4195739-4196803	0.5
mRNA	ID=cds2491	2632254-2633624	0.5
mRNA	ID=cds159	184257-185069	0.5
mRNA	ID=cds3418	3617215-3621450	0.5
mRNA	ID=cds487	522485-526765	0.5
mRNA	ID=cds317	336002-337549	0.5
mRNA	ID=cds739	790262-791278	0.5
other ncRNA		3193121-3193262	0.5
other ncRNA		3192745-3192887	0.5
mgeRNA	gbkey=mobile_element	566000-567257	0.4
mgeRNA	gbkey=mobile_element	1093468-1094725	0.4
mgeRNA	gbkey=mobile_element	390933-392190	0.4
mgeRNA	gbkey=mobile_element	314450-315707	0.4
mgeRNA	gbkey=mobile_element	2168195-2169452	0.4
mRNA	ID=cds1006	1093498-1094364	0.4
mRNA	ID=cds365	390963-391829	0.4
mRNA	ID=cds531	566361-567227	0.4
mRNA	ID=cds297	314811-315677	0.4
mRNA	ID=cds2074	2168556-2169422	0.4
mRNA	ID=cds2304	2433658-2434671	0.37299
mRNA	ID=cds2056	2149735-2150496	0.37299
mRNA	ID=cds2754	2921806-2922135	0.353553
mRNA	ID=cds2409	2544695-2546119	0.333333
mRNA	ID=cds2674	2833195-2835447	0.333333
mRNA	ID=cds1789	1878910-1879833	0.333333
mRNA	ID=cds3115	3314061-3315548	0.333333
mRNA	ID=cds2385	2520751-2522007	0.333333
mRNA	ID=cds259	279651-279959	0.333333
mRNA	ID=cds1948	2030408-2031103	0.333333
mRNA	ID=cds579	609477-611717	0.333333
mRNA	ID=cds324	344890-345561	0.333333
mRNA	ID=cds266	288525-289529	0.333333
mgeRNA	gbkey=mobile_element	3650059-3651253	0.272727
mgeRNA	gbkey=mobile_element	2286941-2288135	0.272727
mgeRNA	gbkey=mobile_element	1394068-1395262	0.272727
mgeRNA	gbkey=mobile_element	2064183-2065377	0.272727
mgeRNA	gbkey=mobile_element	273179-274373	0.272727
mgeRNA	gbkey=mobile_element	3363578-3364772	0.272727
mgeRNA	gbkey=mobile_element	1425623-1426818	0.272727
mgeRNA	gbkey=mobile_element	3128168-3129362	0.272727
mgeRNA	gbkey=mobile_element	687074-688268	0.272727
mgeRNA	gbkey=mobile_element	2099773-2100967	0.272727
mgeRNA	gbkey=mobile_element	573814-575008	0.272727
mRNA	ID=cds1316	1394100-1395116	0.272727
mRNA	ID=cds251	273325-274341	0.272727
mRNA	ID=cds3442	3650205-3651221	0.272727
mRNA	ID=cds3162	3363724-3364740	0.272727
mRNA	ID=cds2175	2287087-2288103	0.272727
mRNA	ID=cds1355	1425770-1426750	0.272727
mRNA	ID=cds651	687220-688236	0.272727
mRNA	ID=cds1973	2064329-2065345	0.272727
mRNA	ID=cds2015	2099919-2100935	0.272727
mRNA	ID=cds2935	3128200-3129216	0.272727
mRNA	ID=cds544	573960-574976	0.272727
mRNA	ID=cds1446	1529840-1530976	0.099384
mRNA	ID=cds3421	3622401-3623537	0.099384
mRNA	ID=cds692	736048-737184	0.099384
mRNA	ID=cds2872	3057775-3058668	0.095971
repeat_region		338981-339338	0.078432
mRNA	ID=cds3752	3999449-4000399	0.046802
mRNA	ID=cds4204	4511429-4512331	0.04631
mRNA	ID=cds1968	2060415-2061347	0.041012
repeat_region		3148576-3148830	0.039216
mRNA	ID=cds807	864352-865587	0.022196
repeat_region		3875609-3875706	0.019608
repeat_region		2712305-2712402	0.019608

repeat_region		3267744-3267841	0.019608
repeat_region		356908-356986	0.019608
repeat_region		4468404-4468491	0.019608
repeat_region		1112640-1112737	0.019608
repeat_region		2652992-2653088	0.019608
repeat_region		3772259-3772357	0.019608
repeat_region		2682139-2682236	0.019608
repeat_region		3561605-3561702	0.019608
repeat_region		430192-430289	0.019608
repeat_region		2943918-2944014	0.019608
repeat_region		3596420-3596518	0.019608
repeat_region		814808-814904	0.019608
repeat_region		2826583-2826611	0.019608
repeat_region		1550095-1550191	0.019608
repeat_region		856839-856936	0.019608
repeat_region		4125975-4126071	0.019608
repeat_region		3510533-3510622	0.019608
repeat_region		4135812-4135909	0.019608
repeat_region		714487-714584	0.019608
repeat_region		844854-844952	0.019608
repeat_region		3648735-3648834	0.019608
repeat_region		3328482-3328579	0.019608
repeat_region		831512-831609	0.019608
repeat_region		2547488-2547585	0.019608
repeat_region		3637232-3637329	0.019608
repeat_region		3674144-3674304	0.019608
repeat_region		1689449-1689545	0.019608
repeat_region		983584-983681	0.019608
repeat_region		356708-356786	0.019608
repeat_region		138699-138796	0.019608
repeat_region		609340-609437	0.019608
repeat_region		1550273-1550369	0.019608
repeat_region		2289175-2289373	0.019608
repeat_region		3068025-3068122	0.019608
repeat_region		3738987-3739084	0.019608
repeat_region		3010423-3010626	0.019608
repeat_region		3099718-3099796	0.019608
repeat_region		698648-698745	0.019608
repeat_region		898919-899016	0.019608
repeat_region		2536557-2536654	0.019608
repeat_region		706993-707157	0.019608
mRNA	ID=cds309	326485-327957	0.010879
